# Computational genome-wide survey of odorant receptors from two solitary bees *Dufourea novaeangliae* (Hymenoptera: Halictidae) and *Habropoda laboriosa* (Hymenoptera: Apidae)

**DOI:** 10.1038/s41598-017-11098-z

**Published:** 2017-09-07

**Authors:** Snehal D. Karpe, Surbhi Dhingra, Axel Brockmann, R. Sowdhamini

**Affiliations:** 1 National Centre for Biological Sciences (NCBS), Tata Institute of Fundamental Research (TIFR), Bangalore, India; 2 Institute of Bioinformatics and Applied Biotechnology (IBAB), Bangalore, India

## Abstract

Olfactory/odorant receptors (ORs) probably govern eusocial behaviour in honey bees through detection of cuticular hydrocarbons (CHCs) and queen mandibular gland pheromones (QMP). CHCs are involved in nest-mate recognition whereas QMP acts as sex pheromone for drones and as retinue pheromone for female workers. Further studies on the effect of eusociality on the evolution of ORs are hindered by the non-availability of comprehensive OR sets of solitary species. We report complete OR repertoires from two solitary bees *Dufourea novaeangliae* (112 ORs) and *Habropoda laboriosa* (151 ORs). We classify these ORs into 34 phylogenetic clades/subfamilies. Differences in the OR sets of solitary and eusocial bees are observed in individual subfamilies like subfamily 9-exon (putative CHC receptors) and L (contains putative QMP receptor group). A subfamily (H) including putative floral scent receptors is expanded in the generalist honey bees only, but not in the specialists. On the contrary, subfamily J is expanded in all bees irrespective of their degree of social complexity or food preferences. Finally, we show species-lineage specific and OR-subfamily specific differences in the putative *cis*-regulatory DNA motifs of the ORs from six hymenopteran species. Out of these, [A/G]CGCAAGCG[C/T] is a candidate master transcription factor binding site for multiple olfactory genes.

## Introduction

Honey bees are central to pollination of most flowering plants. They contribute more than $15 billion to the value of agricultural crops each year in the USA alone^1^. Wild bees are mostly solitary and also provide pollination services to up to 80% of flowering plants. Few important crops such as tomatoes, eggplants, cranberries and blueberries can only be pollinated by buzz pollinators among these species. Extrapolated from the number of blueberries pollinated by each individual, the estimated value of each *Habropoda laboriosa* bee (south-eastern blueberry bee) is $20^2^. With as much as 40% of the honey bees dying each year^1, 3^, these solitary bees are viewed as possible alternatives and must be targeted for our future research.

*H*. *laboriosa*, found in south-eastern region of USA, is one of the earliest branching species in the family Apidae^4, 5^. This soil-dwelling species is oligolectic (specialist pollinator) on blueberries (Genus Vaccinium)^6^. It is the evolutionarily closest solitary bee to the honey bees with a sequenced genome^7^. Similarly, *D*. *novaeangliae* is a soil-dwelling solitary species found in north-eastern USA, but it is an oligolege of pickerel weed *(Pontederia cordata)* ^8, 9^. It belongs to the family Halictidae and is the phylogenetically most distant bee species to the honey bees amongst all the sequenced species^7^. Due to their unique phylogenetic placement and their solitary behaviour, they can be used for comparative analysis of evolution of eusociality along with the honey bees.

The evolution of social complexity in insects is thought to be accompanied with changes in the olfactory machinery. Genes involved in perception of the chemical cues may also undergo selective evolution along with the increase in tasks that need communication among individuals of the same or another colony. The best gene family candidates to test this hypothesis are olfactory/odorant receptors (ORs). First, since they are known to undergo rapid birth and death evolution in response to the needs of each species^10, 11^ and second, they seem to have expanded in eusocial bees and ants compared to distant solitary insect orders^12–15^.

Such analysis of OR evolution across a pair of closely related solitary/eusocial species is mostly hindered by the non-availability of sequenced genomes and gene annotation pipelines that miss a fair amount of OR genes during gene prediction. Recently, the genomes of few solitary bees were sequenced^7^. We addressed the second challenge by building a semi-automated computational pipeline (as described in our previous study on *A*. *florea* ORs)^15^. In this analysis, we have introduced more distant queries and a target focussed approach for search into the solitary bee genome sequences. The identified ORs were validated through domain searches, transmembrane helix prediction (TMH) and synteny analysis. This was followed by phylogenetic reconstruction of ORs from the two solitary bees, two honey bees, an ant and a wasp. Important subfamilies/clades of ORs identified from previous literature were inspected for possible unusual trends shown by solitary bees. Finally, we also investigated presence of upstream *cis*-regulatory elements across lineages and OR subfamilies. Altogether, our analysis sheds light on the evolution of ORs and their putative regulatory elements from solitary and eusocial honey bees.

## Results

### Genome-wide survey (GWS) of ORs from two solitary bees

Our computational genome-wide survey for OR genes in the solitary bees *D*. *novaeangliae* and *H*. *laboriosa* genome resulted in the identification of total 112 putative DnOrs and 151 putative HlOrs respectively (Table 1, Supplementary Tables S1 and S2 and Supplementary Data S1 and S2).

**Table 1:** ORs identified through genome-wide survey.

Species	*Dufourea novaeangliae*	*Habropoda laboriosa*
Total number of ORs found in this study	112 (11) [91]	151 (19) [134]
Complete*	77 (5) [77]	100 (9) [99]
Partial	35 (6) [14]	51 (10) [35]
Novel ORs compared to NCBI annotations	63 (9) [44]	42 (10) [29]
Gene models that differ from NCBI annotations	33 (1) [31]	82 (9) [78]
Same Gene models as NCBI annotations	16 (1) [16]	27 (0) [27]

Numbers in parenthesis indicate pseudogenes formed by in-frame STOP codons or frame-shifts. The numbers in square brackets indicate proteins with 7tm_6 domain according to CD-search. *Indicates full proteins with both the termini and the internal exons or the ones with more than 370 amino acid length.

More than 30 genes are annotated as ‘partial’, due to absence of either termini or missing internal exons in both the species. This is either due to partial genomic scaffolds or their engagement in formation of alternative isoform with a neighbouring gene model. As the presence of alternate gene models is difficult to conclude without transcriptome data, these gene models were retained as partial and are not considered as pseudogenes, unless they possess pseudogenizing elements such as frame-shifts or in-frame STOP codons. Despite presence of such a large number of partial sequences, 80 to 90% of the total proteins from both the genomes passed 7tm_6 (characteristic of *Drosophila-*like odorant receptors) validation and majority of them show presence of six or more TMHs (Supplementary Fig. S1). More than 100 ORs were discovered at entirely new genic regions, where no gene was annotated before by NCBI annotation pipeline. Majority of the remaining gene models differ from their overlapping NCBI gene counterparts and were found to be better in terms of the presence of the 7tm_6 domain and transmembrane helices and hence were retained. 11 and 19 of the DnOrs and HlOrs were pseudogenes, respectively, and were almost equally distributed in both complete as well as partial gene models. Upon annotation of DnOrs and HlOrs, we observed that the bidirectional 1:1 orthologous relationships (as were observed between AfOrs and AmOrs^15^) were rare between these two distantly related solitary bees but 1:many or many:many relationships were more.

### Comparison of number of ORs across various insect orders

**Figure 1:**
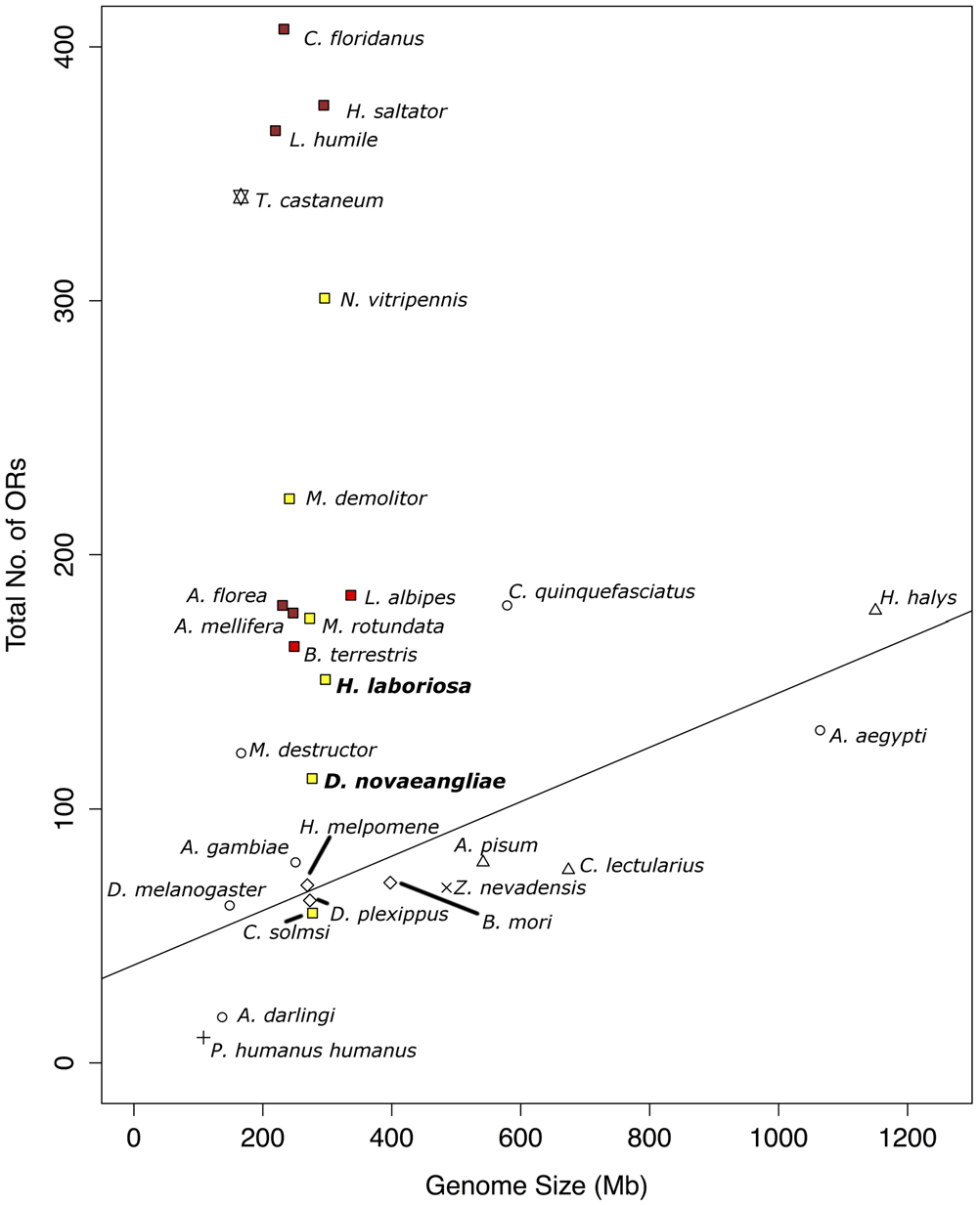
Comparison of total number of ORs and genome size for insects from various orders. Number of ORs and genome size in Mb is plotted for insects from order Diptera (circles), Lepidoptera (diamonds), Hemiptera (triangles), Pthiraptera (plus sign), Blattodea (cross sign), coleoptera (star) and Hymenoptera (filled squares). Line showing correlation between the two quantities for the first five orders is plotted (Pearson’s correlation coefficient = 0.706). Hymenopteran species are further divided into solitary (yellow), primitively eusocial (red) and advanced eusocial (brown) species. Note that most hymenopteran species lie above the line and do not follow any trend across degrees of eusociality.

We compared total number of OR genes with genome sizes across multiple insect orders (Fig. 1). The total number of ORs was correlated with the genome size with Pearson’s correlation coefficient of 0.706 for insects from orders Diptera, Lepidoptera, Hemiptera, Pthiraptera and Blattodea. Interestingly, most of the Hymenoptera and Coleoptera species possess higher number of ORs than the species in the other orders.

### Phylogenetic reconstruction of ORs

**Figure 2:**
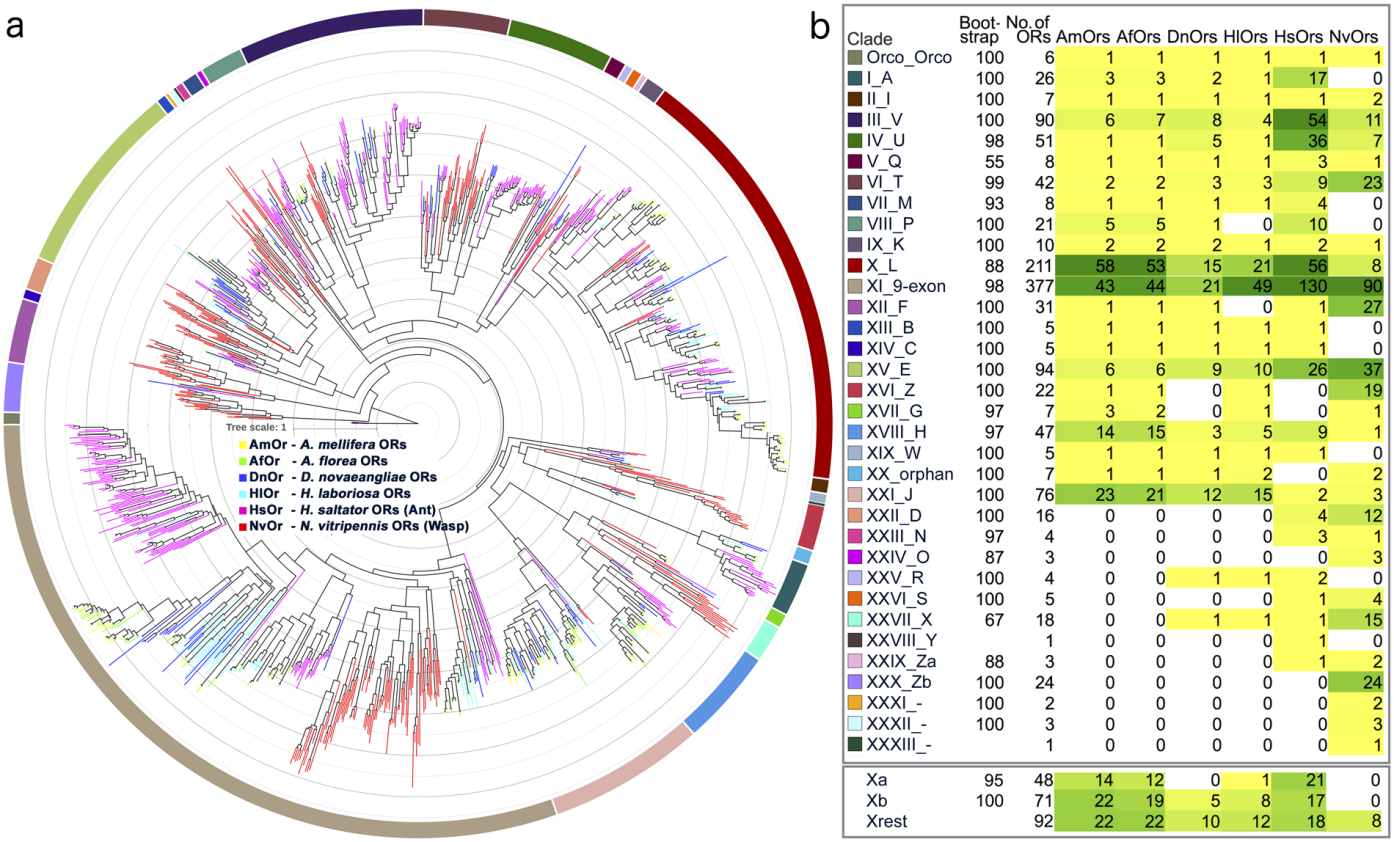
Phylogenetic reconstruction of ORs from solitary bees with other hymenopteran ORs. Phylogenetic reconstruction of ORs from *D*. *novaeangliae*, *H*. *laboriosa*, *A*. *florea* (dwarf Asian honey bee), *A*. *mellifera* (European honey bee), *H*. *saltator* (Indian jumping ant or Jerdon’s jumping ant) and *N*. *vitripennis* (parasitoid jewel wasp). (**a**) Phylogenetic tree of ORs - Branches are coloured according to the species. The clades are specified by surrounding colour strips around the phylogenetic tree. Description of these OR clades/subfamilies is given in (**b**). Clade X is further subdivided into three groups- Xa, Xb and Xrest and respective OR distribution is given at the bottom. Detailed phylogeny with OR names and bootstrap values can be found at Supplementary Fig. S3.

The phylogenetic tree of ORs from six hymenopteran species was divided into total 34 clades, including Orcos (Fig. 2a and b and Supplementary Fig. S3). We observed bootstrap support of more than 95 for most of the clades. First 30 clades follow the subfamilies defined before^13–15^. Remaining clades contain NvOrs that were previously not considered in the phylogenetic analysis but form their own clades (though less populated) and hence are called as separate clades.

First 21 OR clades were identified in both the honey bees^15^. ORs from both the solitary bees also clustered with these clades. DnOrs were missing from the subgroup Xa (part of clade X or subfamily L), XVI (subfamily Z) and XVII (subfamily G). HlOrs corresponding to clades VIII (subfamily P) and XII (subfamily F) could not be identified. Additional two sequences each from the two species clustered with clade XXV (subfamily R) and XXVII (subfamily X).

In most of the clades, the number of ORs from the solitary bees is less as compared to the honey bees. This could be expected from the comparison of the total number of ORs. However, there were notable exceptions such as clade VI (subfamily T) and clade XV (subfamily E) members. The number of HsOrs and NvOrs in the same clade XV is even greater. Clade X (subfamily L and all its subgroups) and clade XXI (subfamily J) show gradual increase in the number of ORs from *D*. *novaeangliae* to *A*. *mellifera*, with almost no OR from solitary bees clustering with the other Xa group members. Overall numbers of ORs from solitary bees were also smaller in clade XVIII (subfamily H), as compared to the honey bees and the ant species under study. The possible implications of these observations are discussed later.

### Syntenic regions

Many DnOrs and HlOrs displayed similar syntenic order, as observed for AfOrs. We focussed on a particular stretch of 57 AfOrs present on one scaffold from *A*. *florea* and its homologous regions in the two solitary bees under study (Supplementary Fig. S2). We confirmed retention of the similar syntenic order as it was observed before in AmOrs and AfOrs, but with fewer 1:1 reciprocal best hits (orthologous ORs) and with more 1:many or many:many homologous hits in similar order on the three genomes. A recent analysis on ORs shows similar trends in corbiculate bees separated over broad divergence times^16^. A close comparison of this *D*. *novaeangliae* scaffold, *H*. *laboriosa* scaffold and *A*. *florea* scaffold revealed an increase in the number of putative tandemly duplicated ORs in *A*. *florea*. Especially two regions on the scaffold consisting of AfOr4-15 and AfOr36-50 presented extensive tandem duplications. Short intergenic regions between pairs a) DnOr3 and DnOr24like_1, b) DnOr35 and DnOr53/54, c) HlOr1 and HlOr16_2PF and d) HlOr35 to HlOr51 also serve as validations that the orthologous OR genes to AmOr4-15 and AfOr36-50 are likely absent in the two solitary bees.

### Putative *cis*-regulatory regions of hymenopteran ORs

**Figure 3:**
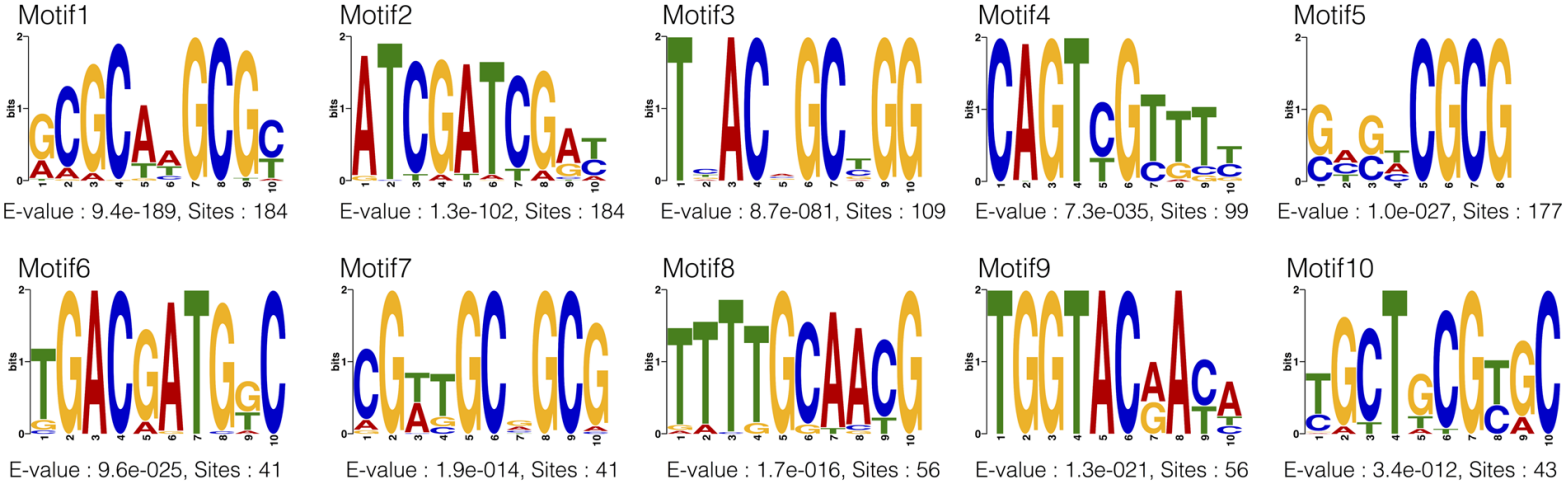
Upstream conserved DNA elements of hymenopteran ORs. Ten upstream conserved motifs modelled using MEME for ORs from six hymenopteran species are shown here. Their E-values and number of occurrences are mentioned below each motif. More information can be found at Supplementary Table S3 and Supplementary Figs S4 and S5.

We identified 10 conserved motifs from unaligned three hundred bp upstream regions of OR genes from six hymenopteran species using expectation maximization algorithm implemented in MEME v4.11.2^17^. We provided E-value cut-off of better than 10^−10^ and the total number of occurrence across all provided sequences to be more than 40 (Fig. 3, Supplementary Table S3 and Supplementary Fig. S4). Comparison of these possible OR *cis*-regulatory motifs across species unveiled differential distribution of motifs between the bee lineage and the ant *H*. *saltator* (Table 2).

**Table 2:** Distribution of putative upstream regulatory elements of ORs across bee, ant and wasp lineages.

Species	Number of ORs used in phylogeny	Motif 1	Motif 2	Motif 3	Motif 4	Motif 5	Motif 6	Motif 7	Motif 8	Motif 9	Motif 10
*A*. *mellifera*	176	**26**.**70**	**27**.**27**	1.70	17.61	6.82	2.27	1.70	6.25	3.98	7.39
*A*. *florea*	171	**28**.**07**	**27**.**49**	1.17	18.71	6.43	4.09	0.58	5.85	2.34	4.09
*D*. *novaeangliae*	92	**20**.**65**	**18**.**48**	5.43	19.57	14.13	9.78	4.35	6.52	0.00	4.35
*H*. *laboriosa*	123	**23**.**58**	**17**.**07**	6.50	20.33	17.89	4.07	9.76	0.00	2.44	3.25
*H*. *saltator*	377	9.81	13.26	**32**.**89**	5.57	18.83	13.00	12.73	16.71	20.16	16.45
*N*. *vitripennis*	301	12.96	7.64	2.33	7.64	10.96	2.66	7.31	4.32	2.66	5.98

Each cell represents the percentage of ORs from respective species with the corresponding DNA motif upstream to them.

Motifs 1, 2 and 4 were more prevalent in bee species, whereas Motif 3 is more prevalent upstream of ORs from *H*. *saltator*. Motifs 6, 7, 8, 9 and 10 were also found to be more prevalent in the ant as compared to the other species. Motif 5 was more prevalent in the two solitary bees and the ant, but it was less abundant in honey bees. None of the motifs were highly prevalent in the wasp *N*. *vitripennis*. This could be attributed to our motif detection method, as it relies on the abundance of the motifs in all the given input sequences (in this case highly dominated by the bee ORs).

We further divided our dataset into the bee lineage and the ant lineage and calculated the distribution of motifs per clade (Supplementary Tables S4 and S5 and Supplementary Fig. S5). In bees, high percentage of ORs from clade IX (subfamily K) and clade X (subfamily L) contain Motifs 1, 2 and 4 upstream. Motif 2 was highly abundant and considerably abundant upstream of bee ORs from clade VIII (subfamily P) and clade XI (subfamily 9-exon) respectively. A large percentage of ORs from clade XVII (subfamily G) also show presence of Motif 10 upstream to them.

Motif 3 was abundant upstream of ant ORs from clade III (subfamily V), clade IV (subfamily U), clade VI (subfamily T) and clade X (subfamily L). Other than Motif 3, clade III ant ORs genes also had a high percentage of Motif 7 and 8 upstream of them. In contrast, clade IV ant ORs had additional Motif 6, 8, 9 and 10. Motif 5 and 9 were also abundant upstream to ant ORs from clade VI. Motif 5 was abundant in few nearby clades as well. Other than these, few clades contain very few OR genes and hence high percentage of motif occurrence upstream of them may not be a biologically significant phenomenon.

We examined whether any of these motifs are already known to be transcription factor binding sites (TFBS). Only Motif 1 had good similarity to TFBS of a known vertebrate transcription factor called as NRF1 or ‘Nuclear Respiratory Factor 1’ with the E-value of 10^−4^.

## Discussion

### All Hymenoptera possess high number of ORs irrespective of their degree of social complexity

The number of ORs in *D*. *novaeangliae* is the least among all the bees studied for presence of ORs from fully sequenced genomes. In spite of that, the number of DnOrs (solitary halictid bee) is marginally larger (total 112) than the number of ORs found in most other insect genomes. The number of HlOrs (solitary Apidae) is considerably higher (total 151). Comparison of genome assembly quality across multiple well studied bee genomes, an ant genome and a wasp genome shows that the overall assembly quality (N50) of *D*. *novaeangliae* is very good only second best to *A*. *mellifera* (Supplementary Table 6). The assembly quality of *H*. *laboriosa* is also good in comparison with other genomes (Supplementary Table 6). Hence there is a very low probability of ORs being completely missed due to the quality of the assembly.

The solitary megachilid bee *M*. *rotundata* is reported to have a similar number of about 140 ORs (NCBI Gene database)^18^. Facultative primitive eusocial bee *E*. *mexicana* possesses 142 ORs^16^. Another primitively eusocial halictid bee *L*. *albipes*, with a colony size of only 10 bees, has around 180 ORs, and the obligate primitive eusocial Apidae *B*. *terrestris* with a colony size of about 100 workers has 164 ORs in its genome^19^. Number of ORs in the last two primitively eusocial bees are similar to that found in the advanced eusocial honey bees (around 180)^12, 14, 20, 21^. Among bees, obligate advanced eusocial stingless bee *M*. *quadrifasciata* possess the highest number of ORs (196 ORs)^16^. The advanced eusocial ants including the most basal species *H*. *saltator* have more than 300 ORs^13, 14, 22^. Both solitary endoparasitoid wasps *N*. *vitripennis* and *M*. *mediator* possess more than 200 ORs. To the best of our knowledge, among all hymenopteran species with sequenced genomes, only the highly specialised fig wasp *C*. *solmsi* possesses less than 100 ORs^14^. All antennal transcriptome based OR studies fail to capture the entire OR repertoire as shown in our previous paper^15^. To summarize, solitary, primitively eusocial and advanced eusocial hymenopteran insect species all have genomes of 200–300 Mb length, but their OR repertoires vary a lot, and our analyses show that there is no correlation between OR numbers and social life style (Fig. 1). However, there could be a correlation between social organization and number of ORs in specific subfamilies involved in intra-specific communication.

### 9-exon subfamily/clade XI is equally large in solitary bee *H*. *laboriosa* and eusocial honey bees, whereas in solitary bee *D*. *novaeangliae* the repertoire size is only half

A subfamily of ORs called as 9-exon (clade XI) has been hypothesised to be enlarged in eusocial species and suggested to be involved in nest-mate recognition via cuticular hydrocarbons^23, 24^. One argument was that these ORs are higher expressed in the workers of eusocial species^13–15, 25^. For example, OR transcripts from 9exon-alpha group were shown to be enriched on the ventral surface of the antennae of workers in the clonal raider ant *Ooceraea biroi* ^25^. The workers touch (or antennate) nest mates with this region of their antennae. In honey bees there is a correlation between worker-specific olfactory sensilla^26^ and a worker-enriched expression of 9-exon ORs (Supplementary Fig. S6)^15^. Indeed, HsOr271 strongly responds to 13,23-dimethyl-C37 (probably a fertility signal), whereas HsOr259-L2 responds to C37^27^. Thus, honey bee ORs from 9-exon-alpha group i.e. AmOr 122–139, 159, 172–177 and their homologs in other bee species, might be involved in contact based nest-mate recognition. Furthermore, many eusocial insects also use saturated and unsaturated hydrocarbons as sex-pheromones, which might have resulted in a higher variety of ORs recognizing such hydrocarbons in eusocial species as compared to their solitary relatives within a lineage^28^.

Opposing the above theory, with more solitary species under study, we do not see increased 9-exon ORs in eusocial insects alone. The number of ORs in *D*. *novaeangliae* belonging to 9-exon clade is almost half of the number of other bees, but *H*. *laboriosa* does have more ORs than any other well studied bee in this clade. Both solitary species form aggregations (*H*. *laboriosa* being more gregarious), but there is no record of active aggregation recognition behaviour by either of the two^6, 8^. Similarly the obligate primitive eusocial bumble bee possesses almost similar number of 9-exon ORs as that of the honey bees, but the obligate advanced eusocial stingless bee possesses only 26 ORs (Supplementary Table 6)^16^. Two facultative primitive eusocial orchid bees possess similar number of ORs as that of DnOrs in this subfamily (Supplementary Table 6)^16^. None of the ORs of the solitary wasp-*N*. *vitripennis* clusters with the 9-exon-alpha group but they do possess 90 ORs that group with other 9-exon ORs. Reanalysing the clustering of ORs of another solitary parasitoid wasp *Microplitis mediator* with AmOrs, we found only 13 9-exon ORs. Seven of these are male enriched (MmedOR3,4,5,7,9,19,26)^29, 30^. The question arises, why do these solitary species have lots of putative CHC sensing ORs?

There are two possibilities. Firstly, all 9-exon-alpha ORs may not be CHC responders, as the evidence for the same is mostly indirect^25^. According to this scenario, the ORs belonging to this group may respond to yet unidentified group of odorants. These odorants must not be linked to eusociality, but to other factors controlling the communication system that are different among these species. Second possibility suggesting that they are indeed CHC responders needs further analysis on the lines as discussed next.

First, the CHC receptor repertoire of any species should be dependent on the complexity of their communication system but this might not necessarily correlate with the degree of sociality. CHCs, probably first evolved as a desiccation and parasite barrier, and later acquired a function as a chemical signal for various communication purposes. CHC profiles vary a lot between species as well as within species with respect to food, age, mating status, etc. Some parasitoid wasps use CHC profiles to identify hosts or preys or use them for mimicry^31, 32^. Solitary insects can use CHCs as male attractants, probably reflected in enriched expression of male CHC ORs of *Microplitis mediator*. Overall the complex chemical ecology of solitary and social insects seems to drive the putative CHC receptor evolution than their degree of eusociality in this scenario as well. It needs further direct experimental probing for cognate ligands of these receptors to know the function of these varying repertoire sizes across species.

### Putative honey bee queen mandibular gland pheromone receptor OR group is not expanded in solitary bees

Insect lineages that have evolved unique chemical signals for specific behaviours, may harbour lineage specific OR clusters. Honey bee queen mandibular gland pheromones have been studied extensively^33^. Unlike many other insect species, the major components of this mixture are keto-acids, alcohols and esters^34^. In all honey bee species studied so far the mandibular gland pheromones are composed of the same components with different relative concentrations^35, 36^. AmOr11 was identified to bind 9-ODA the major component of the queen mandibular gland pheromone^37^. AmOr11 belongs to a subgroup of subfamily L/clade X-subgroup a (Xa) which contains *A*. *mellifera* and *A*. *florea* OR4 to 17^15^. In addition, several ORs from the subgroup Xa from subfamily L show higher RNA expression levels in drones compared to workers in *A*. *mellifera* and *A*. *florea* ^15, 37^.

**Figure 4:**
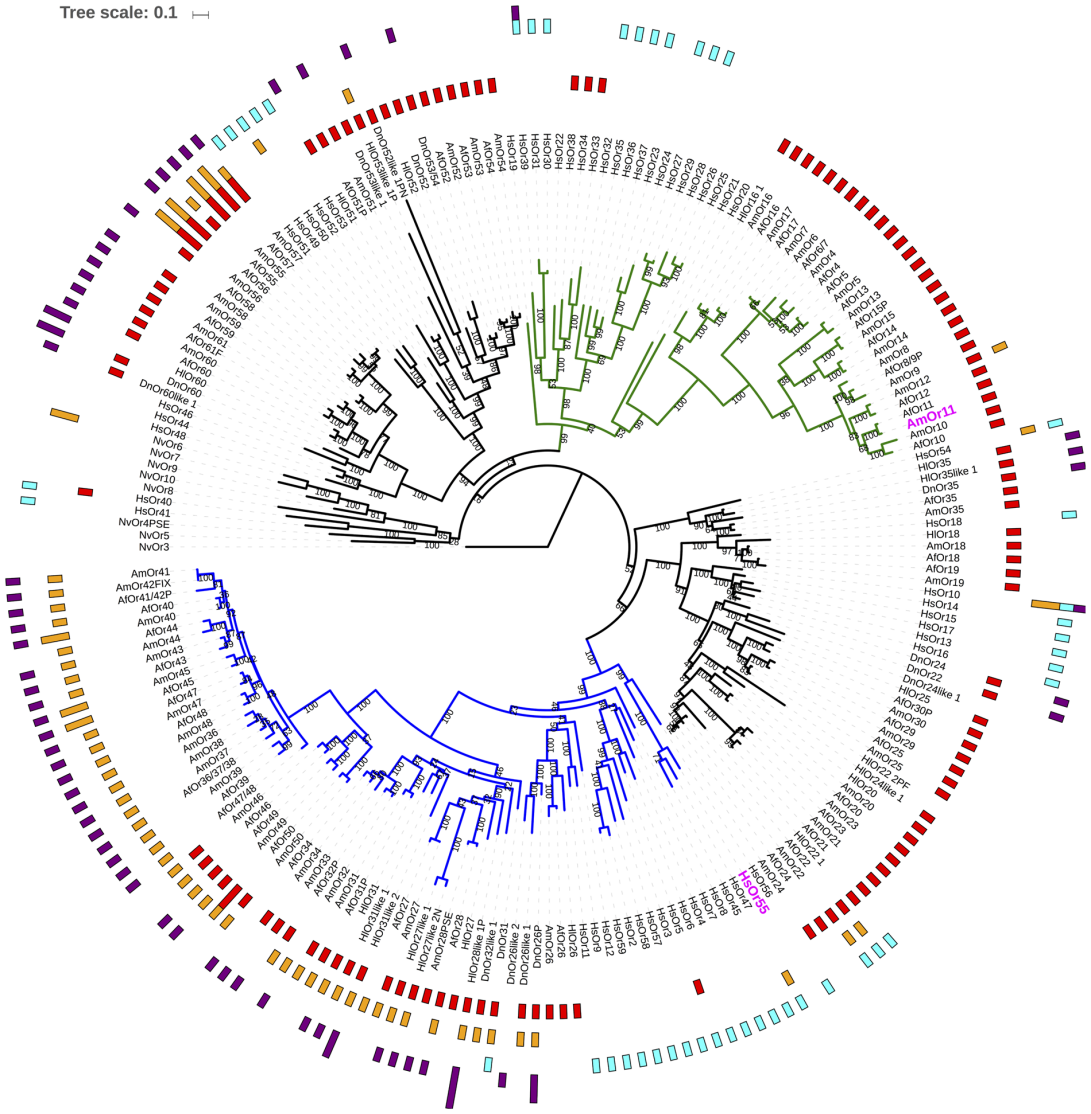
OR subfamily L with distribution of conserved upstream motifs 1 to 4. Phylogenetic tree of hymenopteran ORs from only subfamily L/clade X. Group Xa - putative pheromone receptor clade - is shown in green branches. Group Xb is shown in blue branches. *A*. *mellifera* pheromone receptor, AmOr11, for major component of queen mandibular gland pheromone (9-ODA) is highlighted in magenta colour. 4-methoxyphenylacetone receptor, HsOr55 is also shown in magenta colour. Motif 1 to 4 are shown in concentric circles from centre to periphery with colours ranging from red, orange, cyan and purple. Note that putative pheromone receptors of bee lineage possess only motif 1 upstream to them. Upstream DNA regions of bee ORs from group Xb possess motif 1, motif 2 and motif 4. Motif 1 is completely absent in a set of ORs (AmOr36-45,47,48 and corresponding homologs). On the other hand motif 3 is exclusively present upstream to *H*. *saltator* (bee) ORs. More information can be found at Supplementary Figs S4 and S5.

Closer inspection of phylogenies published for ORs from other corbiculate bee species and ant species shows an interesting trend (Supplementary Table S6)^14, 16^. The total number of ORs in obligate advanced eusocial honey bees, a stingless bee and ant species is higher than that of ORs from a bumble bee (obligate primitive eusocial) and orchid bees (solitary to primitive/weakly eusocial) in the subfamily L. For the two non-corbiculate ancestrally solitary bees studied here, the numbers of ORs in the subfamily L are almost one third of those in honey bees (Figs 2 and 4). The absence of tandem duplication of few of these ORs in solitary bees is supported by the synteny analysis. It also shows an increase in tandem duplication events of ORs in *A*. *florea* as compared to *H*. *laboriosa* and in *H*. *laboriosa* as compared to *D*. *novaeangliae*(Supplementary Fig. S2). Solitary wasp *N*. *vitripennis* has the least number of ORs in this subfamily, all of which do not belong to either the Xa or Xb subgroup.

The increase in number of ORs in subgroup Xa is even sharper with increasing degree of eusociality. Moreover, of all solitary bee ORs analyzed, only one HlOr belongs to the subgroup Xa of putative honey bee queen mandibular gland pheromone receptors (Figs 2 and 4). These findings nicely correlate with the theory of selective expansion of clades responsible for evolution of eusociality in Hymenoptera. Interestingly, there is a considerable number of ORs in *Harpegnathos saltator* that also cluster with this clade X and few of them also show male enriched expression, but they form their own group away from the ORs of the bees. These ORs could be involved in recognition of ligands that are similar to the honey bee queen mandibular gland pheromones or other male attracting sex pheromones. Interestingly HsOr36 is one of the male-enriched ant ORs from the same subgroup. It has been shown to bind to octacosane, a longer chain hydrocarbon, but currently there is no evidence for such CHCs as sex pheromones in *H*. *saltator* ^27^. Five other HsOrs from the same subgroup also displayed male-enriched expression with subthreshold (<30 spikes) response to many CHCs^27^. Hence the cognate odors for these HsOrs are possibly yet to be unearthed.

The other subgroup Xb also shows a big difference in the number of ORs between honey bees and solitary bees (Figs 2 and 4). This group contains 4-methoxyphenylacetone receptor from *H*. *saltator*, HsOr55^13^. This odorant is a component of anise essential oil which has been shown to have repellent effect on mosquitoes^38, 39^ and lethal effect on a few insect pests^40^. In contrast, anise is attractant for bees and beetles, and is often used in honey bee behavioural experiments^41, 42^. HsOr59 has been shown to be stimulated by formic acid (alarm pheromone for formicine ant), citronellol, geraniol and 2-3-butanedione^27^.

### Subfamily H, a subfamily with putative floral scent receptors, is enlarged in generalist flower visitors

AmOr151 and AmOr152 from clade XVIII (subfamily H) respond to linalool and other floral scents^43^ and most ORs from this clade are higher expressed in workers than in drones^15^. Thus this clade has been recognized as putative floral scent receptor clade probably specialised on terpenoids. Interestingly, the number of DnOrs and HlOrs belonging to this clade is very small as compared to both the honey bees (Fig. 2). Both *D*. *novaeangliae* and *H*. *laboriosa* are specialist pollinators. *H*. *laboriosa* is oligolectic on blueberry (Genus Vaccinium) in some states of USA^44^ and *D*. *novaeangliae* is oligolege of pickerel weed (*Pontederia cordata*)^8, 9^. It is possible that these specialist species do not need a variety of floral scent receptors and hence did not expand as compared to the honey bees (generalists). The ORs in this clade could be important for pollen and/or nectar scent detection.

Interestingly, AfOr155 was found to be highly abundant in males than in females (in contrast to the expectation of typical floral scent receptors to be enriched in workers), whereas two other AfOrs do show significant female enriched expression^15^. HsOr210 is a distantly related worker-enriched ant OR from the same subfamily but it gave suprathreshold (>30 spikes) response to a C32 CHC. HsOr209 responds strongly to 2,3-butanedione^27^. In the light of these contrasting discoveries, it is imperative to deorphanize the other ORs from the clade through experimental procedures.

### Previously identified bee specific clade is expanded in solitary bees as well

Clade XXI (subfamily J) was previously identified to be expanded in honey bees and orchid bees as compared to ant or wasp species^13, 15, 16, 45^. The same was observed for a bumble bee, a stingless bee (both corbiculate bees) and a halictid bee^19, 46^. This study establishes that the clade is expanded in obligate solitary bees as well (Fig. 2) and points out to their involvement in a mechanism shared by all the bees irrespective of their degree or plasticity of eusociality. Could this be an OR-subfamily for non-terpenoid floral scents? Cognate ORs for aromatic and aliphatic odours indeed tend to cluster separately from terpenoid ORs in a phylogeny of moth ORs^47^. More experimental analysis in bees will be needed to discover function of these ORs.

### Other important phylogenetic clades

Clades VI (subfamily T) and XV (subfamily E) are expanded in solitary bees, but we do not know about any of their cognate odorants. Other than Orco, the number of ORs from each bee in Clade II (subfamily I - AmOr161 and its orthologs), V (subfamily Q - AmOr160 and its orthologs), VII (subfamily M - AmOr62 and its orthologs), XIII (subfamily B - AmOr119 and its orthologs), XIV (subfamily C - AmOr116 and its orthologs), XIX (subfamily W - AmOr120 and its orthologs) is preserved. These are expressed at similar levels in both worker and drone antennae of *A*. *florea* (except AfOr120)^15^ and are possibly more ancestral and important clades for bees; again possible functions of most of these are unknown.

Recently many ORs were tested for their responsiveness to an array of CHCs^27^. HsOr188 from subfamily B was found to respond to C20 alkane which is a less abundant shorter hydrocarbon for a typical insect cuticle^27^. Homologous bee ORs from bees, AmOr119, AfOr119, DnOr119 and HlOr119 are highly likely to show affinity to the same ligand across both the sexes based on their high sequence identities, conservation of the number of clade members across bees as well as other Hymenoptera and similar levels of expression across males and females of *A*. *florea*.

In addition to above, HsOr170 (subfamily V) responded to longer chain CHCs and HsOr236 (subfamily E) was unique to respond to two even-numbered higher length hydrocarbons found rarely in insect cuticles^27^. HsOr161 (subfamily V) displayed excitatory response to ethyl acetate and inhibitory to pheromone 3-methyl-1-butanol, citronellol, citral and geraniol.

### Hymenopteran OR genes possess conserved upstream DNA elements that are species-lineage-specific and OR-subfamily specific

Analysis of *cis*-regulatory elements of insect ORs has been previously performed in only *Drosophila* to the best of our knowledge^48–50^. Since we are interested in finding conserved elements that are universal across Hymenoptera, we performed a search for possible *cis* regulatory elements across six hymenopteran species (Fig. 3).

We found that the distribution of motifs was highly dependent on the lineage of the species (bee or ant) (Table 2), as well as the subfamily/clade-identity of the downstream ORs (Supplementary Tables S4 and S5), but they do not show exactly same evolutionary pattern as that of the downstream ORs. A motif was found to be conserved at −50 to −150 upstream of translation start site in almost all bee ORs from subfamily L. This motif is called as Motif 1. It was the only motif found upstream to ORs from putative honey bee queen mandibular gland pheromone receptor group (Xa) of subfamily L. Detailed analysis of subfamily shows a gradual decrease in the abundance of Motif 1 upstream to bee ORs from group Xa to Xrest to Xb (Fig. 4). At the same time, the abundance of Motifs 2 and 4 has increased. A subset of ORs from Xb, AmOr36-48 (except 46) and their homologues in other bees seem to have replaced Motif 1 with motif 2 in almost the exact same upstream position. Motif 1 PSSM allows for many substitutions and hence it was found upstream to as many as 4000 genes out of total genes (including OR genes) from four bees. This may seem like a ubiquitous DNA element that is probably found upstream to genes due to their high GC content, but closer inspection showed that the exact 5′-ACGCAAGCGC-3′ sequence was found upstream of total 37 ORs and only around equal number of other genes from the four bees. This is substantial enrichment upstream to only OR gene family as compared to any other. Similarly 5′-GCGCAAGCGC-3′, 5′-GCGCAAGCGT-3′ and 5′-GCGCAAGCTC-3′ are enriched upstream to ORs as compared to other genes. Overall, specifically 5′-[A/G]CGCAAGCG[C/T]-3′ sequence seems to be more enriched upstream to ORs than other genes.

We compared our 10 upstream DNA motifs with the known TFBS to find any known transcription factors that might regulate these ORs. Motif 1 bears substantial similarity with the TFBS of NRF1 - Nuclear Respiratory Factor 1 (central palindromic region - 5′-CGCATGCG-3′) from vertebrate transcription factors. Known ortholog of NRF1 in *Drosophila* is Ewg or ‘Erect Wing’ and is responsible for muscle as well as neural development, but there is no direct annotation for regulation of olfaction^51^. However, it is known to regulate specification and maintenance of photoreceptor subtype R8 in *Drosophila* ^52^. We propose that similar monoallelic robust expression of one or few ORs per olfactory sensory neuron might be regulated through Ewg or another similar transcription factor that recognises Motif 1 (at least for the subfamily L of ORs). Interestingly, Motif 1 was also found upstream to genes coding for transcription regulators involved in neuronal development and differentiation including ‘acj6 - abnormal chemosensory jump 6’, a POU-domain transcription factor known to regulate odour specificities in a set of neurons^49, 53, 54^. Is Motif 1 a TFBS for a yet unknown master regulator for olfactory sensory neuron type determination? More experimental analysis is needed to support this theory.

A detailed analysis of the evolution of ORs has been performed across species that are at the two extremes of social complexity scale. The most recent common ancestor of honey bees (Apidae) and *H*. *laboriosa* (Apidae) lineage diverged from honey bees more than 80 million years ago and *D*. *novaeangliae* (Halictidae) diverged from honey bees around 120 million years ago^7^. As these bees are more closely related to each other than the wasps (which were the only obligate solitary species available for comparison before) the comparison of number of ORs across subfamilies is more meaningful and certain patterns can be derived. We identified the OR gene set from the solitary bees ancestral to two independent events of eusocial development, *D*. *novaeangliae* (112 DnOrs) and *H*. *laboriosa* (151 HsOrs). The entire OR repertoire does not show considerable expansion in eusocial insects. Instead, insects from the order Hymenoptera have a tendency for incorporating larger OR repertoires that cannot be entirely explained by their genome sizes alone. However, a subset of OR subfamilies that respond to queen/female sex pheromones may show a trend that correlates to the sociality status of the species. Examples of such clades are 9-exon (putative CHC receptors) and L (contains putative honey bee queen mandibular gland pheromone receptors) and such trend was indeed followed in the later case. Additionally, subfamily H of putative floral scent receptors is not seen to be expanded in both solitary bees, possibly due to their specialist nature. On the contrary, subfamily J, which was previously found to be expanded in the primitively to advanced eusocial bees, is also seen to be expanded in both the solitary bees, indicating their contribution to bee-specific olfactory requirements that are yet to be unearthed. We also found an array of upstream conserved elements for OR genes, which show species-lineage and OR-subfamily specific distribution, which is not exactly similar to the evolution of OR proteins themselves. These likely *cis*-regulatory elements and their combinations may control expression of hymenopteran ORs e.g. Motif 1 is likely to govern expression of multiple bee ORs from subfamily L and possibly other olfactory genes.

## Methods

### Genome-wide survey (GWS) for OR genes from *D*. *novaeangliae* and *H*. *laboriosa*

We identified OR genes from the genomes of the two solitary bees using semi-automated manual curation of sequence homology based searches. Query dataset for the search was built using previously curated OR protein sequences from closely as well as distantly related species from the insect order Hymenoptera. This includes ORs from *A*. *mellifera* ^12, 20, 21^, *A*. *florea* ^15^, *B*. *impatiens* ^19^, *M*. *rotundata* (from NCBI Gene database), *L*. *albipes* ^14^, *C*. *biroi* ^55^, *N*. *vitripennis* ^56^, *M*. *mediator* (from NCBI Gene database) and *C*. *cinctus* ^57^. Fragmented proteins (with lengths smaller than 100 amino acids) and extended erroneous proteins (with lengths longer than 600 amino acids) were removed. ORs with 7tm_6 domain (characteristic of *Drosophila*-like odorant receptors) were retained using E-value cut-off of 0.01 using batch CD-search^58, 59^. These 1249 curated OR protein queries were used to search against genomes of *D*. *novaeangliae* Version 1.1^7^ and *H*. *laboriosa* version 1.2^7^ ﻿(both downloaded from NCBI) ﻿using Exonerate Version 2.2.0 with BLOSUM62 matrix and maximum intron length of 2000^60^.

Best scoring Exonerate alignment for every unique location on the genomic scaffolds were selected, compared with annotations by NCBI and putative protein sequences for the same were extracted using in-house Perl scripts. In rare cases, our gene models were modified with the help of NCBI gene annotations to exclude pseudogenizing elements or to get better START and STOP positions. Additional Exonerate searches were performed in order to complete partial gene predictions with the help of parameters like maximum intron size of 10000 and PAM250 matrix. TBLASTN^61^ was also implemented in some cases with PAM250 and BLOSUM45 matrices, unmasking of repeat-rich regions and with relaxed gap-introduction and gap-extension penalties to ensure completeness of the gene model. Every gene model was manually checked for presence of START and STOP codons, correct intron-exon boundaries and similarity with the existing gene models from NCBI annotation release 100^62, 63^. They were further stitched/modified wherever needed and OR protein sequences were corrected. If any gene models possessed frame-shifts with respect to the most identical sequence from the queries or intermittent STOP codons even after manual curation, they were declared as pseudogenes. ORs obtained through this genome-wide survey were annotated according to their orthology with AmOrs. Perfect best bidirectional BLASTP^64^ hits were named as ‘DnOr/HlOr’ followed by respective ‘OR type/number’ from *A*. *mellifera*. If the hits were not bidirectional, the respective OR type/number was suffixed with ‘like’. If multiple sequences possessed highest identity with a single AmOr sequence, they were suffixed with ‘_’ and an incremental number. Hypothetical proteins from pseudogenes were created by introducing ‘X’ in place of STOP codon or frame-shift mutation and their names were suffixed with ‘P’. Sequences with only N or C-terminus were suffixed with ‘N’ or ‘C’ respectively. If both termini were missing or the protein was present in multiple fragments stitched together, it was suffixed ‘F’, ‘N_C’, ‘N_F’ or ‘F_C’. In case of gene models with intermittent missing amino acids, ‘Z’s were introduced in the sequence. Very distantly related sequences to AmOrs as well as AfOrs were assigned new numbers 180 and 181. The numbers of DnOrs and HlOrs were compared with ORs across multiple insect orders collected from literature sources (or rarely from NCBI Gene database)- Diptera^65–70^, Lepidoptera^71–73^, Hemiptera^74^, Pthiraptera^75^, Blattodea^76^, Coleoptera^77^ and Hymenoptera^12–15, 19, 56, 78^.

### Validation of OR gene models

Final OR protein sets from both the solitary bees as well as from *A*. *mellifera* - AmOrs, *A*. *florea* - AfOrs, *H*. *saltator* - HsOrs and *N*. *vitripennis* - NvOrs were subjected to transmembrane helix (TMH) prediction using HMMTOP^79, 80^, TMHMM^81, 82^ and PolyPhobius^83, 84^. Consensus TMH prediction was derived for each amino acid of all sequences based on support of at least two out of three methods^85^ and was compared across these datasets. Similarly sequence domain search against Pfam^86^ and CDD^59^ was performed for the same datasets and compared.

### Analysis of syntenic regions in the three bee species

Synteny of OR genes was explored for *D*. *novaeangliae*, *H*. *laboriosa* and *A*. *florea*. As most of the sequences show perfect orthology between *A*. *mellifera* and *A*. *florea* ^15^, only *A*. *florea* was chosen among the two honeybees. FASTA sequences of scaffolds containing OR genes were extracted along with their corresponding annotation files in GFF format. These were analysed using SyMAP v4.2 (Synteny Mapping and Analysis Program)^87, 88^ for syntenic blocks across genomes using default parameters. Here BLAT was performed internally with default parameters (minScore = 30, -minIdentity = 70). The default parameters for defining syntenic regions include Top N = 2 (Retain the top ‘2’ hits for every sequence region as well as all hits with score at least 80% of the second hit) and Min Dots = 7 (Minimum number of anchors required to define a syntenic clock = 7). MCScanX (adjusted MCScan algorithm for detection of synteny and collinearity)^89^ was also used for inspection of syntenic OR gene containing regions and tandem duplications within the genomes with default parameters as follows: −b = 0 (calculate both intra and inter-species collinear blocks), −k = 50 (final score = MATCH_SCORE + NUM_GAPS*GAP_PENALTY), −s = 5(MATCH_SIZE = number of genes required to call a collinear block) and −e = 1e-05 (E-value cutoff). BLASTP of ORs from the three species was performed against themselves (E-value of 10^−5^ or less) and only the best non-identical hits were provided as input to MCScanX along with the combined GFF file derived from all the three species. Largest OR gene syntenic region consisting of *A*. *florea* scaffold NW_003789703.1 (~Chromosome 2), *H*. *laboriosa* scaffold NW_017100842.1 and *D*. *novaeangliae* scaffold NW_015373891.1 were critically examined for presence of previously undetected genes.

### Phylogenetic reconstruction of ORs from six hymenopteran species

OR protein sequences from six hymenopteran species mentioned before were collected and all sequences with lengths smaller than 200 amino acids were removed. Remaining 1240 sequences (176 AmOrs, 171 AfOrs, 92 DnOrs, 123 HlOrs, 377 HsOrs, 301 NvOrs) were aligned using MAFFT v7.123b E-INS-i strategy with JTT200 matrix and 1000 iterations^90^. The resulting alignment was trimmed using trimAl^91^ ‘automated1’ option. Maximum likelihood based phylogenetic tree was reconstructed for the reduced alignment (192 alignment positions) using RAxML v7.4.2^92^ with PROTCATJTTF matrix, 100 rapid bootstraps and six olfactory receptor-coreceptor sequences as outgroup. The output of this was provided as a guide tree for the second iteration of the alignment using MAFFT. This alignment was again trimmed using trimAl option ‘gappyout’ which retained considerable (401) alignment positions. Second round of phylogenetic reconstruction was performed on the refined alignment using RAxML with similar parameters. The tree was visualized using iTOL v3^93^ and it was subdivided into 34 subfamilies/clades with the help of existing hymenopteran OR tree^13–15^.

### Analysis of putative *cis*-regulatory regions of hymenopteran ORs

The information of gene loci of DnOrs (this study), HlOrs (this study), AfOrs^15^, AmOrs^12, 20, 21^, HsOrs^13^ and NvOrs^56^ were collected. The three hundred nucleotide upstream region of all these OR genes were extracted and only the ones with lengths greater than 100 were retained using a Perl script. These were subjected to motif identification using MEME v4.11.2^17, 94^ for maximum 10 motifs of width 6 to 10 with zero or one occurrence per sequence and E-value cut-off of 10^−5^. The motifs were mapped onto the OR protein phylogenetic tree using iTOL and compared for their distribution across species and across phylogenetic superfamilies/clades. All the motifs were scanned against various existing TF databases using TOMTOM module^95^ of MEME suite. To check whether Motif1 is present upstream to only OR genes, 300 nucleotide upstream regions were collected for all the genes in the six genomes mentioned earlier and submitted to FIMO^96^, a module of the MEME suite. A separate phylogenetic tree of ORs from only clade X was built using non-guided manually trimmed alignment and distribution of Motif 1 to Motif 4 was mapped around this phylogenetic tree for better understanding of the evolution of these motifs.

All data generated or analysed during this study are included in this published article (and its Supplementary Information files).

## Electronic supplementary material

Supplementary Tables and Figures

## References

[1] Minchenkov, A. *USDA Begins Surveys to Assess Honey Bee Colony Health, Impact on Agriculture.* https://www.nass.usda.gov/Newsroom/2015/12_23_2015.php (2015).

[2] Moisset, B. & Buchmann, S. *Bee Basics An Introduction to Our Native Bees*. (USDA Forest Service and Pollinator Partnership, 2011).

[3] Berenbaum MR (2014). Bees in Crisis: Colony Collapse, Honey Laundering, and Other Problems Bee-Setting American Apiculture. Proc. Am. Philos. Soc..

[4] Cardinal S, Straka J, Danforth BN (2010). Comprehensive phylogeny of apid bees reveals the evolutionary origins and antiquity of cleptoparasitism. Proc. Natl. Acad. Sci. USA.

[5] Hedtke SM, Patiny S, Danforth BN (2013). The bee tree of life: a supermatrix approach to apoid phylogeny and biogeography. BMC Evol. Biol..

[6] Cane JH (1994). Nesting Biology and Mating Behavior of the Southeastern Blueberry Bee, Habropoda laboriosa (Hymenoptera: Apoidea). J. Kansas Entomol. Soc..

[7] Kapheim KM (2015). Genomic signatures of evolutionary transitions from solitary to group living. Science.

[8] Eickwort GC, Kukuk PF, Wesley FR (1986). The Nesting Biology of Dufourea novaeangliae (Hymenoptera: Halictidae) and the Systematic Position of the Dufoureinae Based on Behavior and Development. J. Kansas Entomol. Soc..

[9] John K. Bouseman. in *THE GREAT LAKES ENTOMOLOGIST* **19**, 203–204 (The Michigan Entomological Society, 1986).

[10] Nei M, Rooney AP (2005). Concerted and birth-and-death evolution of multigene families. Annu. Rev. Genet..

[11] Sánchez-Gracia A, Vieira FG, Rozas J (2009). Molecular evolution of the major chemosensory gene families in insects. Heredity (Edinb)..

[12] Robertson HM, Wanner KW (2006). The chemoreceptor superfamily in the honey bee, Apis mellifera: Expansion of the odorant, but not gustatory, receptor family. Genome Res..

[13] Zhou X (2012). Phylogenetic and transcriptomic analysis of chemosensory receptors in a pair of divergent ant species reveals sex-specific signatures of odor coding. PLoS Genet..

[14] Zhou X (2015). Chemoreceptor Evolution in Hymenoptera and Its Implications for the Evolution of Eusociality. Genome Biol. Evol..

[15] Karpe SD, Jain R, Brockmann A, Sowdhamini R (2016). Identification of Complete Repertoire of Apis florea Odorant Receptors Reveals Complex Orthologous Relationships with Apis mellifera. Genome Biol. Evol..

[16] Brand, P. & Ramirez, S. R. The Evolutionary Dynamics Of The Odorant Receptor Gene Family In Corbiculate Bees. *bioRxiv* (2017). 10.1093/gbe/evx149 PMC5597890 28854688

[17] Bailey TL (2009). MEME Suite: Tools for motif discovery and searching. Nucleic Acids Res..

[18] Megachile rotundata Annotation Report. https://www.ncbi.nlm.nih.gov/genome/annotation_euk/Megachile_rotundata/101/.

[19] Sadd, B. M. *et al*. The genomes of two key bumblebee species with primitive eusocial organization. *Genome Biol*. **16** (2015). 10.1186/s13059-015-0623-3 PMC4414376 25908251

[20] Smith CDR (2011). Draft genome of the globally widespread and invasive Argentine ant (Linepithema humile). Proc. Natl. Acad. Sci. USA.

[21] Smith CR (2011). Draft genome of the red harvester ant Pogonomyrmex barbatus. Proc. Natl. Acad. Sci. USA.

[22] Engsontia P, Sangket U, Robertson HM, Satasook C (2015). Diversification of the ant odorant receptor gene family and positive selection on candidate cuticular hydrocarbon receptors. BMC Res. Notes.

[23] Breed MD (1998). Recognition Pheromones of the Honey Bee. Bioscience.

[24] Breed MD, Perry S, Bjostad LB (2004). Testing the blank slate hypothesis: Why honey bee colonies accept young bees. Insectes Soc..

[25] McKenzie SK, Fetter-Pruneda I, Ruta V, Kronauer DJC (2016). Transcriptomics and neuroanatomy of the clonal raider ant implicate an expanded clade of odorant receptors in chemical communication. Proc. Natl. Acad. Sci. USA.

[26] Breed MD, Smith TA, Torres A (1992). Role of Guard Honey Bees (Hymenoptera: Apidae) in Nestmate Discrimination and Replacement of Removed Guards. Ann. Entomol. Soc. Am..

[27] Slone, J. D. *et al*. Functional characterization of odorant receptors in the ponerine ant, Harpegnathos saltator. *Proc*. *Natl*. *Acad*. *Sci*. *USA* 201704647, doi:10.1073/pnas.1704647114 (2017). 10.1073/pnas.1704647114 PMC5559025 28696298

[28] Van Oystaeyen, A. *et al*. Conserved Class of Queen Pheromones Stops Social Insect Workers from Reproducing. *Science*. **343** (2014). 10.1126/science.1244899 24436417

[29] Ma L (2014). Molecular characterization and expression profiles of olfactory receptor genes in the parasitic wasp, Microplitis mediator (Hymenoptera: Braconidae). J. Insect Physiol..

[30] Wang S-N (2015). Identification and Expression Analysis of Putative Chemosensory Receptor Genes in Microplitis mediator by Antennal Transcriptome Screening. Int. J. Biol. Sci..

[31] Kroiss J, Schmitt T, Strohm E (2009). Low level of cuticular hydrocarbons in a parasitoid of a solitary digger wasp and its potential for concealment. Entomol. Sci..

[32] Wurdack, M. *et al*. Striking cuticular hydrocarbon dimorphism in the mason wasp Odynerus spinipes and its possible evolutionary cause (Hymenoptera: Chrysididae, Vespidae). *Proc*. *R*. *Soc*. *London B Biol*. *Sci*. **282** (2015). 10.1098/rspb.2015.1777 PMC4707744 26674944

[33] Bortolotti, L. & Costa, C. *Chemical Communication in the Honey Bee Society*. *Neurobiology of Chemical Communication* (CRC Press/Taylor & Francis, 2014). 24830041

[34] Oi CA (2015). The origin and evolution of social insect queen pheromones: Novel hypotheses and outstanding problems. Bioessays.

[35] Brockmann A, Dietz D, Spaethe J, Tautz J (2006). Beyond 9-ODA: sex pheromone communication in the European honey bee Apis mellifera L. J. Chem. Ecol..

[36] Nagaraja N, Brockmann A (2009). Drones of the dwarf honey bee Apis florea are attracted to (2E)-9-oxodecenoic acid and (2E)-10-hydroxydecenoic acid. J. Chem. Ecol..

[37] Wanner KW (2007). A honey bee odorant receptor for the queen substance 9-oxo-2-decenoic acid. Proc. Natl. Acad. Sci. USA.

[38] Prajapati V, Tripathi AK, Aggarwal KK, Khanuja SPS (2005). Insecticidal, repellent and oviposition-deterrent activity of selected essential oils against Anopheles stephensi, Aedes aegypti and Culex quinquefasciatus. Bioresour. Technol..

[39] Erler F, Ulug I, Yalcinkaya B (2006). Repellent activity of five essential oils against Culex pipiens. Fitoterapia.

[40] Tunc I, Sahinkaya S (1998). Sensitivity of two greenhouse pests to vapours of essential oils. Entomol. Exp. Appl..

[41] Imai T, Maekawa M, Tsuchiya S (2002). Attractiveness of p-anisaldehyde to the varied carpet beetle, Anthrenus verbasci (L.)(Coleoptera: Dermestidae). Appl. Entomol. Zool..

[42] Frisch, K. v. *The Dance Language and Orientation of Bees*. (Cambridge, MA: Harvard Univ. Press, 1993).

[43] Claudianos C (2014). Odor memories regulate olfactory receptor expression in the sensory periphery. Eur. J. Neurosci..

[44] Cane JH, Payne JA (1988). Foraging Ecology of the Bee Habropoda laboriosa (Hymenoptera: Anthophoridae)&quot;J an Oligolege of Blueberries (Ericaceae: Vaccinium) in the Southeastern United States. Ann. Entomol. Soc. Am..

[45] Brand P (2015). Rapid evolution of chemosensory receptor genes in a pair of sibling species of orchid bees (Apidae: Euglossini). BMC Evol. Biol..

[46] Mainland JD, Li YR, Zhou T, Liu WLL, Matsunami H (2015). Human olfactory receptor responses to odorants. Sci. Data.

[47] de Fouchier A (2017). Functional evolution of Lepidoptera olfactory receptors revealed by deorphanization of a moth repertoire. Nat. Commun..

[48] Ray A, Van Der Goes Van Naters W, Shiraiwa T, Carlson JR (2007). Mechanisms of odor receptor gene choice in Drosophila. Neuron.

[49] Bai, L., Goldman, A. L. & Carlson, J. R. Positive and Negative Regulation of Odor Receptor Gene Choice in Drosophila by Acj6. *J*. *Neurosci*. **29** (2009). 10.1523/JNEUROSCI.3525-09.2009 PMC2782464 19828808

[50] Jafari S, Alenius M (2015). Cis-regulatory mechanisms for robust olfactory sensory neuron class-restricted odorant receptor gene expression in Drosophila. PLoS Genet..

[51] DeSimone SM, White K (1993). The Drosophila erect wing gene, which is important for both neuronal and muscle development, encodes a protein which is similar to the sea urchin P3A2 DNA binding protein. Mol. Cell. Biol..

[52] Hsiao H-Y, Jukam D, Johnston R, Desplan C (2013). The neuronal transcription factor erect wing regulates specification and maintenance of Drosophila R8 photoreceptor subtypes. Dev. Biol..

[53] Clyne PJ (1999). The Odor Specificities of a Subset of Olfactory Receptor Neurons Are Governed by Acj6, a POU-Domain Transcription Factor. Neuron.

[54] Bai L, Carlson JR (2010). Distinct functions of acj6 splice forms in odor receptor gene choice. J. Neurosci..

[55] Oxley PR (2014). The genome of the Clonal raider ant Cerapachys Biroi. Curr. Biol..

[56] Robertson HM, Gadau J, Wanner KW (2010). The insect chemoreceptor superfamily of the parasitoid jewel wasp Nasonia vitripennis. Insect Mol. Biol..

[57] Gress JC, Robertson HM, Weaver DK, Dlakić M, Wanner KW (2013). Odorant receptors of a primitive hymenopteran pest, the wheat stem sawfly. Insect Mol. Biol..

[58] Marchler-Bauer A, Bryant SH (2004). CD-Search: protein domain annotations on the fly. Nucleic Acids Res..

[59] Marchler-Bauer, A. *et al*. CDD: NCBI’s conserved domain database. *Nucleic Acids Res*. **43**, D222–D226 (2015). 10.1093/nar/gku1221 PMC4383992 25414356

[60] Slater GSC, Birney E (2005). Automated generation of heuristics for biological sequence comparison. BMC Bioinformatics.

[61] Gertz EM, Yu Y-K, Agarwala R, Schäffer AA, Altschul SF (2006). Composition-based statistics and translated nucleotide searches: improving the TBLASTN module of BLAST. BMC Biol..

[62] Dufourea novaeangliae Annotation Report. https://www.ncbi.nlm.nih.gov/genome/annotation_euk/Dufourea_novaeangliae/100/.

[63] Habropoda laboriosa Annotation Report. https://www.ncbi.nlm.nih.gov/genome/annotation_euk/Habropoda_laboriosa/100/.

[64] Altschul SF (1997). Gapped BLAST and PSI-BLAST: a new generation of protein database search programs. Nucleic Acids Res..

[65] Hill CA (2002). G Protein-Coupled Receptors in Anopheles gambiae. Science.

[66] Bohbot J (2007). Molecular characterization of the Aedes aegypti odorant receptor gene family. Insect Mol. Biol..

[67] Arensburger P (2010). Sequencing of Culex quinquefasciatus establishes a platform for mosquito comparative genomics. Science.

[68] Marinotti O (2013). The genome of Anopheles darlingi, the main neotropical malaria vector. Nucleic Acids Res..

[69] Andersson MN (2014). Sex- and tissue-specific profiles of chemosensory gene expression in a herbivorous gall-inducing fly (Diptera: Cecidomyiidae). BMC Genomics.

[70] Robertson HM, Warr CG, Carlson JR (2003). Molecular evolution of the insect chemoreceptor gene superfamily in Drosophila melanogaster. Proc. Natl. Acad. Sci. USA.

[71] Dasmahapatra KK (2012). Butterfly genome reveals promiscuous exchange of mimicry adaptations among species. Nature.

[72] Tanaka K (2009). Highly selective tuning of a silkworm olfactory receptor to a key mulberry leaf volatile. Curr. Biol..

[73] Zhan S, Merlin C, Boore JL, Reppert SM (2011). The monarch butterfly genome yields insights into long-distance migration. Cell.

[74] Smadja C, Shi P, Butlin RK, Robertson HM (2009). Large gene family expansions and adaptive evolution for odorant and gustatory receptors in the pea aphid, Acyrthosiphon pisum. Mol. Biol. Evol..

[75] Kirkness EF (2010). Genome sequences of the human body louse and its primary endosymbiont provide insights into the permanent parasitic lifestyle. Proc. Natl. Acad. Sci. USA.

[76] Terrapon N (2014). Molecular traces of alternative social organization in a termite genome. Nat. Commun..

[77] Engsontia P (2008). The red flour beetle’s large nose: an expanded odorant receptor gene family in Tribolium castaneum. Insect Biochem. Mol. Biol..

[78] Xiao J-H (2013). Obligate mutualism within a host drives the extreme specialization of a fig wasp genome. Genome Biol..

[79] Tusnády GE, Simon I (1998). Principles governing amino acid composition of integral membrane proteins: application to topology prediction. J. Mol. Biol..

[80] Tusnády GE, Simon I (2001). The HMMTOP transmembrane topology prediction server. Bioinformatics.

[81] Sonnhammer EL, von Heijne G, Krogh A (1998). A hidden Markov model for predicting transmembrane helices in protein sequences. Proc. Int. Conf. Intell. Syst. Mol. Biol..

[82] Krogh A, Larsson B, von Heijne G, Sonnhammer EL (2001). Predicting transmembrane protein topology with a hidden Markov model: application to complete genomes. J. Mol. Biol..

[83] Käll L, Krogh A, Sonnhammer ELL (2005). An HMM posterior decoder for sequence feature prediction that includes homology information. Bioinformatics.

[84] Käll L, Krogh A, Sonnhammer ELL (2007). Advantages of combined transmembrane topology and signal peptide prediction–the Phobius web server. Nucleic Acids Res..

[85] Nagarathnam B (2014). DOR – a Database of Olfactory Receptors – Integrated Repository for Sequence and Secondary Structural Information of Olfactory Receptors in Selected Eukaryotic Genomes. Bioinform. Biol. Insights.

[86] Finn RD (2016). The Pfam protein families database: towards a more sustainable future. Nucleic Acids Res..

[87] Soderlund C, Nelson W, Shoemaker A, Paterson A (2006). SyMAP: A system for discovering and viewing syntenic regions of FPC maps. Genome Res..

[88] Soderlund C, Bomhoff M, Nelson WM (2011). SyMAP v3.4: a turnkey synteny system with application to plant genomes. Nucleic Acids Res..

[89] Wang Y (2012). MCScanX: a toolkit for detection and evolutionary analysis of gene synteny and collinearity. Nucleic Acids Res..

[90] Katoh K, Standley DM (2013). MAFFT multiple sequence alignment software version 7: improvements in performance and usability. Mol. Biol. Evol..

[91] Capella-Gutiérrez S, Silla-Martínez JM, Gabaldón T (2009). trimAl: a tool for automated alignment trimming in large-scale phylogenetic analyses. Bioinformatics.

[92] Stamatakis A (2006). RAxML-VI-HPC: maximum likelihood-based phylogenetic analyses with thousands of taxa and mixed models. Bioinformatics.

[93] Letunic I, Bork P (2016). Interactive tree of life (iTOL) v3: an online tool for the display and annotation of phylogenetic and other trees. Nucleic Acids Res..

[94] Bailey TL, Elkan C (1994). Fitting a mixture model by expectation maximization to discover motifs in biopolymers. Proc. Int. Conf. Intell. Syst. Mol. Biol..

[95] Gupta S, Stamatoyannopoulos JA, Bailey TL, Noble WS (2007). Quantifying similarity between motifs. Genome Biol..

[96] Grant CE, Bailey TL, Noble WS (2011). FIMO: scanning for occurrences of a given motif. Bioinformatics.

