# Supplementary material for: Computational genome-wide survey of odorant receptors from two solitary bees Dufourea novaeangliae (Hymenoptera: Halictidae) and Habropoda laboriosa (Hymenoptera: Apidae)
Source: Sci Rep. 2017 Sep 7;7:10823. doi: 10.1038/s41598-017-11098-z (PMC5589748; doi:10.1038/s41598-017-11098-z)
Supplement: Supplementary file 1 — Supplementary Tables and Figures [file 41598_2017_11098_MOESM1_ESM.pdf]

Supplementary information for -

Computational genome-wide survey of odorant receptors from two solitary bees *Dufourea novaeangliae* (Hymenoptera: Halictidae) and *Habropoda laboriosa* (Hymenoptera: Apidae)

Snehal D. Karpe, Surbhi Dhingra, Axel Brockmann and R. Sowdhamini*

Table S1-DnOr information

OR name	RefSeq Scaffold	Existing Annotations	Start	Stop	Strand	Complete/Partial	Pseudo (stop codon or frameshift) or normal	Length	Exons	TMHMM no. of TMH	PolyPhobius no. of TMH	HMMTOP no. of TMH	Consensus no. of TMH	CD-search Domain/s	Pfam Domain	Corresponding GenBank Scaffold	Alternate Scaffold Name	Clade	Subfamily
DnOr1	NW_015373891.1	LOC107187462	117154	120283	+	Complete	Normal	405	5	6	6	6	6	7tm_6	7tm_6	KQ434778.1	scaffold16	IX	K
DnOr2	NW_015374014.1	LOC107188740	1366128	1385943	+	Complete	Normal	479	8	7	7	7	7	7tm_6	7tm_6	KQ434893.1	scaffold108	OrCo	OrCo
DnOr3	NW_015373891.1	LOC107187473	122964	125023	+	Complete	Normal	407	5	6	6	8	6	7tm_6	7tm_6	KQ434778.1	scaffold16	IX	K
DnOr20like_1N	NW_015374342.1	LOC107192974_M	45432	45698	-	Partial(N)	Normal	89	1	0	0	0	0	-	-	KQ435134.1	scaffold460	Xrest	L
DnOr22	NW_015373891.1	LOC107187489_M	131218	134302	+	Complete	Normal	400	5	6	6	8	6	7tm_6	7tm_6	KQ434778.1	scaffold16	Xrest	L
DnOr24	NW_015373891.1	LOC107187489_M	134989	138242	+	Complete	Normal	411	5	6	6	7	6	7tm_6	7tm_6	KQ434778.1	scaffold16	Xrest	L
DnOr24like_1	NW_015373891.1	LOC107187479	127717	130715	+	Complete	Normal	396	4	5	6	6	5	7tm_6	7tm_6	KQ434778.1	scaffold16	Xrest	L
DnOr26like_1	NW_015373891.1	LOC107187498	139682	142618	+	Complete	Normal	405	5	5	6	7	6	7tm_6	7tm_6	KQ434778.1	scaffold16	Xb	L
DnOr26like_2	NW_015373891.1	LOC107187549	161606	163977	+	Complete	Normal	369	5	3	7	4	4	7tm_6	-	KQ434778.1	scaffold16	Xb	L
DnOr26P	NW_015373891.1	LOC107187559_M	166914	169246	+	Complete	Pseudo	401	5	4	7	6	6	7tm_6	7tm_6	KQ434778.1	scaffold16	Xb	L
DnOr31	NW_015373891.1	LOC107187508	144007	146024	+	Complete	Normal	406	5	5	6	8	6	7tm_6	7tm_6	KQ434778.1	scaffold16	Xb	L
DnOr32like_1	NW_015373891.1	LOC107187519	147079	150092	+	Complete	Normal	403	5	5	6	7	6	7tm_6	7tm_6	KQ434778.1	scaffold16	Xb	L
DnOr35	NW_015373891.1	LOC107187530_M	151542	153327	+	Complete	Normal	413	5	6	6	6	6	7tm_6	7tm_6	KQ434778.1	scaffold16	Xrest	L
DnOr52	NW_015373891.1	LOC107187559_M	170728	174451	+	Complete	Normal	411	5	6	6	8	7	7tm_6	7tm_6	KQ434778.1	scaffold16	Xrest	L
DnOr52like_1PN	NW_015373891.1	New	176121	178685	+	Partial(N)	Pseudo	310	2	4	6	5	5	7tm_6	7tm_6	KQ434778.1	scaffold16	Xrest	L
DnOr53/54	NW_015373891.1	LOC107187539_M	155513	159575	+	Complete	Normal	411	5	6	6	6	6	7tm_6	7tm_6	KQ434778.1	scaffold16	Xrest	L
DnOr53like_1	NW_015373891.1	New	180434	183441	+	Complete	Normal	411	5	6	6	9	6	7tm_6	7tm_6	KQ434778.1	scaffold16	Xrest	L
DnOr60	NW_015373891.1	LOC107187570	190140	193554	+	Complete	Normal	409	6	6	6	9	6	7tm_6	7tm_6	KQ434778.1	scaffold16	Xrest	L
DnOr60like_1	NW_015373891.1	New	185312	189168	+	Complete	Normal	411	6	6	6	7	6	7tm_6	7tm_6	KQ434778.1	scaffold16	Xrest	L
DnOr62P	NW_015374430.1	LOC107193058	522711	527278	+	Complete	Pseudo	395	4	2	8	7	7	7tm_6	7tm_6	KQ435180.1	scaffold493	VII	M
DnOr64	NW_015374430.1	LOC107193059	528109	531482	+	Complete	Normal	394	4	6	9	7	8	7tm_6	7tm_6	KQ435180.1	scaffold493	VIII	P
DnOr68	NW_015374011.1	LOC107188516	1208787	1211129	-	Complete	Normal	376	5	6	7	7	7	7tm_6	7tm_6	KQ434890.1	scaffold133	XV	E
DnOr69	NW_015374011.1	New	1204855	1207636	-	Complete	Normal	370	5	6	7	7	7	7tm_6	7tm_6	KQ434890.1	scaffold133	XV	E
DnOr70like_1	NW_015374011.1	New	1201788	1203972	-	Complete	Normal	366	5	6	7	5	6	7tm_6	7tm_6	KQ434890.1	scaffold133	XV	E
DnOr71	NW_015374011.1	LOC107188540_M	1194771	1197575	-	Complete	Normal	369	5	6	7	7	7	7tm_6	7tm_6	KQ434890.1	scaffold133	XV	E
DnOr71like_1	NW_015374011.1	LOC107188515_M	1190614	1193040	-	Complete	Normal	369	5	5	7	5	6	7tm_6	7tm_6	KQ434890.1	scaffold133	XV	E
DnOr71like_2	NW_015374011.1	New	1198844	1201021	-	Complete	Normal	374	5	5	6	7	6	7tm_6 and DUF832	7tm_6	KQ434890.1	scaffold133	XV	E
DnOr71like_3	NW_015374011.1	LOC107188514_M	1187883	1189777	-	Complete	Normal	370	5	6	7	7	7	7tm_6	7tm_6	KQ434890.1	scaffold133	XV	E
DnOr71like_4	NW_015374011.1	LOC107188513_M	1180523	1183067	-	Complete	Normal	371	5	5	7	7	7	7tm_6	7tm_6	KQ434890.1	scaffold133	XV	E
DnOr73	NW_015374011.1	LOC107188539_M	1183689	1186388	-	Complete	Normal	365	5	4	7	6	6	7tm_6	7tm_6	KQ434890.1	scaffold133	XV	E
DnOr77N	NW_015374018.1	New	2494	5241	-	Partial(N)	Normal	199	3	4	5	5	5	-	-	KQ434897.1	scaffold140	XXI	J
DnOr80like_1	NW_015374206.1	New	45945	67572	+	Complete	Normal	403	6	8	6	8	8	7tm_6	7tm_6	KQ435047.1	scaffold328	XXI	J
DnOr80like_2PC	NW_015373951.1	New	530697	535663	+	Partial(nearC)	Pseudo	275	3	5	6	6	5	7tm_6	7tm_6	KQ434836.1	scaffold79	XXI	J
DnOr84	NW_015373917.1	LOC107194240_M	1056615	1061057	+	Complete	Normal	401	7	6	6	7	6	7tm_6	7tm_6	KQ434803.1	scaffold28	XXI	J
DnOr84like_1C	NW_015374018.1	New	36586	40412	-	Partial(C)	Normal	349	6	5	7	6	7	7tm_6	7tm_6	KQ434897.1	scaffold140	XXI	J
DnOr86like_1N	NW_015373917.1	LOC107194240_M	1054583	1055254	+	Partial(nearN)	Normal	153	2	2	3	3	3	-	-	KQ434803.1	scaffold28	XXI	J
DnOr87	NW_015374018.1	New	29127	34411	-	Complete	Normal	404	6	6	7	8	7	7tm_6	7tm_6	KQ434897.1	scaffold140	XXI	J
DnOr87like_1	NW_015374065.1	LOC107190299_M	58880	80294	+	Complete	Normal	427	6	4	7	8	7	7tm_6	7tm_6	KQ434940.1	scaffold191	XXI	J
DnOr90	NW_015373951.1	LOC107186183_M	499470	520578	+	Complete	Normal	428	6	6	6	9	6	7tm_6	7tm_6	KQ434836.1	scaffold79	XXI	J
DnOr90like_1	NW_015373951.1	New	469182	490668	+	Complete	Normal	409	6	6	6	7	6	7tm_6	7tm_6	KQ434836.1	scaffold79	XXI	J
DnOr95	NW_015374018.1	LOC107188903	17088	27021	-	Complete	Normal	407	6	8	7	10	8	7tm_6	7tm_6	KQ434897.1	scaffold140	XXI	J
DnOr95like_1	NW_015374065.1	LOC107190300_M	82783	90582	+	Complete	Normal	388	6	6	6	8	7	7tm_6	7tm_6	KQ434940.1	scaffold191	XXI	J
DnOr95like_2	NW_015374018.1	New	6414	12242	-	Complete	Normal	412	6	7	8	8	9	7tm_6	7tm_6	KQ434897.1	scaffold140	XXI	J
DnOr95like_3C	NW_015374065.1	LOC107190298_M	53458	56472	+	Partial(C)	Normal	247	3	0	5	4	5	7tm_6	7tm_6	KQ434940.1	scaffold191	XXI	J
DnOr105like_1C	NW_015373997.1	New	2853073	2856672	-	Partial(C)	Normal	342	8	6	5	6	2	7tm_6	7tm_6	KQ434878.1	scaffold109	XI	9-exon
DnOr105like_2C	NW_015373997.1	New	2851473	2853321	-	Partial(nearC)	Normal	59	2	0	0	0	0	-	-	KQ434878.1	scaffold109	XI	9-exon
DnOr107like_1N	NW_015374017.1	New	289	505	+	Partial(N)	Normal	72	1	1	1	1	1	-	-	KQ434896.1	scaffold141	XI	9-exon
DnOr108like_1N	NW_015373997.1	New	2892125	2892349	-	Partial(N)	Normal	75	1	2	1	2	2	-	-	KQ434878.1	scaffold109	XI	9-exon
DnOr111like_2N	NW_015374170.1	New	22997	22845	+	Partial(N)	Normal	49	1	0	2	1	1	-	-	LGHO01003100.1	scaffold301	XI	9-exon
DnOr112like_1N	NW_015377871.1	New	496	639	+	Partial(N)	Normal	48	1	0	2	1	1	-	-	LGHO01007606.1	C1087384	XI	9-exon
DnOr113F_C	NW_015374115.1	New	1306526	1310141	+	Partial(Fragment + C)	Normal	354	8	5	8	6	8	7tm_6	7tm_6	KQ434978.1	scaffold221	XI	9-exon
DnOr114	NW_015374150.1	LOC107191511	1967096	1969600	+	Complete	Normal	372	6	6	7	7	7	7tm_6	7tm_6	KQ435007.1	scaffold228	VI	T
DnOr114like_1	NW_015374150.1	LOC107191510_M	1962672	1965863	+	Complete	Normal	370	6	6	6	8	6	Glycosyltransferase_GTB_type	7tm_6	KQ435007.1	scaffold228	VI	T
DnOr115	NW_015374150.1	LOC107191691_M	1958965	1961118	+	Complete	Normal	391	6	6	7	8	7	7tm_6	7tm_6	KQ435007.1	scaffold228	VI	T
DnOr116	NW_015374315.1	LOC107192809_M	26358	28178	+	Complete	Normal	415	6	5	6	7	6	7tm_6 and PH-like	7tm_6	KQ435119.1	scaffold445	XIV	C
DnOr117	NW_015373910.1	LOC107193904	3446949	3449158	+	Complete	Normal	403	5	4	6	5	5	7tm_6	7tm_6	KQ434796.1	scaffold21	XX	orphan
DnOr118	NW_015373888.1	New	1488244	1492312	+	Complete	Normal	381	5	6	6	7	6	7tm_6	7tm_6	KQ434775.1	scaffold18	III	V

Table S1-DnOr information

DnOr119	NW_015373918.1	LOC107194401_M	2284217	2285876	-	Complete	Normal	376	6	5	7	6	6	7tm_6	7tm_6	KQ434804.1	scaffold39	XIII	B
DnOr120	NW_015373910.1	LOC107193957_M	7294629	7296403	-	Complete	Normal	370	8	7	7	7	7	7tm_6	7tm_6	KQ434796.1	scaffold21	XIX	W
DnOr121	NW_015373890.1	New	3771181	3774679	-	Complete	Normal	385	7	6	6	7	7	7tm_6	7tm_6	KQ434777.1	scaffold14	IV	U
DnOr121like_1	NW_015373905.1	New	1324325	1329776	-	Complete	Normal	372	6	6	6	7	7	7tm_6	7tm_6	KQ434791.1	scaffold24	IV	U
DnOr121like_2	NW_015373905.1	New	1332610	1340374	+	Complete	Normal	379	6	7	7	7	7	7tm_6	7tm_6	KQ434791.1	scaffold24	IV	U
DnOr121like_3	NW_015373905.1	New	529407	531644	-	Complete	Normal	375	6	6	8	7	7	7tm_6	7tm_6	KQ434791.1	scaffold24	IV	U
DnOr121like_4	NW_015373905.1	New	533111	537059	-	Complete	Normal	375	6	6	8	7	7	7tm_6	7tm_6	KQ434791.1	scaffold24	IV	U
DnOr121like_5F	NW_015377800.1	New	180	501	+	Partial(Fragment)	Normal	80	2	2	2	2	2	-	-	LGHO01007535.1	C1075080	IV	U
DnOr122like_1N_C	NW_015373997.1	New	2875823	2880516	-	Partial(N + C)	Normal	268	6	0	6	3	6	7tm_6	-	KQ434878.1	scaffold109	XI	9-exon
DnOr130	NW_015373997.1	New	2885943	2889628	-	Complete	Normal	391	9	7	9	9	9	7tm_6	7tm_6	KQ434878.1	scaffold109	XI	9-exon
DnOr130like_1	NW_015373997.1	New	2892731	2897414	-	Complete	Normal	359	8	6	7	5	6	7tm_6	7tm_6	KQ434878.1	scaffold109	XI	9-exon
DnOr130like_2C	NW_015373997.1	New	2920305	2923180	-	Partial(C)	Normal	306	7	4	5	5	5	7tm_6	7tm_6	KQ434878.1	scaffold109	XI	9-exon
DnOr130like_3	NW_015373997.1	New	2881548	2884629	-	Complete	Normal	348	7	8	10	9	9	7tm_6	7tm_6	KQ434878.1	scaffold109	XI	9-exon
DnOr130like_4	NW_015373997.1	New	2900495	2907782	-	Complete	Normal	364	9	7	8	7	7	7tm_6	7tm_6	KQ434878.1	scaffold109	XI	9-exon
DnOr130like_5	NW_015373997.1	New	2909869	2914957	-	Complete	Normal	352	8	6	7	7	6	7tm_6	7tm_6	KQ434878.1	scaffold109	XI	9-exon
DnOr130like_6PN_PC	NW_015373997.1	New	2843083	2845555	-	Partial(N + C)	Pseudo	324	8	5	7	5	5	7tm_6	7tm_6	KQ434878.1	scaffold109	XI	9-exon
DnOr130like_7N_C	NW_015373997.1	New	2847630	2851207	-	Partial(nearN + C)	Normal	272	6	4	5	6	5	7tm_6	-	KQ434878.1	scaffold109	XI	9-exon
DnOr132like_1C	NW_015373997.1	New	2814915	2816299	-	Partial(C)	Normal	125	3	2	2	2	2	-	-	KQ434878.1	scaffold109	XI	9-exon
DnOr133like_1N	NW_015374089.1	New	5107	5250	-	Partial(N)	Normal	79	2	0	1	1	1	-	-	KQ434959.1	scaffold215	XI	9-exon
DnOr133like_2N	NW_015374309.1	New	6061	6832	+	Partial(N)	Normal	79	2	0	1	1	1	-	-	KQ435115.1	scaffold435	XI	9-exon
DnOr135like_1PF	NW_015377765.1	New	467	892	-	Partial(Fragment)	Pseudo	142	1	3	3	3	3	-	-	LGHO01007500.1	C1069722	XI	9-exon
DnOr138C	NW_015373997.1	New	2868850	2873045	-	Partial(C)	Normal	343	5	3	9	7	6	-	-	KQ434878.1	scaffold109	XI	9-exon
DnOr140TRA	NW_015374170.1	New	2086	17885	Trans	Complete	Normal	396	9	7	6	5	7	7tm_6	7tm_6	LGHO01003100.1	scaffold301	XI	9-exon
DnOr140TRAlike_1PN	NW_015374259.1	New	856336	865950	Trans	Complete(N)	Pseudo	389	7	4	6	4	4	7tm_6	7tm_6	KQ435083.1	scaffold372	XI	9-exon
DnOr140TRAlike_2	NW_015374089.1	New	5107	17278	Trans	Complete	Normal	404	9	8	6	8	7	7tm_6	7tm_6	KQ434959.1	scaffold215	XI	9-exon
DnOr140TRAlike_3N_3C	NW_015374309.1	New	6689	18042	Trans	Partial(N + C)	Normal	362	8	4	7	7	6	7tm_6	7tm_6	KQ435115.1	scaffold435	XI	9-exon
DnOr142	NW_015373918.1	LOC107194383_M	1583183	1585815	+	Complete	Normal	401	6	6	9	7	7	7tm_6	7tm_6	KQ434804.1	scaffold39	XVIII	H
DnOr146	NW_015373918.1	LOC107194384_M	1586759	1589789	+	Complete	Normal	378	6	5	9	8	9	7tm_6	7tm_6	KQ434804.1	scaffold39	XVIII	H
DnOr146like_1	NW_015373918.1	LOC107194384_M	1592743	1596188	+	Complete	Normal	406	6	5	9	6	7	7tm_6	-	KQ434804.1	scaffold39	XVIII	H
DnOr151N	NW_015374342.1	New	975	1193	-	Partial(N)	Normal	73	1	0	0	0	0	-	-	KQ435134.1	scaffold460	XVIII	H
DnOr160	NW_015373964.1	LOC107186675_M	1924601	1929656	+	Complete	Normal	396	6	6	6	7	6	7tm_6	7tm_6	KQ434849.1	scaffold84	V	Q
DnOr161	NW_015373923.1	LOC107194935_M	1412327	1415315	-	Complete	Normal	377	7	5	6	9	6	7tm_6	7tm_6	KQ434809.1	scaffold8	II	I
DnOr163	NW_015373888.1	New	1481149	1486089	+	Complete	Normal	380	6	6	6	5	6	7tm_6	7tm_6	KQ434775.1	scaffold18	III	V
DnOr165	NW_015374445.1	New	105361	109845	-	Complete	Normal	381	6	5	6	7	6	7tm_6	7tm_6	LGHO01004027.1	scaffold579	III	V
DnOr165like_1	NW_015374445.1	New	111458	116358	-	Complete	Normal	377	6	6	6	7	6	7tm_6	7tm_6	LGHO01004027.1	scaffold579	III	V
DnOr165like_2	NW_015373888.1	LOC107194983_M	1508857	1514983	+	Complete	Normal	377	6	6	6	6	6	7tm_6	7tm_6	KQ434775.1	scaffold18	III	V
DnOr165like_3C	NW_015374445.1	New	121854	126691	-	Partial(C)	Normal	320	6	5	6	6	6	7tm_6	7tm_6	LGHO01004027.1	scaffold579	III	V
DnOr165like_4F	NW_015377469.1	New	5	121	-	Partial(Fragment)	Normal	39	1	0	0	0	0	-	-	LGHO01007204.1	C1039794	III	V
DnOr166like_1C	NW_015374603.1	LOC107193202_M	3	3849	+	Partial(C)	Normal	259	5	4	5	5	5	7tm_6	7tm_6	LGHO01004261.1	scaffold741	III	V
DnOr166like_2PF	NW_015374603.1	New	5089	6415	+	Partial(Fragment)	Pseudo	96	2	0	1	1	1	-		LGHO01004261.1	scaffold741	III	V
DnOr166like_3C	NW_015374445.1	New	102823	103584	-	Partial(C)	Normal	69	2	0	1	2	1	-	7tm_6	LGHO01004027.1	scaffold579	III	V
DnOr166like_4C	NW_015374445.1	New	119043	119415	-	Partial(C)	Normal	53	2	0	0	0	0	-	-	LGHO01004027.1	scaffold579	III	V
DnOr167	NW_015373888.1	LOC107194983_M	1495911	1507636	+	Complete	Normal	385	6	5	6	6	5	7tm_6	7tm_6	KQ434775.1	scaffold18	III	V
DnOr169	NW_015374073.1	LOC107190427	1006581	1009980	-	Complete	Normal	389	5	6	9	8	7	7tm_6 and PKc_like	7tm_6	KQ434946.1	scaffold199	I	A
DnOr169like_1	NW_015374073.1	LOC107190428_M	1012438	1016525	-	Complete	Normal	390	5	6	6	9	6	7tm_6	7tm_6	KQ434946.1	scaffold199	I	A
DnOr171	NW_015373998.1	LOC107188077_M	1048276	1056949	-	Complete	Normal	388	5	6	7	8	7	7tm_6	7tm_6	KQ434879.1	scaffold115	XII	F
DnOr172	NW_015373997.1	New	2820156	2824189	-	Complete	Normal	395	8	6	6	7	6	7tm_6	7tm_6	KQ434878.1	scaffold109	XI	9-exon
DnOr172like_1P	NW_015374537.1	New	83974	87465	+	Complete	Pseudo	379	9	6	7	4	6	7tm_6	7tm_6	KQ435207.1	scaffold665	XI	9-exon
DnOr172like_2	NW_015373997.1	New	2837634	2841175	-	Complete	Normal	357	7	5	7	6	6	7tm_6	7tm_6	KQ434878.1	scaffold109	XI	9-exon
DnOr172like_3F	NW_015373997.1	New	2819343	2822006	-	Partial(Fragment)	Normal	176	3	2	3	3	3	-	-	KQ434878.1	scaffold109	XI	9-exon
DnOr172like_4PN	NW_015373997.1	New	2835213	2835667	-	Partial(N)	Pseudo	152	1	2	3	4	3	-	-	KQ434878.1	scaffold109	XI	9-exon
DnOr174	NW_015373997.1	New	2825695	2830588	-	Complete	Normal	372	8	6	8	8	8	7tm_6	7tm_6	KQ434878.1	scaffold109	XI	9-exon
DnOr174like_1N_C	NW_015373997.1	New	2858384	2866249	-	Partial(N + C)	Normal	345	5	6	6	6	5	7tm_6	7tm_6	KQ434878.1	scaffold109	XI	9-exon
DnOr180	NW_015373918.1	New	1596740	1599167	+	Complete	Normal	383	6	6	8	7	8	7tm_6	7tm_6	KQ434804.1	scaffold39	XXVII	X
DnOr181PC	NW_015373988.1	New	493655	496809	-	Complete(C)	Pseudo	379	5	5	5	6	5	7tm_6	7tm_6	KQ434869.1	scaffold89	XXV	R

Column C refers to known NCBI genes at the current OR positions. If there was none – it is written as 'New'. If the current NCBI annotation had to be modified to make it better, the gene name is followed by '_M'.
In column F, 'Trans' denotes trans-splicing.
Complete in column G refers to completeness of gene (>=370aa or with both termini). 'Fragment' or 'F' means our current annotation does not have both N as well as C terminus.
Subfamilies in column T refer to already known subfamilies of ORs from Zhou *et al.* 2012, Zhou *et al*. 2015 and Karpe *et al.* 2016.

Table S2-HlOr information

OR name	RefSeq Scaffold	Existing Annotations	Start	Stop	Strand	Complete/Partial	Pseudo (stop codon or frameshift) or normal	Length	Exons	TMHMM no. of TMH	PolyPhobius no. of TMH	HMMTOP no. of TMH	Consensus no. of TMH	CD-search Domain	Pfam Domain	Corresponding GenBank Scaffold	Alternate Scaffold Name	Clade	Subfamily
HlOr1	NW_017100842.1	LOC108577300	194109	198633	+	Complete	Normal	404	5	6	6	6	6	7tm_6	7tm_6	KQ414889.1	scaffold697	IX	K
HlOr2	NW_017100385.1	LOC108575019_M	580208	591043	-	Complete	Normal	480	8	7	7	7	7	7tm_6	7tm_6	KQ414731.1	scaffold204	OrCo	OrCo
HlOr16_1	NW_017100842.1	LOC108577301_M	204195	207610	+	Complete	Normal	390	5	5	6	11	7	7tm_6	7tm_6	KQ414889.1	scaffold697	Xa	L
HlOr16_2PF	NW_017100842.1	New	200177	200452	+	Partial(Fragment)	Pseudo	70	2	0	1	0	0	-	-	KQ414889.1	scaffold697	Xa	L
HlOr18	NW_017100842.1	LOC108577302_M	208186	212087	+	Complete	Normal	404	5	6	6	7	6	7tm_6	7tm_6	KQ414889.1	scaffold697	Xrest	L
HlOr20	NW_017100842.1	LOC108577304	217106	219803	+	Complete	Normal	411	5	6	6	7	6	7tm_6	7tm_6	KQ414889.1	scaffold697	Xrest	L
HlOr22_1	NW_017100842.1	LOC108577305_M	221309	224703	+	Complete	Normal	410	5	6	6	9	6	7tm_6	7tm_6	KQ414889.1	scaffold697	Xrest	L
HlOr22_2PF	NW_017100842.1	New	200503	203155	+	Partial(Fragment)	Pseudo	259	3	3	4	4	3	-	-	KQ414889.1	scaffold697	Xrest	L
HlOr24like_1	NW_017100842.1	LOC108577303	212804	215136	+	Complete	Normal	411	5	6	6	8	6	7tm_6	-	KQ414889.1	scaffold697	Xrest	L
HlOr25	NW_017100842.1	LOC108577305_M	226862	230521	+	Complete	Normal	411	5	6	6	7	6	7tm_6	7tm_6	KQ414889.1	scaffold697	Xrest	L
HlOr26	NW_017100842.1	LOC108577306_M	232412	235269	+	Complete	Normal	402	5	3	7	8	7	7tm_6	7tm_6	KQ414889.1	scaffold697	Xb	L
HlOr26like_1C	NW_017125946.1	New	86	250	+	Partial(C)	Normal	54	1	0	1	1	1	-	-	LHQN01048470.1	C5844575	Xb	L
HlOr26like_2C	NW_017106677.1	New	75	236	-	Partial(C)	Normal	54	1	0	1	1	1	-	-	LHQN01029187.1	scaffold6803	Xb	L
HlOr27	NW_017100842.1	LOC108577337	256602	258741	+	Complete	Normal	406	5	6	6	7	6	7tm_6	7tm_6	KQ414889.1	scaffold697	Xb	L
HlOr27like_1	NW_017100842.1	LOC108577307	236799	238924	+	Complete	Normal	405	5	6	7	7	7	7tm_6	7tm_6	KQ414889.1	scaffold697	Xb	L
HlOr27like_2N	NW_017106259.1	LOC108570037	127	1124	-	Partial(N)	Normal	309	2	5	5	5	5	7tm_6	7tm_6	LHQN01028641.1	scaffold6378	Xb	L
HlOr27like_3C	NW_017105734.1	LOC108570053_M	4086	5607	-	Partial(C)	Normal	146	5	2	2	3	2	7tm_6	7tm_6	LHQN01027980.1	scaffold5844	Xb	L
HlOr27like_4C	NW_017100842.1	New	254072	254567	+	Partial(C)	Normal	105	3	1	1	2	1	7tm_6	7tm_6	KQ414889.1	scaffold697	Xb	L
HlOr28like_1P	NW_017100842.1	LOC108577343_M	259722	262204	+	Complete	Pseudo	406	5	6	6	8	6	7tm_6	7tm_6	KQ414889.1	scaffold697	Xb	L
HlOr28like_2C	NW_017100842.1	LOC108577308_M	243548	244951	+	Partial(C)	Normal	125	4	1	1	2	1	7tm_6	7tm_6	KQ414889.1	scaffold697	Xb	L
HlOr31	NW_017100842.1	LOC108577310	266587	273302	+	Complete	Normal	406	5	5	6	7	6	7tm_6	7tm_6	KQ414889.1	scaffold697	Xb	L
HlOr31like_1	NW_017100842.1	LOC108577311_M	274330	277842	+	Complete	Normal	407	5	6	6	9	6	7tm_6	7tm_6	KQ414889.1	scaffold697	Xb	L
HlOr31like_2	NW_017100842.1	LOC108577309	262811	265013	+	Complete	Normal	402	5	6	6	7	6	7tm_6	7tm_6	KQ414889.1	scaffold697	Xb	L
HlOr31like_3PC	NW_017100842.1	New	281170	283561	+	Partial(C)	Pseudo	151	5	1	1	2	1	-	-	KQ414889.1	scaffold697	Xb	L
HlOr34like_1F	NW_017106464.1	LOC108578310_M	424	867	+	Partial(Fragment)	Normal	148	1	3	3	3	3	-	-	LHQN01028951.1	scaffold6588	Xb	L
HlOr35	NW_017100842.1	LOC108577319	284473	287343	+	Complete	Normal	411	5	6	6	9	6	7tm_6	7tm_6	KQ414889.1	scaffold697	Xrest	L
HlOr35like_1	NW_017100842.1	LOC108577311_M	278526	280557	+	Complete	Normal	411	5	6	6	9	6	7tm_6	7tm_6	KQ414889.1	scaffold697	Xrest	L
HlOr49like_1F	NW_017106372.1	New	543	647	-	Partial(Fragment)	Normal	35	1	1	1	1	1	-	-	LHQN01028838.1	scaffold6495	Xb	L
HlOr51	NW_017100842.1	LOC108577312_M	289679	292581	+	Complete	Normal	400	4	6	6	8	6	7tm_6	7tm_6	KQ414889.1	scaffold697	Xrest	L
HlOr52	NW_017100842.1	LOC108577312_M	299621	302172	+	Complete	Normal	404	6	6	6	6	6	7tm_6	7tm_6	KQ414889.1	scaffold697	Xrest	L
HlOr53like_1P	NW_017100842.1	LOC108577312_M	294658	297927	+	Complete	Pseudo	400	5	5	6	6	6	7tm_6	7tm_6	KQ414889.1	scaffold697	Xrest	L
HlOr60	NW_017100842.1	LOC108577338	303128	305940	+	Complete	Normal	407	6	8	6	8	8	7tm_6	7tm_6	KQ414889.1	scaffold697	Xrest	L
HlOr62	NW_017101026.1	LOC108577924	805797	807599	+	Complete	Normal	403	4	3	9	5	6	7tm_6	7tm_6	KQ414940.1	scaffold801	VII	M
HlOr68	NW_017100229.1	LOC108571252_M	3149024	3151146	+	Complete	Normal	372	5	6	7	7	7	7tm_6	7tm_6	KQ414646.1	scaffold57	XV	E
HlOr69	NW_017100229.1	LOC108571252_M	3154922	3156413	+	Complete	Normal	369	5	4	7	6	6	7tm_6	7tm_6	KQ414646.1	scaffold57	XV	E
HlOr69like_1F	NW_017100249.1	New	1948291	1948618	-	Partial(Fragment)	Normal	65	2	0	0	0	0	7tm_6	7tm_6	KQ414661.1	scaffold107	XV	E
HlOr70like_1	NW_017100229.1	LOC108571253_M	3159621	3161363	+	Complete	Normal	369	5	5	7	7	7	7tm_6	7tm_6	KQ414646.1	scaffold57	XV	E
HlOr70like_2	NW_017100229.1	LOC108571257_M	3179617	3181048	+	Complete	Normal	367	5	6	7	7	7	7tm_6	7tm_6	KQ414646.1	scaffold57	XV	E
HlOr70like_3P	NW_017100229.1	LOC108571252_M	3152422	3154098	+	Complete	Pseudo	371	4	5	6	6	6	7tm_6	7tm_6	KQ414646.1	scaffold57	XV	E
HlOr71	NW_017100229.1	LOC108571255_M	3163850	3165551	+	Complete	Normal	371	5	6	7	7	7	7tm_6	7tm_6	KQ414646.1	scaffold57	XV	E
HlOr71like_1	NW_017100249.1	LOC108572161	1945684	1947382	-	Complete	Normal	371	5	6	7	7	7	7tm_6	7tm_6	KQ414661.1	scaffold107	XV	E
HlOr71like_2	NW_017100229.1	LOC108571253_M	3157322	3158949	+	Complete	Normal	371	4	6	7	7	7	7tm_6	7tm_6	KQ414646.1	scaffold57	XV	E
HlOr71like_3PC	NW_017100229.1	LOC108571255_M	3174312	3175577	+	Partial(C)	Pseudo	234	4	3	5	4	4	7tm_6	7tm_6	KQ414646.1	scaffold57	XV	E
HlOr71like_4F	NW_017127561.1	LOC108570050	284	1345	-	Partial(Fragment)	Normal	219	3	2	2	2	5	7tm_6	7tm_6	LHQN01050085.1	C6058879	XV	E
HlOr73	NW_017100229.1	LOC108571256_M	3177232	3179029	+	Complete	Normal	365	5	3	7	6	6	7tm_6	7tm_6	KQ414646.1	scaffold57	XV	E
HlOr76	NW_017100645.1	LOC108576805_M	710	6483	+	Complete	Normal	397	6	5	7	8	7	7tm_6	7tm_6	KQ414828.1	scaffold544	XXI	J
HlOr76like_1	NW_017100942.1	New	1704895	1708526	+	Complete	Normal	413	5	5	7	9	8	7tm_6	7tm_6	KQ414915.1	scaffold718	XXI	J
HlOr84	NW_017100942.1	LOC108577629_M	1692074	1696278	+	Complete	Normal	398	7	7	6	8	7	7tm_6	7tm_6	KQ414915.1	scaffold718	XXI	J
HlOr84like_1F	NW_017113562.1	New	1	192	+	Partial(Fragment)	Normal	64	1	1	1	9	1	-	-	LHQN01036086.1	C5728787	XXI	J
HlOr86	NW_017100942.1	LOC108577628	1685752	1690094	+	Complete	Normal	398	6	6	6	6	6	7tm_6	7tm_6	KQ414915.1	scaffold718	XXI	J
HlOr87like_1	NW_017100942.1	LOC108577627_M	1680266	1683075	+	Complete	Normal	404	7	5	7	5	5	7tm_6	7tm_6	KQ414915.1	scaffold718	XXI	J
HlOr87like_2P	NW_017100942.1	LOC108577627_M	1676514	1682841	+	Complete	Pseudo	398	4	6	6	7	8	7tm_6	7tm_6	KQ414915.1	scaffold718	XXI	J
HlOr87like_3N_C	NW_017100942.1	LOC108577630_M	1700447	1703662	+	Partial(N + C)	Normal	291	4	4	6	6	5	7tm_6	7tm_6	KQ414915.1	scaffold718	XXI	J
HlOr87N	NW_017100942.1	LOC108577630_M	1697444	1699977	+	Partial(N)	Normal	318	6	5	6	6	6	7tm_6	7tm_6	KQ414915.1	scaffold718	XXI	J
HlOr90	NW_017100645.1	LOC108576806_M	7891	13173	+	Complete	Normal	409	6	6	8	7	8	7tm_6	7tm_6	KQ414828.1	scaffold544	XXI	J
HlOr94	NW_017100645.1	LOC108576807_M	21031	24208	+	Complete	Normal	409	6	6	6	7	6	7tm_6	7tm_6	KQ414828.1	scaffold544	XXI	J
HlOr94like_1P	NW_017100645.1	LOC108576809_M	31106	34328	+	Complete	Pseudo	410	6	6	6	8	7	7tm_6	7tm_6	KQ414828.1	scaffold544	XXI	J
HlOr94like_2	NW_017100645.1	LOC108576810_M	36172	39232	+	Complete	Normal	409	6	6	6	10	8	7tm_6	7tm_6	KQ414828.1	scaffold544	XXI	J

Table S2-HlOr information

HlOr94like_3N_C	NW_017100645.1	LOC108576808_M	26061	29233	+	Partial(N + C)	Normal	295	5	3	7	8	8	7tm_6	7tm_6	KQ414828.1	scaffold544	XXI	J
HlOr94like_4F	NW_017122225.1	New	2	154	-	Partial(Fragment)	Normal	51	1	0	0	1	0	7tm_6	-	LHQN01044749.1	C5796391	XXI	J
HlOr94like_5PF	NW_017118244.1	New	49	228	+	Partial(Fragment)	Pseudo	59	1	1	1	0	1	-	-	LHQN01040768.1	C5762901	XXI	J
HlOr94like_6F	NW_017126947.1	New	307	420	+	Partial(Fragment)	Normal	38	1	1	1	1	1	7tm_6	-	LHQN01049471.1	C5883721	XXI	J
HlOr95	NW_017100645.1	LOC108576806_M	15101	19924	+	Complete	Normal	412	6	7	6	8	7	7tm_6	7tm_6	KQ414828.1	scaffold544	XXI	J
HlOr95like_1	NW_017100942.1	LOC108577631_M	1710201	1713345	+	Complete	Normal	411	6	3	6	8	6	7tm_6	7tm_6	KQ414915.1	scaffold718	XXI	J
HlOr104C	NW_017100710.1	LOC108576982_M	1233124	1235257	-	Complete(C)	Normal	394	9	4	7	6	6	7tm_6	7tm_6	KQ414851.1	scaffold440	XI	9-exon
HlOr104like_1C	NW_017100710.1	LOC108576981_M	1228291	1230435	-	Complete(C)	Normal	404	9	6	6	6	6	7tm_6	7tm_6	KQ414851.1	scaffold440	XI	9-exon
HlOr104like_2PC	NW_017100710.1	LOC108576982_M	1242519	1246735	-	Complete(C)	Pseudo	399	8	3	6	7	5	7tm_6	7tm_6	KQ414851.1	scaffold440	XI	9-exon
HlOr104like_3PC	NW_017100710.1	LOC108576982_M	1238237	1240820	-	Complete(C)	Pseudo	403	9	4	7	4	4	7tm_6	7tm_6	KQ414851.1	scaffold440	XI	9-exon
HlOr105like_1	NW_017100710.1	LOC108576980_M	1222003	1226420	-	Complete	Normal	404	8	3	6	7	6	7tm_6	7tm_6	KQ414851.1	scaffold440	XI	9-exon
HlOr106like_2	NW_017100475.1	LOC108575990_M	44728	47792	-	Complete	Normal	360	9	3	6	4	5	-	-	KQ414772.1	scaffold360	XI	9-exon
HlOr108like_1F	NW_017102187.1	LOC108570066_M	20	1690	+	Partial(Fragment)	Normal	240	5	4	6	4	4	7tm_6	-	LHQN01023902.1	scaffold2160	XI	9-exon
HlOr111	NW_017100502.1	LOC108576159_M	28238	30582	-	Complete	Normal	380	9	6	8	6	7	7tm_6	7tm_6	LHQN01017803.1	scaffold417	XI	9-exon
HlOr111like_1	NW_017100475.1	LOC108575988_M	36909	38829	-	Complete	Normal	382	8	5	7	6	5	7tm_6	7tm_6	KQ414772.1	scaffold360	XI	9-exon
HlOr111like_2	NW_017100964.1	LOC108577674_M	368	3431	+	Complete	Normal	390	8	4	6	6	6	7tm_6	7tm_6	KQ414921.1	scaffold872	XI	9-exon
HlOr111like_3	NW_017100475.1	LOC108575985_M	12389	15387	-	Complete	Normal	386	8	2	6	6	6	7tm_6	7tm_6	KQ414772.1	scaffold360	XI	9-exon
HlOr111like_4N_C	NW_017100502.1	New	32566	35359	-	Partial(N + C)	Normal	320	9	5	6	5	5	-	-	LHQN01017803.1	scaffold417	XI	9-exon
HlOr111like_5	NW_017101643.1	New	19342	27644	+	Complete	Normal	389	8	5	8	8	9	7tm_6	7tm_6	KQ415081.1	scaffold1554	XI	9-exon
HlOr111like_6C	NW_017100150.1	LOC108574636_M	776343	776799	+	Partial(C)	Normal	106	3	1	2	1	1	7tm_6	7tm_6	KQ414588.1	scaffold11	XI	9-exon
HlOr112	NW_017100475.1	LOC108575987	19364	21695	-	Complete	Normal	392	9	6	6	5	6	7tm_6	7tm_6	KQ414772.1	scaffold360	XI	9-exon
HlOr112like_1	NW_017100502.1	LOC108576158_M	24793	26859	-	Complete	Normal	390	9	6	6	8	6	7tm_6	7tm_6	LHQN01017803.1	scaffold417	XI	9-exon
HlOr112like_2	NW_017106290.1	LOC108570061_M	4225	6291	-	Complete	Normal	391	9	6	6	8	6	7tm_6	7tm_6	LHQN01028732.1	scaffold6412	XI	9-exon
HlOr112like_3	NW_017103722.1	LOC108570075_M	4357	6438	+	Complete	Normal	390	9	4	6	8	8	7tm_6	7tm_6	LHQN01025741.1	scaffold3760	XI	9-exon
HlOr112like_4C	NW_017105508.1	LOC108570045_M	2556	3035	-	Partial(C)	Normal	94	3	0	2	1	1	7tm_6	7tm_6	LHQN01027734.1	scaffold5614	XI	9-exon
HlOr112like_5F1_F2	NW_017103722.1	LOC108578246_M	7778	9413	+	Partial(2 Fragments)	Normal	191	5	2	2	4	2	7tm_6	-	LHQN01025741.1	scaffold3760	XI	9-exon
HlOr112like_6N	NW_017100502.1	LOC108570070_M	20500	20832	-	Partial(N)	Normal	91	2	2	2	2	2	-	-	LHQN01017803.1	scaffold417	XI	9-exon
HlOr112like_7N	NW_017100502.1	LOC108576158_M	23124	23453	-	Partial(N)	Normal	90	2	2	2	2	2	-	-	LHQN01017803.1	scaffold417	XI	9-exon
HlOr112like_8N_C	NW_017106290.1	LOC108578299	1234	2994	-	Partial(N + C)	Normal	279	6	4	6	3	4	7tm_6	7tm_6	LHQN01028732.1	scaffold6412	XI	9-exon
HlOr113	NW_017100502.1	LOC108570070_M	16948	19458	-	Complete	Normal	391	9	6	6	5	6	7tm_6	7tm_6	LHQN01017803.1	scaffold417	XI	9-exon
HlOr113like_10PF	NW_017127237.1	LOC108570040_M	41	552	+	Partial(Fragment)	Pseudo	106	3	2	2	1	2	7tm_6	-	LHQN01049761.1	C5922691	XI	9-exon
HlOr113like_11C	NW_017100502.1	New	22166	22440	-	Partial(C)	Normal	65	2	0	1	0	0	7tm_6	7tm_6	LHQN01017803.1	scaffold417	XI	9-exon
HlOr113like_1C	NW_017100475.1	LOC108575986_M	16520	18446	-	Partial(C)	Normal	348	8	4	8	4	5	7tm_6	7tm_6	KQ414772.1	scaffold360	XI	9-exon
HlOr113like_2C	NW_017103722.1	LOC108578246_M	11107	13065	+	Partial(C)	Normal	341	8	4	7	5	6	7tm_6	7tm_6	LHQN01025741.1	scaffold3760	XI	9-exon
HlOr113like_3	NW_017100475.1	LOC108575989_M	41110	43517	-	Complete	Normal	391	9	5	8	7	7	7tm_6	7tm_6	KQ414772.1	scaffold360	XI	9-exon
HlOr113like_4	NW_017100475.1	New	31642	34431	-	Complete	Normal	390	9	6	6	6	6	7tm_6	7tm_6	KQ414772.1	scaffold360	XI	9-exon
HlOr113like_5	NW_017100475.1	New	27880	30438	-	Complete	Normal	389	9	6	8	7	6	7tm_6	7tm_6	KQ414772.1	scaffold360	XI	9-exon
HlOr113like_6F	NW_017105508.1	LOC108570059_M	367	1841	-	Partial(Fragment)	Normal	313	7	5	6	6	5	7tm_6	7tm_6	LHQN01027734.1	scaffold5614	XI	9-exon
HlOr113like_7C	NW_017103722.1	LOC108570075_M	1242	2976	+	Partial(C)	Normal	308	7	4	6	4	4	7tm_6	7tm_6	LHQN01025741.1	scaffold3760	XI	9-exon
HlOr113like_8C	NW_017100475.1	New	35194	36047	-	Partial(C)	Normal	134	4	2	3	3	3	7tm_6	7tm_6	KQ414772.1	scaffold360	XI	9-exon
HlOr113like_9C	NW_017106290.1	LOC108578299	7630	8132	-	Partial(nearC)	Normal	120	3	2	2	2	2	7tm_6	7tm_6	LHQN01028732.1	scaffold6412	XI	9-exon
HlOr114	NW_017100562.1	LOC108576617_M	44099	52373	+	Complete	Normal	391	6	6	7	7	6	7tm_6	7tm_6	KQ414804.1	scaffold473	VI	T
HlOr114like_1	NW_017100562.1	LOC108576615_M	30822	34680	+	Complete	Normal	384	6	5	6	7	6	7tm_6	7tm_6	KQ414804.1	scaffold473	VI	T
HlOr115	NW_017100562.1	LOC108576616	35907	40271	+	Complete	Normal	399	6	6	6	8	6	7tm_6	7tm_6	KQ414804.1	scaffold473	VI	T
HlOr116	NW_017100142.1	LOC108571793_M	618947	620817	+	Complete	Normal	412	6	7	6	7	7	7tm_6	7tm_6	KQ414583.1	scaffold34	XIV	C
HlOr117	NW_017100509.1	LOC108576195	4393579	4395886	+	Complete	Normal	401	5	4	7	6	6	7tm_6	7tm_6	KQ414784.1	scaffold212	XX	orphan
HlOr117like_1	NW_017100177.1	LOC108578812_M	1305874	1308263	-	Complete	Normal	401	5	6	6	6	6	7tm_6	7tm_6	KQ414608.1	scaffold62	XX	orphan
HlOr118	NW_017100347.1	LOC108574554	1385620	1387961	+	Complete	Normal	385	6	6	6	7	6	7tm_6	7tm_6	KQ414716.1	scaffold175	III	V
HlOr119	NW_017100191.1	LOC108580221_M	453892	459231	+	Complete	Normal	415	7	7	7	7	7	7tm_6	7tm_6	KQ414618.1	scaffold9	XIII	B
HlOr120	NW_017100187.1	LOC108579497_M	2575369	2577180	+	Complete	Normal	396	9	8	6	8	8	7tm_6	7tm_6	KQ414615.1	scaffold33	XIX	W
HlOr121P	NW_017100190.1	New	203472	204968	+	Complete	Pseudo	391	5	6	8	7	8	7tm_6	7tm_6	KQ414617.1	scaffold2	IV	U
HlOr130like_1	NW_017100616.1	New	443382	446076	+	Complete	Normal	390	7	6	8	8	7	7tm_6	7tm_6	KQ414815.1	scaffold469	XI	9-exon
HlOr130P	NW_017100616.1	New	439272	441964	+	Complete	Pseudo	389	11	4	8	7	7	7tm_6	7tm_6	KQ414815.1	scaffold469	XI	9-exon
HlOr140TRA	NW_017100397.1	LOC108575117_M	890049	892792	Trans	Complete	Normal	407	9	4	6	8	6	7tm_6	7tm_6	KQ414735.1	scaffold207	XI	9-exon
HlOr140TRAlike_1	NW_017100397.1	LOC108575238_M	885446	889153	Trans	Complete	Normal	405	9	8	6	9	8	7tm_6	7tm_6	KQ414735.1	scaffold207	XI	9-exon
HlOr141	NW_017101026.1	LOC108577857	116642	118062	+	Complete	Normal	391	5	5	6	7	7	7tm_6	7tm_6	KQ414940.1	scaffold801	XVI	Z
HlOr142	NW_017100191.1	LOC108580238_M	1054194	1058517	-	Complete	Normal	379	6	6	9	7	8	7tm_6	7tm_6	KQ414618.1	scaffold9	XVIII	H
HlOr143	NW_017100191.1	LOC108580238_M	1061287	1063143	-	Complete	Normal	373	6	4	9	7	7	7tm_6	7tm_6	KQ414618.1	scaffold9	XVII	G
HlOr146	NW_017100191.1	LOC108580235_M	1027904	1030017	-	Complete	Normal	376	6	7	9	8	8	7tm_6	7tm_6	KQ414618.1	scaffold9	XVIII	H
HlOr146like_1	NW_017100191.1	New	1035608	1039184	-	Complete	Normal	354	5	7	8	8	8	7tm_6	7tm_6	KQ414618.1	scaffold9	XVIII	H
HlOr146like_2	NW_017100822.1	LOC108570055_M	1408	5294	-	Complete	Normal	375	6	5	9	7	7	7tm_6	7tm_6	LHQN01020543.1	scaffold748	XVIII	H
HlOr152	NW_017100191.1	LOC108580236	1051848	1053857	-	Complete	Normal	374	6	7	9	9	9	7tm_6	7tm_6	KQ414618.1	scaffold9	XVIII	H
HlOr159	NW_017100616.1	New	466836	469477	+	Complete	Normal	395	9	7	9	10	7	7tm_6	7tm_6	KQ414815.1	scaffold469	XI	9-exon

Table S2-HlOr information

HlOr159like_1PN_PF	NW_017100616.1	New	488417	490350	+	Partial(N + F)	Pseudo	267	5	3	6	6	6	7tm_6	-	KQ414815.1	scaffold469	XI	9-exon
HlOr160	NW_017100500.1	LOC108576129	1342062	1345846	+	Complete	Normal	390	6	7	6	7	7	7tm_6	7tm_6	KQ414782.1	scaffold333	V	Q
HlOr161	NW_017100262.1	LOC108572822	801446	806042	-	Complete	Normal	386	7	5	6	8	7	7tm_6	7tm_6	KQ414667.1	scaffold102	II	I
HlOr162	NW_017100130.1	LOC108572110_M	628482	653065	-	Complete	Normal	395	9	4	6	6	6	7tm_6	7tm_6	KQ414573.1	scaffold16	XI	9-exon
HlOr162like_1	NW_017101643.1	New	29614	38748	+	Complete	Normal	395	9	5	6	5	8	7tm_6	7tm_6	KQ415081.1	scaffold1554	XI	9-exon
HlOr165like_1C	NW_017100347.1	LOC108574554	1398619	1402808	+	Partial(C)	Normal	289	5	4	4	5	4	7tm_6	7tm_6	KQ414716.1	scaffold175	III	V
HlOr165like_2F	NW_017127228.1	New	49	429	+	Partial(F)	Normal	126	1	2	2	2	2	-	-	LHQN01049752.1	C5920683	III	V
HlOr165like_3N	NW_017127184.1	New	58	315	+	Partial(N)	Normal	86	1	2	2	2	3	-	-	LHQN01049708.1	C5913785	III	V
HlOr166	NW_017100347.1	LOC108574586	1405075	1409531	+	Complete	Normal	375	6	5	6	7	6	7tm_6	7tm_6	KQ414716.1	scaffold175	III	V
HlOr166like_1C	NW_017127588.1	New	825	1197	+	Partial(C)	Normal	69	2	0	0	1		7tm_6	7tm_6	LHQN01050112.1	C6082701	III	V
HlOr167	NW_017100347.1	LOC108574587_M	1390524	1395518	+	Complete	Normal	382	6	5	6	7	6	7tm_6	7tm_6	KQ414716.1	scaffold175	III	V
HlOr169	NW_017100493.1	LOC108576089	57442	63131	+	Complete	Normal	400	5	5	9	8	9	7tm_6	7tm_6	KQ414777.1	scaffold334	I	A
HlOr172	NW_017100616.1	New	484184	487277	+	Complete	Normal	392	8	6	7	8	7	7tm_6	7tm_6	KQ414815.1	scaffold469	XI	9-exon
HlOr172like_1C	NW_017100616.1	New	456823	458807	+	Partial(C)	Normal	286	6	4	4	4	4	7tm_6	-	KQ414815.1	scaffold469	XI	9-exon
HlOr172like_2	NW_017100616.1	New	463813	466455	+	Complete	Normal	407	9	6	8	7	6	7tm_6	7tm_6	KQ414815.1	scaffold469	XI	9-exon
HlOr172like_3	NW_017100616.1	New	460289	462532	+	Complete	Normal	405	9	6	9	8	8	7tm_6	7tm_6	KQ414815.1	scaffold469	XI	9-exon
HlOr172like_4PN_PC	NW_017100616.1	New	470868	472961	+	Partial(N + C)	Pseudo	281	6	5	7	5	5	-	-	KQ414815.1	scaffold469	XI	9-exon
HlOr174like_1	NW_017100616.1	New	447015	449909	+	Complete	Normal	394	8	8	8	8	8	7tm_6	7tm_6	KQ414815.1	scaffold469	XI	9-exon
HlOr174like_2	NW_017100299.1	New	355374	358902	+	Complete	Normal	388	8	8	11	10	8	7tm_6	7tm_6	KQ414693.1	scaffold187	XI	9-exon
HlOr174like_3PF_PC	NW_017100616.1	New	451308	454568	+	Partial(F + C)	Pseudo	278	6	5	5	5	5	-	7tm_6	KQ414815.1	scaffold469	XI	9-exon
HlOr175	NW_017100616.1	New	480944	483490	+	Complete	Normal	420	8	7	8	9	8	7tm_6	7tm_6	KQ414815.1	scaffold469	XI	9-exon
HlOr175like_1	NW_017100616.1	New	474769	476964	+	Complete	Normal	429	9	6	8	7	9	7tm_6	7tm_6	KQ414815.1	scaffold469	XI	9-exon
HlOr175like_2PN_PC	NW_017100616.1	New	477938	479941	+	Partial(N + C)	Pseudo	310	8	4	8	6	6	7tm_6	7tm_6	KQ414815.1	scaffold469	XI	9-exon
HlOr176N_C	NW_017100618.1	LOC108576743_M	134741	142357	+	Partial(N + C)	Normal	327	8	6	8	6	6	7tm_6	7tm_6	KQ414817.1	scaffold478	XI	9-exon
HlOr177	NW_017101643.1	New	10183	13918	+	Complete	Normal	398	9	6	8	7	8	7tm_6	7tm_6	KQ415081.1	scaffold1554	XI	9-exon
HlOr177like_1	NW_017101643.1	LOC108578202_M	2999	6982	+	Complete	Normal	339	7	6	7	6	6	7tm_6	7tm_6	KQ415081.1	scaffold1554	XI	9-exon
HlOr177like_2N_C	NW_017100618.1	LOC108576742_M	128298	132135	+	Partial(N + C)	Normal	331	8	5	8	8	7	7tm_6	-	KQ414817.1	scaffold478	XI	9-exon
HlOr177like_3F	NW_017127610.1	New	245	936	+	Partial(Fragment)	Normal	199	2	2	4	2	2	7tm_6	-	LHQN01050134.1	C6090425	XI	9-exon
HlOr180C	NW_017100191.1	LOC108580234_M	1023763	1027143	-	Partial(C)	Normal	358	6	7	8	7	7	7tm_6	7tm_6	KQ414618.1	scaffold9	XXVII	X
HlOr181C	NW_017100168.1	LOC108578619_M	1066750	1071698	-	Partial(C)	Normal	365	6	7	6	7	7	7tm_6	7tm_6	KQ414601.1	scaffold45	XXV	R

Column C refers to known NCBI genes at the current OR positions. If there was none – it is written as 'New'. If the current NCBI annotation had to be modified to make it better, the gene name is followed by '_M'.
In column F, 'Trans' denotes trans-splicing.
Complete in column G refers to completeness of gene (>=370aa or with both termini). 'Fragment' or 'F' means our current annotation does not have both N as well as C terminus.
Subfamilies in column T refer to already known subfamilies of ORs from Zhou *et al.* 2012, Zhou *et al*. 2015 and Karpe *et al.* 2016.

Table S3-Upstream DNA motifs

ALPHABET= ACGT
Background letter frequencies
A 0.323 C 0.189 G 0.181 T 0.307

Motif 1 [GA]CGC[AT][AT]GCG[CT]
letter-probability matrix: alength= 4 w= 10 nsites= 184 E= 9.4e-189

0.271739	0	0.690217	0.038043
0.119565	0.858696	0.021739	0
0.070652	0	0.929348	0
0	0.98913	0.01087	0
0.76087	0	0.038043	0.201087
0.516304	0.13587	0.032609	0.315217
0	0	1	0
0	1	0	0
0	0	0.972826	0.027174
0.043478	0.630435	0	0.326087

Motif 2 ATCGATCG[AG][TC]
letter-probability matrix: alength= 4 w= 10 nsites= 184 E= 1.3e-102

0.956522	0	0.043478	0
0	0.01087	0	0.98913
0	0.940217	0	0.059783
0.054348	0	0.945652	0
0.934783	0.005435	0	0.059783
0.043478	0	0.01087	0.945652
0	0.880435	0	0.119565
0.097826	0	0.902174	0
0.668478	0.048913	0.25	0.032609
0.119565	0.380435	0	0.5

Motif 3 T[CT]AC[ACG]GC[TC]GG
letter-probability matrix: alength= 4 w= 10 nsites= 109 E= 8.7e-081

0	0	0	1
0.137615	0.458716	0.155963	0.247706
1	0	0	0
0	1	0	0
0.394495	0.247706	0.229358	0.12844
0	0	1	0
0	1	0	0
0.06422	0.293578	0.146789	0.495413
0	0	1	0
0	0	1	0

Table S3-Upstream DNA motifs

Motif 4 CAGT[CT]G[TC][TG]T[TC]

letter-probability matrix: alength= 4 w= 10 nsites= 99 E= 7.3e-035

0	1	0	0
0.989899	0	0	0.010101
0	0	1	0
0	0	0	1
0	0.69697	0	0.30303
0	0	1	0
0	0.232323	0	0.767677
0.040404	0	0.232323	0.727273
0	0.121212	0.090909	0.787879
0.020202	0.373737	0.111111	0.494949

Motif 5 [GC][ACT][GC][TAC]CGCG

letter-probability matrix: alength= 4 w= 8 nsites= 177 E= 1.0e-027

0	0.333333	0.666667	0
0.412429	0.344633	0	0.242938
0	0.378531	0.60452	0.016949
0.338983	0.254237	0	0.40678
0	1	0	0
0	0	1	0
0	1	0	0
0	0	1	0

Motif 6 TGACGATG[GT]C

letter-probability matrix: alength= 4 w= 10 nsites= 41 E= 9.6e-025

0	0.04878	0.121951	0.829268
0	0	1	0
1	0	0	0
0	1	0	0
0.121951	0	0.878049	0
0.97561	0	0.02439	0
0	0	0	1
0	0.02439	0.97561	0
0.073171	0	0.585366	0.341463
0	1	0	0

Motif 7 CG[AT]TGC[GA]GCG

letter-probability matrix: alength= 4 w= 10 nsites= 41 E= 1.9e-014

0.121951	0.829268	0.04878	0
0	0	1	0
0.487805	0.02439	0	0.487805
0	0.121951	0.170732	0.707317
0	0	1	0
0	1	0	0
0.292683	0.170732	0.439024	0.097561
0	0	1	0
0	1	0	0
0.04878	0.02439	0.926829	0

Table S3-Upstream DNA motifs

Motif 8 TTTTGCAACG

letter-probability matrix: alength= 4 w= 10 nsites= 56 E= 1.7e-016

0.035714	0	0.053571	0.910714
0.089286	0	0	0.910714
0	0.017857	0	0.982143
0	0	0.107143	0.892857
0	0	1	0
0	0.910714	0.089286	0
0.946429	0	0	0.053571
0.910714	0.053571	0.035714	0
0	0.821429	0	0.178571
0	0	1	0

Motif 9 TGGTAC[AG]A[CT]A

letter-probability matrix: alength= 4 w= 10 nsites= 56 E= 1.3e-021

0	0	0	1
0	0	1	0
0	0	1	0
0	0	0	1
1	0	0	0
0	1	0	0
0.553571	0	0.446429	0
1	0	0	0
0	0.625	0	0.375
0.660714	0.160714	0	0.178571

Motif 10 [TC]GCT[GT]CG[TC]GC

letter-probability matrix: alength= 4 w= 10 nsites= 43 E= 3.4e-012

0.023256	0.27907	0.023256	0.674419
0.069767	0	0.930233	0
0	0.883721	0	0.116279
0	0	0	1
0.093023	0	0.697674	0.209302
0	0.953488	0	0.046512
0	0	1	0
0	0.395349	0	0.604651
0.186047	0	0.813953	0
0	1	0	0

Table S4: Distribution of putative upstream regulatory elements of bee ORs across OR subfamilies

Clades	No. of ORs	Motif 1	Motif 2	Motif 3	Motif 4	Motif 5	Motif 6	Motif 7	Motif 8	Motif 9	Motif 10
Orco_Orco	4	0.00	0.00	0.00	0.00	0.00	0.00	0.00	0.00	0.00	0.00
I_A	9	0.00	0.00	0.00	11.11	0.00	0.00	0.00	0.00	0.00	11.11
II_I	4	0.00	**50.00**	0.00	0.00	0.00	25.00	0.00	0.00	0.00	0.00
III_V	25	0.00	0.00	0.00	0.00	8.00	4.00	4.00	0.00	0.00	4.00
IV_U	8	0.00	0.00	12.50	0.00	0.00	12.50	25.00	0.00	0.00	0.00
V_Q	4	0.00	0.00	0.00	0.00	0.00	0.00	0.00	0.00	0.00	0.00
VI_T	10	10.00	20.00	0.00	0.00	0.00	0.00	0.00	0.00	0.00	0.00
VII_M	4	0.00	0.00	25.00	0.00	**50.00**	0.00	**50.00**	0.00	0.00	0.00
VIII_P	11	0.00	**90.91**	0.00	0.00	9.09	0.00	0.00	9.09	0.00	27.27
IX_K	7	**100.00**	28.57	0.00	**57.14**	0.00	0.00	0.00	28.57	0.00	0.00
X_L	147	**79.59**	**37.41**	2.72	**41.50**	5.44	1.36	1.36	6.80	4.08	6.12
XI_9-exon	157	7.01	<u>26.75</u>	1.91	12.74	17.83	3.82	1.27	3.18	1.27	1.91
XII_F	3	0.00	**100.00**	0.00	0.00	0.00	0.00	0.00	**33.33**	0.00	0.00
XIII_B	4	0.00	0.00	25.00	0.00	0.00	25.00	0.00	0.00	0.00	0.00
XIV_C	4	0.00	0.00	0.00	25.00	0.00	0.00	0.00	0.00	0.00	0.00
XV_E	31	0.00	0.00	6.45	3.23	3.23	6.45	9.68	0.00	6.45	3.23
XVI_Z	3	0.00	0.00	0.00	0.00	**66.67**	0.00	**33.33**	0.00	0.00	0.00
XVII_G	6	0.00	0.00	0.00	0.00	0.00	0.00	0.00	0.00	0.00	**83.33**
XVIII_H	37	2.70	16.22	5.41	24.32	2.70	0.00	5.41	5.41	5.41	5.41
XIX_W	4	0.00	0.00	0.00	0.00	0.00	0.00	0.00	25.00	0.00	0.00
XX_orphan	5	0.00	0.00	20.00	0.00	0.00	0.00	20.00	0.00	0.00	0.00
XXI_J	71	7.04	15.49	4.23	11.27	18.31	14.08	5.63	7.04	2.82	4.23
XXV_R	2	**50.00**	0.00	0.00	0.00	0.00	0.00	0.00	0.00	0.00	0.00
XXVII_X	2	0.00	0.00	0.00	**50.00**	0.00	50.00	0.00	0.00	0.00	0.00
Xa_L	27	**100.00**	3.70	0.00	0.00	3.70	0.00	0.00	7.41	3.70	0.00
Xb_L	54	**51.85**	**83.33**	1.85	**68.52**	5.56	0.00	1.85	12.96	9.26	5.56
Xrest_L	66	**93.94**	13.64	4.55	**36.36**	6.06	3.03	1.52	1.52	0.00	9.09

Each cell represents the percentage of bee ORs from respective clades/subfamilies with the corresponding DNA motif upstream to them.

Cells with more than 35% coverage are highlighted in bold.

Considerable number of ORs from 9-exon clade (underlined) also show presence of motif 2.

Table S5 - Distribution of putative upstream regulatory elements of ant ORs across OR subfamilies

Clades	No. of Ant ORs	Motif 1	Motif 2	Motif 3	Motif 4	Motif 5	Motif 6	Motif 7	Motif 8	Motif 9	Motif 10
Orco_Orco	1	0.00	0.00	**100.00**	0.00	0.00	0.00	0.00	0.00	0.00	0.00
I_A	17	11.76	0.00	5.88	5.88	0.00	0.00	5.88	17.65	5.88	5.88
II_I	1	0.00	**100.00**	0.00	0.00	0.00	0.00	0.00	0.00	0.00	0.00
III_V	54	7.41	12.96	**44.44**	12.96	20.37	7.41	**57.41**	14.81	**64.81**	20.37
IV_U	36	0.00	0.00	**86.11**	0.00	0.00	**83.33**	2.78	**75.00**	30.56	**80.56**
V_Q	3	33.33	0.00	33.33	0.00	**66.67**	33.33	33.33	0.00	**66.67**	0.00
VI_T	9	0.00	11.11	**77.78**	11.11	**66.67**	11.11	0.00	22.22	**77.78**	0.00
VII_M	4	25.00	25.00	**50.00**	0.00	**50.00**	25.00	**50.00**	0.00	0.00	25.00
VIII_P	10	30.00	0.00	0.00	0.00	**40.00**	0.00	0.00	0.00	0.00	10.00
IX_K	2	0.00	**50.00**	**50.00**	0.00	**50.00**	0.00	0.00	0.00	0.00	0.00
X_L	56	8.77	7.02	**71.93**	3.51	5.26	3.51	1.75	14.04	10.53	12.28
XI_9-exon	130	10.00	22.31	7.69	6.15	20.77	3.08	3.85	8.46	6.15	8.46
XII_F	1	0.00	0.00	0.00	0.00	0.00	0.00	0.00	0.00	0.00	**100.00**
XIII_B	1	0.00	0.00	**100.00**	0.00	0.00	0.00	0.00	0.00	0.00	0.00
XIV_C	1	0.00	**100.00**	0.00	0.00	0.00	**100.00**	0.00	0.00	0.00	0.00
XV_E	26	15.38	7.69	0.00	3.85	**42.31**	11.54	15.38	15.38	15.38	0.00
XVIII_H	9	11.11	0.00	0.00	11.11	0.00	0.00	11.11	0.00	11.11	0.00
XIX_W	1	0.00	0.00	**100.00**	0.00	0.00	0.00	0.00	0.00	0.00	0.00
XXI_J	2	0.00	0.00	**50.00**	0.00	0.00	0.00	**50.00**	0.00	0.00	0.00
XXII_D	4	25.00	0.00	0.00	0.00	25.00	25.00	0.00	0.00	0.00	0.00
XXIII_N	3	33.33	33.33	0.00	0.00	**66.67**	33.33	0.00	0.00	0.00	0.00
XXV_R	2	0.00	**50.00**	**50.00**	0.00	0.00	0.00	0.00	0.00	**50.00**	0.00
XXVI_S	1	0.00	0.00	0.00	0.00	0.00	0.00	0.00	0.00	0.00	0.00
XXVII_X	1	**100.00**	**100.00**	0.00	0.00	0.00	0.00	0.00	0.00	0.00	0.00
XXVIII_Y	1	0.00	0.00	0.00	0.00	**100.00**	0.00	0.00	0.00	0.00	0.00
XXIX_ZA	1	0.00	0.00	**100.00**	0.00	0.00	0.00	0.00	0.00	0.00	0.00
Xa_L	21	14.29	0.00	**47.62**	4.76	0.00	0.00	0.00	4.76	0.00	4.76
Xb_L	17	5.88	5.88	**94.12**	0.00	0.00	0.00	5.88	23.53	17.65	0.00
Xrest_L	18	5.56	16.67	**83.33**	5.56	16.67	11.11	0.00	16.67	16.67	33.33

Each cell represents the percentage of ant ORs from respective clades/subfamilies with the corresponding DNA motif upstream to them.
Cells with more than 35% coverage are highlighted in bold.
Considerable number of ORs from 9-exon clade also show presence of motif 2.

Table S6 - Details about few OR subfamilies from sequenced genomes along with their degree of eusociality

OR	Subfamily L (X)				Subfamily 9-exon (XI) (putative CHC ORs)	Subfamily J (XXI) (Bee expanded ORs)	Subfamily H (XVIII) (Putative terpenoid ORs)	Genome N50 in bp (NCBI)	Genome size after assembly in Mb(NCBI)	Eusocial status
	Xa (putative QMP ORs)	Xb	Xrest	Xtotal (Xa+ Xb+ Xrest)						
**AmOr**	14	22	22	58	43	23	14	~997kb (Chromosome level assembly)	246.927	Obligate complex eusocial bee
**HsOr**	21	17	18	56	130	2	9	38321	294.466	Obligate complex eusocial ant
**AfOr**	12	19	22	53	44	21	15	25704	230.485	Obligate complex eusocial bee
**MquaOR (Brand et al. 2017)**	8	16	27	51	26	16	22	12520	256.303	Obligate complex eusocial bee
**BtOr (Brand et al. 2017)**	6	17	21	44	38	17	12	76043 (Chromosome level assembly)	248.654	Obligate simple eusocial bee
**EmexOR (Brand et al. 2017)**	5	12	15	32	22	28	9	38936	596.278	Facultative simple eusocial bee
**EdilOR (Brand et al. 2017)**	5	11	16	32	21	28	16	12398	588.2	Solitary/ weakly eusocial bee
**HlOr**	1	8	12	21	49	15	5	22370	296.955	Ancestrally solitary bee
**DnOr**	0	5	10	15	21	12	3	191865	279.506	Ancestrally solitary bee
**NvOr**	0	0	8	8	90	3	1	18840 (Chromosome level assembly)	295.781	Solitary wasp

Supplementary Figure S1-
Validation of ORs with TMH prediction and sequence domains

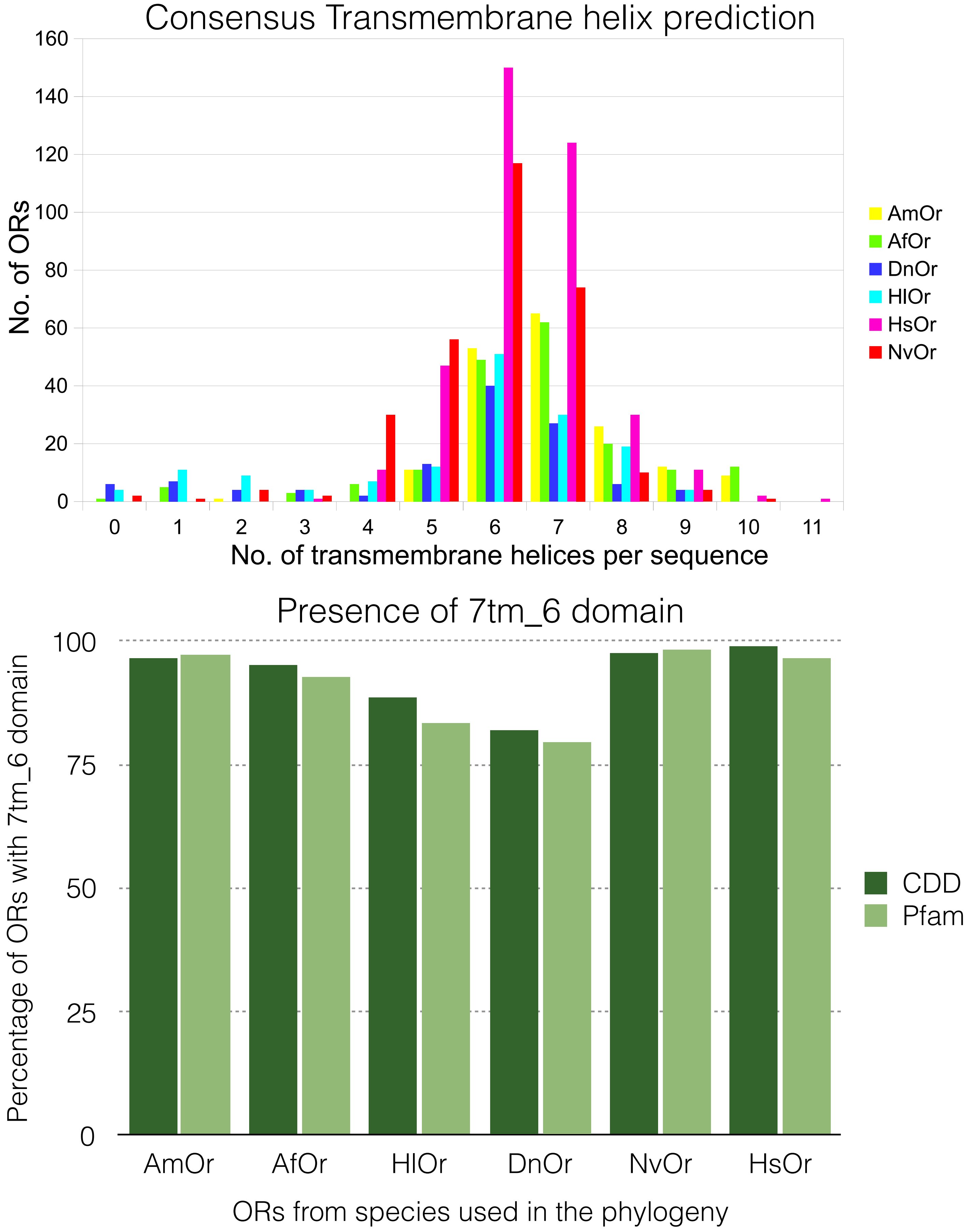

Supplementary Figure S2 - Synteny analysis of ORs

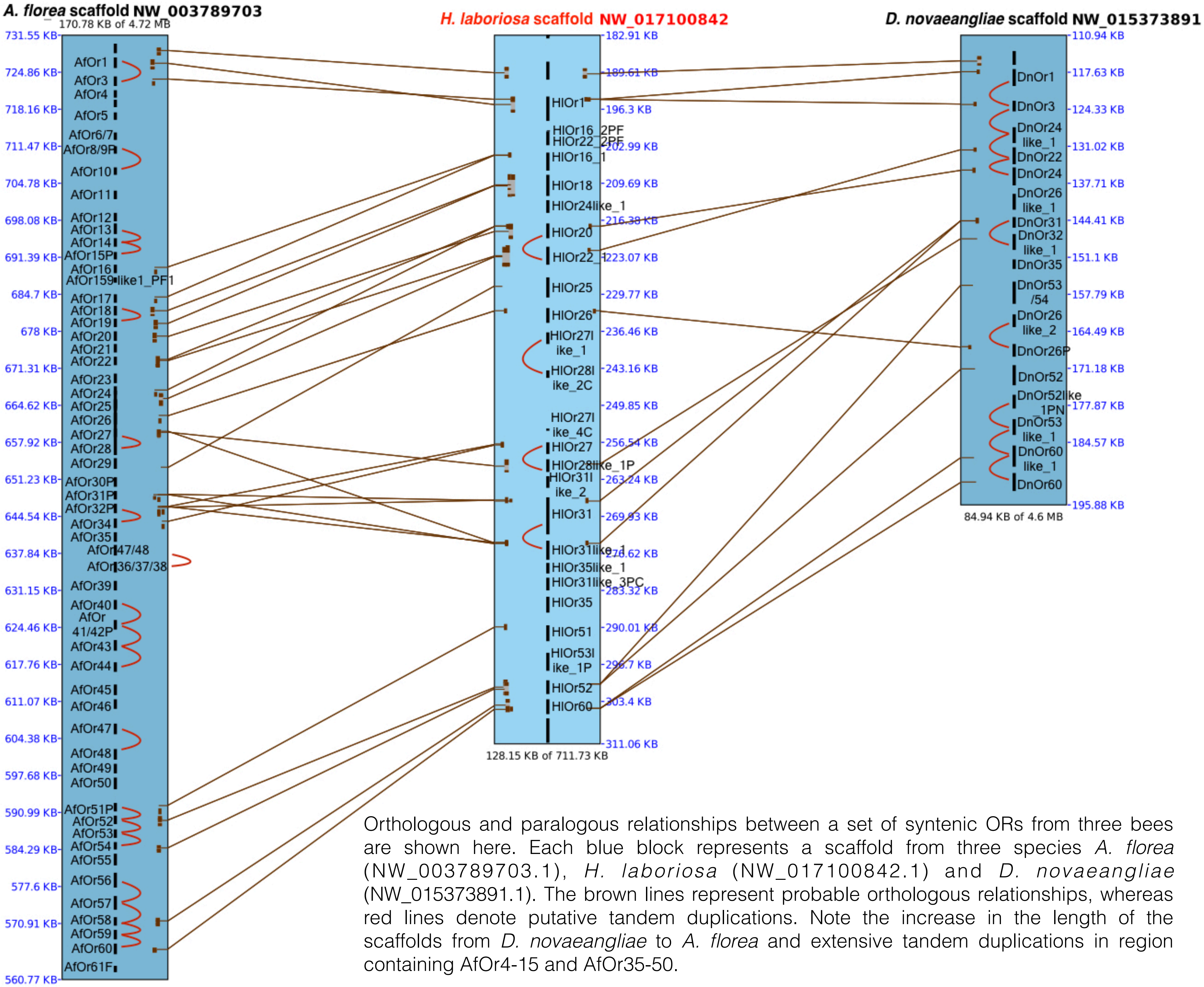

Orthologous and paralogous relationships between a set of syntenic ORs from three bees are shown here. Each blue block represents a scaffold from three species *A. florea* (NW_003789703.1), *H. laboriosa* (NW_017100842.1) and *D. novaeangliae* (NW_015373891.1). The brown lines represent probable orthologous relationships, whereas red lines denote putative tandem duplications. Note the increase in the length of the scaffolds from *D. novaeangliae* to *A. florea* and extensive tandem duplications in region containing AfOr4-15 and AfOr35-50.

Supplementary Figure S3 - Phylogeny

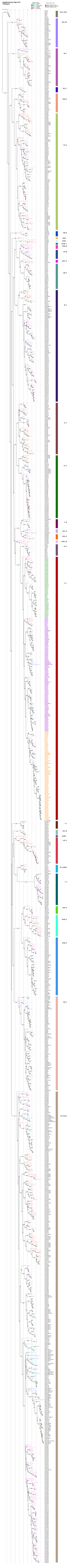

Supplementary Figure S4- Upstream conserved DNA motifs of OR genes plotted on the phylogeny of OR proteins

10 upstream conserved motifs are shown in form of concentric circles made up of bars. From centre to periphery - Motif1 to Motif10, with decreasing intensity of grey. Height of a bar represents number of occurrence of the motif per sequence.

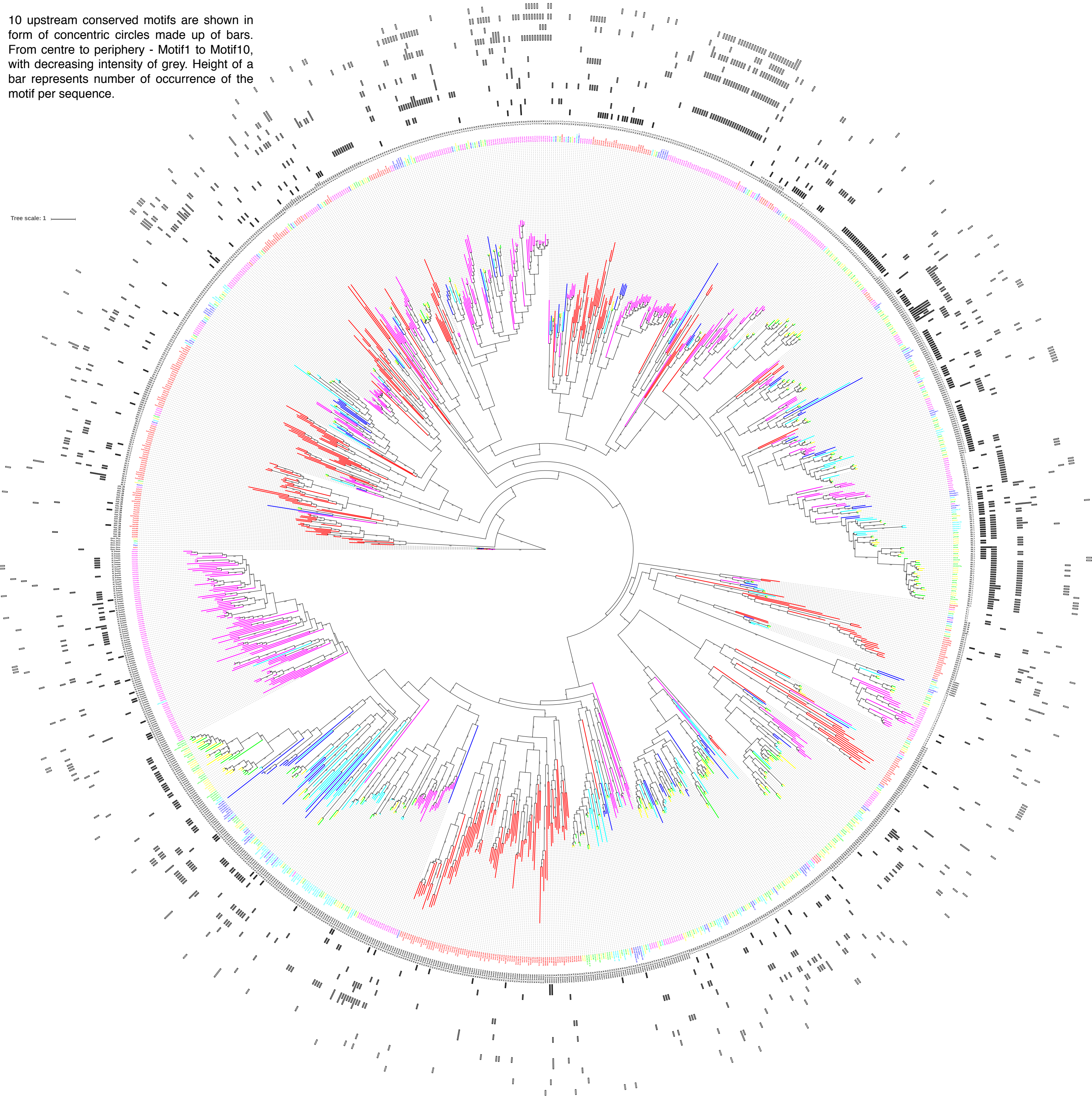

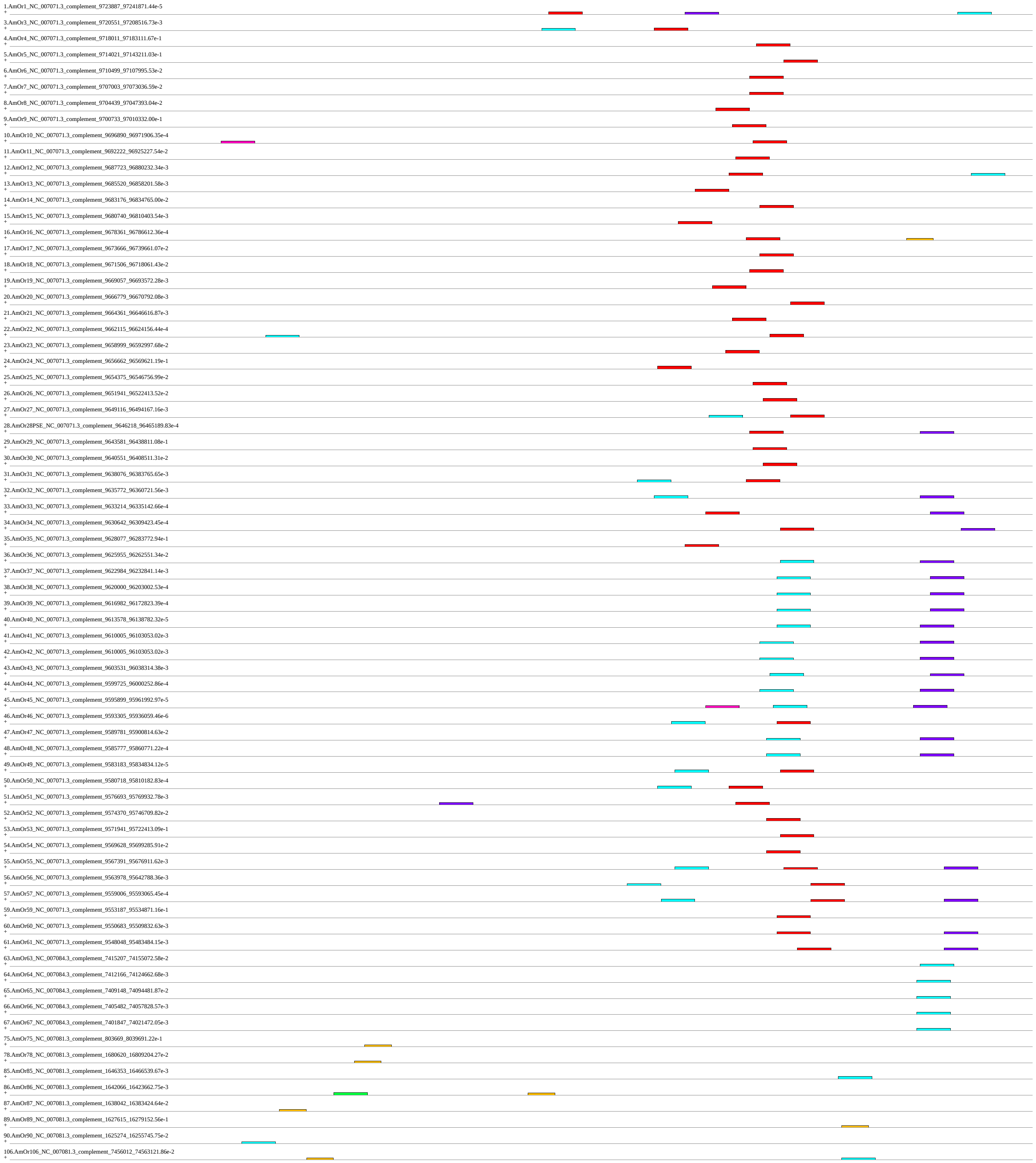

Supplementary Figure S5 - Ten conserved DNA motifs of upstream regions of OR genes mapped onto their sequences.

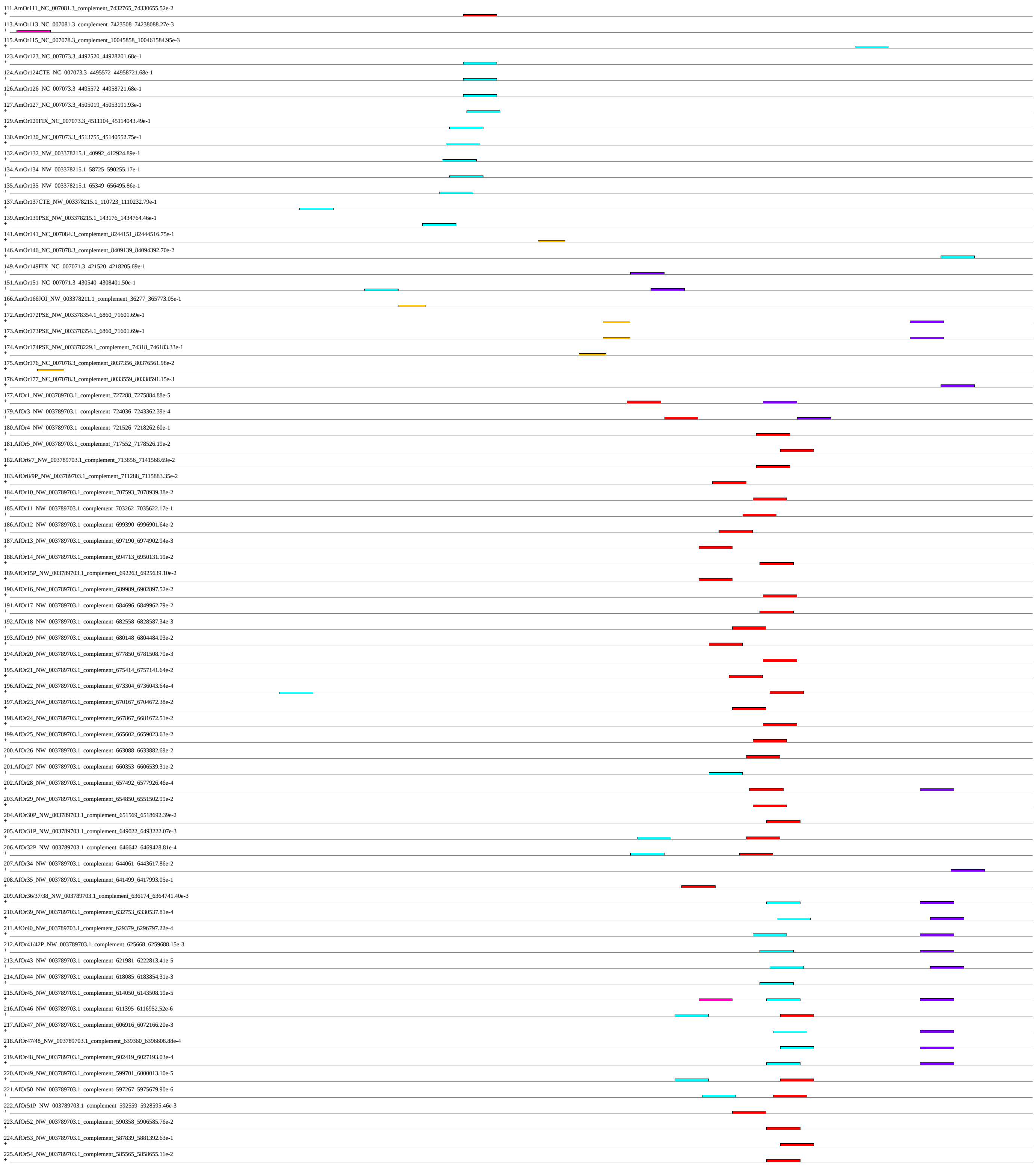
111.AmOr111_NC_007081.3_complement_7432765_74330655.52e-2
113.AmOr113_NC_007081.3_complement_7423508_74238088.27e-3
115.AmOr115_NC_007078.3_complement_10045858_100461584.95e-3
123.AmOr123_NC_007073.3_4492520_44928201.68e-1
124.AmOr124CTE_NC_007073.3_4495572_44958721.68e-1
126.AmOr126_NC_007073.3_4495572_44958721.68e-1
127.AmOr127_NC_007073.3_4505019_45053191.93e-1
129.AmOr129FIX_NC_007073.3_4511104_45114043.49e-1
130.AmOr130_NC_007073.3_4513755_45140552.75e-1
132.AmOr132_NW_003378215.1_40992_412924.89e-1
134.AmOr134_NW_003378215.1_58725_590255.17e-1
135.AmOr135_NW_003378215.1_65349_656495.86e-1
137.AmOr137CTE_NW_003378215.1_110723_1110232.79e-1
139.AmOr139PSE_NW_003378215.1_143176_1434764.46e-1
141.AmOr141_NC_007084.3_complement_8244151_82444516.75e-1
146.AmOr146_NC_007078.3_complement_8409139_84094392.70e-2
149.AmOr149FIX_NC_007071.3_421520_4218205.69e-1
151.AmOr151_NC_007071.3_430540_4308401.50e-1
166.AmOr166JOI_NW_003378211.1_complement_36277_365773.05e-1
172.AmOr172PSE_NW_003378354.1_6860_71601.69e-1
173.AmOr173PSE_NW_003378354.1_6860_71601.69e-1
174.AmOr174PSE_NW_003378229.1_complement_74318_746183.33e-1
175.AmOr176_NC_007078.3_complement_8037356_80376561.98e-2
176.AmOr177_NC_007078.3_complement_8033559_80338591.15e-3
177.AfOr1_NW_003789703.1_complement_727288_7275884.88e-5
179.AfOr3_NW_003789703.1_complement_724036_7243362.39e-4
180.AfOr4_NW_003789703.1_complement_721526_7218262.60e-1
181.AfOr5_NW_003789703.1_complement_717552_7178526.19e-2
182.AfOr6/7_NW_003789703.1_complement_713856_7141568.69e-2
183.AfOr8/9P_NW_003789703.1_complement_711288_7115883.35e-2
184.AfOr10_NW_003789703.1_complement_707593_7078939.38e-2
185.AfOr11_NW_003789703.1_complement_703262_7035622.17e-1
186.AfOr12_NW_003789703.1_complement_699390_6996901.64e-2
187.AfOr13_NW_003789703.1_complement_697190_6974902.94e-3
188.AfOr14_NW_003789703.1_complement_694713_6950131.19e-2
189.AfOr15P_NW_003789703.1_complement_692263_6925639.10e-2
190.AfOr16_NW_003789703.1_complement_689989_6902897.52e-2
191.AfOr17_NW_003789703.1_complement_684696_6849962.79e-2
192.AfOr18_NW_003789703.1_complement_682558_6828587.34e-3
193.AfOr19_NW_003789703.1_complement_680148_6804484.03e-2
194.AfOr20_NW_003789703.1_complement_677850_6781508.79e-3
195.AfOr21_NW_003789703.1_complement_675414_6757141.64e-2
196.AfOr22_NW_003789703.1_complement_673304_6736043.64e-4
197.AfOr23_NW_003789703.1_complement_670167_6704672.38e-2
198.AfOr24_NW_003789703.1_complement_667867_6681672.51e-2
199.AfOr25_NW_003789703.1_complement_665602_6659023.63e-2
200.AfOr26_NW_003789703.1_complement_663088_6633882.69e-2
201.AfOr27_NW_003789703.1_complement_660353_6606539.31e-2
202.AfOr28_NW_003789703.1_complement_657492_6577926.46e-4
203.AfOr29_NW_003789703.1_complement_654850_6551502.99e-2
204.AfOr30P_NW_003789703.1_complement_651569_6518692.39e-2
205.AfOr31P_NW_003789703.1_complement_649022_6493222.07e-3
206.AfOr32P_NW_003789703.1_complement_646642_6469428.81e-4
207.AfOr34_NW_003789703.1_complement_644061_6443617.86e-2
208.AfOr35_NW_003789703.1_complement_641499_6417993.05e-1
209.AfOr36/37/38_NW_003789703.1_complement_636174_6364741.40e-3
210.AfOr39_NW_003789703.1_complement_632753_6330537.81e-4
211.AfOr40_NW_003789703.1_complement_629379_6296797.22e-4
212.AfOr41/42P_NW_003789703.1_complement_625668_6259688.15e-3
213.AfOr43_NW_003789703.1_complement_621981_6222813.41e-5
214.AfOr44_NW_003789703.1_complement_618085_6183854.31e-3
215.AfOr45_NW_003789703.1_complement_614050_6143508.19e-5
216.AfOr46_NW_003789703.1_complement_611395_6116952.52e-6
217.AfOr47_NW_003789703.1_complement_606916_6072166.20e-3
218.AfOr47/48_NW_003789703.1_complement_639360_6396608.88e-4
219.AfOr48_NW_003789703.1_complement_602419_6027193.03e-4
220.AfOr49_NW_003789703.1_complement_599701_6000013.10e-5
221.AfOr50_NW_003789703.1_complement_597267_5975679.90e-6
222.AfOr51P_NW_003789703.1_complement_592559_5928595.46e-3
223.AfOr52_NW_003789703.1_complement_590358_5906585.76e-2
224.AfOr53_NW_003789703.1_complement_587839_5881392.63e-1
225.AfOr54_NW_003789703.1_complement_585565_5858655.11e-2

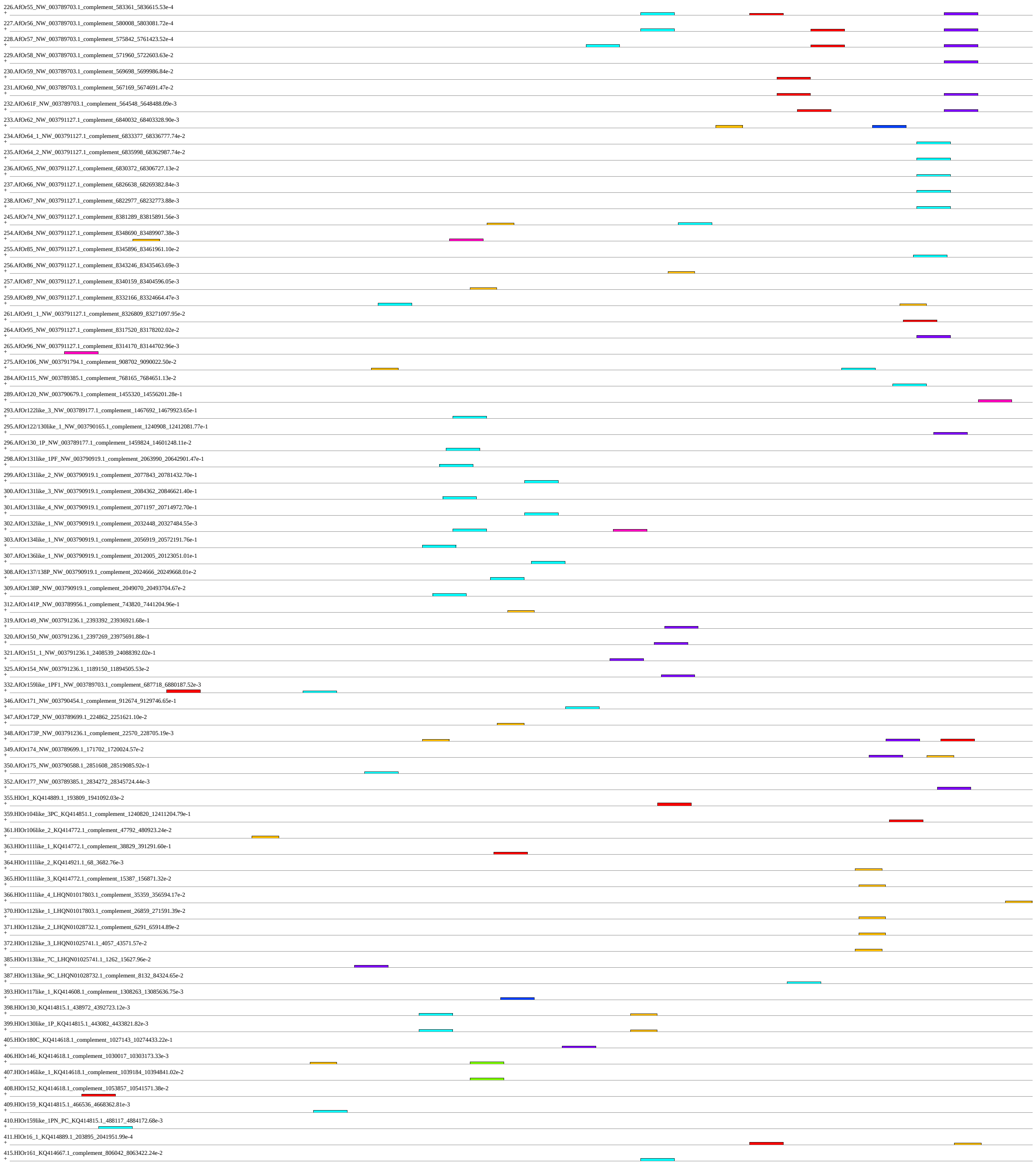
226.AfOr55_NW_003789703.1_complement_583361_5836615.53e-4
227.AfOr56_NW_003789703.1_complement_580008_5803081.72e-4
228.AfOr57_NW_003789703.1_complement_575842_5761423.52e-4
229.AfOr58_NW_003789703.1_complement_571960_5722603.63e-2
230.AfOr59_NW_003789703.1_complement_569698_5699986.84e-2
231.AfOr60_NW_003789703.1_complement_567169_5674691.47e-2
232.AfOr61F_NW_003789703.1_complement_564548_5648488.09e-3
233.AfOr62_NW_003791127.1_complement_6840032_68403328.90e-3
234.AfOr64_1_NW_003791127.1_complement_6833377_68336777.74e-2
235.AfOr64_2_NW_003791127.1_complement_6835998_68362987.74e-2
236.AfOr65_NW_003791127.1_complement_6830372_68306727.13e-2
237.AfOr66_NW_003791127.1_complement_6826638_68269382.84e-3
238.AfOr67_NW_003791127.1_complement_6822977_68232773.88e-3
245.AfOr74_NW_003791127.1_complement_8381289_83815891.56e-3
254.AfOr84_NW_003791127.1_complement_8348690_83489907.38e-3
255.AfOr85_NW_003791127.1_complement_8345896_83461961.10e-2
256.AfOr86_NW_003791127.1_complement_8343246_83435463.69e-3
257.AfOr87_NW_003791127.1_complement_8340159_83404596.05e-3
259.AfOr89_NW_003791127.1_complement_8332166_83324664.47e-3
261.AfOr91_1_NW_003791127.1_complement_8326809_83271097.95e-2
264.AfOr95_NW_003791127.1_complement_8317520_83178202.02e-2
265.AfOr96_NW_003791127.1_complement_8314170_83144702.96e-3
275.AfOr106_NW_003791794.1_complement_908702_9090022.50e-2
284.AfOr115_NW_003789385.1_complement_768165_7684651.13e-2
289.AfOr120_NW_003790679.1_complement_1455320_14556201.28e-1
293.AfOr122like_3_NW_003789177.1_complement_1467692_14679923.65e-1
295.AfOr122/130like_1_NW_003790165.1_complement_1240908_12412081.77e-1
296.AfOr130_1P_NW_003789177.1_complement_1459824_14601248.11e-2
298.AfOr131like_1PF_NW_003790919.1_complement_2063990_20642901.47e-1
299.AfOr131like_2_NW_003790919.1_complement_2077843_20781432.70e-1
300.AfOr131like_3_NW_003790919.1_complement_2084362_20846621.40e-1
301.AfOr131like_4_NW_003790919.1_complement_2071197_20714972.70e-1
302.AfOr132like_1_NW_003790919.1_complement_2032448_20327484.55e-3
303.AfOr134like_1_NW_003790919.1_complement_2056919_20572191.76e-1
307.AfOr136like_1_NW_003790919.1_complement_2012005_20123051.01e-1
308.AfOr137/138P_NW_003790919.1_complement_2024666_20249668.01e-2
309.AfOr138P_NW_003790919.1_complement_2049070_20493704.67e-2
312.AfOr141P_NW_003789956.1_complement_743820_7441204.96e-1
319.AfOr149_NW_003791236.1_2393392_23936921.68e-1
320.AfOr150_NW_003791236.1_2397269_23975691.88e-1
321.AfOr151_1_NW_003791236.1_2408539_24088392.02e-1
325.AfOr154_NW_003791236.1_1189150_11894505.53e-2
332.AfOr159like_1PF1_NW_003789703.1_complement_687718_6880187.52e-3
346.AfOr171_NW_003790454.1_complement_912674_9129746.65e-1
347.AfOr172P_NW_003789699.1_224862_2251621.10e-2
348.AfOr173P_NW_003791236.1_complement_22570_228705.19e-3
349.AfOr174_NW_003789699.1_171702_1720024.57e-2
350.AfOr175_NW_003790588.1_2851608_28519085.92e-1
352.AfOr177_NW_003789385.1_2834272_28345724.44e-3
355.HlOr1_KQ414889.1_193809_1941092.03e-2
359.HlOr104like_3PC_KQ414851.1_complement_1240820_12411204.79e-1
361.HlOr106like_2_KQ414772.1_complement_47792_480923.24e-2
363.HlOr111like_1_KQ414772.1_complement_38829_391291.60e-1
364.HlOr111like_2_KQ414921.1_68_3682.76e-3
365.HlOr111like_3_KQ414772.1_complement_15387_156871.32e-2
366.HlOr111like_4_LHQN01017803.1_complement_35359_356594.17e-2
370.HlOr112like_1_LHQN01017803.1_complement_26859_271591.39e-2
371.HlOr112like_2_LHQN01028732.1_complement_6291_65914.89e-2
372.HlOr112like_3_LHQN01025741.1_4057_43571.57e-2
385.HlOr113like_7C_LHQN01025741.1_1262_15627.96e-2
387.HlOr113like_9C_LHQN01028732.1_complement_8132_84324.65e-2
393.HlOr117like_1_KQ414608.1_complement_1308263_13085636.75e-3
398.HlOr130_KQ414815.1_438972_4392723.12e-3
399.HlOr130like_1P_KQ414815.1_443082_4433821.82e-3
405.HlOr180C_KQ414618.1_complement_1027143_10274433.22e-1
406.HlOr146_KQ414618.1_complement_1030017_10303173.33e-3
407.HlOr146like_1_KQ414618.1_complement_1039184_10394841.02e-2
408.HlOr152_KQ414618.1_complement_1053857_10541571.38e-2
409.HlOr159_KQ414815.1_466536_4668362.81e-3
410.HlOr159like_1PN_PC_KQ414815.1_488117_4884172.68e-3
411.HlOr16_1_KQ414889.1_203895_2041951.99e-4
415.HlOr161_KQ414667.1_complement_806042_8063422.24e-2

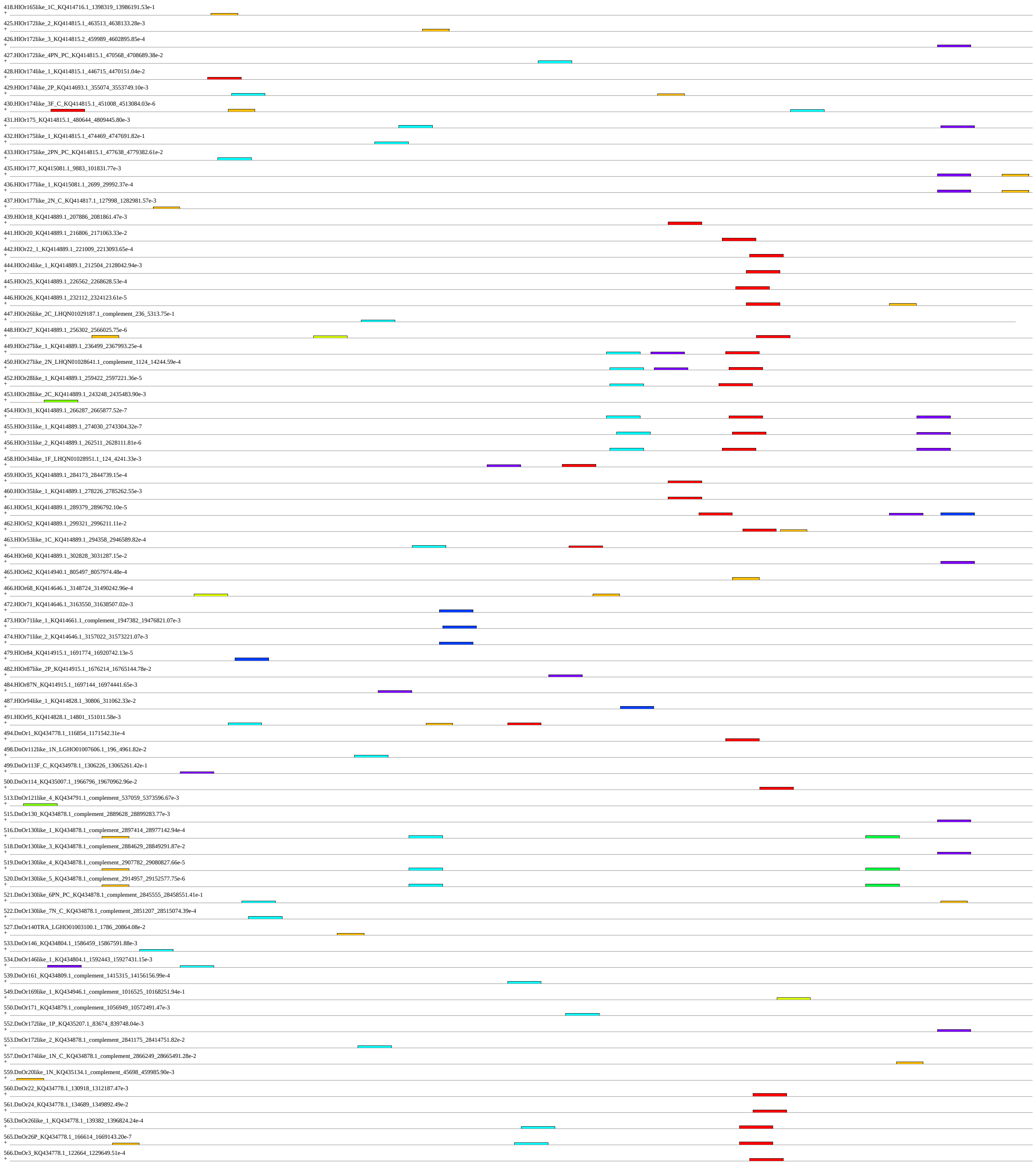
418.HlOr165like_1C_KQ414716.1_1398319_13986191.53e-1
425.HlOr172like_2_KQ414815.1_463513_4638133.28e-3
426.HlOr172like_3_KQ414815.2_459989_4602895.85e-4
427.HlOr172like_4PN_PC_KQ414815.1_470568_4708689.38e-2
428.HlOr174like_1_KQ414815.1_446715_4470151.04e-2
429.HlOr174like_2P_KQ414693.1_355074_3553749.10e-3
430.HlOr174like_3F_C_KQ414815.1_451008_4513084.03e-6
431.HlOr175_KQ414815.1_480644_4809445.80e-3
432.HlOr175like_1_KQ414815.1_474469_4747691.82e-1
433.HlOr175like_2PN_PC_KQ414815.1_477638_4779382.61e-2
435.HlOr177_KQ415081.1_9883_101831.77e-3
436.HlOr177like_1_KQ415081.1_2699_29992.37e-4
437.HlOr177like_2N_C_KQ414817.1_127998_1282981.57e-3
439.HlOr18_KQ414889.1_207886_2081861.47e-3
441.HlOr20_KQ414889.1_216806_2171063.33e-2
442.HlOr22_1_KQ414889.1_221009_2213093.65e-4
444.HlOr24like_1_KQ414889.1_212504_2128042.94e-3
445.HlOr25_KQ414889.1_226562_2268628.53e-4
446.HlOr26_KQ414889.1_232112_2324123.61e-5
447.HlOr26like_2C_LHQN01029187.1_complement_236_5313.75e-1
448.HlOr27_KQ414889.1_256302_2566025.75e-6
449.HlOr27like_1_KQ414889.1_236499_2367993.25e-4
450.HlOr27like_2N_LHQN01028641.1_complement_1124_14244.59e-4
452.HlOr28like_1_KQ414889.1_259422_2597221.36e-5
453.HlOr28like_2C_KQ414889.1_243248_2435483.90e-3
454.HlOr31_KQ414889.1_266287_2665877.52e-7
455.HlOr31like_1_KQ414889.1_274030_2743304.32e-7
456.HlOr31like_2_KQ414889.1_262511_2628111.81e-6
458.HlOr34like_1F_LHQN01028951.1_124_4241.33e-3
459.HlOr35_KQ414889.1_284173_2844739.15e-4
460.HlOr35like_1_KQ414889.1_278226_2785262.55e-3
461.HlOr51_KQ414889.1_289379_2896792.10e-5
462.HlOr52_KQ414889.1_299321_2996211.11e-2
463.HlOr53like_1C_KQ414889.1_294358_2946589.82e-4
464.HlOr60_KQ414889.1_302828_3031287.15e-2
465.HlOr62_KQ414940.1_805497_8057974.48e-4
466.HlOr68_KQ414646.1_3148724_31490242.96e-4
472.HlOr71_KQ414646.1_3163550_31638507.02e-3
473.HlOr71like_1_KQ414661.1_complement_1947382_19476821.07e-3
474.HlOr71like_2_KQ414646.1_3157022_31573221.07e-3
479.HlOr84_KQ414915.1_1691774_16920742.13e-5
482.HlOr87like_2P_KQ414915.1_1676214_16765144.78e-2
484.HlOr87N_KQ414915.1_1697144_16974441.65e-3
487.HlOr94like_1_KQ414828.1_30806_311062.33e-2
491.HlOr95_KQ414828.1_14801_151011.58e-3
494.DnOr1_KQ434778.1_116854_1171542.31e-4
498.DnOr112like_1N_LGHO01007606.1_196_4961.82e-2
499.DnOr113F_C_KQ434978.1_1306226_13065261.42e-1
500.DnOr114_KQ435007.1_1966796_19670962.96e-2
513.DnOr121like_4_KQ434791.1_complement_537059_5373596.67e-3
515.DnOr130_KQ434878.1_complement_2889628_28899283.77e-3
516.DnOr130like_1_KQ434878.1_complement_2897414_28977142.94e-4
518.DnOr130like_3_KQ434878.1_complement_2884629_28849291.87e-2
519.DnOr130like_4_KQ434878.1_complement_2907782_29080827.66e-5
520.DnOr130like_5_KQ434878.1_complement_2914957_29152577.75e-6
521.DnOr130like_6PN_PC_KQ434878.1_complement_2845555_28458551.41e-1
522.DnOr130like_7N_C_KQ434878.1_complement_2851207_28515074.39e-4
527.DnOr140TRA_LGHO01003100.1_1786_20864.08e-2
533.DnOr146_KQ434804.1_1586459_15867591.88e-3
534.DnOr146like_1_KQ434804.1_1592443_15927431.15e-3
539.DnOr161_KQ434809.1_complement_1415315_14156156.99e-4
549.DnOr169like_1_KQ434946.1_complement_1016525_10168251.94e-1
550.DnOr171_KQ434879.1_complement_1056949_10572491.47e-3
552.DnOr172like_1P_KQ435207.1_83674_839748.04e-3
553.DnOr172like_2_KQ434878.1_complement_2841175_28414751.82e-2
557.DnOr174like_1N_C_KQ434878.1_complement_2866249_28665491.28e-2
559.DnOr20like_1N_KQ435134.1_complement_45698_459985.90e-3
560.DnOr22_KQ434778.1_130918_1312187.47e-3
561.DnOr24_KQ434778.1_134689_1349892.49e-2
563.DnOr26like_1_KQ434778.1_139382_1396824.24e-4
565.DnOr26P_KQ434778.1_166614_1669143.20e-7
566.DnOr3_KQ434778.1_122664_1229649.51e-4

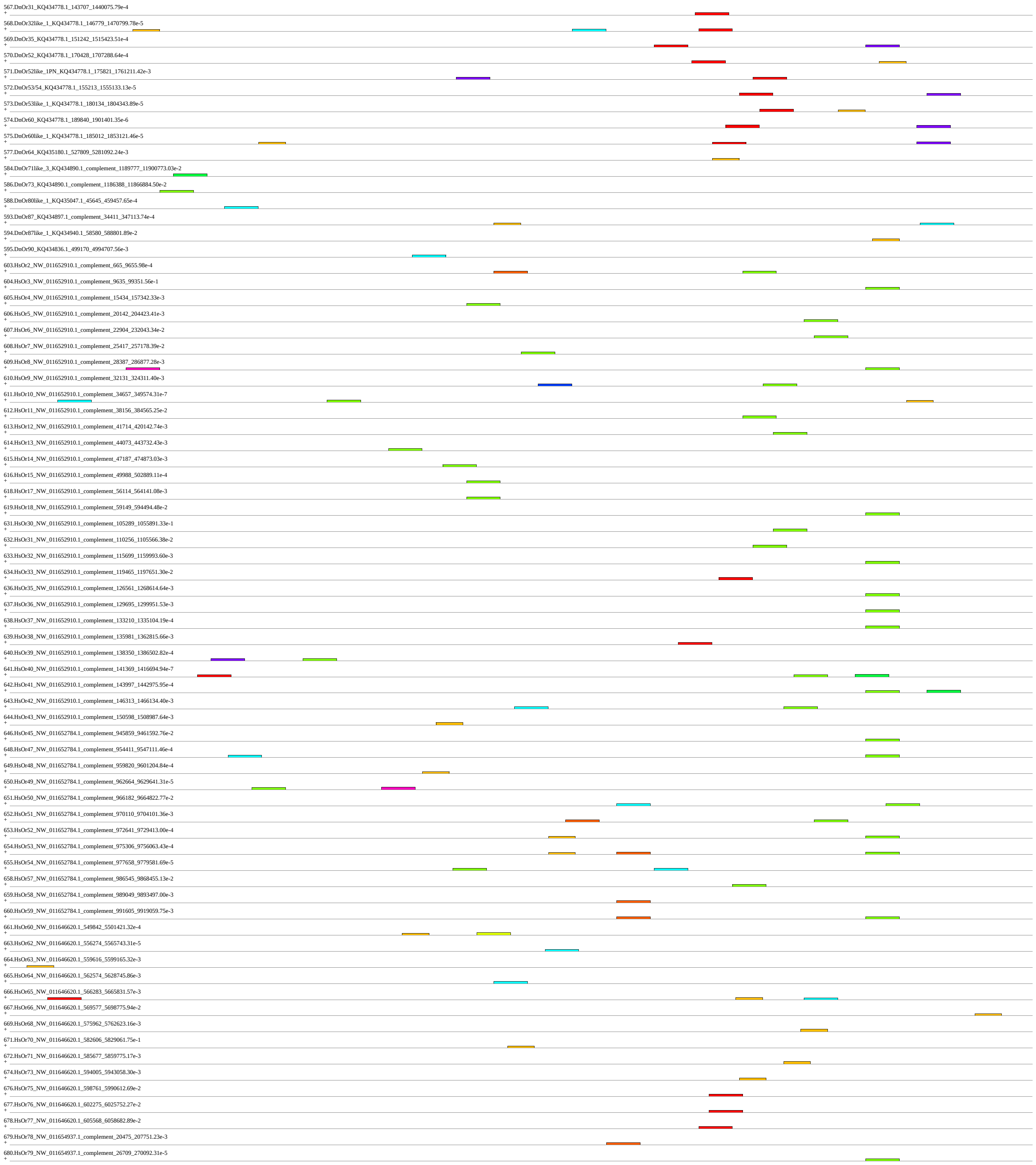
567.DnOr31_KQ434778.1_143707_1440075.79e-4
568.DnOr32like_1_KQ434778.1_146779_1470799.78e-5
569.DnOr35_KQ434778.1_151242_1515423.51e-4
570.DnOr52_KQ434778.1_170428_1707288.64e-4
571.DnOr52like_1PN_KQ434778.1_175821_1761211.42e-3
572.DnOr53/54_KQ434778.1_155213_1555133.13e-5
573.DnOr53like_1_KQ434778.1_180134_1804343.89e-5
574.DnOr60_KQ434778.1_189840_1901401.35e-6
575.DnOr60like_1_KQ434778.1_185012_1853121.46e-5
577.DnOr64_KQ435180.1_527809_5281092.24e-3
584.DnOr71like_3_KQ434890.1_complement_1189777_11900773.03e-2
586.DnOr73_KQ434890.1_complement_1186388_11866884.50e-2
588.DnOr80like_1_KQ435047.1_45645_459457.65e-4
593.DnOr87_KQ434897.1_complement_34411_347113.74e-4
594.DnOr87like_1_KQ434940.1_58580_588801.89e-2
595.DnOr90_KQ434836.1_499170_4994707.56e-3
603.HsOr2_NW_011652910.1_complement_665_9655.98e-4
604.HsOr3_NW_011652910.1_complement_9635_99351.56e-1
605.HsOr4_NW_011652910.1_complement_15434_157342.33e-3
606.HsOr5_NW_011652910.1_complement_20142_204423.41e-3
607.HsOr6_NW_011652910.1_complement_22904_232043.34e-2
608.HsOr7_NW_011652910.1_complement_25417_257178.39e-2
609.HsOr8_NW_011652910.1_complement_28387_286877.28e-3
610.HsOr9_NW_011652910.1_complement_32131_324311.40e-3
611.HsOr10_NW_011652910.1_complement_34657_349574.31e-7
612.HsOr11_NW_011652910.1_complement_38156_384565.25e-2
613.HsOr12_NW_011652910.1_complement_41714_420142.74e-3
614.HsOr13_NW_011652910.1_complement_44073_443732.43e-3
615.HsOr14_NW_011652910.1_complement_47187_474873.03e-3
616.HsOr15_NW_011652910.1_complement_49988_502889.11e-4
618.HsOr17_NW_011652910.1_complement_56114_564141.08e-3
619.HsOr18_NW_011652910.1_complement_59149_594494.48e-2
631.HsOr30_NW_011652910.1_complement_105289_1055891.33e-1
632.HsOr31_NW_011652910.1_complement_110256_1105566.38e-2
633.HsOr32_NW_011652910.1_complement_115699_1159993.60e-3
634.HsOr33_NW_011652910.1_complement_119465_1197651.30e-2
636.HsOr35_NW_011652910.1_complement_126561_1268614.64e-3
637.HsOr36_NW_011652910.1_complement_129695_1299951.53e-3
638.HsOr37_NW_011652910.1_complement_133210_1335104.19e-4
639.HsOr38_NW_011652910.1_complement_135981_1362815.66e-3
640.HsOr39_NW_011652910.1_complement_138350_1386502.82e-4
641.HsOr40_NW_011652910.1_complement_141369_1416694.94e-7
642.HsOr41_NW_011652910.1_complement_143997_1442975.95e-4
643.HsOr42_NW_011652910.1_complement_146313_1466134.40e-3
644.HsOr43_NW_011652910.1_complement_150598_1508987.64e-3
646.HsOr45_NW_011652784.1_complement_945859_9461592.76e-2
648.HsOr47_NW_011652784.1_complement_954411_9547111.46e-4
649.HsOr48_NW_011652784.1_complement_959820_9601204.84e-4
650.HsOr49_NW_011652784.1_complement_962664_9629641.31e-5
651.HsOr50_NW_011652784.1_complement_966182_9664822.77e-2
652.HsOr51_NW_011652784.1_complement_970110_9704101.36e-3
653.HsOr52_NW_011652784.1_complement_972641_9729413.00e-4
654.HsOr53_NW_011652784.1_complement_975306_9756063.43e-4
655.HsOr54_NW_011652784.1_complement_977658_9779581.69e-5
658.HsOr57_NW_011652784.1_complement_986545_9868455.13e-2
659.HsOr58_NW_011652784.1_complement_989049_9893497.00e-3
660.HsOr59_NW_011652784.1_complement_991605_9919059.75e-3
661.HsOr60_NW_011646620.1_549842_5501421.32e-4
663.HsOr62_NW_011646620.1_556274_5565743.31e-5
664.HsOr63_NW_011646620.1_559616_5599165.32e-3
665.HsOr64_NW_011646620.1_562574_5628745.86e-3
666.HsOr65_NW_011646620.1_566283_5665831.57e-3
667.HsOr66_NW_011646620.1_569577_5698775.94e-2
669.HsOr68_NW_011646620.1_575962_5762623.16e-3
671.HsOr70_NW_011646620.1_582606_5829061.75e-1
672.HsOr71_NW_011646620.1_585677_5859775.17e-3
674.HsOr73_NW_011646620.1_594005_5943058.30e-3
676.HsOr75_NW_011646620.1_598761_5990612.69e-2
677.HsOr76_NW_011646620.1_602275_6025752.27e-2
678.HsOr77_NW_011646620.1_605568_6058682.89e-2
679.HsOr78_NW_011654937.1_complement_20475_207751.23e-3
680.HsOr79_NW_011654937.1_complement_26709_270092.31e-5

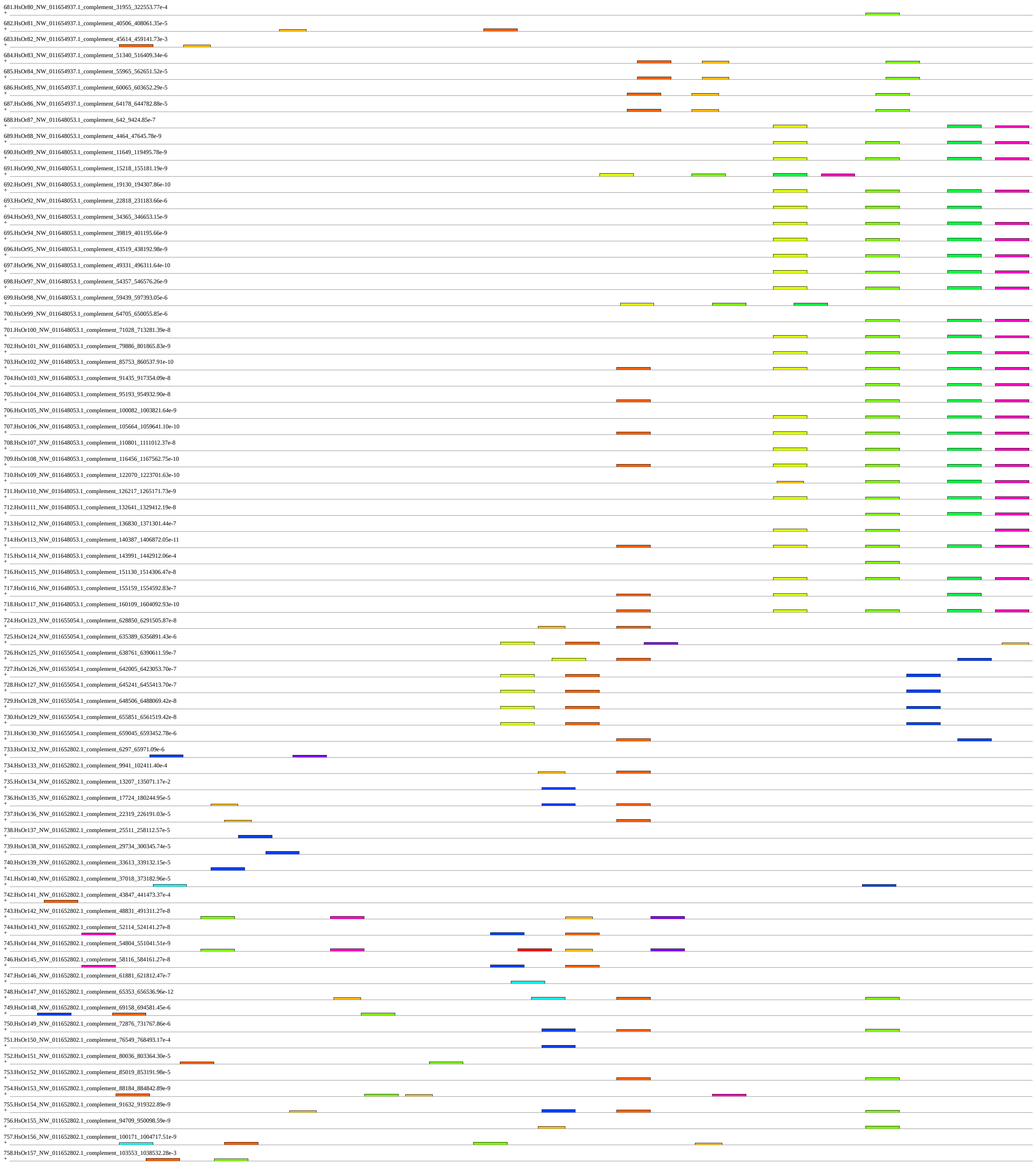
681.HsOr80_NW_011654937.1_complement_31955_322553.77e-4
+
682.HsOr81_NW_011654937.1_complement_40506_408061.35e-5
+
683.HsOr82_NW_011654937.1_complement_45614_459141.73e-3
+
684.HsOr83_NW_011654937.1_complement_51340_516409.34e-6
+
685.HsOr84_NW_011654937.1_complement_55965_562651.52e-5
+
686.HsOr85_NW_011654937.1_complement_60065_603652.29e-5
+
687.HsOr86_NW_011654937.1_complement_64178_644782.88e-5
+
688.HsOr87_NW_011648053.1_complement_642_9424.85e-7
+
689.HsOr88_NW_011648053.1_complement_4464_47645.78e-9
+
690.HsOr89_NW_011648053.1_complement_11649_119495.78e-9
+
691.HsOr90_NW_011648053.1_complement_15218_155181.19e-9
+
692.HsOr91_NW_011648053.1_complement_19130_194307.86e-10
+
693.HsOr92_NW_011648053.1_complement_22818_231183.66e-6
+
694.HsOr93_NW_011648053.1_complement_34365_346653.15e-9
+
695.HsOr94_NW_011648053.1_complement_39819_401195.66e-9
+
696.HsOr95_NW_011648053.1_complement_43519_438192.98e-9
+
697.HsOr96_NW_011648053.1_complement_49331_496311.64e-10
+
698.HsOr97_NW_011648053.1_complement_54357_546576.26e-9
+
699.HsOr98_NW_011648053.1_complement_59439_597393.05e-6
+
700.HsOr99_NW_011648053.1_complement_64705_650055.85e-6
+
701.HsOr100_NW_011648053.1_complement_71028_713281.39e-8
+
702.HsOr101_NW_011648053.1_complement_79886_801865.83e-9
+
703.HsOr102_NW_011648053.1_complement_85753_860537.91e-10
+
704.HsOr103_NW_011648053.1_complement_91435_917354.09e-8
+
705.HsOr104_NW_011648053.1_complement_95193_954932.90e-8
+
706.HsOr105_NW_011648053.1_complement_100082_1003821.64e-9
+
707.HsOr106_NW_011648053.1_complement_105664_1059641.10e-10
+
708.HsOr107_NW_011648053.1_complement_110801_1111012.37e-8
+
709.HsOr108_NW_011648053.1_complement_116456_1167562.75e-10
+
710.HsOr109_NW_011648053.1_complement_122070_1223701.63e-10
+
711.HsOr110_NW_011648053.1_complement_126217_1265171.73e-9
+
712.HsOr111_NW_011648053.1_complement_132641_1329412.19e-8
+
713.HsOr112_NW_011648053.1_complement_136830_1371301.44e-7
+
714.HsOr113_NW_011648053.1_complement_140387_1406872.05e-11
+
715.HsOr114_NW_011648053.1_complement_143991_1442912.06e-4
+
716.HsOr115_NW_011648053.1_complement_151130_1514306.47e-8
+
717.HsOr116_NW_011648053.1_complement_155159_1554592.83e-7
+
718.HsOr117_NW_011648053.1_complement_160109_1604092.93e-10
+
724.HsOr123_NW_011655054.1_complement_628850_6291505.87e-8
+
725.HsOr124_NW_011655054.1_complement_635389_6356891.43e-6
+
726.HsOr125_NW_011655054.1_complement_638761_6390611.59e-7
+
727.HsOr126_NW_011655054.1_complement_642005_6423053.70e-7
+
728.HsOr127_NW_011655054.1_complement_645241_6455413.70e-7
+
729.HsOr128_NW_011655054.1_complement_648506_6488069.42e-8
+
730.HsOr129_NW_011655054.1_complement_655851_6561519.42e-8
+
731.HsOr130_NW_011655054.1_complement_659045_6593452.78e-6
+
733.HsOr132_NW_011652802.1_complement_6297_65971.09e-6
+
734.HsOr133_NW_011652802.1_complement_9941_102411.40e-4
+
735.HsOr134_NW_011652802.1_complement_13207_135071.17e-2
+
736.HsOr135_NW_011652802.1_complement_17724_180244.95e-5
+
737.HsOr136_NW_011652802.1_complement_22319_226191.03e-5
+
738.HsOr137_NW_011652802.1_complement_25511_258112.57e-5
+
739.HsOr138_NW_011652802.1_complement_29734_300345.74e-5
+
740.HsOr139_NW_011652802.1_complement_33613_339132.15e-5
+
741.HsOr140_NW_011652802.1_complement_37018_373182.96e-5
+
742.HsOr141_NW_011652802.1_complement_43847_441473.37e-4
+
743.HsOr142_NW_011652802.1_complement_48831_491311.27e-8
+
744.HsOr143_NW_011652802.1_complement_52114_524141.27e-8
+
745.HsOr144_NW_011652802.1_complement_54804_551041.51e-9
+
746.HsOr145_NW_011652802.1_complement_58116_584161.27e-8
+
747.HsOr146_NW_011652802.1_complement_61881_621812.47e-7
+
748.HsOr147_NW_011652802.1_complement_65353_656536.96e-12
+
749.HsOr148_NW_011652802.1_complement_69158_694581.45e-6
+
750.HsOr149_NW_011652802.1_complement_72876_731767.86e-6
+
751.HsOr150_NW_011652802.1_complement_76549_768493.17e-4
+
752.HsOr151_NW_011652802.1_complement_80036_803364.30e-5
+
753.HsOr152_NW_011652802.1_complement_85019_853191.98e-5
+
754.HsOr153_NW_011652802.1_complement_88184_884842.89e-9
+
755.HsOr154_NW_011652802.1_complement_91632_919322.89e-9
+
756.HsOr155_NW_011652802.1_complement_94709_950098.59e-9
+
757.HsOr156_NW_011652802.1_complement_100171_1004717.51e-9
+
758.HsOr157_NW_011652802.1_complement_103553_1038532.28e-3
+

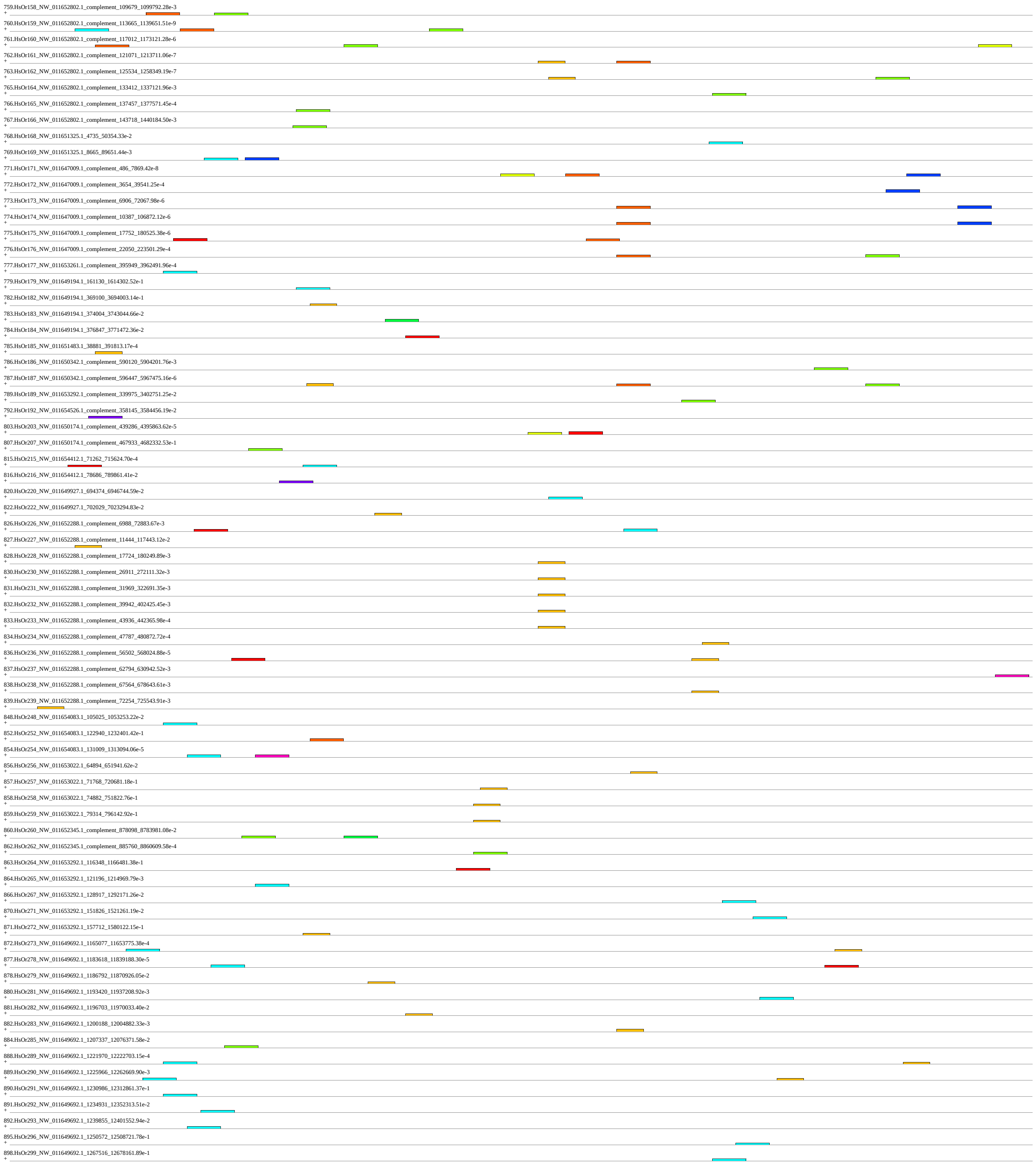

759.HsOr158_NW_011652802.1_complement_109679_1099792.28e-3
+
760.HsOr159_NW_011652802.1_complement_113665_1139651.51e-9
+
761.HsOr160_NW_011652802.1_complement_117012_1173121.28e-6
+
762.HsOr161_NW_011652802.1_complement_121071_1213711.06e-7
+
763.HsOr162_NW_011652802.1_complement_125534_1258349.19e-7
+
765.HsOr164_NW_011652802.1_complement_133412_1337121.96e-3
+
766.HsOr165_NW_011652802.1_complement_137457_1377571.45e-4
+
767.HsOr166_NW_011652802.1_complement_143718_1440184.50e-3
+
768.HsOr168_NW_011651325.1_4735_50354.33e-2
+
769.HsOr169_NW_011651325.1_8665_89651.44e-3
+
771.HsOr171_NW_011647009.1_complement_486_7869.42e-8
+
772.HsOr172_NW_011647009.1_complement_3654_39541.25e-4
+
773.HsOr173_NW_011647009.1_complement_6906_72067.98e-6
+
774.HsOr174_NW_011647009.1_complement_10387_106872.12e-6
+
775.HsOr175_NW_011647009.1_complement_17752_180525.38e-6
+
776.HsOr176_NW_011647009.1_complement_22050_223501.29e-4
+
777.HsOr177_NW_011653261.1_complement_395949_3962491.96e-4
+
779.HsOr179_NW_011649194.1_161130_1614302.52e-1
+
782.HsOr182_NW_011649194.1_369100_3694003.14e-1
+
783.HsOr183_NW_011649194.1_374004_3743044.66e-2
+
784.HsOr184_NW_011649194.1_376847_3771472.36e-2
+
785.HsOr185_NW_011651483.1_38881_391813.17e-4
+
786.HsOr186_NW_011650342.1_complement_590120_5904201.76e-3
+
787.HsOr187_NW_011650342.1_complement_596447_5967475.16e-6
+
789.HsOr189_NW_011653292.1_complement_339975_3402751.25e-2
+
792.HsOr192_NW_011654526.1_complement_358145_3584456.19e-2
+
803.HsOr203_NW_011650174.1_complement_439286_4395863.62e-5
+
807.HsOr207_NW_011650174.1_complement_467933_4682332.53e-1
+
815.HsOr215_NW_011654412.1_71262_715624.70e-4
+
816.HsOr216_NW_011654412.1_78686_789861.41e-2
+
820.HsOr220_NW_011649927.1_694374_6946744.59e-2
+
822.HsOr222_NW_011649927.1_702029_7023294.83e-2
+
826.HsOr226_NW_011652288.1_complement_6988_72883.67e-3
+
827.HsOr227_NW_011652288.1_complement_11444_117443.12e-2
+
828.HsOr228_NW_011652288.1_complement_17724_180249.89e-3
+
830.HsOr230_NW_011652288.1_complement_26911_272111.32e-3
+
831.HsOr231_NW_011652288.1_complement_31969_322691.35e-3
+
832.HsOr232_NW_011652288.1_complement_39942_402425.45e-3
+
833.HsOr233_NW_011652288.1_complement_43936_442365.98e-4
+
834.HsOr234_NW_011652288.1_complement_47787_480872.72e-4
+
836.HsOr236_NW_011652288.1_complement_56502_568024.88e-5
+
837.HsOr237_NW_011652288.1_complement_62794_630942.52e-3
+
838.HsOr238_NW_011652288.1_complement_67564_678643.61e-3
+
839.HsOr239_NW_011652288.1_complement_72254_725543.91e-3
+
848.HsOr248_NW_011654083.1_105025_1053253.22e-2
+
852.HsOr252_NW_011654083.1_122940_1232401.42e-1
+
854.HsOr254_NW_011654083.1_131009_1313094.06e-5
+
856.HsOr256_NW_011653022.1_64894_651941.62e-2
+
857.HsOr257_NW_011653022.1_71768_720681.18e-1
+
858.HsOr258_NW_011653022.1_74882_751822.76e-1
+
859.HsOr259_NW_011653022.1_79314_796142.92e-1
+
860.HsOr260_NW_011652345.1_complement_878098_8783981.08e-2
+
862.HsOr262_NW_011652345.1_complement_885760_8860609.58e-4
+
863.HsOr264_NW_011653292.1_116348_1166481.38e-1
+
864.HsOr265_NW_011653292.1_121196_1214969.79e-3
+
866.HsOr267_NW_011653292.1_128917_1292171.26e-2
+
870.HsOr271_NW_011653292.1_151826_1521261.19e-2
+
871.HsOr272_NW_011653292.1_157712_1580122.15e-1
+
872.HsOr273_NW_011649692.1_1165077_11653775.38e-4
+
877.HsOr278_NW_011649692.1_1183618_11839188.30e-5
+
878.HsOr279_NW_011649692.1_1186792_11870926.05e-2
+
880.HsOr281_NW_011649692.1_1193420_11937208.92e-3
+
881.HsOr282_NW_011649692.1_1196703_11970033.40e-2
+
882.HsOr283_NW_011649692.1_1200188_12004882.33e-3
+
884.HsOr285_NW_011649692.1_1207337_12076371.58e-2
+
888.HsOr289_NW_011649692.1_1221970_12222703.15e-4
+
889.HsOr290_NW_011649692.1_1225966_12262669.90e-3
+
890.HsOr291_NW_011649692.1_1230986_12312861.37e-1
+
891.HsOr292_NW_011649692.1_1234931_12352313.51e-2
+
892.HsOr293_NW_011649692.1_1239855_12401552.94e-2
+
895.HsOr296_NW_011649692.1_1250572_12508721.78e-1
+
898.HsOr299_NW_011649692.1_1267516_12678161.89e-1
+

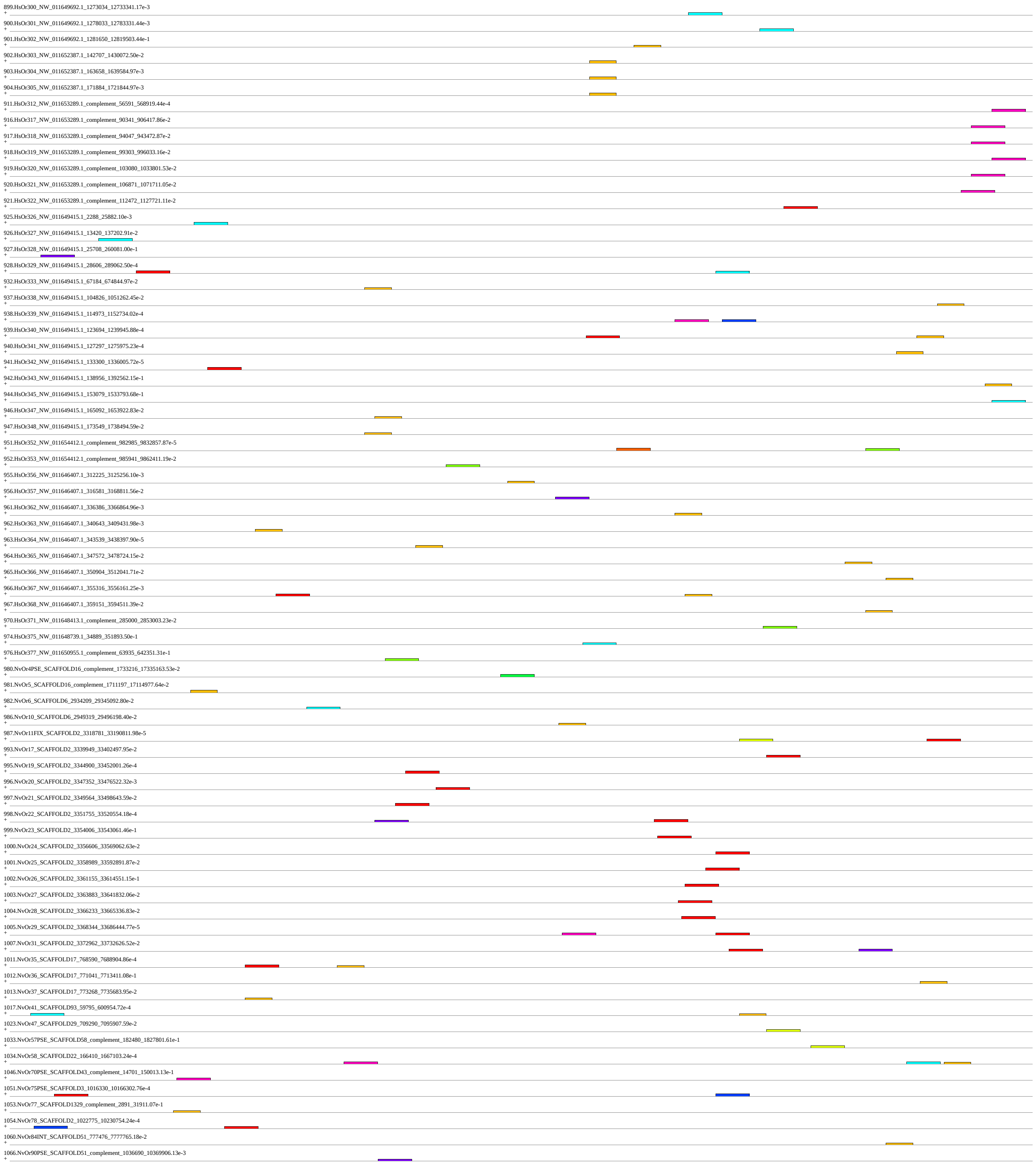

899.HsOr300_NW_011649692.1_1273034_12733341.17e-3
+
900.HsOr301_NW_011649692.1_1278033_12783331.44e-3
+
901.HsOr302_NW_011649692.1_1281650_12819503.44e-1
+
902.HsOr303_NW_011652387.1_142707_1430072.50e-2
+
903.HsOr304_NW_011652387.1_163658_1639584.97e-3
+
904.HsOr305_NW_011652387.1_171884_1721844.97e-3
+
911.HsOr312_NW_011653289.1_complement_56591_568919.44e-4
+
916.HsOr317_NW_011653289.1_complement_90341_906417.86e-2
+
917.HsOr318_NW_011653289.1_complement_94047_943472.87e-2
+
918.HsOr319_NW_011653289.1_complement_99303_996033.16e-2
+
919.HsOr320_NW_011653289.1_complement_103080_1033801.53e-2
+
920.HsOr321_NW_011653289.1_complement_106871_1071711.05e-2
+
921.HsOr322_NW_011653289.1_complement_112472_1127721.11e-2
+
925.HsOr326_NW_011649415.1_2288_25882.10e-3
+
926.HsOr327_NW_011649415.1_13420_137202.91e-2
+
927.HsOr328_NW_011649415.1_25708_260081.00e-1
+
928.HsOr329_NW_011649415.1_28606_289062.50e-4
+
932.HsOr333_NW_011649415.1_67184_674844.97e-2
+
937.HsOr338_NW_011649415.1_104826_1051262.45e-2
+
938.HsOr339_NW_011649415.1_114973_1152734.02e-4
+
939.HsOr340_NW_011649415.1_123694_1239945.88e-4
+
940.HsOr341_NW_011649415.1_127297_1275975.23e-4
+
941.HsOr342_NW_011649415.1_133300_1336005.72e-5
+
942.HsOr343_NW_011649415.1_138956_1392562.15e-1
+
944.HsOr345_NW_011649415.1_153079_1533793.68e-1
+
946.HsOr347_NW_011649415.1_165092_1653922.83e-2
+
947.HsOr348_NW_011649415.1_173549_1738494.59e-2
+
951.HsOr352_NW_011654412.1_complement_982985_9832857.87e-5
+
952.HsOr353_NW_011654412.1_complement_985941_9862411.19e-2
+
955.HsOr356_NW_011646407.1_312225_3125256.10e-3
+
956.HsOr357_NW_011646407.1_316581_3168811.56e-2
+
961.HsOr362_NW_011646407.1_336386_3366864.96e-3
+
962.HsOr363_NW_011646407.1_340643_3409431.98e-3
+
963.HsOr364_NW_011646407.1_343539_3438397.90e-5
+
964.HsOr365_NW_011646407.1_347572_3478724.15e-2
+
965.HsOr366_NW_011646407.1_350904_3512041.71e-2
+
966.HsOr367_NW_011646407.1_355316_3556161.25e-3
+
967.HsOr368_NW_011646407.1_359151_3594511.39e-2
+
970.HsOr371_NW_011648413.1_complement_285000_2853003.23e-2
+
974.HsOr375_NW_011648739.1_34889_351893.50e-1
+
976.HsOr377_NW_011650955.1_complement_63935_642351.31e-1
+
980.NvOr4PSE_SCAFFOLD16_complement_1733216_17335163.53e-2
+
981.NvOr5_SCAFFOLD16_complement_1711197_17114977.64e-2
+
982.NvOr6_SCAFFOLD6_2934209_29345092.80e-2
+
986.NvOr10_SCAFFOLD6_2949319_29496198.40e-2
+
987.NvOr11FIX_SCAFFOLD2_3318781_33190811.98e-5
+
993.NvOr17_SCAFFOLD2_3339949_33402497.95e-2
+
995.NvOr19_SCAFFOLD2_3344900_33452001.26e-4
+
996.NvOr20_SCAFFOLD2_3347352_33476522.32e-3
+
997.NvOr21_SCAFFOLD2_3349564_33498643.59e-2
+
998.NvOr22_SCAFFOLD2_3351755_33520554.18e-4
+
999.NvOr23_SCAFFOLD2_3354006_33543061.46e-1
+
1000.NvOr24_SCAFFOLD2_3356606_33569062.63e-2
+
1001.NvOr25_SCAFFOLD2_3358989_33592891.87e-2
+
1002.NvOr26_SCAFFOLD2_3361155_33614551.15e-1
+
1003.NvOr27_SCAFFOLD2_3363883_33641832.06e-2
+
1004.NvOr28_SCAFFOLD2_3366233_33665336.83e-2
+
1005.NvOr29_SCAFFOLD2_3368344_33686444.77e-5
+
1007.NvOr31_SCAFFOLD2_3372962_33732626.52e-2
+
1011.NvOr35_SCAFFOLD17_768590_7688904.86e-4
+
1012.NvOr36_SCAFFOLD17_771041_7713411.08e-1
+
1013.NvOr37_SCAFFOLD17_773268_7735683.95e-2
+
1017.NvOr41_SCAFFOLD93_59795_600954.72e-4
+
1023.NvOr47_SCAFFOLD29_709290_7095907.59e-2
+
1033.NvOr57PSE_SCAFFOLD58_complement_182480_1827801.61e-1
+
1034.NvOr58_SCAFFOLD22_166410_1667103.24e-4
+
1046.NvOr70PSE_SCAFFOLD43_complement_14701_150013.13e-1
+
1051.NvOr75PSE_SCAFFOLD3_1016330_10166302.76e-4
+
1053.NvOr77_SCAFFOLD1329_complement_2891_31911.07e-1
+
1054.NvOr78_SCAFFOLD2_1022775_10230754.24e-4
+
1060.NvOr84INT_SCAFFOLD51_777476_7777765.18e-2
+
1066.NvOr90PSE_SCAFFOLD51_complement_1036690_10369906.13e-3
+

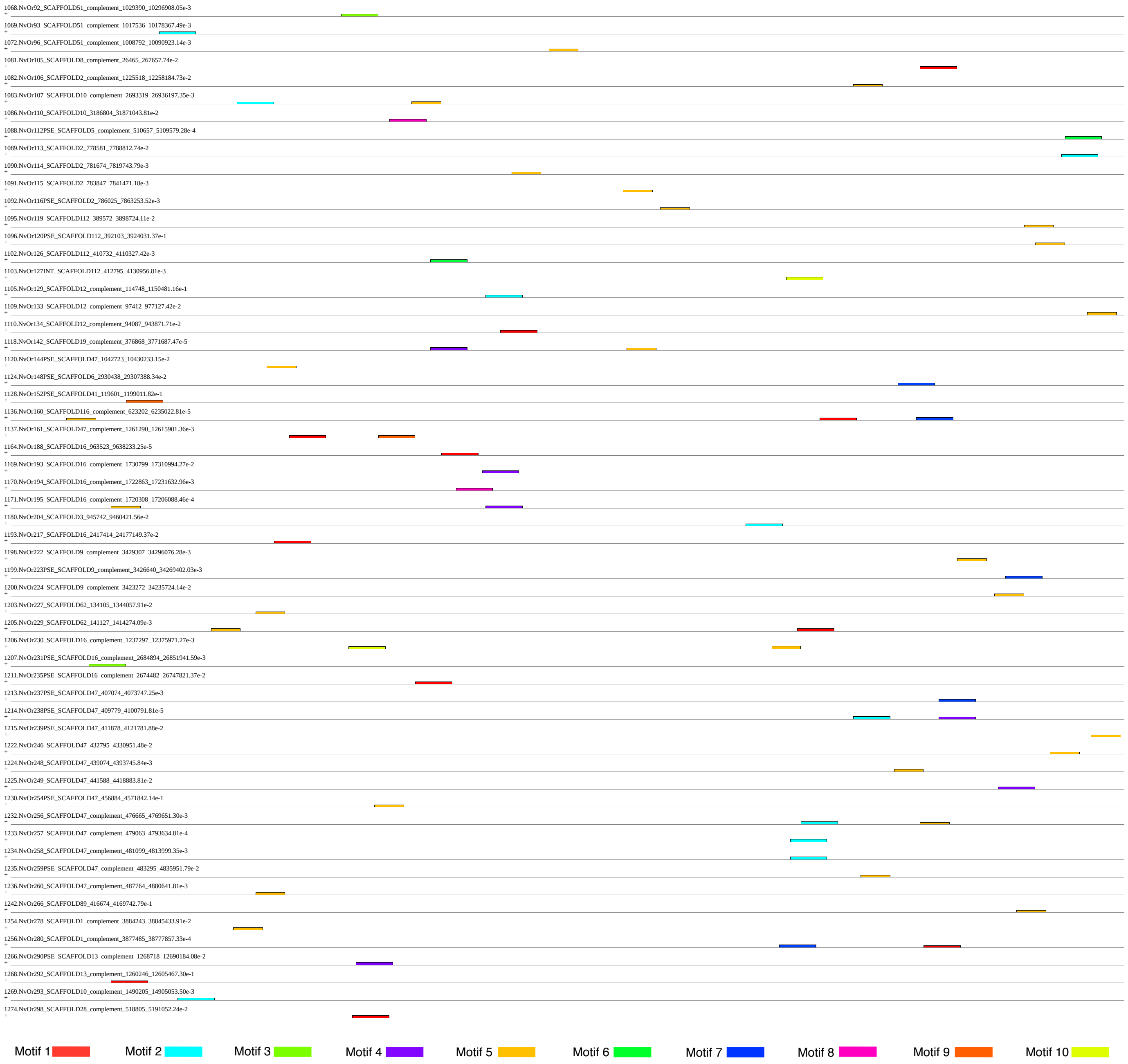
1068.NvOr92_SCAFFOLD51_complement_1029390_10296908.05e-3
1069.NvOr93_SCAFFOLD51_complement_1017536_10178367.49e-3
1072.NvOr96_SCAFFOLD51_complement_1008792_10090923.14e-3
1081.NvOr105_SCAFFOLD8_complement_26465_267657.74e-2
1082.NvOr106_SCAFFOLD2_complement_1225518_12258184.73e-2
1083.NvOr107_SCAFFOLD10_complement_2693319_26936197.35e-3
1086.NvOr110_SCAFFOLD10_3186804_31871043.81e-2
1088.NvOr112PSE_SCAFFOLD5_complement_510657_5109579.28e-4
1089.NvOr113_SCAFFOLD2_778581_7788812.74e-2
1090.NvOr114_SCAFFOLD2_781674_7819743.79e-3
1091.NvOr115_SCAFFOLD2_783847_7841471.18e-3
1092.NvOr116PSE_SCAFFOLD2_786025_7863253.52e-3
1095.NvOr119_SCAFFOLD112_389572_3898724.11e-2
1096.NvOr120PSE_SCAFFOLD112_392103_3924031.37e-1
1102.NvOr126_SCAFFOLD112_410732_4110327.42e-3
1103.NvOr127INT_SCAFFOLD112_412795_4130956.81e-3
1105.NvOr129_SCAFFOLD12_complement_114748_1150481.16e-1
1109.NvOr133_SCAFFOLD12_complement_97412_977127.42e-2
1110.NvOr134_SCAFFOLD12_complement_94087_943871.71e-2
1118.NvOr142_SCAFFOLD19_complement_376868_3771687.47e-5
1120.NvOr144PSE_SCAFFOLD47_1042723_10430233.15e-2
1124.NvOr148PSE_SCAFFOLD6_2930438_29307388.34e-2
1128.NvOr152PSE_SCAFFOLD41_119601_1199011.82e-1
1136.NvOr160_SCAFFOLD116_complement_623202_6235022.81e-5
1137.NvOr161_SCAFFOLD47_complement_1261290_12615901.36e-3
1164.NvOr188_SCAFFOLD16_963523_9638233.25e-5
1169.NvOr193_SCAFFOLD16_complement_1730799_17310994.27e-2
1170.NvOr194_SCAFFOLD16_complement_1722863_17231632.96e-3
1171.NvOr195_SCAFFOLD16_complement_1720308_17206088.46e-4
1180.NvOr204_SCAFFOLD3_945742_9460421.56e-2
1193.NvOr217_SCAFFOLD16_2417414_24177149.37e-2
1198.NvOr222_SCAFFOLD9_complement_3429307_34296076.28e-3
1199.NvOr223PSE_SCAFFOLD9_complement_3426640_34269402.03e-3
1200.NvOr224_SCAFFOLD9_complement_3423272_34235724.14e-2
1203.NvOr227_SCAFFOLD62_134105_1344057.91e-2
1205.NvOr229_SCAFFOLD62_141127_1414274.09e-3
1206.NvOr230_SCAFFOLD16_complement_1237297_12375971.27e-3
1207.NvOr231PSE_SCAFFOLD16_complement_2684894_26851941.59e-3
1211.NvOr235PSE_SCAFFOLD16_complement_2674482_26747821.37e-2
1213.NvOr237PSE_SCAFFOLD47_407074_4073747.25e-3
1214.NvOr238PSE_SCAFFOLD47_409779_4100791.81e-5
1215.NvOr239PSE_SCAFFOLD47_411878_4121781.88e-2
1222.NvOr246_SCAFFOLD47_432795_4330951.48e-2
1224.NvOr248_SCAFFOLD47_439074_4393745.84e-3
1225.NvOr249_SCAFFOLD47_441588_4418883.81e-2
1230.NvOr254PSE_SCAFFOLD47_456884_4571842.14e-1
1232.NvOr256_SCAFFOLD47_complement_476665_4769651.30e-3
1233.NvOr257_SCAFFOLD47_complement_479063_4793634.81e-4
1234.NvOr258_SCAFFOLD47_complement_481099_4813999.35e-3
1235.NvOr259PSE_SCAFFOLD47_complement_483295_4835951.79e-2
1236.NvOr260_SCAFFOLD47_complement_487764_4880641.81e-3
1242.NvOr266_SCAFFOLD89_416674_4169742.79e-1
1254.NvOr278_SCAFFOLD1_complement_3884243_38845433.91e-2
1256.NvOr280_SCAFFOLD1_complement_3877485_38777857.33e-4
1266.NvOr290PSE_SCAFFOLD13_complement_1268718_12690184.08e-2
1268.NvOr292_SCAFFOLD13_complement_1260246_12605467.30e-1
1269.NvOr293_SCAFFOLD10_complement_1490205_14905053.50e-3
1274.NvOr298_SCAFFOLD28_complement_518805_5191052.24e-2
Motif 1
Motif 2
Motif 3
Motif 4
Motif 5
Motif 6
Motif 7
Motif 8
Motif 9
Motif 10

Supplementary Figure S6 - Bee ORs from 9-exon group show worker enrichment in *A. florea*

9-exon clade with six hymenopteran species is shown here. Non-bee specific subgroups are collapsed. Species follow following branch-colours-
D. novaeangliae - blue,
H. laboriosa - cyan,
A. florea - green,
A. *mellifera* - yellow,
H. saltator - magenta and
N. vitripennis - red.
9-exon-alpha group, with putative hair - like sensilla ORs, is shown in grey box. Branches of *A. florea* ORs with expression restricted to only workers are shown in dashed green lines whereas branches of the *A. florea* ORs with no expression in both sexes are shown in purple. The log2fold expression of worker against drones for these ORs is represented in heat-map on the right hand side with scale ranging from -1.8 (yellow) for AfOr162 to 6.85 (dark red) for AfOr110. The log2fold expression for ORs with no expression in drones was adjusted to be 8(dark red). Note that all worker restricted and many highly worker enriched AfOrs are present in the grey box - putative basiconic sensilla.

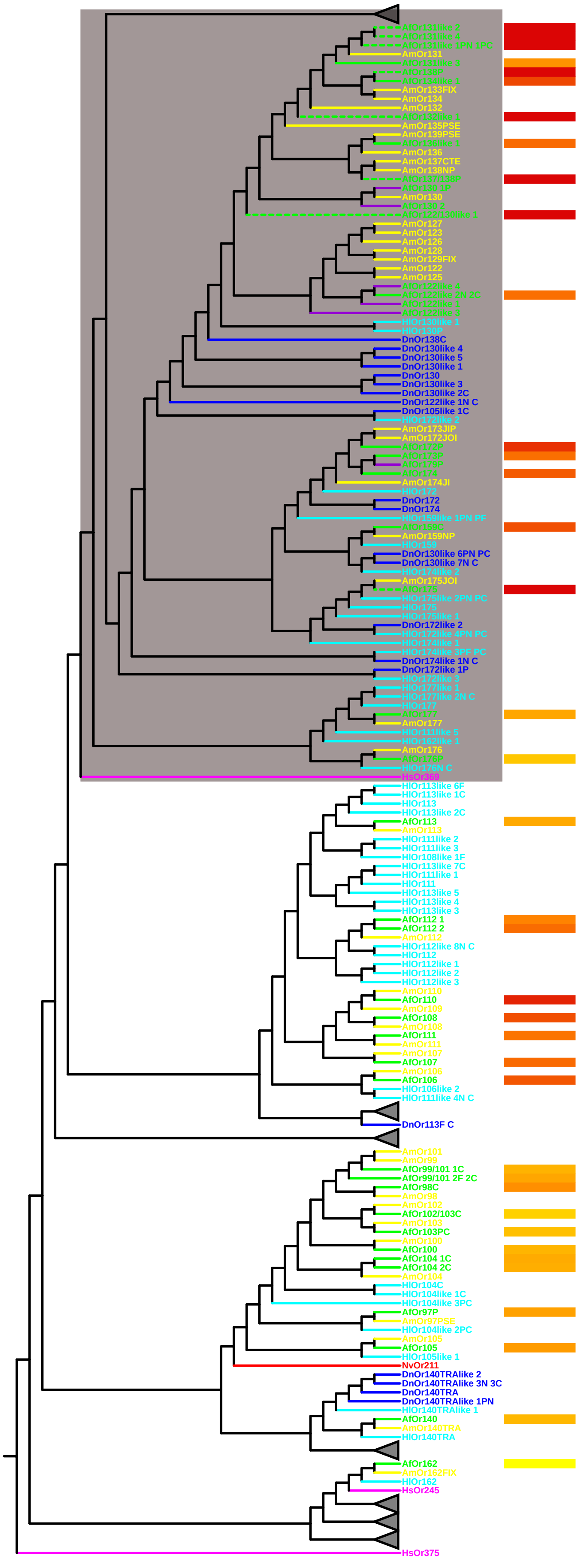

Supplementary Data S1-DnOr protein sequences

>DnOr1
MTDARNPPSYRNVHYKVDTEYTVQVAKNLLTPLGVWPLHRGDLFLDNVKSFVHIGTIFSLMCFLLI
PHIIYTYHDVEDLTRYMKVIAAQVFSFLAIAKFWTMVINKKDIRFCLTEMEAHYRDVECEEDRLVM
KNSAKVGRFFTTLYLGLAYGGALPYHIILPLMSERIVKADNTTQIPLPYLSNYIFFVIEDSPIYEI
TFVSQIVISSIILSTNCGIYSLIATIAMHSCGLFVVVGRQLDTLLEYKQDKLYDRLRNIILHHLKA
IEFADTIEMALSTVFLLEMVGCTIIICFLEYGVIMEWEDHKILSMMTYVVLMTSIFVNVFIICFIG
DCLKQESEKVGETSYLLSWYDLPEDIIKSLKMIIVRTSRPANLSASKLFELSLQAFCDVCKTSAAY
LNFLRTMTS
>DnOr2
MMKFKQQGLVADLMPNIQLMKATGHFMFNYYTDNSSKFIHKVFCFVHLFLILLQFGLCGINLMLTS
DDVDVLTANTITMLFFTHSVVKVLYFAIRSKLFYRTLAIWNNPNSHPLFAESNARFHQLAVKKMRI
LLMAVMCTTMLSVIAWTTITFIGDSTMKVVDPVTNETTYVEVPRLMLYSYYPFDPSHGMAHVLTLI
FQFYFLLFAMADANLLDVLFCSWLLFACEQIQHLKDIMKPLMEFSATLDTVVPNSGELFKASSPSH
PAESHEPPPVSDLQGDNMLDMDLRGIYNQRQDFTATFRPTAGTAFNGTVGPNGLTKKQEMLVRSAI
KYWVERHKHTVRLVTAVGDAYGIALLLHMLATTVTLTLLAYQATKINGINVYSASVIGYLLYTLGQ
VFMLCIFGNRLIEESTSVMEAAYSCHWYDGSEEAKTFVQIVCQQCQKAMSISGAKFFTVSLDLFAS
VLGAMVTYFMVLVQLK
>DnOr3
MTDTKNPNTYQNFRHKSDFVFTIRAARILLTPVGIWPLDRSSSITADVKSMIQVGVIFGLMSFLLI
PHVIYTFHDCEDLTRYMKVIAAQVFSLLGIIKYWTMIFKKQRIRSCLVKMQIQYKTVECEENRLVM
KNSAKIARFFTTIYLSLCFGGALPYHIILPLLSEKIVRADNTTQIPLPYLSNYVFFVIENSPFYEI
TFASQIVISTIILFTNCGTNSLIASMTMHSCGMFEVVNRQLETLMLDQRRDEMKASLRNVVQYHLR
AIEFADMIERNLNGIFLSEMVGCTLIICFLEYGVIMEWEDKNILSMVTYFLLMTWMFLNVYILSYI
GNYLKQESERVGVMSYFLPWYDHSEEVNKNLRMIILRATRPTCFTAAKFFDLSLRGFCDVFKTSAA
YLNFLQTMTG
>DnOr20like_1N
MKEHFWTRWHKDYLHELTVRRKWHTSTPTEVKEGALVIVHEDNVPPLCWALGRIVALHPGDDHITR
VVTIKTVAGEYKRSLKKLSLLPL
>DnOr22
MRGPETQVKNSNLSLQWSRWMLKLFGAWPYSSDMSRQKKHMQWLINIVCYFLISFLFVPCGLFVTL
EVEDTQNKLRLFGPLSFCVMAYMKYYSLLAHANDIRECIKRIEWDWRNIEHRQDRDIMMENAIHGG
RLVKICIFFMYSGFVLYYIALPMSRGKVTAENQNLTFIPMVFPISSLMADSRNSPGNEIFFSIQFF
GGIVIHGISAAACSLAAVLAVHVCGQMKVLMCWMGHLINAREDMSRSVDVRIANIVSQHVRILKFL
IVTEEALQNISFVEFVGCTLNLCLLGYYFITEWHSNDLTSAITYLTLLISLGFNIFIFCYIGELIA
EQCNKVGEMAYMVDWYRLQGKKQRCIVLIIAMSNSSTKLTAGNMVQLSLSTFGDVVKTAVGFLNML
LALM
>DnOr24
MQTPTKDPKKSSIRWNEYEENVDLSIQWNRWILKPMGVWPSSHNVSCLEKSFNRLMNVVCYGLISF
LFVPCGLYVMLEVKDTYNKLKLFGPLIFCMTAYIKYYSLMVHANDIRECIRHIEWDWKNVQHQEDR
SIMIANANFGRRLVRVCTFFMYSGFLFYYIAVPVNVGKVVAEEGNRTFVPMVFPFSRLIADTRDSP
TNEILFSIQLLGGMLIHGIAAGACSLAAAFAVHACGQMEVLKCWMEHLVDGREDMSKSVDVRIANI
VSQHVRILKFLALIEKALTQISLAEFLGCTLDICLVGYYIIVEWSSNDMTAAVTYTIILTSLIFNI
FIFCYIGELVAEQCKKVGEMSYMIDWYRLPENKKLGLVLIMAMSNSSIKLTAGNLVKLSLTSFSDV
VKTSVAFLNMLRTLT
>DnOr24like_1
MREEETKASYLSLQWTRWILIPFGVWPHSPNTSKQKKYICRLINVICYFLISFLFVPCGLFVILEV
KNVYYKLKLFGPLNFCAMAYVKYYSLMVHKKSIRECIKHIEWDWKMVNYSEDRNIMVENAIIGSRL
VKIYIFFMYSGFVFYYIALPISNGKVTVEDRNLTFIPMVFPISRLIVDSRLSPGNEIFFLIQFFGG
IVIHGITAAACSLAAALAVHACGQMKVLICWMEHLIDGREDMSRSVDVRIVNIVRQHVRILRFLFT

TEKTLQQISFVEFVGSSINLCLLGYYFFVICMYGRNTKGLNVLNKIHNANAHCSYIGELVAEQCNQ
IGEMAYMIDWYKLRGKKRRFLTLLIIMSNSSTKLTAGNMVALSLRTFGDVVKTAVGFLNILLALT
>DnOr26like_1
MEKGSHVKMVNNVHHAEDYDYSIQVNRWILQPIGVWPSINKLSKAKWFLSFLLNLACHAVIMFTVA
PCLMFILFEDESIHMRMKAIGPMSAMLMGELNYWSLSLKTKDILRCFDYLDTDWKMVGRPQDRRVM
LKNAKVGRMVVTVAAFALNIGVFSHSLMAGFKKMEFNIGNDTYAMLRLPCPCYSKLIDARYSPINE
IVFFVQVLSGLIVNLVTVGAYGFAAVFAMHVCGQLSIAMSYLDELVEPNMDQPNAQEKLGDIVDIH
LRALNFVSHIEDVMHLTCLIELTGCTLNMCMLEYYMITESSKENAVAYGVIYLSMTFNVFIFCYIG
EKISEQGEMVGEKAYMTEWYRLPYKTASGLILVILRSSTVTKITAGKVLPMSIATFASVVKTSFVY
FNMIRTVTM
>DnOr26like_2
MKGDRVDTVSNVHHVEDYNYSVQVNQWILKPSVLYILFEQADTHTRMQAIGPMSHWMLGGVNYCCL
SLKSKDILSCIEHLETDWKMVKRPNDRQLMLKNAKVGRLIVSIAAVCMNVGVLSHTLIAGFKRAVF
QVGNESYSMLRLPCPVYTQLMDVRFSPLNEIVFFLQCWAGLIVNLVTTGACGFAAVFAMHACGQLD
IVMLHLEEMVEQNEDQPSAQKKLGTMVDIHLRALDFVSHIESVMHITCLIELTECTLNMCMLEYYM
ITENSKETAAAYGIIYISMTFNIFIFCYIAEKLTEQCKMVGETAYMTEWYRLPYKTAAGLILVILR
AGSVTRITAGKILPMSLSTFADVVKTSFVYLNMLRTVAE
>DnOr26P
MVNGARVITVNNVHYVKDYNYSIQVNRWSLKPIGIWPSLSEFSRAESILSRLXHALIVFTVVPCVL
YILFEDESMHIRMKAIGPMSHWLMGELNYCSLSLKTKDILRCIDHMETDWKTVKRPHDRQLMLKSA
KIGRIIMTVAALCMNIGVFSYNFVTGLKKTVLHVGNESYSTLRLPCPFYTKLMDVRYSPANEIVFF
LQCFSGLIVNSVTVGACGLAAVFAMHACGQLNIVMSQLEDLVERNKDQPSAQRKMASIVDIHLRAL
NFVSHIENVMHLVCFVELTGCTLNMCMLEYYMLTENSKETIATYGIIYASMTFNIFIFCYIAEKLT
EQCKRVGEKAYMTEWYRLPHKTAAALILVISRSGLITKITAGKILPMSLATFGDVVKTSFMYLNML
RTVAV
>DnOr31
MASESVTFEADNLNDYSLQLSRWYLLPIGAWPSSSSASRLERIVSVTLIVLSYSLIMFTVVPSFLS
IILEDETVRLKLKLLGPLGHWFVGGINYTTLLMRSKEIRHCIEHMGTDWRIITRTKDQQIMMKNAK
FGRYVSAFCAACMQGGVLSYCVVSALTMRVVQVGNETRTVHVLPCSVYKKLLNVDNSPMNEIVLVS
QFLSAFIVNSSAVGAFSLAAVFAAHACGQLDVLMIWITEFVNDSRRQRKNACLNKIGVLVEHHLRV
LSFISRIEDVMTRICFMEMFKCTLDICMLGYYILTEWSDKDVRNLTTYFVILMSMCFNIFTVCYIG
EILSEQCIKVGEVVYMSNWYHLPDKLVLDLVLIITRSSVVVKITAGKLIRMSIYTFGDVMKTSFAY
LNLLRQTT
>DnOr32like_1
MLGEATMIERDLGDPSDYSLQLNRWYLKPIGAWPTLPSTSRLERIVAIVLILLCYCSISFTVVPCM
LHILLEDEDIRMKLRAAGPLSHWFAGGINYTTLLMRSNEIRHCVEHLQTDWRLVTRVKDRQVMLKN
AKNGRYVAVCCAAFMQAGVLSYCAVTAWSTRIVENGNRTGIVHVLPCVLQTSVVGIFSLAAVFTAH
ACGQLNVLMAWITELVNESRGRNARIYLNEIGVVVEHHLRVLSFISRIEDVMTRICFLELVRCSFG
ICMLGYYILTEWSDHNFQNLTTYFMIMGSMTFNVFIVCYIGELLTEQCKKVGEVIYMTEWYCLPHK
CVLDLTMIIARSSLLIKITAAKMIHMSVSTFGDVMKTTFAYLNLLHQMV
>DnOr35
MATTDGDNRGDDWMHSVRINRWLLKPIGVWPVSLCVDTMEKMITVLLVLISASLIGFLLVPCALCT
ILDKTDDLDMKIKMIGPLSFCVMAAIKYCILVSRGGQISKCIRIIRSDWTRTNMDELVGNKEIMIE
YANIGRSLAIFCAGFMYSGGFFYTAVMPLCTKRTEIIDNVTVRSQAFPIYRGLLDPRTSPSFEIVQ
LTQCLAGFVIYSVTVGACSLAAVFVMHACGQFRILGMKLDRLVNCSMGRADRKVTEQCLGDIVQHH
LRILGFITQVEELLNEICFVEFVGCTLNICFLGYYLLTEWEQSDPIGALTYCTLLISFTFNIFILC
YIGEILSEECKKIGLTAYMINWYRLPGKEALDLILIFAVSNSSRKLTAGKLVELSLGSFCSVLKSS
LAYLSLLRTLTT
>DnOr52
MQPKGQNRLNNYDHETDLDYTLEMCRWLLTPIGVWPLVKKCAGKHERAMSIVLLLVCFTCLLFVIL
PSGYNIFFVEKSMQNKVKLLGPVGFCLSSAIKYCYLVAKGTVFGRCIEHVQKDWQTVEQPCHRDIM
LKHASLSRQLIAICAIFLYTGGMSYHTVMQFLSKDRSKRNFTVRPLTYPCFDTFLDTQSSPTYEIV

FSVHCVAAMIMYSVTTAAYSLAATFVTHICGQIQIQMARLENLLGDSREKSSFHDRMTVIVRSHVQ
VLRFSKNVEKALRGICLTEIVESTLIMCLLEYYCMMEWRNSDAIAILTYLTLLISFTFNVLIFCYI
GEVLSEQCSKMGPASYNVEWYNLPAKEAHNLVLVSAISLYPPKLTAGKIIELSLNTFGTVVRTSVI
YLNLLRTVTTW
>DnOr52like_1PN
MRDQADKHSSYPTYKDDANYVLGYCRKLSRIIGLALLYDPVNKKEKIASIMYIVLCFAVLLFATIP
PFYYVIFDEKNTNKRLWALGXVGYNVTSTIKYLFMVYNANLFKNCLIRVDQDWQIVREPSHREIML
KXVTIYRKVSFYYLITFYAATGFNNTIIPFLAPRWHSNNTKKMLRVPGYDKFFDTQSSPTFEILYS
LQLFSSVIKITSTVVATILITTCVCHICSLMQIHIMQLNHFVENRQNLDDRLNPLTMIVHEHAETL
RRIGKVVQLSSFTTYPNIIQIGGTFLQFHHIFQMFHNECYISILLY
>DnOr53/54
MYARSCSRIDKNRPNNNDHETDLNYTLEMCRWLLKPIGMWPLVKKRACKLEKATSIVILFACFTCL
LFVILPSGYSAFIVEKSVQNKVKLLGPVGFCVSSAIKYCYLITKSTVFGRCIEHVQRDWQMVEQPC
HRDIMLKHASFSRQLIAICAIFLYTGGMSYHTVMQFLSKERNKQNFTVRLLAYPCFDTLLDTQSSP
TYEIVFSVHCVAAIILYTVTTAAYSLAATFVTHICGQIQIQMARLENLVADSRGKSSFQDRLTVIV
RSHGEVLRFSNNVEEALREICLTEIVESTLNMCMLEYYCLKEWENSDAIAILTYFTLLISFTFKVL
IFCYIGEVLSEQCSQMAPASYNVEWYNLPPKEAYNLVLVSAISLYPPKLTAGNIIELSLNTFAAVV
KTSVVVYLNLLRTVTTW
>DnOr53like_1
MPDRSQRTMIVGQTGNVNYENDVSYVLEVCRFMSKPLGIWSLIYNDTSRLERTISVVLLTAGFCGL
LFIAVPVTLHIIYEEMPLHVRIKKLGPDSYCILALIKYFYLALKGTAIGRCIEHVTNDWRTVKEPR
HRDIMLKQAGVSRNLSKLCIVFIYTAGLSYHTVLPVLAKRRIQSNQTTRVLAYGGYDRFFDVQSSP
NFEILYGLQCVAGFIKYNVTAAIFSLTVIFVTHICGQMQIQISRLGELTNKRREKDVVGSGSIGVI
VQDHVEILRLSKNVGEAFSEIILIEIMGSTFLLCLAEYCCLMEFQAASALAITTYVVYMVSFTFNV
FIFCYIGEILTEQCEQIGVASYEMDWYNLSPRIACNFMLLNVMSLYPPKLTGMNIIDLSLNTFSVV
MKSSVVYLNLLQTVV
>DnOr60
MESFPVQEPNCGEPRNPYYEKDILQILRYNKWILSSIGIWPIRSGDTNRYLPKLVIGLCNGILFFA
IVPCTLHITFELKDTVMRLKLLGLLTFCVTSFFKYWVLAACRSGLRECVECVQNDWKQVERGGDRE
SMLKYGNVGRNLTLLCLVFMYTGGMMYHTLVQYAIGSFVDEQNRTIRPVIYPAYSGLYDPQRTPIY
ELVYGLHFMCGYVIYSVAVGACGLAALFVTHVCGQINIIMSRLENLIDRKKNTDPGQRLIEIVEHH
IRTLRFSTKIERLLQEVCFVEFIGSTFLICLLEYYTITDWEFNNTLSLVTYFILLMSLTFNIYLLC
YIGELLVEKTSNVGLFCFTIDWYCLPTETVRGLVLIIAMSRNPAKITAGRVADLNLYTFVTVLKTS
LAYLSFLRTTVME
>DnOr60like_1
MESFPVQKQNNGIPRNPSYEKDILRILRHCRWILSSVGIWPILLKDTSLYLSKLAMGFWHVVVVVT
MVPCSLHMKYELNDSLRQLQLAGLMVFCMLSMSKYWALVARRLIFIECIERVQDDWKQVDCAGDRK
LMLKYGNMGRNLTLLCLVFMYTGGFMYHTVVQYMIGSYVDEQNRTIKPLVYPPYSGLYDSQRFPIY
QLSCVLQCMCGYVIYSVAISGCGLVAIFVTHVCGQIDIMLSRLENLVDHKENTDPGMRLKEIVKHH
IRTLRFSATIQTLLQEICFCEFAGSTFLICVLEYCTISGWDDSDPIALFTYTMLLISLTFNIFILC
YIGELLVEKTSDVGISCFMIDWYHLPTKTIRCLILIIAMSGNPAKITAGQIADLNLSAFGNVLKTS
LAYLSFLRTSVME
>DnOr62P
MEERLVFSERVLRLLGVWPLCGNDIMXTSLGRWCFAMLTQISTICSLSMEVYRHCLDMDDTMDAFV
MDLSSVICLSKVLILRYNWKHTYSLVNTIAEDWSTVEDSRHRRIMAEYREKGRFVSSTMLYLGYAS
GLSFVVKALPIHDLLPFQMLENSENSTGRVGQSPKKNYFMATYCVFGPQPFTRRAFVLVVQAVCIF
VNAIGHCGNDGFFFSLTMHLCGQFEVLKMNLGEIEIGKAGHRRRMGMLVKRHCRLILLADDLEKSF
NMVMLVQLLMSLLLLCVEDFMVLVYLNSMDNVAVLKCLVIIVTLLTQLYVYTYAGHVLESRTEEIS
YAAYDSPWYRSRGHAARDLALIINRGNSPYRITAGRFVSMNLVTFKEILKASVSYMSVLKVMMDT
>DnOr64
MKTPTNKDFDYAMTPIKVLSWPVGTWPLQTYNFVSGLRCAISIVLLLLMLLIVNTEMYLDHSDPEK
NLDALLFIACGILAIWKTLCFRVCCSGLVSNFTSALKDYNELLEQEKRTIVRQHAKMGRIACASVI

FFSYLDSTIFTTIPMLAGEDEVAFSKSNVTQEDSLNYPIPSEVTLKFLQVPESLYVVIYITEYLFL
LITSTGNLGGDSLFFGIMFHLCGQAEVLKVDFDRFVEGTENLSQRFNALVLRHQELLRLSEQLNET
ISLVMVIQLFLSCILICTTGFQCILSLSNNNIVMTVKTFSVVTTLLIQLFAYSYIGEYMKNQFDTV
GYRAYCSDWYNIPCDLSRDIIFVLMRSQNPVQLKAGSFFVVNMETYMGILKTSMSYLSVLRVMVTS
>DnOr68
MTVLQPAFFLLTICGCWRPSMWTMRYKRVLYTLYTILVISFLHTFCISQFLNVILNVETADELSDS
VYMFIANVLSCFKIITLLMNHNNIEMLCKKLNRKPCKPRNHEEAKIQRSFDNRIGSVTIYYTALVE
TTVLCMILSSIFTDFRHKKLAYNAWLPFNYSSRRLYYVVYGHQIIALIGTSLLNVACDVVICGLCV
HSCSQVEILKHRLEELPMQVRPQIGDIVHFHNYLYGYVDTIQQKFRVIIGVQLVSSTLVVCFILYE
LSNTPLMTAKYLQFLLYMACMMTQIFFYCWYGNGLKLKSVQVVDTISEMDWTRLDNSTKKSLIMIM
RRAMNPIELSSTYVFTMDLNTFVSILKMSYSTYNLLQRKKETNV
>DnOr69
MRILKFTFSVLTVCGCWRPESWTSQCKRLLYTVYTLIVLFLVYSITFYQFMDILVNVETQNEFADN
LYMFLAMMVSCQKIYGLLASRGNIAGLMNSFDEKPFLPENFEELGVRTKYDKKAHVNAFLYMILLL
GTVSCMSMTVFLTSEKWQLVFRLWLPYNYTTIHLYTLTYVQQSLALYLGALLHVACDSLICGMLIH
INSQCEILGCRLKALKNGEYEAVKSCVRYHNRMYRFARALNDEFKMIIFVQFAVSLLVVCFNLYRL
TQTTNPDAKLLEILLYSSCILTQIFIYCWYGNEVKMKSLEISDTVYDVNWAMLDNNEKRILLMIMR
RATVPIEFTSVHIASLKLESFVSILKASYSAFNVLQRGQR
>DnOr70like_1
MRTLRYVCTLLSVCGCLRPSSWTSPSKRILYRVYTAVVWLLIHTLAISQVLDLIVNVENQDDFSDN
FYVTLAALGICLKMCNVLMNQRNIGDMIDRLEKEPFSSVNKEEDEIRTRFDKVSNRNTLAYTMIIK
FYVLSMCLMSVFNDFRYNKLLYRAWLPYDYSTSFRFTVTYVHQIVAIIYCAFITVACDSLFSGVLI
HTYCHFEILGHRLKSIAKNDSESAKQCVYLHDHIYKYATMLNKQFKVIMFSQSLVSINTVCFNLYQ
LTQRDLGSKLFEVILYSFNTLMQIFYYCWYGNEVKLKSLEVPKMIFECDWACFDNDTRMTLLLIMR
RATIPVEFSSIHVVSMDLDTFTKVLKMSYSAYNLLQ
>DnOr71
MYILRRLNTFWMLCGCSRPTSWTSPTKKFLYTVYTIFLLLTIHSFLLTQILDLVLVVDNQDDFGDN
FYVTMSMLVTCCKLNSMLITRENILGLLNTLEGEPFLPVNDEELEIRTKCDRITEWNAMAYSISLL
SCVTWMFLMPVIMEYENRKLTFRVWLPFDYTSAGAYCLAFSHQLLAAIICCVSAIACDGLFSGLLV
HIYSQFETLGNRLRNIHRDENDSVKQCVRHHDEIYKFAGMVNQQFEGIMLIQFLTSTAAMCFDLYR
LTQKDFDLRVVDVLLFMFCTLIQIFYYCWYGNEVKLKSLEVPGMVFESDWLSLSNRSKKSLLMIMG
RASVPIEFTSIYFLSMDLESFKALLKISYSAFNLLQQTR
>DnOr71like_1
MYILRRLNTLRSLCGCSRPTSWTSPTKKFLYTVYTIVVLLTIHSLLLIQILDLVLVVDNQDDFGDN
FYVTLTVLTTCIKWNSMLMTRENILGLMNTLQGEPFSTVNDEELEIRTKFDRIAEWNAMAYTITLV
SCVTWMFVMSLIMEYENRKLTFRVWLPFDYTSAGAYCLAFSHQLLASMICCVSIVACNGLFSGLLV
HIYSQFETLRNRLRNIHRDENDSVKQCARHHNEIYKFAALVNQQFEGIMLVQFVTSTATLCFDLYR
LNRMNLDLNIVDVLMYTACTLLQIFYYSWYGNEVKLKSLEVPGIVFESDWMFLSNSSKKSLLIIMQ
RASVPIEFTCVHLLSMNLDSFKALLKTSYSAFNLLQQTR
>DnOr71like_2
MQMLQWTSGILQFLGCLRPPSWTSSSKKFLYNMYTLFVFTLMHSLLITVLLDIVFNIENQSDFSDN
VYATMSIMISCMKMCCFLASRQHIVVLLDTLEKEPFLPVNAEEMKIKRTFNRTNETISRSYTILLE
SCVTLVFIASLFRDAKNRKLTYRAWMPYDYSSLILYSVTFCHQVVSVVMCSLVTIGYDTLFSGLLF
CINCQLDILGHRLQNLSKDEKDSAKKCARYHNRIYEFAANVNKGFQTVVCVQFLTSSVIICFNLYR
ITQKDLQSTFFETVLYSFCILMQIFYYCWYGNEVKRKSLEIPKVIIESNWSALDDNGKKILLMVMR
RAMFPIEFTSIHVLSVNLESFMTLLKGSYSVYNLLQQRESSKFS
>DnOr71like_3
MHTLRLVSKILSIAGCWPPTSCSRRKKLLFDVYTVVVFLFIHTFTITLILDIFLIVDNLADFNDNF
CITVPLLSTCFKLYSLLANRRNMETLLDTLEQEPFAPVNIDECEIRRRFHRLTEWNTTVYMILVKF
CIMWMFVTSSTNYRLRKLKFRAWLPYDYSSTVLFNLTYFHQIVSALYAGLLGVSSETLYSGLLIHT
HGQLELLEYRLKNIEKNENYSVKRIKSVFIFLPRFAARVNRGFKTIVSTQFLFSTLVVCFQLYQFT

QMKRTMNSELLEMMIFLFTILIQVFYYCWYGNETKLKSLAIPDMIFGSNWTSFNNNTRKILLMIMR
RATVPIEITSVFVVSMNLQTFVTILKNAYSACNLLQKHQR
>DnOr71like_4
MGLSYLFTISTFCGIWRPLSWSSRFKKVLYNMYSVFVVCMNVTFTISQIISVLTVDNLDDFAENTY
MLVPAIVCCCKLINLLVYRGGIIELVKIFLEEPCATLNDKEELIQMRYDKQIRSRTNIIKYTRMVK
SSTVMILFTSFLNDFRNGRLTYRAWIPYNYSSTTAFGLTYIHQMMALAIAGYVHLACDVLLCGLLS
QICCQFDILQCRLNEVKKNETIALRECVRHHNRIYKLAAMVNDTLNLTIFAQFFGSFLALCLSLIK
LIKENTLSTQVISLLFFVVMIMTQSFLYCWYGNEVTLKSIAVADMIFQMNWIETNEQAKKILIITM
SRSRSPIQLETAHVMTCDLDFFVALLKSSYSMYNVLQSTRK
>DnOr73
MHKLPLSFAFLTYSGYWRPTRWSVHSLKYWLYNVYSVFMVLLLYTFTFCAFVDSVISKDLRTTTDK
FSVFISILGVCIKIANLFLQREKIIRVVDILLTENCVPRDEDEMMIQRKSDAYARRLTIYCEILNE
SAASFATVAQFDKLIRTRTLPISDWVPYDLSSRKVHMISLLHQTFGLMICANASVANETLIAGLMI
QVRAQFEIFCHRARSLPVSLLEVQRNSVSFAETVNAIFQYTIFLQFMISSTVLCLSIYTMSTADSF
DVNFVWTFSYLCCMLMQVYLYCWFGNEVTLKSMQVSDAIYEMDWTTLPVNIMKDLLMIIKRSKRPF
RMSSAHIITLSTDSFMAIMKITYSSYNILKDSSKK
>DnOr77N
MSWIKNKDISIAISALLMNCVGIWCADNPSEQRFRNATLMYTVCALIFAVWVQAQDFYHSWGDFGT
CAFIASNILCLNIALLKIFILSIRKKKFLDLVVYMEKHFWHSNYTPHEQTIYNSWKRLCTYFICFF
TFFTEASIVGYVIEPITANIGKNESNRTLPFNIWLDLPLSMTPYFEITFLFQVIINLMNIILTY
>DnOr80like_1
MQTKRHSDISIGSCAFFMKIIGLWTAENEREQLYRNIALVYTHVANVFGACVLFRDLYFCRSDSNL
YAVCNILNKLIVSIKLIVLTAHRGEFLDLLTYMQKHFWNGNYDHHEKTIIATSLKNCILFTTIVTA
ISHITLVCFLITPIIENKGKNESDRMLPFNMWLVNLPLSITPYYEMMFTFQGLLLYYTAICYFCFD
NVLCVMSQHVCGQFRVLQYRFTKLYDSEETIKNQNENYAAKSYVQFRKCVRQHQELLNYCWRLENV
FPVIVLGQVVIFSVLICLYGYQAFLAQSTIARRIIYIWFLVGSASLLFMFTYSCHELAVESENIGD
AVYSAPWTLVPMNKDGKMLRDNLKLTIFRSSKACCFTAYGFFPVYVETFTTVINTAVSYFTLLKQS
LKDSLEA
>DnOr80like_2PC
YKVCVFGFLYCVLLGGGKYPLTFELRLVCSLCILLIFRVXWLDVGLSFRGRFLVAYSLEGFLLYYT
AIYYFCFNNVLCVMSXHVCGQFRVLXYRFTKLYDSEETIKNQSENYAAKSYVQFRKCVXQHQKLLN
YCWRMENMFPVTVLGRVVIFSILICLYGYQAFLAQSTIVRRIMYMWFFLVGSASLLFMFMYSCQEL
VVEIENIGDAVYSAPWTLMPMDKGGKMLRNDLNFIIXRSGRVYCLSACGFFPVYFQIFTTVITENL
LNPVFVIHTIT
>DnOr84
MRLARSDDISIDWTSLLMKIVGLWMAADRAEQRRRNFALIYTITIIVIATSIAFRDIYFSWGNLND
CVFVSCNILYLMFVFFKIVVLYVHRIEFFALVRYTQKNFWHSNYDPREKVILADCKRVCAIFIVVI
SFCTQGTCAGYVMTPLFANVGKNHSDRILPFNMWVKFPVGISPYYEISFLIQTLCVYHVAVGHICF
DYFLSIVNLHVTCQFRILQYRLLSLGNVIETQTDEESLSRYTNVCLAKLRGCIQQHQALTEYCRRL
ENIFTLIILAQVLFLSIVLCLISYQLFLVNVPHFRNISLVMNMIGILCMLFMFTYSCDGLIRQSTN
VGRVTFLAPWSSMSMNKAGKTLRQNILITIMRSNQSCRLTASGYFPISLETYTGVLSTAMSYFTLL
RHKSLNVEVH
>DnOr84like_1C
DDVFVSCNILYLMFVFFKIVVLYVHRIEFFALVRYTQKNFWHSNYDPREKVILADCKRVCAIFIVV
ISFCTQGTCAGYVMTPLLLANVGKNHLDRVLPFNMWVKFPVGISPYYEISFLIQRLCMYHVAVGHI
CFDNFLGIVNLHVTCQFRILQYRLLSLGNVIETQTDETNVYLAKLKGCIQQHQALTEYCRRLENIF
TLIILAQVLMSIVLCLISYQLFLADVPQFRNISFVMNMIGTLCMLFMFTYSCDGLLGQSTNVGRVI
FLALWSSMSMNKAGKILRQNILITIMRSNQSCRLTASGYFPISLETYTGKLRDGSFLPLLQSTCYP
RTVGFFLPKSCHGGSELTM
>DnOr86like_1N

LCIPLSCVLMKPTGCWLANNNGERRRRRFALTYTFALLFFSIYINGTDIYHTWGDFSNDTFTACNF
MRTIMAIAKTVLLCRRKTEFCELVLHMQRKFWHSNYDSREQMFLKDARNFLTLCVVFVCFFTKSTL
VNYVTTSIVGKLNTEVSDTV
>DnOr87
MRTEQSKDYSIIMISFLTKLVGFWPATNPVEKRWRQFAFIYTICLLFFGAYLEMVDMYYAYGDFND
FIYLTCNLVTIILVLYKILVLYVNKKQFAELLLYSEKNFWHAKYDSYEKLALAKCRRTCTRLICIF
SFFAQGTAVSYTIGPIITYIEKNESDRTLPLRLHSNFPLYETPYFETIFTIQVLILYQYGVCYLFV
DNCMCVINLHVATQFRILQYRLKNMYTVSTKKKQGNTSETTSSIYADKCYATFKRYIKQHQALIAY
CNKMENVFTRIVLGQVLLFSLLICLDGFLVLLDEAPRTRRLIFAFHISGCLVQLLMFTYSCDCLMQ
ESTKIASAMYSAPWTHLPMNKSGRMLRNDMRLMILRARNPCCLTASGFLVVSLETYTKVLSTAVSY
FTLLRQY
>DnOr87like_1
MGKLLNLRKINASNHSESPVVSEQSRDISIKMSLFFMKLTGFWTSSSIEEDRLRTLAFVYTHCAIL
YGMFVQLRGLYYTFGDVSEFLYITVITLSITLVLLKTVLLSIHKKGFLDLIVYMDSNFWRANYDSH
EKQIIRDCNKVCSFFILFFTFFANGTVVCFTLQPIVENMGRNKSDRKLPFEMYFTEARWLSVSPYF
EMVFVAEIATLFHIALGYFCFDNFLCIMNIHVTGQFRILQYRLSKLYEEKMLQTDDHTPDKILLQY
TTKCYAALQSCIRQHQALITYCNVLEVVFSKIVLGQVLTFSALICLQGYQLLLSDAHLSRRLLFMC
FLTTTMCQLLMFTYSCDGLVQESSGIGTAAYISPWPLLPMSRQGHKLRDDLRMVVIRSRTPCCLTA
SGFFPISLETYTTVLSTAMSYFTLLRQSTIS
>DnOr90
MMTPVRDQEDKLGRPFTTQAKRHLDISIRSCAFFMKIVGLWTAENEREQLYRNLAIVHTLVSNFCG
YVILFRDLYFSWSDSNLAMYVFCNILTMLLVSVKLFALMAHRGEFLDLLTYMQKHFWNGNYDHHEE
KIIATSRKNCILFTSIVTIVGHATMVSYLLTPIIENIGKNESERLLPFNLWVQNLPLSMFPYYEII
FAIQGILLYYTAIAYFCFDNFLCIMSQHVCGQFRILQYRFTKLYASEETMTNLNENYAAKSYVQFK
KCVRQHQVLLNYCWKMENVFPLVVLGQVVIFSVLICLYGYQVLLAQSTFARRFIFISFLIGSGSLL
FMFTHSCHKLTEESANIGDAVYSASWTIMPMDKDGKMLRNDLNFTILRSRKACCLTACGFFPVQLQ
TFTTIVSTAVSYFTLLKQGLEDAVETQ
>DnOr90like_1
MRTERYSDISTRLCAFFMKIVGLWTAENKREKLVRDLTLIYTFVTHVLATTILYRDLYFSWSDYNV
SILQMALYVVSNCLSLNIVTVKLFTLTARRREFLELLAYMQKHFWHEKYDNHEKQILAATKKKCIL
FTSIVAMFAHATILCYVITPIITERILPFNLWLDNLPLSISPYYEITFFIEIMLLYYNGICYFCFD
NFLCILCQHVCGQFCILQYRFTKLYEADKETKNRNKIINSANFTMKSYTELKKCIQQHQLLLYYCN
KMKNLFPQVVLGQVIIFSALICLYGYQAFLARSTATRRFNFIFIMSEGSYLLFMFTYSCHRLIVES
ENIADAAYLAPWTIMPMDKYGRRFRSDLHLTILRSKRACCLTACGFFPVNMKTFTTVMSTAVSYFT
LLKQRLENTLEA
>DnOr95
MFATQSDDTSIAITVFFMKLAGYWTAVGRAEKCCRFCALVYTIASLIWGIYVHTFDFYYTRNDFDA
SLYAICNLLSVLMCVLKLFVMLSHKKDFFRLFLCLERKFLNSNYDSYETMVMGTCKRSCTAFIGFL
TFVTLATICSYTVSPLITNIGRNESDRVLPFNLWVGVPISMTPYYEIAYVIQILTAYPTGVCYFCF
DNFLCIMNMHVATQFRILQYRLANMKTSNVEEYPRGEFRSCISCSAEMYYDKFKGYVQQHQNLIAY
CDKLEEVFNLIALGQVLLFSMLICLDGYQILMANVGTERRLIFVFHLLTSMAQLLMFTYSCDGLIR
ESLNVATAAYVSPWIYLPMNKYGKTMRNDLILVIMRSRSPCCLTGRGFFVVSLETYTSVLSTAVSY
FTLLQETHDV
>DnOr95like_1
MSWIKNKDISIAISALLMKCVGIWCANNPSVQRFRNATFIYTVCTLVVAVWIQSQDFYHSWGDFGT
CTYIVCNILCLNIAFFKIFFLSIQRKKFLDLVVYMEKHFWHSNYTTYEQTIYDSWRRLCTYFICIF
TFFTEASNISYVIKPIAANIGKNESDRILPFNIWLDLPLSMTPYFEITFLLQVLSLYHVGLCYICC
DNFLCIMNLHVAGQFRILQYRFRSLHTLQIKDKEHQIQNTGSSYIAEEYYNSFKTCIQQHQALTTF
CAGLESVFSQIVLAQVLTFSLLICLVGYQLVLTNSSSTSHVIFVNLLSSTMCQLFMFTYSCDCLIR
ESTDVCTEAYVAPWSELPIDRYGKMLRKDLQFVMMNEALSSRGSSLEARGFYVLHVHC
>DnOr95like_2

MLEKSDDFSLSFTIPIIKMIGFWIAPNRRLQILTNCNLIFSCTQVSFLFYGTMYDIFYHCPDFNYL
IYLLCNLVTVGLCLFKTVIMLTYKRKFFSLVLYLQRNFLKSKYDAYEWSLMVTCKHTCTFFICTFT
ILTYATTSSYVISPLIENIGKNESDRKLVINTWIETLTYSPYYEIMYIVEVLCIMKCGFLHYIYDN
FLCILNLHVANQFRILHHRLENMKSPYDEGLVQEAMTTEDMRKNESRIGAEYYATFKKYIRQHQIL
IAYCENLEEVYNIYALGQILVFSMILCLDAYQLAEAPLTRKFIFLFHLLGSMAQLVMFTYSCDGII
QESLNMATAAYASPYTNLPMNVFGKMIRTDLIIVIMRSGVPCYITACKFFPVSLETYTAVIVVSTG
VSYFTLLRQAGETSP
>DnOr95like_3C
ICFLLLNTFVQNLSITAFIQVLSLYHVGVCYICCDNFLCIMNLHVAGQFRILQYRFRNLHTLQLKD
EEHERPTTGSFYSAQKYYNSFKICIQQHQALTTFCAGLESVFSEIVLAQVLTFSMLICLVGYHLVL
AESSSTSHVIFVNLLSSTMCQLFMFTYSCDCLIRESTNVCSEAFASPWSELPIDRYGKMLRKDLQF
VIMRSKRSCCLTANRFFPVSLQTYTSILSTSMSYFTLLKQNSDDTTNT
>DnOr105like_1C
VATFFTHEYDRRLLSRTAVFILAMTAFLDKYALFYVHMEKVRTLFDRIEYDWSRLANMNEYNILKS
YTENGKNVQRGCIVCVIPILIVFQLYEYSPMILDFVLPLNESRSNELIFKAELFVVDVEEHHMSVH
LFATIVLTMGLGTLVLADGTVTMFILHSCAMFKITGYICLFCRYRIQCAINTENKRLACKRLKDAV
LFHRRSIEFADSVKEFCSTSYFIFLICGSCSITMNLYRVSRNLWNDKKELAEGVYFSIYQFFYLFV
SNYASQMLINHSSHIFPLTYDTEWYSLPLQAQRSIITILGRSMKPSILTLGKMYDPSCEGFAKIYN
MSMSYLMFLCSI
>DnOr105like_2C
YDTEWYSLPLQAQRSIITILGRSMKPSILTLGKMYDPSCEGFAKLLPLTVQFLAILYSL
>DnOr107like_1N
MDEQAIKAEYLGTNIFCGKLVGIWPEQRKSLKYLARTIVLILAIIVAVTQVCTYVTRDPVVAVLHS
AQIENP
>DnOr108like_1N
MINVVTTFLGLVMPLNTSCPHHFVVLIEYTMVQEKSLFLLYAHLLTIYVFGTSMVAATNSLIFLFI
HYRCAMFRI
>DnOr111like_2N
MRLLEEPQYYIMKFFMMYIGLWPYESRKLAMVKQMFAVGLIISIAIPQV
>DnOr112like_1N
MDFIGYRYYKTIVYFLNLTGLWPYNTSRFKAAHTIVTYIILLSSFVAQ
>DnOr113F_C
KFLGQWPNQDPMDRGRRIIFIVFIIIIEVITTVTIBBQLEYLQSQINSDYKERTSEKELEIMERYW
SQGKQYAKGYMYFLYTATAVYCGFVFETRLLDIISPRNETRPYKFIYPAYFALDEHKYYYLISVFL
SGYILLDIITFIGFDTMLIMYAQYICGLLAFIGNQLEYELYKCTKDGEPIEHQRRAYKSIIEDSVR
THRKTLQLFVNRLDSAFMLPQLLQLGLNMIFLSISLVKVSDNQRGTFETFRHSIYIMGQLIHLLYI
CYQSERLIDHSLAISQNAYNGQWYNIPTETQKMLMLISRRSSIPSYVTAGKIFIFSMQSFSMVIQN
SMSYFMALTSLQ
>DnOr114
MDVKKELTYVYGWNHYTMRFIGIWPDERKWNQASSYLVLTPILMMLCFVCIPQTLNIPIIWKDLYL
VVENLSVANITITIALLKTIAFWFNGKPLKSLLRCMSRDWASDVKPSEREIMMGIARTTRTTIIRS
TLMVNAVVGFYCFLRYYSVNYSENKLLFRGYFPYDQTTSRNILMTAFGQLIATLYAAASYTAVDTF
VAMLVLHVCGQLSILKQDILSLRENGEEKMQIALGRIIRKHVYLNAFVETIEKCFNMMLLLQMLGC
TVQLCFQVFQAIMSLGGEEKWIAIFQILFLALYVVYVTLQLYLYCHVGEKLTVESADIANAAYNSE
WYNLSAKNAKLLVIVICRGQLPLQITARRFFSLTMVIYAEILKRSMGYISVLYAMKNK
>DnOr114like_1
MLITGVWPDPETRNGRSGGYRALISCSCIMSFIVMPQTLNLFRLDGDLELITQTLTLAQIPALNAV
IKIAFAWYRQTGKTMIKSFYDDWYTVRTEEERETMLNHAKVTKNISICCTILTQTMVTAYISLRLF
EILTSDWNEESQHRLVIYPGFFPFNVRPTLICILINMAQVFAAYCAREVDGRNERAKKSYNESMVV
LSSQDILSLSSRNKYNDEHFLFHRRMAASIEKSFNKLLLLQMLLCTVEICFQWFLFFSVIMKDENG
LLNFQMVFFVLFVVFITVHIYLYCYVGELLLVGGSSMAISAYNTSWYNVTPAEARCLLFIMLRSTR
PLCLTAGKFGTFSMELFSSVIRTAMGYLSVLLTVTGSDN

>DnOr115
MGWSQFNLTLLGVWPEPRKVSNISRAISSLVFWSTTFVTFMFICVPQTANLILKSTSQDEVIENLS
INIPIFFALAKQIVLWYYKNDLALLLRQILDDWSEPISGPDRKMMLKTAKLSRMISIFCSTLAYIM
LFAFISLQIWSNMENSSEADLGGLLHPATFPYDTKKSPNFEITWFGQFIGTTLMAICYSCVDTFFA
VLVLHLCGQLTVLRAALENLANATKENDFAKFQERLAFIVCRHNQLSRFAVIVEDCFNLTLLVQIL
IGTAMLCLTGYRMLSSVGEEDADLPVVGLIFYILHVICTMLHLFIYCYVGEALVTESTGLGQSTYD
CNWYDLPPKQAVSLVLIICRAKVSFQITAGKFSPFSLKLFSAVIKTSAGYLSVLRAMKT
>DnOr116
MDVFALERTKRRNITPNKHLNISLLMIYYMGMWPAQGKYRYLYFMYTICCFTFLLGIILTTEIAYL
IVNWGDIAKLAAGATLLMTNTCHACKVIIILCRHERIRCLIDVTRSKLFSRDNIKYERIVTYYTWQ
GIFHHIAYQSFGTMAVISWGVSPIIDLLSERSKQLPIEGWYPYNVTVSPVFEITSFYQAVAIFICC
FNNVAIDTLITGFITIACCQLTLLNRNISSLNPEAEKQSIVSKDDVDVKRSTIETYNRTYDNLKLC
IEHSNMIFDFSKEIQSIFGTAIFFQFLVNCIIICLIAFNMAQMKVYILHVLFGMLMYMCCMTYQIF
IFCWHGNELYLHSLDVVLASYSNDWWQRTKNFKRAIQIIMTRSQRPLILTAGNIMELSLQNFVRIL
RMSYSIFTVLQTSTD
>DnOr117
MKELSKSSIDHYILPNKIFCGMAGMWPIDENSPTYSKVFAYFRLLFALTAISSVFVPEVMAIAANW
GDLKVLAGVGCVLTTVGQLLFKMIYLLARRKKAYRLYYELRDLWITSNDPEERQSCEELAHWARLL
TLIFYISCMCNVVTFSVAAGFDYFKIKYNVSNAAEEIRHLPFDVWYGTDITGSPEFEIAFACQFVA
SMICAAGISGLDATFMTIILHVCGQFKLIKIWITKIGTEISCDSINGNSSRKLEVNLLRCIRHHQR
MLNVVNEVNNLLTPVIFVQLLTSGVEICLSGFAVLDNTTGTDLLKFISYLVSMGVQLLLWCWPGEI
LVEESQQIGHVIYLNVPWYVLPPIYRRELCLIIVRAQQQCSISALTFQTISIRTLTSVFNTAASYL
TLLRQMQQK
>DnOr118
MTVTSMINRPLELSIRLFGVWPNSSRWILKGVTWTTLITTFLVFQYRYCVTHFKSGNLIELLDGIS
ILLSNTLVLFKFVVIWIYRRTFHSILTTMLEDWQDHTSAAKTKQIMVNQATLSSRISNFLFVYYVL
CYMSYVIIFFRPFGTNHGSDFNSRKLLLKMEFPFEVTYSPLYEVILVLQFVMEMFIALAASTMTAL
ITALIIHVGSQVDVFCNKLQEISDSTGKHELPIVEIRDIVLRHQRILSLSRNIEVVFTYMSLMQFL
ANTMVICFMTFVLTLALHSAEGSDIIAKYVPYYIAINFEAFILCYTGEYLTSKGDSITQAVYNFSW
YELRPSDTRVTVFFIILRSQSQLKLTAGKFVTLSLTAFAGVRCFIVCFA
>DnOr119
MFSAMSIFAVTLFADIYSNIDNLSVATDDGCISAGIIVVIFKAIIYQVNREKIVRVIRDVLKCADD
LTEFSNVDRIRDIIKSHYTFNKVISHGFNILGCVLVIALLFFSPTENNLPIRAKYPFNTTISPWHE
IALGIEICAVSGGVLAILSMDSITVLLCNLIIMQFDILNVNFENCRRRVVNDVSKKVNFSSQTNRG
CDEQCYKDFERHVEKTESSVFLSRYKTCIRFYQRLVSVTKDYNKTYSSSLFIQMLSSTSMICLTGF
QVVVVGGQSSDVIKFGIYLSAAVSQIFYSCWIGNELIYSSSVLDRSQWLSDWHQEPLSDIVQVFTL
SMMSARQSTQIKAGGFYVMSLQTFITIIRRSYSIFTLLNNMQVTNS
>DnOr120
MNIENYTFINQLVLKFVGLYPINITRYIICINCMLLIVIPLVAHIYKNWENLNIILETSSVLLTIS
LALTKSLIWIFNRKELESFIDIMLTDYWKLVEPDVFTDLQKYAIYAKNVTKGYLFLIVNALLFFYS
LPVIDSFMSIIRDTNDNSTVPKNFPFVASYPLTFYKFPFYEIVYISQMLGTCICGLMIVATDTLIA
SALLHTCGHFKILKRNLRQLDSDIYYVSLMKFRFCSNMQKNFHSLLFVQTMASSIIICFIGLQVSM
TLMDQSNLMKYASHLMMALFQLLLFCFPGDMLISQSSSISRAVYSIQWYKLPLFIKYETSMIMLRS
QKPSYITAGKIYVMCLESFSSTLSTALSYFMMLRSFNLEA
>DnOr121
DYEWSVILIRYILDAVGLWPRQLSTEQKVLDKIQATVVLISVITIILIPLICALVLLLRDDLLAFL
ESFGFVMAIVTSLVKYVTFYANKEDLLLILKMMEDDWKKPKMNAEREVMIQHARIARAFVSLSYAL
VSWAAIALIFLPKLGMPLRHSTKETEMFLLPTYYIIDISRSPYVEIIYVLQLITALMVACCYVGVD
SFFGLLVLHISAQLENLQTRLANIKTSTHFDRVLKDTVMDHTRLIRAVDVIENTYMLLLLILLTNF
GIFSCLSIFEIVTVINGKDNYSKSILYLQLPSYINTFLQTCLYCIAGQLLVTQSEGVYEAAYDCEW
LNLKPKDAKNLILIMARSKKPLYVTAGKLFPMTMLTFCNLLKISLSYMSFLLTK
>DnOr121like_1

MNILGLWPQELNPEEKVLDKIRAITILLVTIIIVVIPSICTLLILSDDLPSRLEYFEFIIATAMAL
IKYGIVYKNKDFLPVLKMMENDWNEPKTDAERNVMIQYAGIARKFAKFCHGAIVFLTFTLLFLQKL
GMPVRHTTKETEMFIFSTYYVIDVSRRPYFQIILILQLISLFAQIYAYVGVDIFFGMLVLHISAQL
ENLRTRLLNIKTSNRFDRVLMDTVMRHTRLIRAVDVIENTYTLLLLMLLLYFGLFNCLCIFEIITI
INGKDNYSASVLYFQLGSYINTCVQTSLYCITGQFLATQSERVYEAVYDCEWLNLKPKDAKNLILI
MMRSRKPLYVTAGKLFPITMLTFCNVLKVSFSYMSFLLTKV
>DnOr121like_2
MNIAGLWPRELTGREKILDKIRAIAIVLVTIIIVAIPLTCTLLILSDDLPSRLENFEFIIAIVVAL
IKYVVLYKNKTDFLPVLQMIKDDWKEPKTDAERDVMIQRARIARSFIKFCYAAVFLITLTFIFLQT
LGMPLRHTTKETETFLFSTYYVIDVSRRPYFEIIYILQVISVLAIVYSYIGVDIFFAMLVLHISAQ
LENLRMRLANIKTSNCFDRVLKDTVMRHTRLSAVDVIENAYTLLLLILLFYFGVYNCLSIFEILTI
INGKADFPASVLYFQIGCYISVFIQTSFYSIVGQLLATQSELVYEAVYDCEWLNLKPKDAKNLILI
MMRSRKPLYVTAGKLFPITMLTFCNVLKISFSYMSFLLTKNLDTSGHA
>DnOr121like_3
MGTIGLWPQELSPTQKVLDKIRAAAIVLVTISIMIIPLICSLLILDDWHHDLPSCLENFEFLLAIL
TALIKYIFMYKNKQDFLPILKMMEDDWKKPKAAAERDVMIQRARIAKTLAKFCYVVVVLLTFTVIF
LQKLGMPLRHATNETEMFLFSTYYIVDVSRSPYFQIIYVLQAISLSAIVYAYLGADIFFGLLVLHI
SAQLENLQTRLSKIKTSNRFERVVKDAVMHHTRLIRVIDVIENTYTLLLFMLLLYFGVFSCLSISQ
IITIITGQSNYPASVLYFQIGCYATTFVQTSLYCIAGQLLVTQSEGIYAAAYECEWLNLKPNDAKN
LILIMTQSKKPLYVTAGKLFPMTMLTFCNVLKISFSYMSLLLRKL
>DnOr121like_4
MSIVGLWPRELGRRQKVFDKIGAVAFVLVMITVMVIPIICSLLLLSDWQHDLPSCLENFVVLMAIL
AGLIKYIAVYKNKDFLPILKMMEDDWRVAKTDAERDVMIQRARIGQTFAKFCFTAAALLTFTIIFL
QKLGIPLRHTTKETKMFVFSTYYIIDVSGSPYYEIIYVLQIISVFAIVYAYLGVDIFFGMLVLHIS
AQLENLQTRLADIKASNRFERVLKDIVMHHTRLIRAVNVIENTYTLLLFMLLLYFGAFNCFSITQI
IGIITAKGDYPISVLYFQAGCYINTVAQTSLYCIAGQLLATQSEGVYEAAYNCEWLKLKPKDARNL
ILIMIRSKKPLYVTAGKLFPMTMLTFCNVLKISFSYMSLLLRKL
>DnOr121like_5F
SAVDVIENAYTLLLLILLFYFGVYNCLSIFEILTIINGKADFPASVLYFQIGCYISVFIQTSFYSI
VGQLLATQVNTDFS
>DnOr122like_1N_C
MSHGKDFYGLSRKFLMIAGLWPFSGRTTKIVIBBRLSSPLVLDIVSPLNTSRSLHIFLIIEIHSKD
KYFFPIYAYVISSHIAGMVLITAVDCTSSIVVHYHLAMFETSYRMKNAINDWLDPENRTIEKDHQL
YRGICEAMEIYREISRYQIFSHRQLVEFFHSPGQADFSVLYGITTVMLFLYSYRENSISQTYIDSS
LNVFHETCSMRWYELPLKAQKLLILVLQRTSKGFVPVLAGIYVPSRSGYASMLNTSFSYLAVIYSL
RYGS
>DnOr130
MDVIAKECSTYNFMLRVGGLVPYANSFFTKIHRTVFLFILFSCLAIQVIRMLRITKQLLRNMFLAS
TFACPMILFICRYIGFVSTVPIVQVRLCFDSLQKDYYAIKNPVEMGILMKQIDTSKRIILAVLCTS
IAGLFYVYVVLSVPVMLDLLMPLNQSRPRMLHSFGFLVDGSTYIDLVALHIAATTTIGVVTLVCTE
STLVIVVCYICGLFKIANYRIRCAVDKAANLVADSKENTLIHLRICEAVEAHRKAMEFSQETCTSY
LITIVTAMISLALNLYRLFLAVISMDDFKEFIMSVAFVNVHLAIAFMCNIVGQLVINSSAELFYET
YNSDWYSIPVKMQKMLLLIMVRSSVDSMLDLSGLFVPCCEGFGIMMSTSFSYFTMLYSLSS
>DnOr130like_1
MDVFQRNYNMYYNVLCITGLWPFDDSLPSKIQRVVISVLTLWCIGVQLKGLTTVDFTIYNLLTMLS
YFCPMLLYFVRYVGFIYSFPSIRFMFKSIMADCVTLKNPIEVELLMKRMKATRTMFLIYLCKTFCI
LGFSCLAIALISILLLVPIFLQPKLQSRALRVMGFFFDEKSKQLNWICVHVTLTSLIGLLGIACTE
ASLAVFSFYLCGLFEIASYRIRTAINSGVKLATSNPIDVRPAVIMHRKAVESLISTITDLRELDNV
AMSILMVLVHLIIMYLNNYNGQQLINSSIELFDDVRNSLWYKAPLKTQKMLLFILMRSSTEVQFNL
AGLFIPSYTGFSTMISSSFSYFTVLYSVQ
>DnOr130like_2C

LQIRLIFESLQNDYYSLKNPVELEILMKQIDTSRRIILAVVCISSAGVFYVNTILSVPVMLDLLMP
LNESRPRMLHSFGFQVDGNIYVDLISLHVVSTCAVGMVIIVCTESTSVIFVCYICGLFKIANYRIR
SAVDGAANLISGSKESTLMHLRLCDAVEAHRKALDLVKRYGKDTCTSYLLAIIAVIISFALNLFRF
FLAVLAKEELKELLLCFVFVNVHVAIVFLCNIIGQLVINGSRELFSETYNSKWYYVPVRTQKVLLL
IMLRSSVDCMIDLAGLFVPCCEGFGMMMSSSFSYFTVLYSLT
>DnOr130like_3
MDVIAKECSTYYFMLRLAGLVPYANSFFTKIHRTVFLFILFSCLAIQIFIVIPHASLANLFVASTF
SCPIILFFCRYIGFIYTVPMVIAGVFYIYVVFSVPVMLDLLMPLNVSRPRMLHSFGFLLDGSTYVD
LVALHVAATCTIGVVTLVCTESTLVILVHYICGLYKIVNYRLRCAIDEAANLVAGSKESTLIHLRI
CEAVEAHRKAMEFVLLFCTSYLITIVTAMVSLALNLYRLFSAIISMDDYKELIMSVVFVSTHLAIA
FLCNMVGQLVINSSTELFYETYNSNWYCTPVTMQKMLLLIMLRSSTDCKIDLSGLFVPCCEGFGIM
MSTSFSYFTMLYSLSS
>DnOr130like_4
MDAFRKNYNFYYKVLRLTGLWPFSNSLPTKIQRVALCVIILWCMGIQLKGLTTVNLSIYNLLTMLS
YFFPMMLYIVRYIGFICNFSSIRFIFENIEADCCTLKNPIEVELLMKHIRTTRKVFLTYLGFSCLG
IFFMSILLLTPTLLVPKYQIRALRMVGFFFDKNSTQLNWICVHVTVVTAIGLLGIACTESYRIRTA
INNGVKLATSNPIDIRPTVIMHEKALRSANEISTVMVLPYLLAIIVVVVSFAISLCRLSSTITEMA
ELDNIVISIVIVLIHLIIMFLNNYSGQQMMNSSLELFDAVWNSLWYEAPMNTQKMVLYILMRTSTE
VQFSLAGLFIPSIGGFSTMISSSFSYFTVLYSVQ
>DnOr130like_5
MDAFRKNYNFYYKVLMDAFRKNYNFYYKVLRLTGLWPFSNSLPTKIQRVALCVIILWCMGIQLKGL
TTVNLSIYNLLTMLSYFFPMMLYIVRYIGFICNFSSIRSIFENIGADCCTLKNPIEVELLMKHIRT
TRKVFLTYLGFSCLGIFFMSILLLTPTLLVPKYQIRALRMVGFFFDKNSTQLNWICVHVTVVTAIG
LLGIACTEVALSVFSFYLCGLFDIASYRIRTAINNGVKLATSNPIDIRPTVFMHEKALRSLSSTIT
EMTEPDNIVMSIVIVLIHLIIMFLDNYGGQQMMNCSNELFDAVWNSLWYEAPMNTQKMVLYILMRC
STEVQINFAGLFIPSIGGFSTMISSSFSYFTVLYSVQ
>DnOr130like_6PN_PC
MESVNDHYCRISKVLLSTTGLWPYDVTWLTYVKRVLYSLVLLSSFVCQNGVYSQDVVVCCVTQLTF
NIIFLFKHHAFLYRIKNVSRNNLNVLBBIQTYYFTGDDEVPSHVMYLHVFTFLYLGITGLVGPESA
TIVLASHGCTLFNVIRYRIKNVFNQSPGASVFEKEKSVVDKLINAVKLHNETIRYCKQLNDSFSTS
YFILIMLGVMSLSLNLLYMSQSLQQHWQRKELMLQVMMIFTHLLYIFLANYIXQTVTDSSSKLFTE
LYNCRWYTASVPTQKLLLFIMHHSMKTYTFLLGGIFTPCYEGFSTVIRMSFSYFTVIYSAQL
>DnOr130like_7N_C
LKYRQSTLGKVLLSFIGLWPYEETWFAYAKRMFYSLVIFTSFVFQFAALFHTGVYTQNVIITFMTS
ATLYTIYTFRYYAFLYKIKNVRCFFLMECLIFFVRHYKDKIKTYQTYNIIFLSVNLLRVSTTIVTG
EYRLBBIISNIYSVCRPNLIVSACFCVLFKMQMSQSLKQRCEFNVLIVQMAIISIHLFYLFLCNYT
CQIITDSSNMLFMETYNCQWYIGPVRSQKLLLFIMHCSMKSYCFQLGGLFVPSYEGFSTVIRMSFS
YFMVMYSTQL
>DnOr132like_1C
RYIDLVQTVINIAYLVLINCGITGLAVALIRIRQSLIVTHDMSGVAFGISQFLLITLYVYGANICG
QELIDLSEEAFRKLANDRTLSIDTPIQIELLLQMKAKDNCQYSIGGGIIISSFDCLANV
>DnOr133like_1N
MRLLEKPQYCLIKFCLKYVGLWPYQNRKLAMVKQTFGVGLIISIAIPEMILVNTNTCSIRIVDSKL
TITSVLRVMVLFH
>DnOr133like_2N
MRLLEKPQYCLIKFCLKYVGLWPYQNRKLAMVKQTFGVGLIISIAIPEMILVNTNTCSIRIVDSKL
TITSVLRVMVLFH
>DnOr135like_1PF
NTLIHATLVEECVCQNDFMKFVSCNNFSGIDSPKIKXFRILGFSCLGIFFMSILLLTPTLLVPKYQ
IRALRMVGFFFDKNSTQLNWICVHVTVVTAIGLLGIACTEVALSVFSFYLCGLFDIARYVVSKLWL
AEITRLIPLH
>DnOr138C

MMLTLNKKITFFQFMSLAKTDMSWTNIVKTMTLAGPVTCTLLRYVSFIHNLPISLQIHVSINTLST
ISHTELMPNVFNRSFLTRSVKFFLILDVAGSMMITAAGIISSPIIFDIVTPLNVSRTRALTLIGFF
YNNSSHIDLVSAAVLLITGISFTMVLCTEAVVTVLYYRITGAVVEASKFTSTSKQYRTYYTNIQRA
VDIHLTAIQFVHFFEFHCSLLYRLDPVNSPIWTVSNYNLTLYNTLYNVDGVLIDLSIPTSAYTLVY
QRKYQTVNMIILVSSKKIYIYSSPWYFIPPEMQKSLLIIMQRSRIDCTYDLAGFFTPCYNGFATIM
STSFSYFTVIYSV
>DnOr140TRA
MRLLEEPKYYLMKLYMTYVGLWPYQNRKFAIAKQTLRIFFILSITIPEVAIIEAWGKDYDLTIQCI
PPVFTMCVMTAKYVNVIGKMEQFKTMFALIKSDWDRMTNQPEHQIFRKCAMEGRVCIGALTVLYVL
MVTVMMVHDNLRDRNETEPRQMLYEVEYFIDNEKYYYLLILHALILSLWTVMLLVAGDVIYIILIQ
HACVLFSITGYRLKTVHVLDVNTVCTSEDITEASKRNFSKREHEQIFQKFILCIREHNRALAFADH
LQDTFSVTLFIQMAMNIICVSATGVMILIRSNSTETLLRLALWLLGQLIHLFGICLPGQRLTNFSE
NLYYDALGCMWYICDLKTRILYQFFIMKTMTPQYLVAFKMMTLSMETFQSVVRVAASYFTFLSSSL
>DnOr140TRAlike_1PN
MDFFEQPDYYIVRFYMKYIGLWPYQSTRAATVKHTLVICILWSMVLPGIIAMVKEALTKNYVVFIM
CIPPLTCTTVAVIKYTSASIKTKKQIRTIFESIKSDWNKLMNLPEGQIMCKQAMEGRKFTIYLTFV
IITATVIYMLLSIVLITQDSKNMKNNSEPRVMLFNVEFFVDQQKHYYSFNIYFCLMSMNVVFVILT
IDTLYIVLAEHACVLLAVIGYRLKTVHVLNVNTVCTDEDIQTACDRVFSKEEHEQIYQKFKLCITE
HIRALDSTAVLVRVNSRGFFLNVSHYPVFTNPLXILLRSSSTEDMIRLVMWLTGQLTFLYSICVPG
QRFMNYSEKVHYCAIECMWYICNSKTQTIYRYFLMNTLTPRCLVACKLITLSMDTFLSV
>DnOr140TRAlike_2
MRLLEKPQYCLIKFCLKYVGLWPYQNRKLAMVKQTFGVGLIISIAIPETAMIEAWGKNYDISIQCI
PPVFTMCVAVAKYINVMVKMEKIKTMFESIKNDWDRITEQAEQQLLREYSMEGRKNTITFVSVCVG
AMASLYMLLTTATMVHDNLRDRNETEPRQMLYEVEYFIDSEKYYYLLILHAYILSLWTVILLVAGD
VIYIILIQHACVLYSITGYRLKTVHVLGVNTVCTGEDIMNASNRNFSKREHEQIFQKFKLCIREHN
RALEFADHLQDTFTATLFIQMATNIVCISATGVMILIRSNSTETVLRLAMWLFGQVIHLFGICLPG
QRLTNFSENLYYDALGCMWYICDLKTRILYQFFIMNTITPRYLVAFKLTTMSMDTFQSVIRVAASY
FTLLSSSL
>DnOr140TRAlike_3N_3C
MRLLEKPQYCLIKFCLKYVGLWPYQNRKLAMVKQTFGVGLIISIAIPEBBQIKTMFASIKNDWDRM
KEQPEQQLLREYAMEGRFVSVCIAVSGSMYMLLMTVTVVHDNLRDRNGTEPRQLLYELQYFIDQEK
YYYLLNLHAYILSLWTVILLVAGDVIYIILIQHACVLYSITGYRLKTVHVLGVNTVCTGEDIMNAS
NRNFSKREHEQIFQKFKLCIREHNRALEFVDHLQDTFSVTLFCQIAANVICISATGVMILIRSNST
ETALRLALWLSGQVIHLFGICLPGQRLTNFSENLYYDALGCMWYICDLKTRILYQFFIMNTITPRY
LVAFKLTTMSMDTFQSVIRVAASYFTLLSSSL
>DnOr142
MGLHVTPEKAIRFTKITVALAFTWPPSINSSNNRLLLFNVLWWAAFVSCLGLLLPLLSAIYVYSNE
PVLLGKTVSLFSAVAQVMIKMIVCRLMQKRFRRLFFDMENFCKYATEEDRIVVQRHMDSYKYSHVV
YAIWCFLTCLFVVSGPLYSPQMFPTLAIYPFPVEYQPMKSIIFFHQILVGCQATAGMAIDAQIALL
LRYATVRFEILAIQLRKAKSGQDLNACIREHVKLLWYTKSVYKSVRFIALATVATTHIAVIFGSLN
LVTKQPLLFKLLFALVVFSASVELIMYAWPADSLIHASNKTAAAAYETNWFEENVDIQRKIRFIIL
RSHRLEAIEISGIMPKITLSYYASFLYTAMTCFTALRIIVEKSNHD
>DnOr146
MFDQVSPEKVINFTRVCVALSFCWPLPTTATRSELRRFKFVRSILALNAIALMSALLYSLYGYYDD
PANFAQAACLMTAITHALMQICFGITQYEHLQWLIEEITAYCEEAKTNERQILQRYVDRYSMFYGI
SAIWFYLTAVMVVVGTLFISQPFPTKAEYPFPVDYEPVRSIIFFSQALAGFQCAAHISFNIFVAML
LFFAVARYEILMMELQNVTRFVDLTNCLRKYSHAKVYAQEVANCAWYVVATTVSVSTVALVVGGVN
FFGHQPLTVKLQYVCILGTGLLEVFMCALPADHLLDMSQKALHSVYEGRWYEQDVKLQKTVLYTLT
YRNPVAVRIKCIVPVLSLKFFGSFVSNAFSFCTALRVIMSDEDGEL
>DnOr146like_1
MFDQLSPEKVMKFTRICVALSFCWPLPTTATRSEHRRFKFVRSILALNATVHMLALLYSLYLNYGD
RANSAQTACLASTVGHVLMQICFGITQYEQLQWLIEEMIAYCEKAKTNERQVLQSYIDRYSMFYFL

VAIWFYMTAVMVFVGAYLLNQPFPTKAEYPFPVDHQPVKSIIFLMQTVIAFQCSAHVCVNVFVGML
LFFSVARYEILMLELQKVTRFVDLTNCLRKYSHAKRLLTRIVITYDSNGILELFKSEISPELPNNQ
PLSVKVQFVGILGTVLLEVFISALPADHLRDMSLKSLHSVYEGRWFEQDVKLQKTVLYTLTYQNPV
EIRINCIVPILSLNYFCSFVSNAFSFFTALRVIMSDEDGEL
>DnOr151N
IRQHFWTRWHKEYLNELTVRQKWHRHRDADIREGTLVILKEDNLPPLCWPLGRITSIHPGEDGIVR
VVTVKTV
>DnOr160
MTVGTKKPISLNVELFYDENVLSWSKRLLSLSGLWPDNGNDVRFFFYITYVMIFTWLEIVTLLQNM
HDLEKSLQNITLSFPTILIIILKAVMFRLNMHLLLPLLAVVKRDVEQGLYQSLEERRTVMWYNIAAT
LFSTSSALSLFFVPTLFYAKPIVSCLLSKFDNCSLPYELPMRVGSVYEVTEMQTYALFCVYLIPTS
LMLTIGATGADSLLVTLTFYLCSQLSILSQRIRNIDMEPKKYLPKMRALVERHTELLRLAATLAET
FSSLMFVQTLGLIFSLCIVVYQLLMTSESDGSDVNTIHFIIYSCAVVLLAFCYCFLGECLITESSE
IQFACYYTKWYDLPEEYVRSLTFCIARSQKPLYLTAGKFYVFSLETFGTIMKASMAYLSVMKSII
>DnOr161
MDFQLYGRREYDALIKPIMITGRIISIWPIAEKSGTATVLFRRFHQFCMFFLVTVMSIAVTADVIH
NLDDLDEATECALICTAFYLCVVRMVVYTIHQKDMLYVVNTMRDDWTTSTYEDRAILAGKTMFAFR
LAKYFISTVAVTIVLFMCVPLLEIYVAGKDKILPFRGYFYINQTVSPTFECLYLFNVSAGGFGGSM
IAGATSFNLIVIMHGSAKFAVLRKRLEAINSEDRDSATVITLYADALERIINVLALGQFVISTGLL
CFAGFQITSMMEDKGRLMKYSTFLNSAILELFMFSFSGDGLIEESEAVGESVYNGGWIGSRYGSSL
MIIMMRSTVPSKITAAKFYSMSLQSFSKVLSTSFSYFTVLTATKDE
>DnOr163
MASKTVITYPIEFCLLLVGVWPRSSYRIFQRIVWSVAMVFTLSSQFWYFAKKDLPDLLDGLSLVFS
NLVAFIKLIILWLNYGIFFNALTIVFEDWDNIALSGRNSRFMLEKALTSFHVSRSLIYIYSAAWIL
YSASAMMITDGTDEGSVSNQRKLLLKMKFPFEATVSPVYEVIVIAQFLLEYTAALIAAMLMALFAA
LVLHIGSQIDVMCQELMEIPNQRKEDRLRVFRSVVVKHQRIIYLSTNVKDLFTYISLVQFLANILV
ICFLGFNLVKSLGTENGSMLIAKMLPYYLATNVEAFILCYTGEYLASMSENISGTACNMDWYKLNP
PEIRLVKLLLLRAQKQLTLTAGKFVNHRNVCQCKMLNASASYISLLLAM
>DnOr165
MKSPSTVSLSVKYGLHFIGIWPGTPCPGLHKVLWVTCMMICQSYQYGYMIVHFKTDSLVTIIDCLS
IALPFTLVFIKLIVAWMNHSVLCDILSTMEEDCEKYARFDTNNFISKTADLSFRITTVIPSLYLIS
AGFYAAGTFAVQNANDSRELLHKMDLPFDSNESPAYELVVTAQFLHQAASAFTFGVFSSLLLMTIL
HVGCHVDVLCQILTTVSPDDNGQLRFVLSRHQEITIFTKKIEKLFTYIALSQLVSNTLITCCVGFL
IAISIQMENGLPLLIKSITFYFVICLEVFIYCFAGEYLDIKSNLLGSAAYNSLWYDSECSKSRQIV
LLLLRSRKGFPLTFGKFSTLNLESFTSIMKASASYMSVLLAM
>DnOr165like_1
MKLPSTVSLSVKYGLHFIGIWPGTPCPVLHKVLWITCMLICQSYQYGYIITHFRTDSLVTIIDCLG
IALPFTLVSIKLIVAWMNHNVLCDILSTMEEDCEKYARLDTNNFISKTADLSVRLTSVILCLYLMS
VGSYVAGTLAFQNTNVSRELLLKMDLPFDISESPAYELTVTAQFLHLTASAFTFGVFTALLLMAIL
HVGCHVDLLCHILTAVSSEDIGQLRFVVSRHQEIIVFTKKVEKLFTYIALSQLVSNTLITCCVGFI
IAISIHMENGIPLLIKSILFYFAICLEVFIYCFAGEYLDIKSDLLGKAAYNSLWYDSESSKSRLIV
PLLLRSQKGFPLTFGKFSTLNLESFTGIMKVSVSYMSVLLAM
>DnOr165like_2
MKSPSTISQSIVFGLHFAGIWPGTPYPGLHKVLWVTCMGICQTYQYKYVITRFRTESLIMLIDSFS
IALPLTMVCIKLIVAWTHRGVLRDILSTMEEDCQKYAVIDTNNVISRTAAISYRVTSTILILYVSS
VGFYAVGTFAFQNGNHSRELLLKMDLPFDINESPAYELVVTAQFLHQVASSITFGAFLGLLLIAVL
HVGCQIDIICQRLTDISIKTKGQLQFFITRHQEIIVFIDRVEKFFTYIALSQLVVNTIITCTVGFL
IVVSLKSENGVALLIKSVMFYIVICLEVFIYCFAGEYLNMKSKLIGDTVYESLWYDLQPSNCRQVV
FLILRSQKGLTLTFGKFSVLSLESFTGIMKASASYMSVLLAMS
>DnOr165like_3C
KTESLVTIIDCLGIALSFTLVTIKLIVAWMNHSVLCDILSTIEEDCEKYAGLDTNNFISKTADLSV
RLTSVILCLYPMSVGCYAASTLAVQNKNHSRELLHKMDLPFHVSETPVYELAVTAQFLHHVAAAIP

FCIFTAFLVMAILHVGCHVDILCHILTTASSEDNGQLRFVLSRHQEIIVFTKKVEKLFTYIALSQL
VTNTLVTCCVGFLVAISIHVENGLPLMIKSLVFYLVLTLEVFIYCFAGEYLDTKSDLLGKAAYNSL
WYDSESSKSRLIVPLLLRSQKGFPLTFGKFSTLNLESFTGIMKVSASYMSVLLAIS
>DnOr165like_4F
DSVLCDILSTIEEDCEKYAGLDTNNFISKTADLSVRLTS
>DnOr166like_1C
ADLSVRLTSVILCLYLMSVGFYAAGTFAVQNTNDSRELLHKMDLPFDVSETPVYELAVTAQFLHHI
ASAITFGIFTALLVMAILHVGCHVDVLCHILTTASSEDNGQLRFVVSRHQEIIVFTKKVEKLFTYI
ALSQLVSNTLITCCVGFLVAISIQMENGLPLMIKSIVFYFVISLEVFIYCFAGEYLDTKSDLLGNA
AYNSLWYDSESSKSRLIVLLLLRSQKGFPLTFGKFSTLNLESFTGIMKVSASYMSVLLAIS
>DnOr166like_2PF
LILVNRHGIIVICIGVLYCFVEEYLITKNKIISDAAHEPQXYDFQPSSSRQLVPXLISKSQKGLSV
TSVKFSALSLESFTDVRHLHFTHDNIYYSL
>DnOr166like_3C
CNIVIFLEVFIYCFAGEFLNTKSKTISDAAYKSQLYDVKPSSSRQPVLLMSTGQVKLPLIFGMFAV
LLV
>DnOr166like_4C
LLLISKSQKGLSVTSVKFSALSLESFTDVTKASASYMSVLLAMSPELFPSSVV
>DnOr167
MISVSRPVEIGLRVTGIWPSSYENVFRFVWILAMCTAQVLQYWYMMNHFNLDDLTDLVECVSTSLP
YSLVCFKLITFWTKRGILKHILLTMTADWENTSVTKHSLDAMIRKTQLSNWCSQLILSVYAIAVFL
YSSVYINVFRKAGRDDVIVDSSQLLMRMELPLASYERPAYQYVMIAQFVQLMFVATAIGTIDAFMI
TLILHVGGQVEMMHQALAAVCSKNKEQCLPKSTVKRLVNRHQKILNFTKYIENLFSYIALMQILCN
TISICCIGFLMSFDTDQNLRNVIKILFFYIAIVLEAFIFCFAGEYLSSKSTSINSAAYNSLWYLWK
PNESRTMLLFMIRSQKRSTITAGNVMELSLEGFATILKASASYVSVLYAMY
>DnOr169
MNTHFQKFNYLNVCVNVFSANMLPTTSEKIRTPGIMKFYVLFVWTIVLACLSATLYGMVHVPNDKM
LKDSTVYTMVSFESILLVVYLHNQKKSLRRLIETLNCLFDIDDKTIRIATTRLVTPFEKPMKIYVI
ASVGAVIVWTFLPLMEIFHRNQFYYEDYQIPMGVSREPFSTGIFIGGIAFQIVGCMYTTVRKASVD
IYTMHIILLMTAQYKYLNMKFTSALARYPVPGDEDIIRQELGGLVQHHKIVLRISNILKNVFTPNV
ALLYINNVFRFCFLTLMLVMNNEGPYVTMCVVSYTIGALIQLYMFCFCIQQLLESSTTMMDDVFHG
KWYLRDVSLQRTIMMMTMTDKLGCKLSRIRNINLTLPSFMSILNQAYSVCLLFLKSRQS
>DnOr169like_1
MNFQKLNNLNILGNIVSANMLPMTGEKTKMPTVLKLYVLVVWTFSMIYFITILYGLLKVPKDKVLK
GGAINMVVMFENLFLMLHFYNNKKVLRQLIRTLNSVLTIPDEILRTVTTKTVMILQKAMKTYIIFS
ISALIFWMPIPVMEAFHRNQFYYEDYQLPMAVSKEPFSTQIFVAGSVFQFFGAVYSLIRKLSLDLY
SMHIILLMTAQYKYLNKKFTSALASDPVPGNENQFRRELGGLIRHHRIVLRMTALLKQVFSLNVTL
LYVTNVFRLCFLSFMIATASEESLVACLVIIYTIGALMQLYMFCFCVQQLVETSTTMMDDVFHQKW
YLYDVPVQRSIALMILATKLDCKLSRYRTLDLTIPSFMSIINQAYSVCLLFLKTRQV
>DnOr171
MATLPLYQTYVSFVNRSLRICGLYPDTIHRSLSSRIIPPFWLLCYGIILYTVTTCLVQNVTDMSKV
TAGLSGIIAIVNAIVKAFRFVTYRGELEHVNGVLETVFEESSDRKPANSPAFRLMGSFYRLGYYQY
VCLLFTSFVFVVRPLLAVRFRNANYSLPVPGVYPWTINSTPVYVIHYVIESHICWSLCVVTIGVDA
FFTLCTFRISSVFKLLALEFEELPKRPIDKENRELTLRTCIRRHSTLIHCYDVIQEIYGPIVLVVA
LGNALSMCSMMFEVFQSKEFTVGKIIATVIYLFGKLIQTFIYAWPGEVVATEEVYCNDWYEHIDDP
SAKFFLMILKQKPIVIRACSILEVSLDLFAKIINRTISYYFLLVTLDDLDDGS
>DnOr172
MFGSAEEEDFPGYQYFRINRLLLDSVGLWSTQNQTITKIKKVLILSILLSVFVLQLTAFITTDISM
VTFLQNFVNITVTLTITLKYLTFLLNMKETQVSDMYNEIRYDWNSLTNEDELRIIHDYYRMMKKLL
PLFVVIILPSGLVITFTGVKSPILDFLYPLNYSRPREMPVQAEYFLDQEKYYYLLCMHEYTVALLG
MTVVFSTETLAMLNLRHICSLLKIISYRLEHVFDPHIPGGSFVQGSCVVYKRIVSIVELQRRVIIY

INIVRTKFMPQYFILFIFGAVCITLAAYHLLKALLLNSGLNEVLASLILVFCSTCYTFTLNHFCQD
LIDHMSSISQQSYNIQWYNASGTEQKLLLFIMFNNSNSKVLTIGGIYIPSYEGFGKVITENYTYF
>DnOr172like_1P
MNFLESRYYRITRVVSMPLGLCPYKRCDATKLLHVLSASLLETXDLILFKFRLQILALTTQERNLN
TILRILSSITPPVVCLVRLFTYYPNIDKVNEIRLLLKYTTQGKFLTLFYTSVFMFIAYPFVSVVFV
REVSSNPNRSSLPHLPILVEYFVDRNRYAYWILLHIDAQAFLGVSVLIATESATVTFIYHVCGVFQ
IVGDRIERTIEKSILNVLRGNKYSFVREEFRTAVELHQRLLELIEYLNKSFAKHYVLLVYIGVTTL
SINLSVLAVSLSLKKITDEFLLALIVICCNLFCLACANYFGQLIIDSSNDLFIKTYLTQWYESPLP
LQKSLLFLRQRCMKCSSYVADTLFVASMECFSSLTNTSMSYFMVLYSTM
>DnOr172like_2
MDFIGYRYYKTIVYFLNLTGLWPYNTSRFKAAHTIVTYIILLSSFVAQKFTYFNRLTVNVPAIRVY
CYAKLKEAMEQLKHHWNILRDSKEVELLERYARFGARCMIVYILHSYVIFSCMFVGIVFSTALTSD
KSITNVSRAFSINMDFFVDEEKYYYWIISYQYILCLMAGTIIACTEGLFLMMILHTQGLYNIASYR
LRKAIDSSSWKIPISEKQMLCHKGIANSMEIYRFIGMLVFLQCIIKNYLISIFITYTGFNDVFINC
ILLFEAFLLIFVTNSLMQRLIDEHIMFFATPYDTPWYDAPLPIQKLLLFLMQGQMKIISIKIYGHL
QPSMETTTMQMKQSFSYFMTLHSTRQL
>DnOr172like_3F
RTETFIGVVIPVIILPSGLVITFTGVKSPILDFLYPLNYSRPREMPVQAEYFLDQEKYYYLLCMHE
YTVALLGMTVVFSTETLAMLNLRHICSLLKIISYRLEHVFDPHIPGGSFVQGSCVVYKRIVSIVEL
QRRVIIFLQFLGPSFALVYSNNLSMHQRCDTRYLQHINHCEIHL
>DnOr172like_4PN
MDKLDFYYKPCRVHLLLVGLWPYGTSKLCKIQNFICFSVFCTAIAIQVERLNXFVFTNPLYLVTYL
QNSRIIFRRTYIGYLMCLVINWSFLLKYYSFWRYSTIVTFFQYIFVTTIVFESTINYRNLRFCNID
YTLPVAQSYLFRITFGGDI
>DnOr174
MFGSAEEENSPGSQYFRINRLLLYSVGLWPPQKQIITKPKNVVILLLLLSGSVFQLNAFVTIKFSI
KTCLQNIVTITLSLAFTLKYLAFLLNMKEVTTKTVIGDMIPVVVFPSALAVAFNGMKNQILDLVYP
LNYSRPREMPLPAVYFVDQEKYYYLLCTHEYVVMCLGVIVLLGTESTAMMVLRHICSLLKIASYRL
EHMFDPITPGASFAQRSLIIYRRIVSIVELHRRVIKYIHILRAKFSAPYFILLGCGTVCITLTFVQ
LLKALQLKSSLYELLASLLFVFSITCYMFIANHFAQDVLEHISYIYHQSYNSQWYNESTAEQKSLL
FIMLNSFNTNVLKFVTYIPSYEGFGIIMKTCFSYAMVIYSIE
>DnOr174like_1N_C
MILVKFYDNKIHKRLLLTLGLWPGSCPKIQRLRQLIVYFVILAGTLSQVFVVTEFLVIETGECGFK
GTVQIMSAVFPNLIAIVKYMNYHLSLGTVSEPFVAKKLLDVYANIDVIDHPVCVSFYKTMQTVHFN
DTLLSVLGIFWTFVLLILLVLSLHDLPANFVRIHVFSYLGEHIDRQTYTDTVAFSTFVIWIVDIYI
IIATEFSIFILLEHVNVCFKIVGBBRFVDIIWDTFGTTYWIIIPLGIAGMSANMMNVVQGIKSKEH
ILVITSAVHIITLMFYAFAGTFCGQRLIDYSVSIFYDAYNSRWYEIPVDIQKLYLFILQRSLKCCS
LSNKSIFVSSFEGLVVVKKENTSYYLIVRF
>DnOr180
MRRLRRNGLMNLLKVCCQLFGIWLPDSNASWWRVMFSRAMYWINFWNEFYGALAVLNGLCCCFTSV
SEVAKSFVEMVFMLEMLFNLIYYRWRSSEFQRLIIEMDKFFGTTNPRDRIVRQNYLNRTKTMYNLL
FGVFVVCALSFAFSPFITRNKVTPLNTAYLFLPREKYWGLRVTFALNIFHLMFTTPVILLDLLIIT
IIWHASCKFVEVGEALRTIKAKKLRGWIREHQAAISYADDVNHLMAPLAIKSTIAVTLSVIISGLV
IAHVSVSDFIIRNTGLPLFEMSKFFMLTAFSMLRFLMCSYAADVMIVEAQDIAWRVYDSPWLNATP
GVRKEAVIIIQRCQKPIAIHSAGFLTAWSLRFCGQVFYTIFTYFSTLRAILRV
>DnOr181PC
PLEYPQRTLSVIGVWPEQYRTTKGDIAYSITFCYFAIAITLIAYGAYKNSHVLDVLIIIVLELILT
GTNFIYMYILRASKDLERVIVMMKKEIKDGTIFENEEEKCVYLPYNRLSYYYSKYAITSAFCISYL
MYFVPLIELAVHFHAGNTTRTYEMPFPLYIMFDYEHNAWLYACTYIAEFFFTYVGIIHTSIVSFEV
DVVLHICGKLCIMCHRIRTIPAQPPELFEKSAKRMASSHVKLTKTHPVQFPEVVLQXSFEYKKKCM
CLNLYIFLILQALETNPVAATKFFMYTLQGLSLIFLYSYVGEIVLSENENVENALSDIDWPEVAIK
DRRTFLIFLVNAQQALYLTGGKFYKFSLYGFTDVIKSAMAFLSMLRATM

Supplementary Data S2-HlOr protein sequences

>HlOr1
MVEEIAKKSNYRNVNYKLDTEYAVRVAKALLTPIGIWPREDSLRDRVRLFAQTGVVFALMCFLLVP
HVIYTYFDCEDLTRYMKVIAAQVFSLLAIIKFWTMIVNRKEIRFCLLEMETQYRDVECEEDRQVMT
NCAKIGRFFTTLYLGLSYGGALPYHIILPLMSERIVKEDNSTQIPLPYLSNYVFFAIEDSPIYEIT
FVSQILISSIILSTNCGIYSLVATIIMHCCGLFEVTSRRMETISVPTGKDIRGRLRDVVQFHLKAI
KFAEMIEKALNIVFLSEMLGCTIIICFLEYGVIVDWADHKTLSMMTYFVLMTSIFVNVFIISFIGD
RLKQVSERVGETSYFMPWYDLPDDLAKNIRTIILRASHPSSLSGAKLFDLSLQAFCDVCKTSAAYF
NFLQAMTG
>HlOr2
MMKFKQQGLVADLMPNIKLMKASGHFLFNYYTDNATKGMHKIYCIVHLVLILIQFGFCGINLMLES
DDVDELTANTITMLFFTHSVVKLGYFAVRSKLFYRTFGIWNNPNSHPLFAESNARYHQIAVKKMRI
LLMAVMGTSVLTVISWTTITFIGDSVKKVVDPVTNETSFVEVPRLMVRSWYPYDPSHGMAHILTLV
FQLYWLIFTIADANLLDVLFCSWLLFACEQIQHLKNIMKPLMEFSATLDTVVPNSGELFKAGANMD
HGKDQDLPPPATPHPQTDNMLDMDLRGIYSNRTDYTTTFRATAGATFNGGVGPNGLTQKQEMLVRS
AIKYWVERHKHIVRLVTSVGDAYGVALLLHMLATTITLTLLAYQATKIHGVDTYAASVIGYLLYSL
GQVFMLCIFGNRLIEESSSVMEAAYSCHWYDGSEEAKTFVQIVCQQCQKAMSISGAKFFTVSLDLF
ASVLGAMVTYFMVLVQLK
>HlOr16_1
MKMSGIARAEEDLRYATRFVKPILGIVGAWPIPVNSSPFTRLVRRIEHVVTYFLFFLMIVPGLLYV
FLKEKNGKIRLKILGPIVNCSIQFFKYSVILYRIDKIQKSLNTIRRDWIEATDENRLIFRSKAKIG
RRVLLIAAATMYGGGLCYRTILPLLRGTIVTPDNVTIRPLPCPVYFVFFNEQRSPNYEIVFILQIM
AGFVTYAVISGTCGMCALFVLHACSMLRILVNKMNALVDKADMTETLVHRKIADIVEYQTKIKRFL
KNIETITEYICLTEMVGGTGLEWENNNTTAVIVYITLQISCTFCVFILCYIGQLLIDQNHVVGLTS
CTVNWYRLPNRHARSMILIIAMSNYPIKLTAGKMFEMSLASFTDVMKVAMGYFNILREVV
>HlOr16_2PF
FCIFILCCIGQLLIDENYVVGLTSCTVNWCRPPNRHVRYLTXLIIAMSNYLIRLASSKVVEINLAT
FTDV
>HlOr18
MRQIFLNRNYKNDLSFNVRLNVWTLKIIGSWPRSIDRSWLENSQHILLNVLSHCLLGFIVIPTGMF
AILVVEDFYDQLKICSALSFFMMAFTKYVIMTVREGDIRKCVEYVEADWRNVEHEEDRKIMLDNAS
FARKLIVICGAFMYGGVIFYYIALPLTRDKVVEEGGNLTYRRLVYPVPKFILDVRYSPVNEIFYTV
QLLSGFVAHNITVGACGLAALLAMHACGQLQILMSWMEKLVDGREDEEKNLDQRLANIVEQHVRVI
NFIALTENLLHEISLIEMVGCTLNMCFLGYYTMIEWDSKDPISSLTYIVLLTSVTFNIFIFCYIGE
VLAEQTVKVGEKSYMIDWYRMPGKKGLVVPLMISMSHSTTKITAGNLIVLSISSFGDVIKTSVTYL
NMLRTFTT
>HlOr20
MQKLATNKEQSPRTDYEYEKNINLSIQWNRWLLKPMGLWPNSRTTTRSRKILYKLINALCYGLISF
LFVPCSMYVFLEVEDLYDKLKLFGPLIFCVMAFVKYYSLIVHKADIRECVRRIERDWRNVRCEKDR
EAMIVNANFGRKLVLLCTFFMYSGFVFYYIAIPISVGRILSENGNLTFIPLVFPFTKYIIDTRYTP
TNEIVFFLQLIAGALMHGITSAACSLAAAFAVHACGQIEVLMNWLQHLIDGRADMSDTVDGRIASI
VSQHVRILKFLTLTERTLQQISFAEFLGCTMDICLVGYYIIVEWKSNDVTSAVTYIILLISLTFNI
FIFCYIGEIVAEQCRKIGEMSYMIEWYRLTGKKKLWSVLTIAMSNSSIKLTAGNMVELSISTFTDV
VKTAVTFLNVLRTLT
>HlOr22_1
MQKPVTDSLSSTRVEYDYKTNVKLSVQLSRWILKPIGVWPNSANISTVERYLYWLINVVCYSLITF
LLVPCSLYVILEVEDVYNRIKLLGPLSFCVMAFLKYHLLILYENDIRECIERIEWDWKNITYSKDK
DIMIENAIFGRRLIMICTFFMYSGFVFYYIAIPFSVGKIPAQDVNVTFIPMVFPFSRFIVDTRYSP
VNEILFSLQIMAGALMHSITSAACSLVAMFAVHACGQMQVLMSWLKHLINGRADMCNNVDRRIAKI
IPKMHPILQFLKVSEKALQQVSFVEFLGCMLNICLLGYYVIVVRKKITTSAVTYFILLMSLTFNIF

IFCYIGELVAEQVKKIGQVSYMIDWYRLTGNKKLCCILIIAMSNSSIKLTAGNMVELCLTTFGDVS
ITITVTIVKELFKT
>HlOr22_2PF
LPLKYNGQHSKVVCIQWNRWILKSMALWFDKSNISPVEKXLHXRLISAVCXGLMGYFLILCSLHVF
LDVENILKKIKLFVPLVLLCLATILKCHLSVLHDDDTYTCGEHIKWEWRYCVTHDEDKKIRDKRDL
LCLLHVQGAKKLMCTDFTFYFFVVSLGLEKMVAEDLSVTSFSGFIIDVRYSPVNEIFFSIRIVAVI
LIHGVTVGACCLTALFAVHDCGQMDVLMAWLEHLMDGRSDMSKNVNGRMVTIVSQHIRILK
>HlOr24like_1
MQKPGGKDEKLQVEDCTYEKRVNLSIPWNRWLLKPTGLWPDKSYVSPIEKNIARLVNTVCYGLIGY
LVIPCSLYVFLDVENIFKEIELFGIFAFCVAALWKYHSLILHGDDIRTCVEHMKYDWKNITHEEDK
EIMALNASFGKRIVTICAFFMYTGFTFYFLVVPISADKMVAEDLNVTFRQMVVPVSRFIVDVRYSP
VNEIFFLIQIIAGVLIHAIAVVACSLAAVFAVHACGQMGILMSWLEHLIDGREDMSKNVDDRIAVI
VNQHVRILKFLELVEKALRQVSFAELLVCTVNLCLVGYYIITEWNSNNLTTTVTYVVLYISLTFNI
FIFCYIGELVSEQCKKVGEVSYMIEWYRLPGNKKLCCILIMAMSNSSVKFTAGNMVELSISTFSSV
VRTSVAFLNALRTFS
>HlOr25
MKKQRSANLASLATENDYQEYLNLSIQLNRWLLKPLGIWPSSRKSSRIGRCFSWLMGFLCYALMSF
ELVSSGLYVVLEVKGAYNTLKMIGPMSFFAMSLLKYSMLTFHEDDILECVERIKWDWKNIRYLEDR
NVMVTYAYYGRKLITICTFFVFSSFVFFYIVVPINAGSVATEDGNHSYIQMPFPFPKIIADVRYSP
FNEIFFSVQFVTGIVLHGVTSAACSMAALLAVHACGQMEVLVSWLGHLIDGRADMSKTLDDRIASI
VTHHVRILNFLALIEKTMQQIAFVEFLGCTIILCLIEYYIITEWNPKELTFAVTYSALLLSFTFNI
FIFCYIGEIVAEHCRKVGEKSYMIEWYRLTGNKKLCCILIIAMSNCSTKFTAGNMVELSVNTFSDV
VKTSMGFLNMLRTLT
>HlOr26
MLDSSRDTLNATRDYEYSIQVNRWFLTPMGAWPRVTETSRAQQLLAKLLRLTCHSLMISSIVPCIL
FILFEDTSLEERMKSIGPMSHFLMGELNYCCLLAKANDILYCVEQVKQDWRNVKKPVHRDLMLKNA
KLGRSIACIAAFCMHTGVGSDEIVTGLRMSTFYVGNESYSMYPLPCTFYTKLMDTRFSPTNEIMFV
LQCLSGFIVNSVTVGACGLGAVLAMHACGQLNVVMSKLDDLVDAKGQEERVAQRKLGFIVEHHLRT
LSFIRNIENVMNLICLVELVGCTLNMCMLEYYLLTEKSKEKLASYVIVYVSMTFNIFIFCYIGETL
TEQCTKVGEKVYMTEWYRLPHKTALGLVMIISRSSMVIKITAGKFLQMSVATFGDVFKASFAYFNM
IRTVAM
>HlOr26like_1C
NIFILCYIGETTKVGGKVYMTEWYRLSQKTALGLVMIISRSSMVIKITAGKFVY
>HlOr26like_2C
NIFILCYIEETTKVGGKVYMTEWYRLSQKTALGFVMIISRSSMVIKITAGKFVY
>HlOr27
MTNEFAVVKTNFKSYSDYSLQLNRWFLKPIGAWPPSSSTTTLERNVSIVLNIVCYGSILFTLIPCL
LHVFLDDDSFYMKVKNFGPMSHWIISCANYTLLLLQGKDIRYCVEHIETDWRMVRREKDQQVMMKN
ARFGRYVATFCAAIMQSGVFCFFVSSALNTEIIHVGNETTIVRVLPVTVYKKLLNVDQSPTNEIVV
FMQTWSSIIATTSTVSIFSLAAVFATHAYGQLTVLMSWITEFVNESRNREKTFPFKQIGVIVEHHL
RVLSFISYIEEVMNRICFLELFRCTLAICMLGYYILAEWSTQDIQNLSSYFMILISLSFNIFVICY
IGEILTEQCKKVGDVVYMTDWYYLPDKRILDLILVIARSSVVVQITAGKFFHMSIYTFSDVIKTSF
AYLNLLRQVS
>HlOr27like_1
MSNDKTVVTEINITASSDYSLQWNRWAMKSIGTWPQFSSNLERNISRILNVICYTAILFSIIPCSL
HLMLEKGTFYTKVKILGSLSHWVFCIISYTMLLLRRKTIRHFFNHMETDWRTTTRKEKKEVMLKYA
KFSRYAAISCTIFIHGGILGYCVMTASTTMVVVVGNKTTILRVLPLPMYKGLLPVDTNTMNDIVVL
SQFLSGFIANNSAISVISLTSALTSHASGQLSVVMLSIEDFVSEARRRGKDDHFDEIPTIVEEHLR
VLNFISRIEAMMNKACFLELMRCMIAVCALGYYIFMEWAESDAKILITYFVALVAIVSNTFILCYI
SERLTEQCLKVGEVVYMTDWYYLPQRRILDLILIIKRSNVVVKVTAGKVLHMSLYTFGDVVKTGFA
YMNMLCQMS
>HlOr27like_2N

MSNDKTVVTEINITASSDYSLQWNRWAMKSIGAWPQSSSNLERIISRILNVICYTAVLFTIIPCSL
HVMLEKETFYTKVKILGPLSHWVFCIISYTMLLLRRKTIRHFFNHMETDWRTTMRKEKKEIMLKYA
KFSRYAAISCTIFIHGGILGYCVMTAFTTMVVVVGNETMILRVLPLPMYKGLLPVDTNTMNEIVLL
SQFLSGFIANTSAISVISLTSALTSHASGQLGVVMLSIEDFVSDARRRGKDDHFDEIPTIVEKHLR
VLNFISRIEAMINKACFLELTRCTIAICVLGYYIFMVDVILIVRA
>HlOr27like_3C
EEHLRVLNFISRLEAMMNKACFLELMRCTMAICAIGYYIFMEWTESDAQMLFTYIVTLVAVVSNTF
LICYISDRLTEQCLKVGEVVYMTDWYYLPQRRILDLILIITRSNVVVEITAGKIFHMSLYTFGDVV
KTGFAYLNMLCQMS
>HlOr27like_4C
EWTESDAQMLFTYIVTLVAVVSNTFLICYISDRLTEQCLKVGEVVYMTDWYYLPQRRILDLILIIT
RSNVVVEITAGKIFHMSLYTFGDVVKTGFAYLNMLCQMS
>HlOr28like_1P
MTNKSVFVETSFTSHSDYSLQFNRWFLKPIGAWPPSSSTTRLEKIISVILIFICYSTILVTLIPCV
LHMLLEKESLYMKTKVLGPLTHWFVGCINYTTLLLRGRDILSCVEHVENDWRIITREKDQNVMLRY
AKFGRYVAASCAAFVQGGVLCFCLVTVLSTEVIQVGNETRIVHLLPCAVYKELINVNDSPMNEIVL
VVQFVSGFIVNSSTVGIFSLAAVLTAHACGQLNVLMEWITEFVDESRNRKKKAPFKEIGVIVEHHL
RALTFISRIEDVMNRICFLELFRCTLDICLLGYYILMEWAEQDIQNLSTYFMMLIAICFNVFIICY
IGEILTEQCSKVGDVVYMTNWYYLPDKSILDLILIIARXNRREISSNVGLHVRRSSPLIFQVVKTA
FAYLNLLRQMT
>HlOr28like_2C
LDFCYAYLDDILVGYYIFMEWAESDAKILITYFIALVAIVSNTFILCYISDRLTEQCLKVGEVVYM
TDWYYLPQRRILDLILIIKRSNMVVEITAGKVLHMSLYTFGDVVKTGFAYLNMLCQMS
>HlOr31
MANESLVIEANLNSLSDYSLQLNRWFLKPIGAWPPLSLTSKLERIVSYALIVICYCSILFTVIPCL
FHLVLEDESIRTKVKVFGPLSHWFVGGINYTTLLLRSKEIRGCVEHMQTDWRIVTRPEDKQVMLKN
ARIGRYIAIFCAAFMQCGVLGYCVITAFTMQTVEVGNETRIVHLLPCAVYKKMIAVDTSPTNEIVL
VSQFVSGFIVNSSVVGAFSLAAVFAAHACGQLSVLMMWIKEFVNRSRDSNKNVCFDKIGVVVEHHL
RVLSFIARIESVMSEICFMELFKCTMDICVLSYYILTEWTDHDFQSLTTYFMILISMTFNIFIVCY
IGEILTERCKKIGEVVYMTNWYYLPGKDILDLILIISRCSVVIKITAGRIVPMSVYTFGDVMKSAF
AYLNMLRQTT
>HlOr31like_1
MTNNCAINVTANFDSLSDYSLQLNRWFLKPIGAWPLASPTTKFERILSHLLIFLCYCFILSTVIPS
IFHIILVDESLHLKLKLLGPLGHWFIGGINYTTLLLRSKEIRGCVEHMQTDWRIVTRPEDQQVMLK
NAKIGRYVAIFCAAFMQCGVLCYCVITAFTMQTVQVGNETRTVHMLPCKAYKKIVAVDTSPTNEIV
LASQFVSGFIVNSSAVGAFSLAAVFAAHAYGQLSVLMIWITEFVNHSRDQNKNVYFSEIGVVVEHH
LRVLSFIARIEDVMNRICFMELFKCTLDMCMLGYYILTEWSDSDVQTMTTHFMILISMCFNIFTVC
YIGEILTEQCKKVGEVVYMTNWYYLPDKDILNLLLIISRSSLVIKITAGKLIQMSVYTFGDVIKTT
FAYLNILRQTT
>HlOr31like_2
MTDDLESFGSLSDYSLQINRWLLKPIGAWPLSASISRMERIIWFLLIVVCYCLILSTVIPSILHIV
LEAENFHMKLQVLGPLGQWFVGMINYTWLLLHSKDIQGCVQHVQEDWCIVTRLEDQRIMLKNAKYG
RYVAASCAIFMQTSIMCKCLVTAFTTQVIEDGNETRILRMLPCPVYKEIIPVDTNPTNEIFLATQF
LSGFIVTATTVGAFGMTAVFAGHACGQLNVLMSWITEFVNQSRDENKNLYFTEIGVIVEHHLRVLS
FIDRIKNVMSTICFVELFKATLDICMLGYYILTEWESHDIQNLTTYFMILISMCGNIFLMCYIGEI
LTEQCKKVGEVVYMSNWYYLPYKDILDLILIILRSSVVFKFTAGKILHMSMYTFSDVIKSSFTYLN
LLRQTM
>HlOr31like_3PC
FLSICLSIILTLERHLRPRYISSIEYLKYVIYFVEVKTMKITRVTVRFIEWSDGDVQTMTTHFMIL
ISMCFNIFTVYYIGKILTEQCKKVGEVVYMTNXFYLPDKDILNLLLIISRSSLVIKITASKLIQMS
AYTFDDAIETTFAYLNILR
>HlOr34like_1F

SDYSLQWNRWAMKSIGTWPQFSSNLERNISRILNVICYTAILFSIIPCSLHLMLEKGTFYTKVKIL
GSLSHWVFCIISYTMLLLRRKTIHHFVNHMETDWRTTTRKEKKEVMLKYAKFSRYAAIFCAIFIHG
GILGYCVMTASTTMVV
>HlOr35
MALVTSSTDSTWDTDWMYSVQMNRWFLKPIGVWPLSLCVTTLEKISSVVLALISCFLIGFLLVPCA
LCTVLDKTGDLDTKIKMIGPLSFCMMAAIKYYILISRGGKIGQCVKDIRADWSLTLSHSQEEEREI
MRDSARIGRSLAIFCAGFMYSGGFFYTTVMPLCTVRTEIIDNETVRSQAFPIYRGLLDPRTSPSFE
IVQFMQCLAAFVIYSVTVGACSLAAVFVMHVCGQFRILVTKLDKLVDGVKGRKGLSTHEQRLGDII
EHHLKILGFISQIEGLLNEICFVEVIGCTLNICFLGYYLLTEWEQSETIGTITYCTLLVSFTFNVF
ILCYIGEILSEQCLKVGLSTYMIDWYRLPGKTAQGLILIFAVSNSSIKLTAGKIIDLSLSSFCSVL
KSAFAYLSLLRTLTT
>HlOr35like_1
MALVTSSTDSTWNTDWMYSVQMNRWFLKPIGVWPLSLCVTTLEKISSVVLALISCFLIGFLLVPCA
LCTVLDKTGDLDTKIKMIGPLSFCTMATIKYYILMSRGGKIGKCVNDIRTDWSLTLSYRQEEEREI
MRDSARIGRSLAIFSAGFMYSGGVFYTAVMPLFAVRTEIIDNETVRSQAFPIYRGLLDPRTSPSFE
IVQFMQCLAAFVLYSVTVGACSLIAVFVMHVCGQFRILVTKLDKLIDGVKGRKSLSTHEQRLGDII
EHHLKILGFISQIEGLLNEICFVEVIGCTLNICFLTYYLLTEWEQNDAIGTFTYLMFLISLTFNVF
ILCYIGEILSEQCLKVGVSTYMIDWYRLPGKKAQGLILIFAVSNSSIKLTAGKIIDLSLSSFCSVL
KTAFAYLSLLRTLTT
>HlOr49like_1F
YSFISRIEAMMNKACFLELTRCMIAVCALGYYIFM
>HlOr51
MRSFSSDRPSNDRYETDIRYTFELCHWILKPIGMYPFVFSRASRRERTASVLLILVCCSILQFIIV
PFSHHVLFSDNDMNTFVKNLGPLTFCLTTFFKYCYFSMKGSAIGRCVKHIERDWKMLRDEDHRVIM
LRYVTMSRNLIKLCAIFLYIGGLSYNTVIPLFSKKSVNGNVTIRPLTYPGYEEFFDVQKSPAYEIV
FCMHCVYYVMITANITMAAYSLTTIFATHTCGQIKIQTTRLEDLTKRGMLLEKGVSDRLAVVVSGHV
EILKFTRMIESALHEIFLIEVIVSTVLLCLIEYLLMMEWESSDSIGILTYVTLLTSFTFNILIFCY
VGELLLGQGSEIAHASYDTEWYNLPRRKARDIVLVLAITKHPIKLTAGKIFVLSLNTFGAVRESKL
VLIH
>HlOr52
MYDRSCSSVDGQLRNYHYQNDIHYTLQMCQWLLKPIGVWRLLDQRSSKLEHLVSIVLMTICFSSLF
FIVLPSGHYIFFAEKSIHVKVKVFGPVGFCLSSIVKYCYLCLNGGAFERCIQHVERDZZZZZZZH
RTIMLRYVTMSRNLTTVCAVFLYAGGMSYHTVMQFLSKDRSRENYTYKPLAYPGYDWFIDTQTSPT
YEIVFFFHCFAAMIMYNVTTAAYSLAAIFVTHICGQIQIQMARLEDLVERTGEKDDGPDPLVVIVR
DHVKVLRFAKNVEETLRDFCLVEIFESTLILCLLEYYCLVEWQNSDTIAMLTFFTLLTSFTFNIFI
FCYIGEILSEQCSQIGSASYEIDWYNLPAKRAYDLILLSVISQYPPKLTAGKIIDLSLNTFSSVVK
TSVVYLNLLRTVTNW
>HlOr53like_1P
MDDTNRNDVANSLKYCRRLLKPIGLWPLIYSHTSKLNKVLSIIILIAWCTLTVLFVLVPCGYFCVFH
VKNVKLKMKLLGPVLYSMLSMLRYSCLVLKTPAFKHCIEDIENDWKLIDNPEHRAIMIKNAMVTQR
MTVAFTIIIYSSAVSYHAIVPFFSNALENKKNNTIRALPIPGYELFVDAYSSPTYEIIRTHLFVYX
ELISLFTRSSSTKLVSYFVAHAYGMLQVQMARLKYLVADNRKESDRSRMLVVIVNGHSNALRYTRR
ILNALETLCFVEVLLSSILLCLCENLCVEGWKTHDMIFGVTIGGFMLSITFNIFVLCHAGELLVEE
AEKFGNAAYNIEWYNLPPNRALDLVLMIGIAKFPPRLTGGKIFDISINTFSAVLKSSVVYLNLYQT
MTEW
>HlOr60
MHLSMREQIDRKPRNLNYKKDIVYVTKHSKWILKSLGIWPAVLDETAGFLPKITIGLSNLVLLFTV
IPCILHIIFEQKDTIIRLKLFGLLSFCLISLMKYWALATRKPRIKECIEEVQSDWKEVESLRDREM
MLKYGQMGRNLTIICAIFMYTGGTIYHTVMQFATGTYVDEYNRTIKPLVYPTYSALFDVQTSPIYE
LVYFVHCMCGYVIYSITAGACGLAALFATHACGQIDIVMSRLGDLVNSKESSDLDKRLIEIVEHHL
RILRFSAIVQTVLQEVCFLEFIGSTLLICLLEYYCITDWESSNTVSLTTYTVLLISVTFNIFILCY

IGEILMEKSSSVGLSCFMISWYHLPTKTIHGLILIIAMSSNPAKISAGKVVDLSLSTFGNVLKTSL
AYLSFLRTTVI
>HlOr62
MKSTEENSGEKIAREFLVQETVLRIIGIWPSSVNNFASIGRWTFAILTHISTIYSLSLEVYRHCLD
LDDTMDAFVMDLSSVISLSKLLVLRMKSKHTYALVDSIVKDWTNVDHSRHERIMTEYFNKGRIVSL
TILYLGYASGLSFVLKALPLHLLPFQKLHSWANSTVNTNDTARLNYFLATHCVFGPLPLQHHVCVL
LLQGMHIFFNAVAHCGNDGLFFSLTMHLCGQFEILKMNLAEIEFEAIACRKRIGVLVKRHCKLAVL
TNDLEQSFSMIILVQLMMSALLICVEGFVFLVALSTKDNVAALRSFVLMVTLLIQLYLYAYAGNTL
ESRTEEIAQAAYDSLWYRCHGHAARDLLLIIHRGNSPYRITAGKFVPMNLFTFKEILKASGSYLSV
MKVMMDA
>HlOr68
MAILRPAFNILTICGCWRPCSCRTTRAKAAYIVYTMFVVLLLHSFCISQFFNVILNLRTADELSES
FYMFIASVLSCCKIVTLLTNHGSIEILRRRLEEEPCKPVSAEEAVIQSKFDRNIGSVTIYYTILVE
LTVLCMILSSLLGDFNERELAYRAWLPFDYSLPTYYYIAYVHQIVALIGTSLSNVACDVIICGLLV
HACGQQEILKHRMKEMTQEQRPNIGKIVRFHDYLYGYVFTMQEKFRWIIGVQLLSSTLVVCFILYE
LANTPPISSKYLQFVLYLACMMTQIFFYCWYGEQLKLKSVEIVRTIFEMDWIPLQNGIKKDLIVVA
RRAMIPIELTCAYMFTMDLNTFVSLLKMSYSTYNLLERRSDK
>HlOr69
MHLLQKQLQFCAMCGLWPPPSSAPLFKRFAYSVFSWYTLILVYITVFSQIMDLIVNVTTQDEFSDN
FYILLACAISLQKAQSVLNSRKNVHRMIDLLNSEPFHPECKEEIEIRSRISEHARSSTYYAILVES
TVMTLSFGALLKPESRPLPYRIWLPCNYTPLITYCSIYTLQCVALALSAMMHVACDSLICGLLLHT
YGQIEILGCRLKTIKENESKTSKLCVRYNNLIYRFTAMINEQFEMAIFTQFAVSTLAICFNLYLLT
GSDVTPIRYVEIIMYSSCMLTQIFIYCWYGNEVKLKSLEISNMIFHLDWTPFDKATKRNILTMMMR
ASSPIEIISVRVLSVNLDSFVALLKTSYSAYNVLQRGEG
>HlOr69like_1F
SLEISNMIFHLDWTPFDKATKRNILTMMMRASSPIEIISVRVLSVNLDSFVALLKTSYSAYNVLQ
>HlOr70like_1
MHTLQLSLKTLTICGYIRPTSWKSPGLKLLYNVHTVTVVVVLSTFFVAQILDIIFNVENQDEFSDN
FCITCCVMNSLGKLYSLLIGREMIQNLIDSLQSDPLRPLDEVENEIRLRHEKTIEKLSMAYTVCTV
LSATGVWMLSAMTAFRIRELPLRVWLPFDYRATLGIFSLTYAYQFVTLIFITTAIMGTDNLFSGLL
IHIYCQFEILQHRLKSIKPDREYSAKQCAFHHYRIYKFAEIINAKFSITMATQFLVTTMTMCFNLF
RLTQTKVYSQVLALIPFMVSVFAQIFYCCWYGNETKVKSLEICDTVFESNWTALSQSTKKTLLTIM
LRSLKPIEFTGAFVFPVNLESFKSLVKTSYSVFNVIQQT
>HlOr70like_2
MKVSVLFTFLSIGGCWRPISWSSTYKTAIYNVYTVLVLIMGVNFMVSQFGSMFTIETVQDFVDLTY
ICVSVFVGCCKLSNVVFYRKDIIDLMNIFMEDPCACSNKEEDQVQARYDDQLRKNAVRYTMVVEMS
VASTILNSLVTNFQHGRLTYRGWFPYDYTTAVLFPLTYALQLLCVLVYSWIHVSVDILFFGLLMQL
CCQFDILFSRFSSITSENKGVLRRCIRHHDRIYRLAEIMNDSLQLTMFAQFFGSFMVICLSLIQLL
NADILSTEFLATIFYLSSILLQSFLYCWYGNEVRTKSVDLADVMFHVDWTGLSEKGRKIILFAMTR
TRSPILFESVHVITVNIDFFVVLVKSSYSIYNVLKNT
>HlOr70like_3P
MDTLSWTFKLLTVNGILAPSAFMSQWQTILYGMYMFFMVSMLYAMECCLILGMIFNVDSQEDFSEN
LYITLILMCSCCKIYMLVTSAKGIKVMVDVLREEPFVPMNREENEIRVRFEEKIDWNAKAYTYFLD
ILVVVLWITSYFTDYRRRKFKFRVWLPYDTTTQPVFTLIYCQQIVCTWYSVNINVICDCLFSGLMI
QICSILRKMKPTRRNCVLVTIIRYTSQSRQEIKRYVXLAQIVNEKLGNVSSIQFAISTGAICFNLY
RMSVIELGPKFVEALTITVCLLAQIFYYCWYGNDRNYDSLEIVDRVTNSNITVSDESSKKILVLMI
RRALEPVEFTGLRIMSVNLESFTSLLKSSYAAFNMLQERREE
>HlOr71
MSVLKSTSLLLKISGCYLPHSWTTPFERSLYNIYGVFYLLLLSSLALSQILDVAINVESQDDFSDN
FSITAVVFLTWFKLSILLIRRGNFIFLIDTLQNKPFSPVDTEENEIRAKFEKITEWNTIGYWSLLL
ICAFWIYVRSLLTDFSSRKLMFRAWLPYDYSPAPIFILTFVHQAIAATICCTSSVTVDSLYTGLLI
NIYCQFEILEHRLRNVKTEQDDSVKQCAQHHDRIYQFSKKVNEEFKLILISQFCISMSVICFNLYR

MTQIKMDTKFVEIILYSFCTITQIFYYCWYGNEVKHKSLQLPYMIFHSDWTSLNNNVTRALLIMMR
RAVRPIEFTSIHVVSVNLESFMAVIKTSYSAFNMIQQSKQS
>HlOr71like_1
MSVLKSTSLLLKISGCYLPHSWTTPFERSLYNIYGVFSFLLLSSLVLSQILDVAINVESQDDFSDN
FSVTAVVFLTWFKLSILLIRRGNFIFLIDTLQNKPFSPVDTEENEIRAKFEKITEWNTIGYWSLLL
ISTFFIYVRSLLIDFSNRKLMFRAWIPYDYSPAPIFILTFVHQAIAATICCTSSVTVDSLYTGLLI
NIYCQFEILEHRLRNVKTEQDDSVKQCAQHHDRIYQFSKKVNEEFKLILISQFCISMSVICFNLYR
MTQIKMDTKFVEIILYSFCTITQIFYYCWYGNEVKDKSLQLPYMIFHSDWTSLNNNVTRALLIMMR
RAVRPIEFTSIHVVSVNLESFMAVIKTSYSAFNMIQQSKQS
>HlOr71like_2
MSVLKSTSLLLKISGCYLPHSWTTPFERSLYNIYGVFSFLLLSSLVLSQILDVAINVESQDDFSDN
FSVTAVVFLTWFKLSILLIRRGNFIFLIDTLQNKPFSPVDTEENEIRAKFEKITEWNTIGYWSLLL
ISTFFIYVRSLLIDFSNRKLMFRAWIPYDYSPAPIFILTSVHQAIASTICCTSTVTVDTLYTGLLI
NIYCQFEILEHRLRNVKTEQDDSVKQCAQHHDRIYQFSKKVNEEFKLILISQFCISMSVICFNLYR
MTQIKMDTKFVEIILYSFCTITQIFYYCWYGNEVKDKSLQLPYMIFHSDWTSLNNNVTRALLIMMR
RAVRPIEFTSIHVVSVNLESFMAVCIPNKPKRFLFTENSTF
>HlOr71like_3PC
CIWFSSIFTAFKIRELPLRGWLXPFDYRATLGIFSLTYAYQFVTLIFLTSAIIGSDNLFSGLLIHI
YCQFEILQHRLKSIKPDREYSAKQCAFHHYRIYKFAEIINAKFSITMATQFLVTTMTMCFNLFRLT
QTKVYSQVLALIPFMVSVFAQIFYCCWYGNETKVKSLEICDTVFESNWTALSQSTKKTLLTIMLRS
LKPIEFTGAFVFPVNLESFKSLVKTSYSVFNVIQQT
>HlOr71like_4F
DNLFSGLLIHIYCQFEILQHRLKSIKPDREYSAKQCAFHHYRIYKFAEIINAKFSITMATQFLVTT
MTMCFNLFRLTQTKVYSQVLALIPFMVSVFAQIFYCCWYGNETKVKSLEICDTVFESNWTALSQST
KKTLLTIMLRSLKPIEFTGAFVFPVNLESFKSLVKTSYSVFNVIQQT
>HlOr73
MHRLPLPFALLTCCGYWKPTKWPASSLKYWLYDVYSVFMVFLLYFFTFCACIDSLISKDLKTMTDK
FSLFISVFGVSLKVANLFLQRGRIINVMNILLTENCIPRDEQEKIIQRKNDNYARKLTIYCEILNE
SAAFLATVAQYNEFIRSKTLPISDWVPYDLSSQKLYTISLLHQTIGLMVCANTSVANETLIAGLMI
QAGAQFEIFCHRARNLTALLLDTRRNSESFTQTVNTVFQYMIFLQFSISSTVLCLSIYKMSTINPF
SMNFVWCASYLCCMLTQVYLYCWFGNEVTLKSEKVGEAIYEMDWTALPTDIRKDLLLIMARSKRPV
KMTSGHVVVLSAESFMSIMKITYSSYNLLTNSTNK
>HlOr76
MTSNNRDIAVTVTAFYLKIIGFWLADTYEEKRRRKFAQNFTIFMLVCAVVIETRDIYYTWGDFSIV
VYIMCNILTLGISLFKIFLSMWSKKKLLELIKFARTNFWHSDYDSQEQIIVNECKRTCTFLICVFN
FFANGTVSGYIIRPIVASIGKNESDRLLIVNFWIDLPITITPYYEILFTIQIFIIMYVCISYLCID
NFLCMINLHTATQFRILQYRLSNVCGANERSDKISKKTPSNSDECYAKFKNCIQQHQALIEYCNKL
QEVFGIFVLAQVLLFSLLMCLDGYLVMEDTPIMQRLTFLFHLTGCLCQLLMFTYSCDCLIRESLNV
ANAIYDCSWIHLPMDRSGRMLRKDLTFVIGRSRVPCCLTACGFFPVSLETYTSVISTAVSYFTLLR
Q
>HlOr76like_1
MTSKKNRDVSVSVTAFYLKIIGFWLADTYEEKRRRKFAQNCTILMLMCAVLLEVRDIYHIWGDFGA
VVYVMCNILTVGISLFKILVSMRSKEKFLELIKYARTNFWHSDYDSQEQMIVNECKRSCTFLICVF
NFFANGTVSGYIIRPIVESIGKNESDRILIVNFWIDFPTTMTPYYEILFTSQVFIVMYVCISYLCI
DNFLCMINLHTATQFRILQYRLSNVCGANERSDKISKKTPSNSDECYAKFKNCIQQHQALIEYCNE
LQEVFGLFVLAQVLLFSLLMCLDGYLVMEDTPIMQRLTFLFHLTGCLCQLLMFTYSCDCLIRESLN
VANAIYDCSWIHLPMDRSGRMLRKDLTFVIGRSRVPCCLTACGFFPVSLETYTSVIHSTFHTISML
EYFKYFFLYLINVFGKQ
>HlOr84
MHLVQSKDISIVWSFFLMKIVGLWLAADEAEQRRRNFALIYTLNAIFIASCIAMRDIYYSWGNVND
CIYVGCNILYLAIVFFKILVLYKHRIEFYGLIRFTQEKFWHFDYNSREKLILSECKKICTVFIVAF
SFCTQGTCAGYVVTPILANVGKNQSERMLPFNMWVDFPTGLSPYYEVLFIIQTLCVYHVGICYMCF

DNILSLLNLHVATQFRILQYRFMNLSNVIEKQTDGREFYLQLKSCVQYHQALTGYCKKLENIFSLL
VLGQVLFLAMVLCLVGYQLFLTDSPPSRNVGLVLNLAGTLCQLFMFTYSCDGLTRESMDVSRAVFA
RPWANLPMDRNGKSVRQSMLMVIMRSNRCCCLTASGFFPVSLETYTGVLSTAMSYFTLLRQSTMSI
AD
>HlOr84like_1F
YKHRIEFYGLIRFTQEKFWHFNYNSREKLILSECKKICTVFIVAFSFCTQGTCAGYVVTPILGN
>HlOr86
MQATKYRDISLKCGIFLLKLVGLWMSMNSVEERRRRFTLFGSVFALSYGVYVNVNDILHSFDDLSH
CIFLTCNTLCILLAIFKIFILSNHRTEFKEIVLYAQKHFWHDNYSKEERVLYGDCQRFCKLWVILI
CFLTQASLFYFVTPLTANMSRNKSDRVLPFKMWVDWPLSDTPYYELMFTFQALCVYQIGAAYICSD
TFFCMLNMHVICQFRILNYRLLNMWPVIDERTNMTEYTNKCYTKLIDCIKNHQSLIEFCVKLENVY
TLTILGHIVVFSLLMCFDTYEVVLAKVPTTTRLIFLFHMIGSFIHIIFFTYSCGGLIEESMDIATA
TYAGMWTVLPMNKVGKMIRGNMKLMTVRSLQPCCITAGGFFPVSLKTATALMSSTMSYFTLMRERS
AE
>HlOr87like_1
MVTDTTKYFSIRLTRILMKIIGFWNVQTRREQLILRVNYLYMLLVLSVSITIELLDLYYCRHDLYA
ATYTACNTIPLFVILMKTVVFTIRRKEILWLIEFAQKYFWHDKHSSFGKKAMDNLDKRALIYMSSF
TILIYATVVIYVLSPILENIGKSDEERILPFPFRVFNLPTSLTPYYEILFVIETLSVVHSGICFCC
FDNFLCILSMHVGGQFKILQNKLQTVLSEKSSKEKSAGLYKEFKECVQYHHLLLSYVEKLEYVFCV
PLMIQLLVSSIVFLYYLLFCSILFIRYLVNGVLMKRLLFLNYFLGGVIQIFLITLNCNDIMEQSGA
IGTAIYSCNWERNVYNHFYQFRKDMMIVMVRAKRPCYISAAKFFPISLESFTKVLSATASYYTLLR
TMEMDVIE
>HlOr87like_2P
MSNKEAHSPLAARVTAKLMWITGIWVASTEKEEKIFAILREILALNIFAMSWTIIYEIYVCDSSFY
SITQVASNAMPVFVVTIKTWSLLRNRQRILALIRFTETHFWNARLDKIDADEVKGIDKVNVCWIFV
LVYLNTFNISSNNVGRNASDKVLLYDTYLGLPKTESPFFQINFISEIFLCMYLLLCYIGFDASACV
LIIHAAAXFRILGRLLSYIFTEELYDKEISIQKQMSQKFEKCLRRHYLLIDYVEELNNLFKYTLLL
QMMTSSLLICMSGYECLVYQASLLKRMVYIMYMVASVSELWLIVRTVDELTTHSKAIGDAMYSSDW
GVIFGKLEKKLKFALTTMVIRSIETCSMKAGFFAVSSVTSTAFSYLTLIRTVEVNNVDG
>HlOr87like_3N_C
MTSKKNRDVSVSVTAFYLKIIGFWLADTYEEKRRRKFAQNCTILMLMCAVLLEVRDIYHIWGDFGV
SBBQVLSLYHVGVCYICFDNLLCLMNLHAATQFRILQYRLSNLGDTNRKQVDDNESSAALSRFAEN
CYKAFKNCVRQHQNHITYCHRLNEIFTVIVLGHILVFSLLICLVGFQVLVTPVTPARRLTFVFHIV
GSLCQLLLFTYSCDGLIEESTNIGTAIYLGPWIHLPMDKAGRRLRRDLTMVIVRSRKSSCLTASGF
FPVSLETCTTVLSTAMSYFTLLRHSFV
>HlOr87N
MRKISEEVKVGVMSARSKAVKKGSHTKDFALLMTSFLMKIVGLWLAKDSKEERKRRYTLIYTVVAI
LFGVWVQFRDFYYSWPNFGDCAYTACNILCLIMVLLKLFVVFIHRKEFIELLVYMYKNFWHLNYDC
NELLLLQDCRRISTICITLINFCAQGTIVSYVLTPIIANIGRNSSDRVLPFNMWVDLPLSVSPTYE
IVFVLQVLSLYHVGVCYICFDNLLCLMNLHAATQFRILQYRLSNLGDTNRKQVDDNESSAALSRFA
ENCYKAFKNCVRQHQNHITYCHRLNEIFTVIVLGHILVFSLLICLVGFQVLVEN
>HlOr90
MQTEKDSDISINLSKFFLRNVGLWRAENHAENRKRKALVAYTIWNMFLAIVVETRDMYFTWFYKGD
ILYVSTNLLSVILTTIKLTVVLIHKVEFMSLIDYMYEHFWNVDYDLHEKRIMDNCKKTCIIFTSSV
TTIGICAIISYIATPFIMHSQSNKSERAFLFDMWLNLPLTVSPYYEGMLAVQISGLYFTGICYFCF
DNIFCIMSVHLSGQFRILQYRLTKLSDVQYQISEKNMESVLEDCTRRSYEKFKSYVRQHQTLIDYY
EKLEHVYTKIILGQVLLFSVLICLFGYQILLANTSLARRSIFMFLICGAMVLLFMFTYSCNMVIEQ
SDNIAFAAYSALWTGMPMNKTGKKLRHDLIMVIGRSRRVCCLTANGFFPVSLETYTTILSTAASYF
TLLRNNVEDVVKA
>HlOr94
MSLKYRQDVSVRLANFYLYVVGFWFAANRIEEWFGNAAILYTIVTVFFVMWLQLRGLYFSWGNFEV
CMYMACNSLALVLDIIKIFVLLVRKKKFLGLIEYMQKNFWHLNYDQDENSIIAHAKRTCIYFVCVF

SFCSQSTVFSYIIRPFVSNLGKNESDREHIYDMWLDLPLSVTPYYEITYIIQALSVYQVSACYLCF
DIIFFILCLHVAGQFRILQYRIANVSGLSEIVKSSENPSTDELYSEKCYIKFKNCIQQHQALIEFC
ATLEEVFTVIVLGQVLMSSILICFIGYQVLLIKLSFESRVAFACFLMMGMSQLWMFTYSCDCITTE
SLNIAESVYAGPWINLPMDKFGKLLRKELQMLILRARRPSALTACGFFSISLETFTKIMSTSMSYF
TLLKQCIEDTVNS
>HlOr94like_1P
MSLKYRQDVSFRLANFFLYVVGFWFAANRIEEWFRNAAILYTIVTVFFVMWLQLRGLYFSWGNFEV
SLXYMACNSIALVLDIIKIFVLVVRKKKFLGLIEYMQKNFFHLNYDQDENSIIAHAKRTCIYFVCV
FSFCSQSTVFSYIIRPFVSNLGKNESDREHIYDMWLDLPLSVTPYYEITYIIQALSVYQVGVCYLC
FDNIFFIMCLHVAGQFRILQYRIANVSGLSEIVKSNDNTSTAELYSEKCYIKFKNCIQQHQALIEF
CEILEEVFTVIVLGQVLMFSIIICFAGYEVLLITLPFESRVAFTCFLITGVSQLWMFTYSCNCITT
ESLNIAESVYAGPWINLPMDKFGKLLRKDLQVLILRARRPSALTACGFFSITLETFTKIMSTSMSY
FTLLKQSAEDTVNS
>HlOr94like_2
MSLKYRQDVSIKLANFFLYVVGFWFAANRIEEWFRNAAILYTIVTVFFVMWLQLRGLYYAWGNLEV
CMYMAGNGLAMVLDIIKIFSLVVHKKKFLGLIEYMQKNFLHLNYDQYENSVIAQAKRMCIYFVCVL
SFFSQSSVLSYIVRPLVSNLGKNESDREHIYDMWLDLPLSITPYYEITYIIQALSVYQVSACYLCF
DIIFFILCLHVAGQFRILQYRIANVSGLSEIVKSSENPSTDELYSEKCYIKFKNCIQQHQALIEFC
ATLEEVFTVIALGQVLMFSILICFVVYQALLVSIVRYNKYSMMFATMTGVSQLWMFTYSCDCITTE
SLNIAESVYAGPWINLPMDKFGKLLRKDLQVLILRARRPSSLTACGFFSISLETFTKIMSTSMSYF
TLLKQSIDDTVYS
>HlOr94like_3N_C
MSLKYRQDVSVRLANFFLYVVGFWFAANRIEEWFRNAAILYTIVTVFFVMWLQLGGLYFAWGNFEV
CMYMAGNGLAMVLDIIKIFTLVVHKKKFLGLIEYMQKNFWHLNYDQYENSVLAQAKRMCIYFVCVL
SFFSQSSVLSYIIRPLVSNLGKNESDREHIFDMWFDLPLSVTPYYEITYIIQBBYNGTQNKLSFEW
RVALACFSMTGVSQLWMFTYSCDCITTESLNIAESVYAGPWINLPMDKFGKLLRKDLQVLILRARR
PSSLTACGFFSISLETFTKIMSTSMSYFTLLKQ
>HlOr94like_4F
KLSFEWRVALACFLMTGVSQLWMFTYSCDCLTTESLNIAESVYAGPWINLP
>HlOr94like_5PF
YDMYNGTXNELSFEWRVALACFSMTGVSQLWMFTYSCDCITTESLNIAESVYAGPWINLP
>HlOr94like_6F
LTQALSVYQVNVCYLCFDNIFFIMCLHVAGQFRILQYR
>HlOr95
MSSFTTDDISISLTSVFMKIVGLWMASNRFEQRVRNITLCYTLIAILFALWIQMTDLYYSWGDFGA
CLYTACNILSLTIPFLKILVLLAHKNDFFHLILHLQREFLHADYDDYEKKILLGCQRQCTFFVCFF
TLFTKGTVISYIVNPLIANIGKNESERILPFNMWVNLPLSMTPYYEITFSLQVLSLYHIGVSYFCF
DNFLCIMNLHAATQFRVLQYRMANMTDLKDKERKERNMKTSVSSACFATECYNVLKDYIRQHQNLI
AYCNKLEKVFNIIVLGQLLMFSMLICLDGFQILMANTPTRTRLIFIFHIGACMCQLLMFTYSCDCI
IRESTSTAEAVYSGPWTLLPMSADGKMIRKDLMLVIMRSSVPCCLTARGFFVVSLETYTGVLSTAA
SYFTLLKQRSENADSS
>HlOr95like_1
MKLFKTDDLGFSVTSMFVKMIGLWMAKNQFEQHVRNVTLFYTFFFVSFGFYVEATNLYHSWGDLSD
TLFDICTLLSVIMPAIKIIILFINKEEFFRLIVYTERQFMNGDYNEDERKIVFDCKRLCTFFTCFL
SSVTLSCVLSYVVTPIYMNIGRNESDRILPFKMWIDIPTSMTPYFEIFFTLQVLSAYQIGVSYGCF
DNVLCIMNLHLATQFRILQHRLVNINGLKSKQTPEKGETSIIDSTEFADNSYDVFKRCVRQHQSLM
AFCEKLEEMFSMIILGQLLVFSIFMCLDVCQVLLPGASVGKQMIFTFHLAGCMCQLLMFTYSCDCI
ITESLNVTEAVYDGPWPRLPMSTRGRMIRKDLILVIIRSRVPCCLTARGFFVISLETYTKVISTAV
SYFTLLRQRIEESNS
>HlOr104C
NEIFQIQHYQPLQKFLQFLGQDPSQRDGTRGSIVFVMVLSVLGILIPTFIEIYVQLCEKNMDAVIE
CLPNGIAAATSVVKLLNVYLNRENFQKMYDLVTREWEQLRMNDELHVLEDITMQGGKLAQFYRNTL

LSFMVVFLLVPLVFPFLDVVLPLNETRPRQQGFRVNYLYFDEADYFFSVYFQLAWGAVVVVMIIIT
VDSLYMIIIHHASGMFAVCGYQVKTATEYKDSIYGNDISENHTHEQFKRCVTTHDKAIEFYDILNE
SSRNSYLIQVGLNMMGISVTAVQTVLTLDRPAEALRCAVFLVAEQFHLFIISLPGQTLVDHCTKLS
KDIYSSTWYKVPAKFQRVIYTMQIRALKPCVLTAGGLYEMNMENFANTFKTCMSYFTMLMSLRE
>HlOr104like_1C
SAFQTNKRQNYEIFDIPDYQPLEKLLLFLGQHPYQTETTKSVIVLLMTFTLLSMAIPSLIEMYVQL
RKKNMDDMMESFANTIAAATSLVKLLNVYLNRENFRKMYNLVTIEWEQLRMNDEVHVLQEVTLKGS
KLAQLYRNALLFAMCVFLLVPLFGPALDIVLPSNETRPRQQLFRVNYIIFDQADYFFYVYIQLFWC
TIVLVMAIITVDSLYMIIIYHASGMFAICGYQVKKATEFVDSIDNSNISQNHTHEQFKRCVTMHDK
AIKFYDILNESSRNSYLLQVGLSMMSISVTAVQIMLALDRPSEAIRYSVVLGGAQFHLFIISLPGQ
TLVDHCTMLTNDIYSSTWYKVSAKFQRVIYMMQIRALKPCILTAGGLYEMNMENFANTFKTCMSYF
TMLMSFRE
>HlOr104like_2PC
SVFQKRERHEGSIFDFPYYGSFKNNMLFLGQYPYQSKLTEIFNETLMVCTYTSLIILQVIQMFEAI
IVKDIDATMETVPVIVTELVCVIKLLNHIINKGKFDKLYDIMKKEWDMLSLDEQQILEEITKRGSK
LTNIYRSTLFYFSIIFVLFPLIPPILDVVSPLNETRSRIEVIKLQYLFDMEKNFYSLYVYTGWCSF
MTMMVIITIDALYVVIIHHTCGLFAVCGSQVSDKYSYNIXSLKKKKKXRVQTFLRFVIFIHDFSSF
DTINEITSTCYLFQVGLNMIGISVTSVLAFINLDRPEEFARIACFLIAQQLHLLVISVPGQIITDQ
SSKLTIDIYNSMWYQTPVWIQKILHIVQIRSGKPCKLVAGGLYEMNIENFGSTFKTCISYFTMLLS
FK
>HlOr104like_3PC
KRSVLQKSGRAPREVCNIFYYRDIRKYTMFCGVDGNKINNTILIVTICSLMSMVVPAVREIYISVR
EKNRNATIEVLVSITATITAIIKLVNVYAHRQYFKNLCISVVEKWEELRISNELSVLEEVTAQGSK
FVQLYRIVLFSVLFIFLVVPIFPPLLDILKPLNETRARQKIFKVNYLIIDRDEQALITYFHLSWTT
ITLGISVLSSELIYVLLIHHTSGLFAVAGIDTIYKXIQSNDLIVGKNLEDEIYEKFKRCVDTHNEA
IECYNVLQKSSVLSYLFQVGLNIVGLSVSAVQTVGNIDDPTVALRNATVWGAKQIHLFIISIPGQV
LIDHCVELSNDIYSSKWYNNGMEAKKILQVMQQRASKPCILYRVCKINMKLCFNFKTFKTCISYCT
MLLSLRE
>HlOr105like_1
MYSVFQKKSKQAYNVFSMIYYKSMKSYMNILGINPYQDKRAGIALQSTAMFLCVSVMSPMFLKIYV
AQREKDFDGVMQVLPNIIAQTGALVKVMNIRINKANVCIYIVAKKEKGIFVCEKIVMIERISSFEN
YSISWYEDSLWIVLLVYMSFPLINPVLDVVAPLNETRPRRLLLPVSYIFIDEYEHFYSVNFHLYTS
VISCGMMIIAVDMLYITIIFHLCGLFAVCGHRIQVTTEMNIVNKDETKKSEAGYKQFKQCVTMHHE
ALQYCQILERSSQHMYLIQVGLSVFIISVTAAACIMNIDRPDELIRLAMFVVAQKFHLLILSLPGQ
MLLDHSLGLADIIYASKWYELPVKGQKLIYTMQMRSIRPCILTAGGVHEMNIESFGIAIKACMSYF
MMFLSLKE
>HlOr106like_2
MDEEEVINRYLKNVRYYGRILGIWPLDNSWKPRVVPKLLYIRCITSLRLQSARIVVAYSFTVLTEQ
LPYLCIALMMIVKHGNCFLQLSKLRQIVTNMISDWQKKMIDEELAIMNKYAKRGALFSFLYITIYI
GVSVFSTAPLIPFILDLISPLNESRPRLYAYKAYCYFNPDDYYYYVVGYTSACIGMAGLVLVGADI
IMIHLVLYRYRFEYAVATVENSTKEEIYKMRRASYMKLRAAFQSHNAAMEEATYTLNLVPQVFITI
VVFSVTLFQVIAQTVHMLFLTIQGQFVIDSHDTVYNVCMAIWYKMPPKLQLLDVVALRKSLTPPIL
TAGGLMRLDLNSFAQLLKACVSYYTALRPS
>HlOr108like_1F
ESLISGARNDWSIGRPQGEIDIMTKYAERGAFLISTYLVNAIVCVILFVSTPVTPHILDILLPKNE
SRDVAYIYPAYYFTDEHKYDVYIITHMASVILVVFYVYFACDTSYIYVVQHGCGLLAVSGYRYKCA
IVELSSRKRNSVTQDEIYRRVCHSIQGHQHAIKYIDQIAKIHAVYFFICVGVIMICFSVILVKVSN
SEINAEFTKDCTFIMIQLVHILYLTLQGQFVIDANEEIFESM
>HlOr111
MEASDVERNYLYVNKFCGTMAGIWPYGDEKMKWVSRFVILVITVTAMTTQVAYVKVNLSTDTVIEQ
CPFFTMGLGIFLKELNYIIHEQQLRKLLGCVFADWIVKRPKPEMDIMDKYAKRGLFFSRLYVANGI
FCYVLFTAVAITPRVLDLISPKNESRGVGFIYPANYGVDEEEHYYYLMGHMLSVITVVFFVYISCD

TIYMNIVQHACGLLNLSGTRSFMINYTEVDDTYRKVRHSIKAHQHAMEYIMQIEDVHDSYLFIIVA
LIMLSFSITLVRTSMMDPCMEFYKYCAFLVVQLVHLLFLSIQGHFVILSHDMTYDNIYSAKWYNSS
PRTQTLYVLALRRSLTPPLLTAGGLISLNLETFAEILKASVSFFTVMKST
>HlOr111like_1
MEADDVERNYLHVNKRCGTVSGIWPYGSSRSKWIGRITMIFVTVTGMMSQIAYVAVNLSIDNVIEQ
CPFFTIGVGLLIKEVNYILHEEQVNTLLSCIFDDWLIKRPKTEMDIMDKYAKRGRFFSRLYTVNGL
FCYVIFTSVAITPRVLDLISPKNESRGVDFIYPAYYGLDEEEHYYILMGHMLCVITVVFFVYISCD
TIYMSIVQHACGLLNVSGYARRHCFKWAVETVITKNEKMHTEVDDTYRKVRHSIKAHQHAMENRYI
MQIEDVHDSYLFIIVALIMLSFSITLVRTSMMDPCMEFYKYCAFLVVQLVHLLFLSIQGHFVILSH
DMTYDNIYSAKWYNSSPRTQTLYVLALRRSLTPPLLTAGGLISLNLETFAEV
>HlOr111like_2
MDAITLEEQYIKLTKICAQFSGFWPWQSKLSKYVSRIITYTIVFTVLFTQVAQVVCFFSLDVLIDQ
LSFFGAVSGTLLKQGNYIVFKEEYESLISGARNDWSIERPQGEIDIMTKYAERGAFLISTYLVNAI
VCVILFVSTPVTPHILDILLPKNESRDVAYIYPAYYFTDEHKYDVYIITHMASVILVVFYVYFACD
TSYIYVVQHGCGLLAVSGYRYKCAIVELSSRKRNSVTQDEIYRRVCHSIQGHQHAIKSVLKKIRQI
VLNVLCFFSSMLLLRPTIAWKQVSNSEINAEFAKDCTFIVIQLVHILYLTLQGQFVIDANEEIFES
IYSALWYNSDKRTQSLFVLALRNCLSAPQITAGGLITMNLQSFSEIVKTSVSYYMVLKSV
>HlOr111like_3
MDAITLEDRYIKLTKICAHFSGFWPWQSKAIFIRFLGKTDRRLILQVAQVVCFFSLDVLIDQLSFF
GAVSATLLKQGNYIVYKEEYESLISGARNDWSIERPQGEIDIMTKYAERGAFLISTYLVNAIVCVI
LFVTTPVTPHILDILLPKNESRDVAYIYPAYYFTDEHKYDVYIITHMASVILVVFYVYVACDTSYI
YVVQHGCGLLAVSGYRYKCAIVELSSRKRNSVTQDEIYRRVCHSIQGHQHAIKSVLKKIRQIVLNV
LCFLSSMLQLRSTISWKQVSNSEINAEFAKDCTFIVIQLVHILYLTLQGQFVIDANEEIFESIYSA
LWYNSDKRTQSLFVLALRNCLSAPQITAGGLITMNLQSFSEIVKTSVSYYMVLKSV
>HlOr111like_4N_C
MDEEEVINRYLKNVRYYGRILGIWPLDNSCVVLIFQVLNWSRSNTAIVVQLLQSARIVVAYSFTVL
TEQLPYLCIALMMIVKHGNCFLQLSKLRQIVTNMISDWQKKMIDEELAIMNKYAKRGALFSFLYSI
TIYIGVSVFSTAPLIPFILDLISPLNESRPRLYAYKAYCYFNPDDYYYYVVGYTSACIGMAGLVLV
GADIIMIHLVRBBVFITIVVFSVTLFQVLRLCTFLIAQTVHMLFLTIQGQFVIDSHDTVYNVYEAI
WYKMPPKLQLLDVVALRKSLTPPILTAGGLMRLDLNSFAQLLKACVSYYTALRPSY
>HlOr111like_5
MADIFEDRFFKMSRRCISLVGLWPYDDASFDKSFRRVSIALVYLIFAEVQLAKFFTTRFTFDIFLD
TITAILMELLILAKCSIFWFNGEMLKQLLERVKHDWDAVRGREELIIMRKYTKYYRMFGILYTYFY
SCGIGLFVTATLLPIVMCRIQPRNESHVHTIPFQGEYFFDQEKNMVFIILHMILTVTITHCIAVMT
DMILLVLTMHACALFAVVGHRFERLFDNAPGYAATKDALANWNCRFDERQDYCIEYHLRTMKGVQY
AVHRVLKVCCARCSQLFDKLMPLKSVNEVVIPLFHVWAFLVVVFLNIYPAQMVMDTSEVIFYQAYY
GSWYAAPTRAQKLLLLVLQKCMVPCSLSAAKIVTASLQIYATIVQTSVSYFSVLYALKE
>HlOr111like_6C
LKCIILCKVFIVYLLCTFIMIQLVHVLYLTLPGQFVIDANEEISESIYSALWYNSDKRTQSLFVLA
LRNCLSAPQITAGGLITMNLQSFSEIVKTSVSYYMVLKSV
>HlOr112
MYAETVEERFMKLNKVLGLICGVWPRQNSRRKLITQILSLLLMASSIVTQIANTVLFFSMDNLVDG
IPFNVAAVGTFVKLGNYIINETKLKSMVEQIFDDWTSITSETECKIMVTYARKGQLIALCYAAHLF
LPAAIFLCVPFAPIILDIYAPLNKTRKRVFVYPAYYWMDPEQYYMIMIIHIMFVVIMVCVIFCACD
MNYVYAVQHACGLLAVTRYRFSQAYKTLSINGDENGARFFDKRTYRDVCHSVRGHQRVVQYLKHIE
DSHRIYLFISMGMLMIAISVSLLTMASSHNLPQKVVHGTFFVGQAFHVFFLNVQGQFVINAFDELY
DKIYESQWYNFTPRTQALYVLALRSCLNPPLLTAGGMTTLNLRSFAEIIKASVSYYTVMQTK
>HlOr112like_1
MDAITLEKRYLRINKKLGMLTGVWPYQKSSPKWISRTVVLFVIIPCYVTQYARIVTFPRIHIILND
YPYLITSLGVIIKFGNYFINESELKYLLGKIYEDWRAMTSEDEYKIMTKYAEKGIFSVILYILHIS
LCAALFMCLPFVSPILDILMPLNETRARLFVYPAYYFVDEDKYHYLIVGHMYLVVVMLISVFCACD
ANYVYAVQHACGLLAIAGYRFKYACYEVIPEDEKEAVKMMRKMYRNVCHSIRIHQRALQYVKEITA

AHDTCMFISVGLLMMSISTSLLQISEHEHDAEWTLYCTFFTAQLLHMLFLVVQGQFVLDAYDDVYN
TIYESTWYNSSCKTQALYILALRSSLNPPLLTAGGFITLNLKTFSEIIKSSVSYYTVMQS
>HlOr112like_2
MDAITLEKRYLRINKKLGMLTGVWPYQKSSPKWISRTVVLFVIIPCYVTQYARIVTFPRIHIILND
YPYLITSLGVIIKFGNYFINESEVSAILSKIYEDWRAMTSEDEYKIMTKYAEKGIFSVILYILHIS
LCAALFMCLPFVSPILDILMPLNETRARLFVYPAYYFVDEDKYRYLIVGHMYLVVVMLISVFCACD
ANYVYAVQHACGLLAIAGGYRFKYACYEVIPEDEKEAVKMMKKMYRNVCHSIRIHQRALQYVKEIT
AAHDTCMFISVGLLMMSISTSLLQISEHEHDAEWTLYCTFFTAQLLHMLFLVVQGQFVLDAYDDVY
NTIYESTWYNSSCKTQALYILALRSSLNPPLLTAGGFITLNLKTFSEIIKSSVSYYTVMQS
>HlOr112like_3
MDAITLEKRYLRINKKLGMLTGVWPYQKSSPKWISRTVVLFVIIPCYVTQVEKIVTFPRIHIILND
YPYLITSLGVIVKFGNYFINESELKYLLGKIYEDWRAMTSEDEYKIMTKYAEKGIFSVILYIVHIS
LCAALFMCLPFVSPILDILMPLNETRARLFVYPAYYFVDEDKYHYLIVGHMYLVVVMLISVFCACD
ANYVYAVQHACGLLAIAGYRFKYACYEVIPEDEKEAVKMMRKMYRNVCHSIRIHQRALQYVKEITA
AHDTCMFISVGLLMMSISTSLLQISEHEHDAEWTLYCTFFTAQLLHMLFLVVQGQFVLDAYDDVYN
TIYESTWYNSSCKTQALYILALRSSLNPPLLTAGGFITLNLKTFSEIIKSSVSYYTVMQS
>HlOr112like_4C
KVVHGTFFVGQAFHVFFLNVQGQFVINAFDELYDKIYESQWYNFTPRTQALYVLALRSCLNPPLLT
AGGMTTLNLRSFAEIIKASVSYYTVMQT
>HlOr112like_5F1_F2
MYAETVEERFMKLNKVLGLICGVWPRQNSRRKLITQILSLLLMASSIVTQIANTVLFFSMDNLVDG
IPFNVAAVGTFVKLGNYIINETKLKSMVEQIFDNWTSIKSETECKIMBBVVHGTFFVGQAFHVFFL
NVQGQFVINAFDELYDKYESQWYNFTPRTQALYVLALRSCLNPPLLTAGGMTTLNLRSFAE
>HlOr112like_6N
MYAETVEERFMKLNKVLGLICGVWPRQNSRRKLITQILSLLLMASSIVTQIANTVLFFSMDNLVDG
IPFNVAAVGTFVKLGNYIINETKVR
>HlOr112like_7N
MYAETVEERFMKLNKVLGLICGVWPRQNSRRKLITQILSLLLMASSIVTQIANTVLFFSMDNLVDG
IPFNVAAVGTFVKLGNYIINETKVR
>HlOr112like_8N_C
MYAETVEERFMKLNKVLGLICGVWPRQNSRRKLITQILSLLLMASSIVTQIANTVLFFSMDNLVDG
IPFNVAAVGTFVKLGNYIINETKLKSMVEQIFDDWTSITSETECKIMVTYARKGQLIALCYAAHLF
LPAAIFLCVPFAPIILDICAPLNKTRKRVFVYPAYYWMDPEQYYMIMIIHIKLBBKVVHGTFFVGQ
AFHVFFLNVQGQFVINAFDELYDKIYESQWYNFTPRTQALYVLALRSCLNPPLLTAGGMTTLNLRS
FAEIIKASVSYYTVMQT
>HlOr113
MDAATVQARYVKYTKRFALMAGIWPGQKRLTKYFLRTIVFMVIISSLLAQLARMLQTLTVDVIVEQ
VPFFFAIVLLLCKQGNYIINTDKFEYLLDSMCEDWNQERSYKEIGIMKKYGQRGNLLSRFYVVNAY
ICSFLFIQVPWSTRLVNSYKTQNITPSLPVVVPGYYFVDEVEYYYYIILHGTCTILIVVVVYCACD
TSYMVLVQHTCGLFAVAGYRFKNSIREDNSVKGKYFDHWTKEAYKNACFSIQGHQRAINFVKEIER
GHDTYLFTCLGLVILCFSITLIKLSTSDFDIDFYRYCSFTLCQLMHLFVIMLQGQFVLDSCERIHE
AIYESLWYTSSSKTQALYVVALRRNLTPTCLTAGGLVQLNMQSFAEVVKMSVSYYTVLKTT
>HlOr113like_10PF
KEAYKNACFSIQGHQRAINFVKEIERGHDTYLFTCLGLVILCFSITLIKLSTSDFDIDFYRYCSFT
LCQLMHLFVIMLQGQFVLDSCDRIHEAMXVEGINNRNKRI
>HlOr113like_11C
KFIVYRYESTWYNSSCKTQALYILALRSSLNPPLLTAGGFITLNLKTFSEIIKSSVSYYTVMQSK
>HlOr113like_1C
ASLIQLARMLETLTVDVIVEQTPFFCPLVLLLCKQVNYIVNTDKFEYLLDSMCEDWNHERSYKEIG
IMKKYGQRGNLLSRFYVVNAYICSFLFIQVPWSTRLVNSYKTQNITPSLQFVIPGYYFVDEVEYYY
YIILHGTCTILIVVVVYCACDTSYMVLVQHTCGLFAVAGYRFKNSIREDNSVKGKYFDHWTKEAYK
NACFSIQGHQRAINFVKEIERGHDTYLFTCLGLVILCFSITLIKLSTSDFDIDFYRYCSFTLCQLM

HLFVIMLQGQFVLDSCERIHEAIYESLWYTSSSKTQALYVVALRRNLTPTCLTAGGLVQLNMQSFA
EVVKMSVSYYTVLKTT
>HlOr113like_2C
LARMLQTLTVDVIVEQVPFFFAIVLLLCKQGNYIINTDKFEYLLDSMWEDWNQERSYKEIGIMKKY
GQRGNLLSRFYVVNAYICSFLFIQVPWTTRLVNSYKTQNITPSLQFVIPGYYFVDEVEYYYYIILH
GTCTVLIVVVVYCACDTSYMVLVQHTCGLFAVAGYRFKNSVREDNSVKGKYFDHWTKEAYKNACFS
IQGHQRAINFVKEIERGHDTYLFTCLAMVIVCFSITLIQLSTADLDINFYRNCSFMLCQLMHLFVI
MLQGQFVLDSCDRIHAAIYESLWYTSSSKTQALYVVALRRNLTPTCLTAGGLVQLNMASFLEVVKM
SVSYYTVLKTT
>HlOr113like_3
MERNELEETYLYVSKKCGVVAGIWPYGSDRIKWIGRITMMLITITSLITQIANVLVNLSTDTAIEQ
CGYFMMAVGIILKEMNYIIHARELKTLFECTFNDWITSRPKSEMDIMNKYARRGLFFNRLYVVTGI
CCFTMFISAPYIPHVYDVMLSKNESRKLGFIYPAYYGVDQDKYYYYIIAHMTSGGLVVFFTFISCD
TIYMNIVQHACGLLLISGHRFKWAIEEMGGNNEKLNPTMLEGIHRRVCQSVKAHQHAINYIERIEE
MHHTYLFIVVLVVVLAFSITLLRASMMDPCVEFYKDCVFLVVQLVHLLFLAIQGHFVTVSHDQVYD
SMYDAVWYNSTPRTQTLYMLALRRTLTPPLLTAGGLISLNLETFSKILKTSVSWFTVIKST
>HlOr113like_4
MEASDVERNYLYLSKRCGIVSGIWPYGNDRIKWIGRTTVLFIGATSLITIAHVVMNLSTDTMIEQC
AYVVLAVGIILKEMNYIIHAREVRLPYFRIFNDWITRRPKSEMEIMDKYARRGLFFNRLYVANGVF
CYIMFSSAPFIPHFIDVALSKNESRKLSLMYPAYYGIDQDKYYYYLMAHMMSAGAAVFFVFMSCDT
IYMNIVQHACGLLFISGHRFKCAIEDDGADDEKVNPTMLEDMHGRVRHSVKAHQHAINYLGEIEAM
HHTYLFIVVGMVMFAFSITLLRASMMDPCVEFYKDCSFLVVQLVHLLFLSIQGHFVIVSHDQVYDS
IYSATWYKSTPRTQTLYGLALRRGLTPPVLTAGGLISLNLETFAEILKASVSWFTVMKST
>HlOr113like_5
MEAREVEKTYLYLSRRWGSMAGIWPYGNDRTKWIGRVTMILVAASSLLTQIAHILVNRNTDVVIEQ
CPFLMVAIGIILKEANYIIHEKQLKEMIECLFNDWVTTRPKSEIEIMDKYARRGLFFNRLYVANGI
FCYVIFTSVAITPRVLDLISPKNESRGVGFIYPAYYGLDEEEHYYYLMGHMLSVITVVFFVYISCD
TIYMNVVQHACGLLNVSGHRFKWAIAAEKSNAKHAFMMEEAYARVCHSVKAHQHAINYITEIEQIH
GTYLFIAVGMIMMSFSITLVRVSMMNPCVEFYKYCGFLVVQLVHLLFLSIQGHFVIVSHDMTYDNI
CAATWYNSTSRTQALYMLALRRNLTPPLITAGGLISLNLETFAEILKASVSWFTVMKSA
>HlOr113like_6F
SSSLFIFQHSKFSLLARYLLIFRTGLFALFQLARMLQTLTVDMIVEQTPFFCPLVLLLCKQVNYIV
NTDKFEYLLDSMCEDWNQERSYKEIGIMKKYGQRGNLLSRFYVVNAYICSFLFIQVPWTTRMVNSY
KTQNITPSLPVVVPGYYFVDEVEYYYYIILHGTCTVLIVVVVYCACDTSYMVLVQHTCGLFAVAGY
RFKNSICEDNSVKGKYFDHWTKEAYKNACFSIQGHQRAINFVKEIERGHDTYLFTCLALVILCFSI
TLIKLSTSDFDIDFYRYCSFTLCQLMHLFVIMLQGQFVLDSCERIHEAM
>HlOr113like_7C
MNDTKLQLKEMIECLFNDWVTTRPKSEMDIMDKYAKRGRFFSRLYVVNGLFCYVIFTSVAITPRVL
DLISPKNESRGVDFIYPAYYGLDEEEHYYILMGHMLCVITVVFFVYISCDTIYMNIVQHACGLLNV
SGHCFKWAVETVITKNEKMHTEVDDTYRKVRHSIKAHQHAMEYIMQIEDVHVSYLFIMVAVVVLSF
SISLVRTSMMDPCMEFYKYCGFLVVQLVHLLFLSIQGHFIILSHDMTYDNIYSAKWYNSSPRTQTL
YVLALRRSLTPPLLTAGGLISLNLETFAEILKASVSFFTVMKST
>HlOr113like_8C
RYIMQIEDVHDSYLFIIVALIMLSFSITLVRTSMMDPCMEFYKYCAFLVVQLVHLLFLSIQGHFVI
LSHDMTYDNIYSAKWYNSSPRTQTLYVLALRRSLTPPLLTAGGLISLNLETFAEILKGSVSFFTVM
KST
>HlOr113like_9C
YITEIEQVHGTYLFIAVGMVMMSFSITLVRVSTMNPCVEFYKYCGFLVVQLVHLLFLSIQGHFVIV
SHDTTYDNIYSATWYNGTPKVQALYVLALRRNLTPPLITAGGLISLNLETFAEV
>HlOr114
MIDLKYVYGWNHYTMRFMGIWPEERKWNQPSSYLALIPVLMMFLFVCVPQTINLSFIWGDFNLVVE
NLSMGNITITISLLKTIAFWINGKPLKSLLKCMADDWSTVSKKDDLDTMSNIARITRKTIISSTVL

CHIVVITYIFLRFLTRKYSGTRLLFRAYFPYDSDVSPNYEFTVIAQIIAAFYAATTYTAVDTFIVM
LILHVCGQLSNLRNEFGKLQACDKVTLQAKLGKIVQKHEYLNRFAETIERCFNMMLLIQMLGCTVQ
LCFQCFQAVMSIDEEIDENMIFQILFLLFYVVYVMLQLFLYCYVGERLSVESVEIANAAYDSEWYN
LSPKNAKLLLIIMRRARLPLQITAGRFATFTLMLYSQILKTSVGKGYVSVLYAMKYKETTL
>HlOr114like_1
MELVGIWPEPSRSDQRWPNFKALLFIFVIIFFGTGPQSVNLLFIWGDLGLVTENLSTANIPGINAM
MKLIIAWHHKAFKPVIKSFYDDWKTSRTKEEKETMLKRAKFAKQISIWCSVLTLTMVTVYLSLRAW
IIYQLDRANQKQDRLLLYPGYFPFDIRPIGVLLSTNFGQVLAAYSAVISYTTVDTFIAMLIMHICG
QFEILRQKLRRLMDDEKKTRSVDEIQKELVLIIERHEHLNWFATMIEDCFNTLLLIQMLLCTVEIC
FQGFLFFDVILENEKGIFNVQLLFFVLFVCFILVHIYIYCYIGEMLLTHSREMSNSAYESNWYNVS
PPEAKCLLFIMHRTARPLCLTAGKFSTFSMEMFSTILKTAMGYLSVLLTVTGSD
>HlOr115
MDFAMGWNRFSLSVLGVWPEPRKMSNLSRFASNFIFWFTTLITVVFICAPQMTHLVLKSTNLDEVI
ENLSVNIPMLFSLGKQIILRYRRKALVFLMSQIFDDWAEPIPEVDRHTMLKTAKISRMISIVCSIL
TYVMVLAFLSLQIWSSMQNTSEFNVGGLLIPATFPYDTNKSPNYEITWLGQFVGTVLSAICYSCFD
TFVAVLVLHICGQLTVLRIALENLANTTIKDNYSKFQERLGYIVHRQNQLFRYAVIVEDCFNIMLL
VQTLMCTAMLCMTGYRMMTSVNQEQEDVPIIGVIFFISHVTYTMLHLFIYCYVGETLLGQSTGIGQ
SAYDCNWYDLPPKHAVSLVIVICRAQVSFQITAGKFSPFSLELFNAVLKTSAGYLSVLLAMKDRLV
EEN
>HlOr116
MDEFAGKQTKLKKIKPNKHLKNCMLLFYYAGIWPGQMKYKRLYYLYGISSFVILLAFTLVTEFAYL
CVHWGDYAEIMSGATIFMTNYSYCLKVIVIVLRRKRIKDLINITEGKLFIRNNDKYERIVTYYTWQ
GIFHHITYQSFGIIAVIFWSCTAVVNLMKRTYQLLLIKAWYPYNVTISPAYEFTILHQVVGVTVNC
INNVAIDTFITGFIITACCQLTLLSYNISSIHYEAEKEFVARTDDISADKSTSKTYNQLYEDLKIC
IEHSRLISDFARKIQDTFGTVIFFQLLVNCITVCLLAYNISQLKYYVLSDLFGMFMHMCCMTYQIF
IYCWHGNELYLHSMNICFAAYMNNWWHNTKDFKRALLVVMSKAQRPIILTVGNIMELSLENFVLIL
RTSYSIFTVLKTSTNA
>HlOr117
MKELSKASIDYYILPNKIFCSIAGMWPIDEKSSTRSKLFAYFRLIFGLTAISSVFVPEIMAIVWNW
GDIRILAGVGCVLTTVGQLLFKMIYLIARRDKTYKLCNEIRDLWDSSDDPKERQSYEELAHWARNL
TIIFYSSCMCNVFTFTIAAAFDYFTIEYSVSDADKSRHLPFEVWYGIDITPSPRFEIAFLCQIVAS
MICAAAISGLDATFMTTILHVSGQFKLINRWISKIGIEINIEPDYPRKLKANLIKCIRHHQRMINV
VNDVNNLLTPIIFMQLLTSGIEICLSGFAVIDNGTGTDLLKFVSYLTSMGVQLLLWCWPGEILVQE
SLEIGHVVYLNIPWYNLPPIYQKQLCLMIIRAQQYCSITALTFQTLSIHTLTAVFNTAASYFTLLR
QMQQT
>HlOr117like_1
MKEPSKTSIDYYILPNKILCGMAGVWPIDEKSSTRTKLFAYFRLIFGLVALNSMLVPEIMMVISNW
GNLRILAGVGCVLTTLGQSLFKTIYLIMRRDKTYRLYYEIRDLWHSTDDPKERQNFENLAYWARTI
TIVFYSACMWNVFTFTFAATHDYFMIEYNISNAEKSKHLPFEVWYGTDITASPLFEIAFVCQLIAV
AFVAASTSGLDGSCMTTILHVSGQFKLISTWVSKMGVETNVKPVYSRKLEVDLIKCIRHHQRMINV
VNDVNNLFTPIVFVQLLTSGIVICLSGFAVFDNDARTDILKYVSYLSSIWLQLLLWCLPGEILVQE
SLEIGHVVYLNVPWYNLPPIYQRKLCMMILRAQQYCSITALTFRTLSIHTLTSVFNTAASYFTLLR
QMATK
>HlOr118
MTSTTIINRPLEYSLRLFGAWPDSSYSFLKSIIWSTIMATFLMFQYWYCITHIRSGLIDLLDGLSI
TLSNSLVFLKLIIIWLHSRTFYGILTTVFEDCNNYASTVENKRIMTDKAMLSSRISNFLIGYFSIT
FLVYSGLALVLFDEDQNGPVSKQRKFLIRMEFPFEATVSPRYEIILVVQFIFEAFIVYGAATSIAL
IAVLILHVGGQIDLLCQNMRDISRNHEKKVPQKLTIKDVVVRHQRIIRLSKNIETVFTYISLCQFL
SNMLVICFISFVLVTSLNTEQTTGLILKCFPYYVAVNCEAFILCYTGEYLTSKSERITQSVYNFLW
YDLKPREARIVLLIILRSQKELILTAGKFVNLSLAAFANMLKASASYVSVLHAMC
>HlOr119

MAYVPGHEWMRSFRQLIHVQISYIKYSGLWKIESKHSSFMTIAYFAYKLWILIMMNVFAVTIFADI
YENMDNLSIISDDGCFLAGILVVIFKAMNYQIHQKSIIKLLDDILNCADDLCRFSNTETNKYIINR
YYKFNKVVFYGFSMLGCVLGIALLFFSPLQNGLPIRAKYPFNTTISPFREIGLAVETFAVSGGLLS
IVAMDNITAMMCSQITAQFDMLNVLFEKCTSQVENKNVNVTDEIFKTETNRINKTHDNTFLRRYKT
CLQFHQRLISITKDYNRIYSSSMFVQMISSTSIICLTGFQAVVVGGQSSDIMKFGIYLSAAISQLL
YICWLGNELSYSSSVIDRSQWLSGWNHEHLTSIVKIFTLSTMFTRQSITLKAGVFYVLSLETFIAI
VRRSYSVFTLLNNMHSTSP
>HlOr120
MNIKNYVFINQLALKFVGLYPISIIRYLICISCIMLIIIPQITMIYTNWDDLNIVLETSSSLLTIS
LATLKSIIWMFNRKKLAVFIEFMLTDYWEIVKTNVSEYLQEYAIYAKNITKGYFISMCNALLFFCS
LPIIEIFVTEHQNSDNLTLKNFPFAATYPTVFYNFPFYEIAYVSQALATSICCLMILATDGLIATA
VLHTCGHFAVLKENLRRLDLCIYRVCVNSFENNSKHINAKLYQIKVQITHLIKHHVVLWFCDNMEK
NFHLMLLLQTMTSSLMICFVGFQVSTTLTEQSKVIKFASHLIVTLFQLLLFCFPGDMLIRQSFSIS
TAAYSIQWCQLPTFLKDEVCMIILRSQRSSYITAGKIYVMHLENFTAILSTACSYFMMLQSFNSEA
>HlOr121P
MNRSSLRIIGLWFEEDHAGTMKFLRKLRNMGIVLIILFVCVLPCLYSLIKQCDSLMAVIDNLSYTI
PLTITLMKYIVILSKKEVLLPLVNMIAEDWAKLKTDFERDVMIQRTRITRATSILSYALIFTAVMF
MIVLPRTGITTRYVTNETNIKKLFPVPTYYIYDISETPYFEIMFLLQMATLVLMALCYIGVDNFFG
ILILHICGQLENLRTRLANIKHLETFDRILVTTVEDHVRLIRAVDVIESIFTMLVLALLFYFGTFA
CIYGLLLMTILTQEEQFSVLRITYLACIFLNTFLQTSLYCITGQILVNQSEEVYVAAYECEWLNLK
SNKAKSLILIMARAQRPLCLTAGKLFPMTMLTFCXRFATSLSYISFLLTKL
>HlOr130like_1
MDVFNKQYRTYRLMLSLLGLWPYSQSIYSTIHRISFATIILSCIIFEIATLVSFERASENILQTCF
VCPLLVLFFERYVSFICNFPFVKSIFECIQTDVYTLQDSNEMKILTKYIDDSSRIITIFLYAAYFG
ITIIVTTLLYPVSMDLLVPLNNSRPRYLGYFVILPSNQIQYIDFMTFYGTLVPFVGLLTVACTESL
LSISAMYLCSLYKITGYRLGKAISNATKTSFMSVQNYTDFYCAVDIHTRIIKLVNILQSKMKVPYM
MASVLAVVSFVLCLSQIVKAIVTMNDQLEILLSIMIVSNHIVFLFFNNYAGQLLINHSEEVFVESY
NSTWYCVPLSAQKLLLFIMMKSTIPCKINLSGIFAPCYKGFSSMISTSFSYVTAIYSTYN
>HlOr130P
MDVFNKQYRTYRLMLSLLGLWPYSQSIYSTIHRISFATIMLSCIIFEIATLVSFERASENILQTCF
VCPLLVLFFERYVSFICNFQFVKFIFECIQTDVYTLQDSNEMKILTKYIDDSSRIITIFLYAAYFG
ITITVTTLLYPVSMDLLVPLNKSRPRYLGYFFILPSNQIQYIDFMTFYGTLVPFVGLLTVTCTESL
LSIIAMYVSSLYKITGYXNVHGQFSYISSYRLGSNATKTNYTDFYCAVDIHTRIIKLVNILQSKMK
VPYMMASVLAVVSXHVSQIVKAIVTMNDQLEILLSIMIVSNHIVFLFFNNYAGQLLINHSEEVFVE
SYNSTWYCVPLSAQKLLLFIMMKSTIPCKMNLSGIFAPCYKGFSSMISTSFSYVAAIYS
>HlOr140TRA
MDFFEQPAYLLNKRLLTIVGIWPFQSTSSRLLRQTVMLFFCFTLLIAELRGLYRIWGVDYDVVIEC
MPPIISIFLSLTMYLNGIFNMNKIKDVLLFIKYNCNYYMNQPENMILKYYERQGKKITLYYTSYVY
MTLLIYLLLPPMSLIIDDVISSNYSQERNFLFELDYGVDSQQYFYYISIHSYMGTAVVANLIATCD
TTYMLYSQHAYALFAIVCSQLKTVHILDTGNLINVKDSYILEKYKNIELLPGEQKKVCRKLLNCIK
EHQNAIEYSSVLESLFTKSILSQLFCNIICLSITGVETVMKLGNIGDMIRFGSFTFAQAVHIFLIC
LPGQRLVNHSEQVYATACEVMWYTLPKKCHSLYKFLLARSSKPSKITAYKLAPMTMETFLAIIQTA
MSYFTVLLSTT
>HlOr140TRAlike_1
MDFFDQPTFSLNKFLAVLFGTWPFQSITQTVVRNILVSIGIISIIIPEVNHLCKTWGVDKDVSFGC
MSSLLTVIIPMIFQYNSLLRKKGVNIFLIFIKHDYERLINLPEYNVLYSVAIKGRRNSLVYIIVYV
CISATMYFTLSIAVVIFEELNKNRTEPRQTLYPVEYHIDSEKYFYLITAHCYASTVLLLLQTVVHD
TMYIVLVQHGCALFAVTGQVYCLKGAHCLDSKGLLKDDDFEQYKNRCYTLSEQKKIYQRLVLGIKE
HKRAIEYTKLLHAVFSECLFIVLMLNIISLSINGVQALINLHEIQTSIRLILWEIGLFIHLFWLCL
PGQRLTDFSERVYYDVLNCLWYTFYSISHVLYRFLIMNTLKPCELIALKIIPLNMETFLSVTQTAI
SYFTVLSST
>HlOr141

MKGKDEGGEFDRLIGLNLDLLRLCGVIPCGSGFLGRNGLACVAFGCLTVYSISYTYQFVTCMENLD
TIFESFTMIISIVGGQARFSILSWFRGTCRTMLEACEFLWSTLKPREKEIVRSYANKTRLLTRCYL
ASCVFTIFIYGVFALFDQSQPSPENLSENGTVDSTVRRLPYVFFLEVQQTPWYEITCALQLFAMLN
VGITCVGVDTIGPLFVLLTCAHFDVIRSRIESLHAIDRSSSSVQTRNLRVLVIRHRMLLEFCQDIE
RVTNVMFFTQLFGSTYNISLVGFKLIGDDPDKYKYISQLSIAIIQLFLCNWPADFLYSKSEAIGRA
AYTVPWHWYPRCLRKPTNMLMIRGQKPVRLTAGKIVGLSLETFASMISSAVSFFTVVRTMN
>HlOr142
MRKSITPEQAISFTKLSVAVTCSWPPSPLATRTQVLLFNTLWSTAFVSTIALLLPLLSGIYEYRKD
PIILGKTVSLASAVAQVAIKMIMCRFQQKQFQVLYFDMENFCRYATSKEKVVLQRYVDRYKYFHGI
YILWCFLTTIFVISGPLYSPQTFPTHAIYPFQVEHTLFKIVIYFHQSLVGFQASAGMAIDTQIALL
LRYAAARFELLGNEMRNANTHCEFNACIEKHSELLRYTKTIRQSIKFLILTTIATTTVAVIFGSLN
LIANQPLLLKALYAVVVFSASVELFMYAWPADNLMRMSTKLAADIYNTDWFEKDIRLQKKVYYIIQ
RSQRLEAIRINGIVPKLSLPYYAMYLYKSLSYFTALRIMVESETAVESI
>HlOr143
MSLFNANIHRVLHILELAGRFTCTWPLDSNSSKREVVFRNVRWGFTILNVILLLISLILAIFHFRN
DITILMKTISEMTALLEVLMDMILCKMKSAQLQVLVGKVKAFVKVANKNEIKIIQGYVDRYKRFFG
TIAMGYVMTGITFSLAPLFSVQELPADGWIPFSIKPLGVYCLVYFVQVYCILQTAFCISVDFMIAL
LISFSAAKLDILGMKLRKANGHDVLVSCVKEHQEIIGFVEDIKTSVETLLFKTNATMGSAVICGTF
PLIYNQSLTVISQFLPLMLSGCGRLYVISWPADDLKESSITFAKSLIDSPWIGKPRKMTNIVIIMM
QRSQRILLITMGGILPAISLEYYANFLTTVGSYFMAMRTMIES
>HlOr146
MLKYPSPERAISLVQVITAFTCCWPLPSTATKSLVLQFKILRSFLLLNALVLFLPLLYALHVHRND
AENVAKAACLTLALVQIFFQTFVCIGQYDHFQKLIEEMKICCETAKPYERDVFQRYVDKYSLFYVT
CSSWFYLTAVIMVLGSRIISDPFPTNAVYPFPVNFEPLRSIIFMHQSFVGIQCAAHTSMNVLTALL
LLFAAARFEILMMELRNVSDIETLIKCMKKYSIVRRYATDVVTTIRLLVLITVITCTVACVFAGIN
LIGKQPFAVKAQFLTVSGTALLEVLMCSLPADHLIDMSENIMQGIYESKWYERSLKIQKSTVLMLT
PQSPVTVKIKCLIPVLSLNFYCSYVSNVFSLFTALRIVMIDDENGN
>HlOr146like_1
MLKYPSPERAISLVQVITAFTCCWPLPSTATKSLVLQFKILRSFLLLNALVLFLPLLYALHVHRND
AENVAKAACLTLALVQIFFQTFVCIGQYDHFQKLIEEMKICCETAKPYERDVFQRYVNKHSLFYIT
CSSWFYLTAIIMVLGSLLISDPFPTNAVYPFPVNFEPLRSIIFMHQSFVGIQCAAHASMNVLSALL
LLFTTARFEILMTELRNVNDIETLIKSMKKYSTVRRYATDVVTTIRFLVLSTVITCTVACVFAGIN
LIGKQPLAVKVQFLTMSGTALLEVFMCSLPADHLIDMSENIMQGIYESKWYERSLKIQKSTVLMLT
PQSPVTVKIKCLIPVLSLNFYCSYVSNVFSLFTALRIAMINDETEN
>HlOr146like_2
MLKYPSPERAISLVHVITAFTCCWPLPSTATKSLVLQFKILRSFLLLNALVLFLPLLYALHVHRND
AENVAKAACLTLALVQIFFQTFVCIGQYDHFQKLIEEMKICCETAKPYERDVFQRYVNKHSLFYIT
CSSWFYLTAIIMVLGSLLISDPFPTNAVYPFPVNFEPLRSIIFMHQSFVGIQCAAHASMNVLSALL
LLFTTARFEILMTELRNVNDIETLIKSMKKYSTVRSDREVAEEVGAVRRGNRFKRVLRTRTNLLIR
LPKQPFAVKAQFLTVSGTALLEVLMCSLPADHLIDMSENIMQGIYESKWYERSLKIQKSTVLMLTP
QSPVTVKIKCLIPVLSLNFYCSYVSNVFSLFTALRIAMINDETEN
>HlOr152
MLKQLTPEKVIDIIWFSVALAVCWPLSSSASKAQVVAFKILQICSAISALILLLPLLYSTYLHFDD
VIVLSQSICLSMGVFQMIMQTVICFTNYDLLQRIITEMITYVKEAQQFEREILHNYIQKCKLFYGG
SLIWTYLCATCFVLGPVVLPNPFPSSAEYPFRVNRTPMNAIVYLHQSFISFQYSAHICVSIFGALL
LWITAARFHCLTLEVQQSSSIHMLIICVKKQLLLRRYAKEVVDGFRLIVLNAIAMSILALTLSSIT
LIMEIPLIVKVQFVTVSFTVLAEIYMYAWSADYMKDMSAKVSQSVYDLTWYGQTLEMQKNVLYILV
HQEPVILSVSCIVPELSLRYYCSYLSNAFSVFTALRVVLDAKSA
>HlOr159
MKFVDYHCYKSNPIILFIVTLWACDNVWLKRLQNLISFFVLTSALFVQLMPFATQSLKFGDILNDI
LFISLTLTFIIKYVTFYYSTKQVKKLFNSIHRHWDELDNKEERKIMEKYSDNGGFFVMLSIMYLIP
IIIAVILLPFYDIIFNIVTSLNVSREIELPADLEYFTDESKYYPIKLLHVVSHAITGVTVVIFIDS

TLLIFLQYSCALFKTTYSYRIGYAIESYSWLNEPQRYYIILTKLIKAVESHCLAIKFVGQLTNCFK
YCYMFSLLLFVPSVSINLYCLSQSMLTMDDFEDLCRNIFLLMGQLGYLFIINYMGQDLVNHNNEVF
QTAYNSLWYIAPTPVRKLLLTMMLQNVRQRVFKLMGGLYITNIEGFSMILRTCISYFMFMYSTQY
>HlOr159like_1PN_PF
MDFVGNDYNGFIRKLLKFLGLGPYQKVTWFIRIWRLIIAIILINGIIFQVNVIRIPRETFLIYYGN
LVVLRLQIHLFLHHLSQSKRILAXVKSILERDQQDWKTLKNEDEVKIMRNCRKQANLLIVIFSNKE
RILIBBRYIDITVSIFSATYFVLIGLGVVIVTATLFRVSLRQALISMELTSCLXLPDLCLQLFYIF
ICNYIGQGVKNCSSEVFYDICNSVNWYTAPIAIQKLLLIIMQNTIRNSSITLFGMYISSFDGFFMV
NNTII
>HlOr160
MRRSVSLNVELFYDENVLSWSKRLLGLSGLWPDNRNDVRFFFYISYVVIFTWLEMVTLLQNIHDLE
KSLKNITLSFPTILIVLKAVMFRLNMHLVLPLLEAVRKDVKQGLYRSQEERRTVVWYSVAATLFST
SSAMSLFFVPTLFYTKPIVGCLLSKFSNCTLPYELPMKVNHLYEVTEVRTYALFCVYFVPTSMLLT
IGATGADSLLVTLTFHLCSQFSILSSRVKNIDVEPQKYFPQMKALVERHTELLRLAGILAETFSTL
MFIQTLGLIFSLCIVVFQLLTMSESGEDMNTIHFIIYSCAVILLAFCYCFLGECLINESSEMQQAC
YFSNWYDLPDKYTRSLIFCIARSQKPSYLTAGKFYVFSLETFGIIVKASMAYLSVLKSII
>HlOr161
MNARPYNNKEYDELIRPIMLTAKIISIWPLAENSATGAILFRRFHLLCMFFLVVVMSIAVTADVIH
NIDDLDEATECALICTAFYLCVVRMVVYAIHQKDMLYVVNTMRIDWTESSKEDRVILAEKTMFAFR
LAKYFISTVAATIVMFMCIPILEIYVLGKDKVLPFRGYFFLNQTVSPIFECLYLFNVTAGGFGGTM
IASCTSFNLVVIMHGSAKFAVLRRRLEALSGKDPNSTTVMGDLVIRHQEAIEYADALERIINVLAL
GQFVISTGLICFAGFQITSMMEDKGRLMKYSTFLNSAILELFMFSFSGNGLIDESDAVGESAYGSG
WIGSRFSQSLQIMMMRSRIPCKITAAKFYSMSLESFSKVLSTSFSYFTVLTATKDD
>HlOr162
MDNTQYEYLRFNKYFLFMIGVWPYQTVLQRILVGIIFIPIISAQTILQTGGMVTAMTDGDVESFLE
SFAPLIISLMCLSKYINFFYNYKKMRYLLNIMQEDWRIYKKVKNEFDVLCEQYAVGKKLTTSFAAF
LFGLITPFAAMPLLLNAADTLGFSNVSSDRPLVFRVEHFVDVDKYYYTLLVHSYFGTIAFTAIVVS
INSIIAVYVLHECGLCEILRVKLENFVEDDTINVELHLNKRIDRWYQNFRECVLLHKHIIEFANLL
EEANTTSYLFQLGFNMICVSFTQFQTVINLQDTPKALRYVSVTICLLCDLLFVSWPGQRLSDYTER
IFEYTTNGKWYESSYNCRKLLIIMLSKSLTSLKLTACKIYTLNLESFNAVVQTSFSYTMVLCSLQ
>HlOr162like_1
MDLFQNRYYKISKFLLILIGQWPYDHSWTKMCKQIFIYSYLLSYLGVQMCLLWTSEMNFELLVLVL
SPSLVNILCMAKYLFYSLKGDVKLLENLRHDWTVLNNANETKVLKMHAAVGGKLMLFYIGFVYIGI
FSYAIVQFLPYIIDVIAPLNESRHHVLPYAGEYFVDQQKYFLPIALHMLGTVTLGLTVATAVDSIF
IFFMFHVCAKFNILGDRLEHFMDDNRDKKQPPLHGNAGSHFLQMKIARCVRFHQQTIQLVKLIMDC
YNASYVVQGSLNPIIITVIMLRVLIQMFMCSKLGMFINGRYCHLIQMFMCSKLTFPMQKLLDNSSD
ILYRMFYSRWYDAPLKIQKLVLIIMNRSFEPVGLRIGDYQDVTIENFSSIIQVSFSSFMVLYSLQ
>HlOr165like_1C
GVFSDILSTMEEDCQKYAAIDINKLMSKTGQLSFYLTSIVMSLYMASAAFYITNTIGFQGNNASVS
RELLMKMDLPFETSESPNYELVVTTQVLIHLSVAVAYGAFNALLLMVVLHLGCQIDIMCRNLLDVS
FKDGKQIKFFINRHQEIIVFAERIEKLFTYMALSQLVSNTIIICCVGFLVVIAVHIDNGVPLLLKS
LLFYVVICLEAFIYCFVGEYLSIKSQLIGNTAYEFLWYDLRSTESQLLIPVILRSQKGFTFTFGKF
SSLSLESFTAIMKVSASYISVLLAVY
>HlOr165like_2F
IFHSVLSDILSTMEEDCQKYAAVDINKLMSKTGQLSFYLTSIIMSSYMVSAAFYITGTIGFQENNA
SVSRELLLKMDLPFGTSESPNYELVVTTQVLIHVSAALTFGVFSALLLMVVSMNNLYLFK
>HlOr165like_3N
MILRNTMSQSVKFGLHFVGIWPGTPFPSLHKIGWVIAMAVLQTYQYRYIITHYKSDSLMVTIDNLS
ITMPFTLVLIKLIITWANYG
>HlOr166
MTSINTISQTVKHGLRYAASWPGTPSLILHKFFWTIVMCGLQIYQYGYVIRHYKYNTLVDTVDNLS
ICMPFTLVCIKLFVAWTHRGLLYDIISTMEEDCKKYAVLDTKNLISKTAHLSFRLTNIVICICVSS

TACYAIGVLAPQRTNITAPRELLIKMDLPFNTNNSPIYELVIVLQYLCKVSTAFTFGVFSSLLLMM
VLHLGCQIDIMCQTLKEVPYKNKTQLRFFISRHQQIIIFAERIEKLFTYLALSQLLSNTLITCCVG
YIIAFALQNNNGFKMLIKFVVYYMAVCSEAFIYCFAGEYLNIKSKLIGDTAYEFLWYNMHSKESRL
MLLVILRSQKGFTFTFGKFASLSMESFAGIMKASASYISVLLAMS
>HlOr166like_1C
SQLIGNTAYEFLWYDLRSTESQLLIPVILRSQKGFTFTFGKFSSLSLESFTAIMKVSASYISVLLA
VY
>HlOr167
MSHNKSISRPVEIGLRLTGIWPGSSYEIIVRVTWIILMTVVQIFQYRYIIKHLDGSNLANLIDSVG
TTLPYSLLYVKLIIFWTKRRIFENMLVGMSNDWSNSSSTKFDINAMISKAELAYRYSKVIMSTYAI
AVFAYTSVFFEFMRHDNDGEYDLKSRELLMKMDLPFAYYKTPTYQWVFVVQFLQLLINASAIGMLD
ALIITLIFHIGGQIEMLQKALTNISIKDEKHRLSRNTTKSLIHRHHRIIINSDSIESLFSYIALMQ
WLCNTLVICCIGFLIVIVSNDGNIKTFIKTLLFYIAITMEAFIFSFAGEYLSNKSLSISNAAYSSP
WYLLDPQDRHIIILLMIRSQRRLTITAGKFMDLSLEGFANVSLLFSYVMHML
>HlOr169
MDFQSLSQLNMLINKYSGNQLPMTDKKTKLPYILKSYSIMSWLIQLTYLTVCVFGLFNVPRDKALK
DGTVNIVVALEEIVLVIYLHNRKTLVRRLIGELNRVLAVDNEMLRNVTINTVKSLEKPLKIYTIAS
VNAVMLWTALPLVQVFRRSEFYYTDYSVPAALSKQPFSYDVFVGGVVLQIIGGVYTVMKKIGVDIY
TMHLILLMTAQYKYIRGKVAMTFRQYNEAFDDFGNSITGLKVSCEKQKVIKQEMRSASHHYEVIVK
MTIMLKKLLSPNIGILYVNNVFRFCFLSLMIITNSGDNFEKLIIVMYTIGALTQFYVLCFCIQELV
EASTSVADDVVHERWYFCDVLVQRVIAMIILADKMECRLSSFRNIDLTLPSFMSILNQAYSTSLLF
LKAK
>HlOr172
MDFAGSQYYRFNRILLSTIGLSPYNSTKYTKFWNTFIILVLFSGIFVQLMTFITIKMNAKQFLKTL
SYIAISFSFVSKYLTFLIHFDVEKEFLERIEHDWNSLTNKEELRIIHEYSSVTRVLTICFSLIIGP
SLLVLILGIFNNSILDILIPLNYTRPRTIPINSEHFVDQQKYFYPIAAHVVLFVIIGAIVLLSTEI
LIMAIIQHTCGLYTIVGYRIDHTFVANIPGLSPAERRVLIHRKIVSLVNLHKRVKELVSCYQVIVL
LTLQQNNIPNFVAFSYPHKLVKAVTKSKQLDEMLEFLFFACGIICYEFVANYFAQKIINHSSHIFV
HAYNSEWYRIPVAEQKVLLLLMQNSLKDDMILLGGIIPASFQGFSLMIRTSFSYFMVLYSFE
>HlOr172like_1C
IREIYDNIADDWKSLKNMEEIEIIRKYANGGRRFTLAIAISTYCGALVITTLQLFPILLDIVVPLN
ESRPCELYLTAEYFVDMKKYMHYIIAHIITCLLIAATTLTANESLLITNVLHVCGLFKLTCSYRLE
HSLDHVTSQKLGSTKRTYVRLIEAVNIHRKAYETISIHLVSQLLEEVIAMENMKELLAAMLLMISH
FLYTFLANYAGQIVMDHSIDVFACIRRYLIPWYRAPLRIQKYLLFISERSKRVSSLCMYGMIIASF
EFFATFMKAAMSYCTVLYSINN
>HlOr172like_2
MDFSGYHYYKFSNDLLRYLGLWNLNAGRLERLQRFVFCSVFVMGIVVQRAAFFTHEYDLQLLSRSV
VFIVGVYMFLNKYMMLYINMDKVRLLFLRVRYDWNTLQNTDEYNILKKYTESATLVNKIHLMCFIP
VILFICLLEVLPDILDVVAPLNESRSHQYLITAEFFITDSSPNYIVTCVGTDITLIMGVTTIIASE
TFTFLLLQHICAMFKITCYRIQYAINQKEIRLIYTKLINAVVTHQRTIQLVLRFAEITEKTIATHF
AYLLVAGLLSITMNMYNVSILWLLSQVILVTKDAKNFSEAAFFVIYQYFYMLMCNNLGQMITDHSF
DVFTQTYDTQWYRLPLPAQKLVIFIMQRSMKSNVFTVCKLYVPSRQGVAKKNESCIRYFHVYISYS
YMFTVHLLELQ
>HlOr172like_3
MDVEKIPFYSFGRSMNVLTGMWPKTGLLTQIHRCFITILLASNILNQLQLGQFFQLGALVTYKNNY
QICCKIIAFVMPTLVYLLKYTFHIVKVKNNSKLLEYVRTDWNNYTKKGEVEIMTKNTKSERNSIIC
FLSIVFLCLIMIALIPYLPRIFAIVTHQNRTRPLKIPIIVEYHVDHNEYFDLIIILHINFCHFIGFC
VVISAEVTNLVLAKHICSLFDLVSYRVRIAVDDTILHSNEYFIRKRAKAVVEIHCRAIFLVDIMNE
MFAFHYATLMVIGVLSLSLNFYVCAQAIFYTGNLEESLLEILLIFSQVLYTFYCCFVGQLIQNKSY
GIFFTVIVLYIYIFRYCTQWYLAPMPTQKLILLVMTRCMRGSKFKTIFILEASLELFSMIMSATMS
YCMVLMSLS
>HlOr172like_4PN_PC

MDFAGEQYYVYVKFFSSIIGLWPYTTGRLKTFCNMFIYILFSMAFLIQVBBTNLLITSVILSMSPL
ILDIIIPLNTSRPRYFPFTFDFIPNQEEYFXPITIFSGILCICGLAAMISSEGLLMMNVLHVRGLY
NIATSYRLKKAMSKNSLQMPVSEKRVLIQKGITESMEQFCQEIFRMTNYNAVFIYSLVLSESALFV
FYIYSRIQILLDESMRFFDSLYNSEWYCSPLPIQKLLHFIMQGRINLNIFFMYGTFVPSMEGFGAH
AKITWSYFVMLYSTQQR
>HlOr174like_1
MDFLGYEYYSLNQQLLTLVGLWPYKRYKLTFLYNTYMLFFFITAFFLQVLVLFFTTELDVDTILSD
GACIIMLLIYIVKYCTFCKQAKNVSLMVSTVEICSFFILCMIFRFIALNFNNFIIFLIFTISVVFA
LSLISFTLVEFVVHFLDILMPMNVSTRHELPFLIQCFCDQDKFFHYILVFLIVLTILGGSVVLATT
TTYLIFSQHCCGLFKIAGYKIQHLLDNDEFEVSDIDQNLTVYLRIINAVKMHKRALVFLDGIKTTF
QMSYTILVILAVISLILNLFQSMMIMHNTKKSLLILLVIIIHFAIIFFCNYTVQNIIDSSTSIFYD
TYYTKWYEAPVFVQKMYLFLMLRTTKDVKIVLGFYTPSIEGFSSLMNITFSYFMVLYSVRMTRM
>HlOr174like_2
MDSLEHHHYKLSKLFLSINGLWPYENSWFMHVKRLLSFVTVVSCIVLLSQNSSLQTSIQHVLIHYF
LFFLIERFFVSNSSSGFQITTYFMHRKKCSKIILLNTLTYVMLTLVLWISKSGSFVTFIVTVVIYL
ATCIYIYIMYKKPLNLSICESSSNLYFTIDYFVDDEKYFYVKFLHVYLYFSLGSTVILGSEVTTIM
FANFASGLYQVVCYRARSVFSKKVLNASSTSRDSVVHEKLVEVVKMHRRAVELVNLLGESFGSSYF
LLLLLGVACTSINLVHVTQIKMPRSNLQLLILEVSMILACFAYLFLTNHMGQTIMDSSSKLLGEAY
SSGWYTASVPAQKTLLYIMQHSIQDYNFSIGGLFVPSYQGFSTLTRMSFSYFMVMRSK
>HlOr174like_3PF_PC
FHKRLLLLIGLWPGIDFKYRTFRVAFFYTIQLSNMLYQVIIRLINYVVSTYTWFYTSECNLQCTLY
VLSIFLQNVLGIIKYTSGHIWMKTVYTFYSEIEFDWKLLKYDKENVEILRKYAGTTHVFAVAATSK
KLGRNYWFLLFNBBLNGTVFFLIPRYKRVIKVHMIKLVXQLVTTKGDERIILVMFMYTSNMCNYGV
LTTFFGQRIIDHSSNVFYDACNSHWYDVPLDARKLYLLILQRSMKSSSFSISDLYVSSLQGLSALV
STGMSYFTFLYSVQK
>HlOr175
MGEFITYYKLSIFILSWIGLWPYKKLKFQWLYNVAISLISLSTIIPQVCRSTLNFTWVTMLMKVSS
VLMTLCAFIKYNIFWYQIDAVKENMEQVKYEWQKNRDECLEILRKYASTGKYYSNIFASLIYPSVL
ILILCHMSSFVLDVIVPLNETRTHQFPVSMEYFIDQQKYSLFITFHQWFSFLVVATIIVGIETLIV
MWLEHAASLFTLKAVLEDCAQSPHVTRRYLSANCKQNLVNAVVAHRKAIWFVDHLKDRYVFSYAIV
LLFGVVSLSINLFALLQSIFLSKKIQDTLICVLFIVSELAYMFYMNYMVQRTVDTADNIITFIYSN
NWYETSIPLQKLLLIIMMRSIDSCSYDFFGFYTASIQGFSTLLKNSVSCFMMITSMTNRSPMKL
>HlOr175like_1
MDRVHESLHENYNFTVSTMSAIGLWPYRNINIRRLQNFGINVIFFALLFLEVSLIAVANLRWTLET
FVADMISMTILLGSINKYNAFLCQEKIVKQLMEKIKYDWMTINDEGELRILKNNAYRGKGFTIIFA
LLVYPTIFTMILLLFSSKILDSLLPLNESRPSWSLIRIEYFINEQKYFYLSVIYQTVTILLFGTTY
VATEAIFTMWLQHCISLFEIVSYRIEKLIIQCNAEPYVSQKRLHRNSRKILINAVVMHRRVLQFCE
KLRIHFTLAYSCLLILGVISVSVNLFQLSQTIFLEGNIQQFLTSLSSVFSLMGYMLYINYMMQEWQ
DSSNNILTTIYNSEWYTAPLSIQKLLFLIILRTTKPPISKAVFVYTASMESFTLVKSSVSYFMVIY
SIQ
>HlOr175like_2PN_PC
MQYYNFHKNLLTAIGLWPYHNTKFRTLQNSIVSFIRFSFVAVQCLPLCTTEITVQVLLKNLLLIII
FSGSFFKYNIQWYQITRVBBTTEYFIDEKKYFIFIISHVYLSLIMHATVYYVATESXVIMWMVHTIG
LYNIIRYRIQKIVRKDLNSRLNTNIKKGVIHAIATHRTAIQFFHDVIRDSIPAYSVLLLFAITSLS
INLYRVTQAVTHLDEIEETLITLMFASGELSYMYYLHYILQKIIDHSDNLVTLIFNSNWYETSLTI
QKMLYTMVLRSSKSMLVTFFGFYYASVQGFSSLLQMSVSYFMMMSSMQ
>HlOr176N_C
METFFESRYFNYVRFCARQIGLWPFDDEKEQRIKRYVVFMFSVLYTLLQVWTLMTLKDDLKTTIAS
LPYVVSSLIITIKYFTCYLNYNMIKKLFVTIGENWMELKCKNEMNLMEKYTHDGYLYTVFFBBDTV
DSKVSVLLFCVIFIMIIAVMLTTDLIYITLTYHACAMISVTGLQHLFDDVKRMRNTEQKNTLLHVR
VAKAIRYHWKCMKYVNGLESCYTVSLFLQHIMITIVITSKLYRVYSFSMTGRANLIIVSVDIAYTL
GLLFANVYPAERLTDCGQNLYHNVRYNAHWYDTPASTQKVIQIMLQKISEPFYFGISGLFSASFE

>HlOr177
MDFLEQSCIKKYYPLLFHIGVWPFDYSKRKTVQRICFCLYQISFISVQVSVFFTTEISIDLLIDTM
PATLMCMLVVLKYATFWVKNDGVYNQAFEHMRIDCCGLKDVEEINALRRGYSKASRYSTFLLVSSC
LLLFSIVFLHTLSQTTNTITSRNETRANGLIVLTEYFVDYEQYIYPITTHVVVGIFMGFAIQIGSD
LLNMIFMMHVSGMFDAIGIVAEHLFDKYESSDGKIDENLIFHARLLRLVNMHKRNLKLFEIYKDCM
YNSGLIQLVIATIVVTNVFYLHLQMLHIISTSEGNRTKIAALLLISSYLLILYINLLPTERIAESS
GDLFFKAFSGKWYTAPVKSQKTVLFLMQKFIKPCKIEVISTVAAGSETYASIVQIGFSYFMVLYSL
QE
>HlOr177like_1
MDFLEQQSCIKKYYPLLFHMGVWPFDYSKKKTVKRICLCVYHLSFISVQVWCFILTMHINTFNYIQ
AYEHMKIDWCMLKDAEEINALNRVYSSASHCNWLLFVFSYLLTSSILLSQTLVPLTDVNANDLLVR
AEYFVDYEDYFYLITTHTVVENLCAFGMQISSDILNTIIMMHISGMFEVTGLSKEDITRLYGFFFS
RLFKIFEDCMHSSGVFQICLVTVSITTIMLKALEMMSPTGNLQNKFFTVVFIVSYYILLYIDMIPG
EKIIQSSEVLFFKAYCAKWYTTSVKSQKMLLFLMHRFMEPCRLQLKNVLAMGNEMYVVLVQTSFSY
FMVLYSLQE
>HlOr177like_2N_C
MKFFEQSCIKKCYPFLLRAGLWPFDYSKRKVVHRICLCVYHLSFISVQVFVYFTTEINLRLLIDTI
PALMIYMIVFLKYVTIWVKNEAYSNASKYNMLLFVFSFLLLLGFVFLQILPQILGIISPLTEINTN
KLLVRTEYFVDYKDYIYLITIHATIAIMCSVTMQISSDILNNIFMMHISGMFEVIGIVVEHLFDKC
KSADENIDENLIFYTRLLRIMNMHRRNLKLBBNENFEYKFFVVLIAFVYLSIMYVNLSPAEKIIQS
SEVLFFKTYCAKWYKAPVKSQKMLLFLMHRFMEPCRLKLKSLVAAGNELYATMVQTGFSYFTVLYS
LQE
>HlOr177like_3F
TLSQYNQGYEHMRIDWCKLKDVEEINTLNREYSNASKYNMLLFRKEIQYYSSRETFNPKMIDFSII
PVFSFLLLLGFVFLQILPQILGIISPLTEINTNKLLVRTEYFVDYKDYIYLITIHATIAIMCSVTM
QISSDILNNIFMMHISGMFEVIGIVVEHLFDKCKSADENIDENLIFYTRLLRIMNMHRRNLKSVSV
N
>HlOr180C
FFTKWPLDPNAPKWRHALSKIIYCLHFVYELYGILAIINGLHCCFTTLGEFVKSFVEGIFMLEMLF
NLIYYRLREMEFQRLIIQMEHFFEMSNPSYSSQLRSVYYILPTTFVIGGLLFAFVPLFSENHLTPM
NTVYHLIPREKYWGICLSYALNVGHILNAASVMFLDLLVITIIWHATCKFSILGSKLKTTNEKKLK
MWIREHQNAIDYVREVNCVVAPLAIKSTIAVALYMIVSGLVLIHVRTSSFRNIYLKVLFIEISKFS
VVAMFSMLRFLACTWAADRMTENAHSIAWDIYDSSWINASTKVRSRIVLIIQRCQRPITIHAIGFF
TAWSLKFCGQVLYTIFTFFSALKAVLRE
>HlOr181C
ICDAWPLRKRYVKFILYAIYLIIIIILMYMDLFEVLGNLVLMVENIIHNTIIVTTFFMLFLLRCNK
LIAHVIITVKQEIAEIKFRNKDEMDLYLSYHSIADKFGRYAVSTTAVVAILLYLTPMLHMLITYSG
QSNETRVYELPFPSYHVLPYKHDFRSYIIMYAFQGPLMLVGLNHITTLSLIVSLVLHVCGKFSILS
YRIQNIQIQSKDNLNRKIEDFVTDHIKLILMANSINSALEVILLIDLTQTSIRMAVLIYSALLSPE
ANLVGTFTYVLYILLVTSLLYLYSFIGERLAYESTKVTEAYYDTEWQELSVRNQKLLLLAMRSGRR
TLYLTAGKVYNFSLYGFIHIMKTSFGYVSLLRTLT
